# Multilayer UWB bandpass filter using liquid crystal polymer technology

**DOI:** 10.1038/s41598-024-66928-8

**Published:** 2024-07-08

**Authors:** Khaled Aliqab

**Affiliations:** Departement of Electrical Engineering, College of Engineering, Jouf University, Sakaka, Saudi Arabia

**Keywords:** Bandpass filter, UWB, Multilayer, Liquid crystal polymer, Engineering, Materials science

## Abstract

This proposed design presents a novel bandpass filter employing a Marchand balun to attain ultra-wideband (UWB) performance extending from 3.1 to 10.7 GHz with 6.8 GHz central frequency and 110% FBW. The UWB bandpass filter’s fractional bandwidth can be tailored owing to the diverse input/output impedances of the planar Marchand balun. This adaptability is accomplished by connecting two planar Marchand baluns consecutively, leveraging the concepts of transversal filter ideas and multilayer LCP technology resulting in 0.3 dB and 12 dB insertion and return losses respectively. In-depth guidelines for the formulation and synthesis of the UWB bandpass filter are incorporated.

## Introduction

The effectiveness of wireless communication hinges on the availability of suitable RF components. The Bandpass filter serves as a critical element in eliminating unwanted frequencies while permitting only the desired range to pass through. In recent years, Ultra-wideband (UWB) wireless communication technology has shown substantial promise in advancing contemporary indoor and handheld systems. This progress is attributed to the authorization granted by the Federal Communications Commission (FCC) in 2002, permitting unlicensed operation within the frequency range of 3.1–10.6 GHz^1^. Designing a wideband filter inherently presents more complexity compared to a narrowband filter. Researchers have encountered a significant challenge in crafting a UWB bandpass filter with minimal insertion loss, enhanced selectivity (featuring two transmission zeros), compactness, and a well-defined stopband response. Filters exhibiting outstanding performance and occupying a small physical footprint are highly coveted in the realm of UWB technology.

Drawing on concepts from transversal filters, various wideband planar filters with dual transmission pathways have been successfully achieved^2,3^. Harmonic suppression is accomplished in these filter architectures by dividing the input signal into high-selectivity filtering responses, allowing propagation through several feedforward signal routes. However, adjusting the fractional bandwidth of these filter structures is not simple. Techniques like stepped impedance resonators (SIR)^4^, metamaterials^5^, a highly small Ultra-Wideband Bandpass Filter (BPF) described in reference^6^ utilizing a tapered microstrip line and an open-loop Defected Ground Structure (DGS), and others^7–12^.

Here, a novel ultra-wideband bandpass filter is introduced, utilizing the cascading of two planar Marchand baluns. Due to the varying input and output impedances (Z_in_ and Z_out_) of the planar Marchand balun, the fractional bandwidth of the UWB filter can be adjusted. A robust agreement is observed when comparing the predicted and actual outcomes.

## Filter design

**Figure 1:**
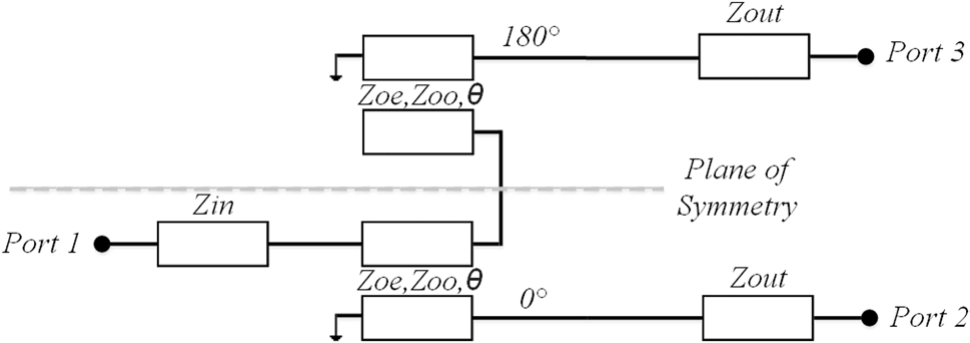
Circuit-model of Marchand balun.

Figure 1 illustrates the planar Marchand balun circuit design. Ideally, when a signal is transmitted from port 1 to ports 2 and 3, achieving an equal power split with a phase difference of 180° (S_21_ = − S_31_) is feasible. Additionally, it is imperative to satisfy the following requirement as well^4,13^:

1 $$ S_{11} = {{\left[ {1 - K^{2} \left( {\frac{{2Z_{out} }}{{Z_{in} }} + 1} \right)} \right]} \mathord{\left/ {\vphantom {{\left[ {1 - K^{2} \left( {\frac{{2Z_{out} }}{{Z_{in} }} + 1} \right)} \right]} {\left[ {1 + K^{2} \left( {\frac{{2Z_{out} }}{{Z_{in} }} - 1} \right)} \right]}}} \right. \kern-0pt} {\left[ {1 + K^{2} \left( {\frac{{2Z_{out} }}{{Z_{in} }} - 1} \right)} \right]}} = 0 $$

Where

2 $$ K = \left( {Z_{oe} - Z_{oo} } \right)/\left( {Z_{oe} + Z_{oo} } \right) $$

For various values of Z_in_ and Z_out_, the connection between Z_oe_ and Z_oo_ can be expressed more simply as:

3 $$ K = \left( {Z_{oe} - Z_{oo} } \right)/\left( {Z_{oe} + Z_{oo} } \right) = {1 \mathord{\left/ {\vphantom {1 {\sqrt {\frac{{2Z_{out} }}{{Z_{in} }} + 1} }}} \right. \kern-0pt} {\sqrt {\frac{{2Z_{out} }}{{Z_{in} }} + 1} }} $$

**Figure 2:**
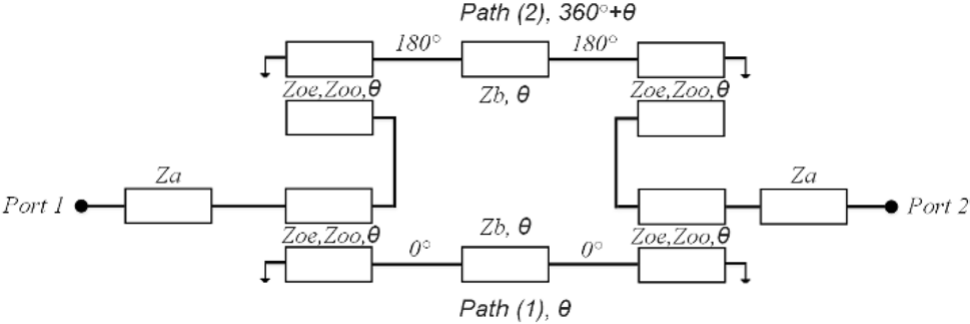
Circuit-model of cascaded balun-based filter.

**Figure 3:**
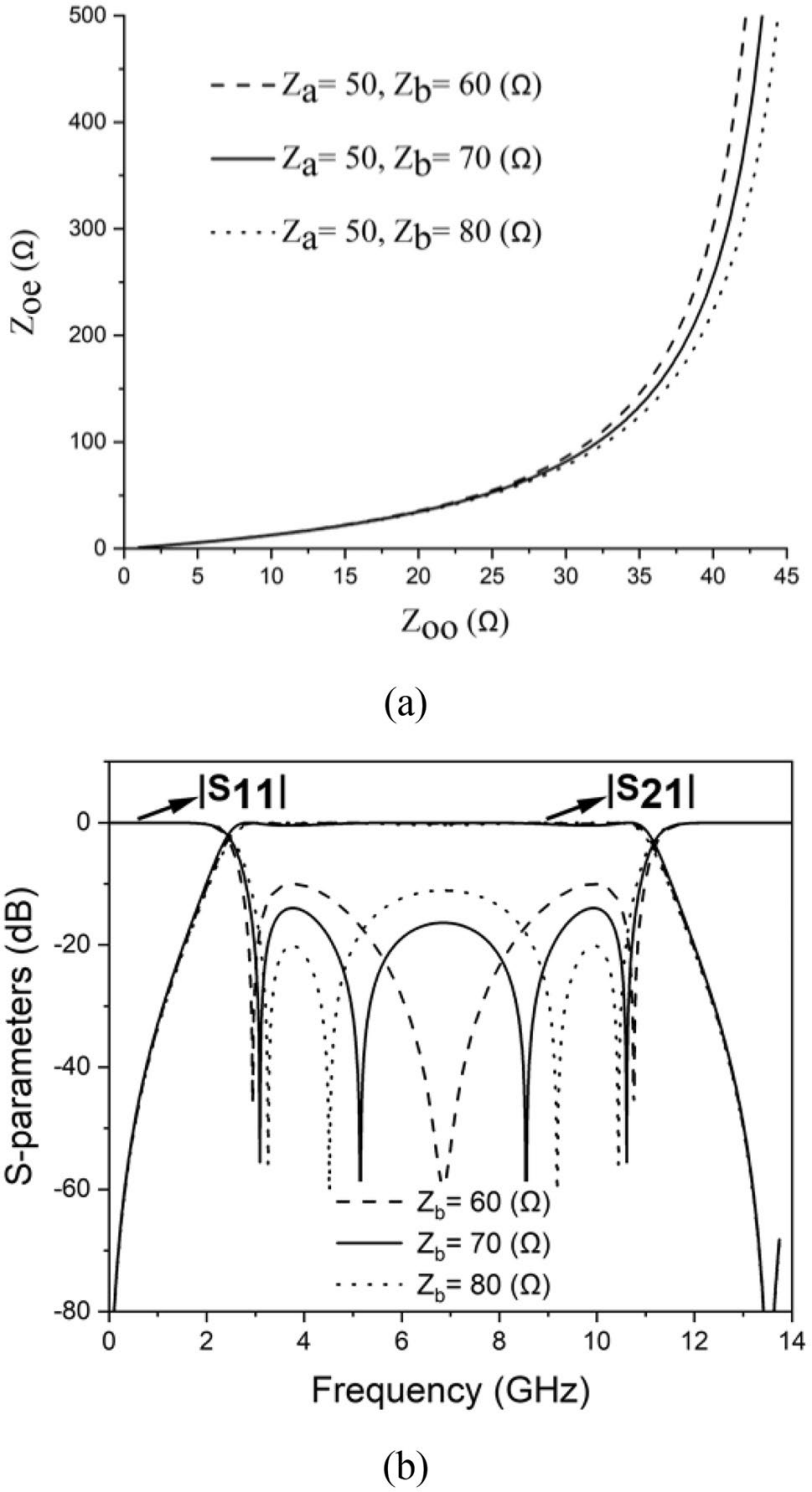
(**a**) Relationship between Zoe and Zoo for varying output impedance. (**b**) Circuit-model frequency response of the proposed design.

Figure 2 depicts the suggested design, which involves a cascade of two baluns, resulting in a 2-port filter. The ideal relationship between Z_oe_ and Z_oo_, considering varying Z_in_ (Z_a_) and Z_out_ (Z_b_), is presented in Fig. 3. There is not a singular set of coupled line parameters for a specific Z_in_ and Z_out_ combination. The design’s response varies based on the values of Z_oe_ and Z_oo_, as indicated in the same figure. In Fig. 3, we can observe the simulated frequency response of the design. In accordance with^5^, specific criteria were applied to achieve a passband:

4 $$ \theta_{1} \left( {f_{0} } \right) = \theta_{2} \left( {f_{0} } \right) \pm 2n\pi , \quad \left( {n = 0,1,2 \ldots } \right) $$

In this setup, the central frequency of the filter is represented as *f*_*0*_. As illustrated in Fig. 2, a UWB bandpass filter utilizes a series of two planar Marchand baluns. The output ports of these two balun structures are connected through two transmission lines with an electrical length denoted as θ. Due to the inherent 180° phase difference of the Marchand balun, the value of *θ*_2_ (*f*_0_) = *θ*1 (*f*1) + 360° can be achieved at *f*_0_. The frequency responses depicted in Fig. 3b correspond to the simulated data of the schematic illustrated in Fig. 2. To examine the impact of Z_b_ on the matching bandwidth response, three distinct values of Z_b_ (60, 70, and 80 Ω) were tested, while keeping Z_a_ fixed at 50 Ω and Z_oo_ fixed at 38 Ω. These Z_b_ values corresponded to Z_oe_ values of 159, 186, and 217 Ω, respectively, as depicted in Fig. 3a. Figure 3b illustrates that a Z_b_ value of 60 produces a passband response with a single deep transmission pole at the center, as indicated by S_11_. Conversely, a Z_b_ value of 70 results in an equal-ripple four transmission poles resembling the Chebyshev response in the passband, with a matching bandwidth of 17 dB. Further increasing Z_b_ adversely affects the matching bandwidth. Consequently, the intentional selection of Z_b_ = 70 Ω was employed for this design.

The connection between Z_oe_ and Z_oo_ is distinctly characterized as rational and favorable, contrasting with the linear characteristic arising from the utilization of a microstrip quasi-TEM structure. The nature of the relationship between Z_oe_ and Z_oo_ is predominantly influenced by the type of structure employed. Specifically, the TEM propagation supported by the broadside-connected stripline establishes the desired behavior, whereas the coupled microstrip quasi-TEM lines exhibit a linear relationship. The rational behavior demonstrated in this context is preferred over the linear structure due to the considerable mismatch between the impedance values of Z_oe_ and Z_oo_. This mismatch results in a more pronounced coupling, which is essential for an Ultra-Wideband (UWB) design. However, it is noteworthy that non-TEM or quasi-TEM coupled line configurations can also significantly impact the wideband response. Primarily due to the disparate phase velocities of the even and odd modes.

Attaining optimal UWB performance requires a substantial coupling factor between the connected lines. This necessitates a high impedance ratio between Z_oe_ and Z_oo_, thereby requiring narrow coupling gaps. Additionally, it is crucial to ensure that the odd and even modes of the tightly coupled line possess identical phase velocities. Achieving this, however, is challenging when employing edge-coupled, quasi-TEM transmission line configurations, commonly utilized in earlier studies. As a result, a broadside TEM coupling arrangement is employed in this case.

The 60 Ω output line illustrated in Fig. 3a exhibits various combinations of Z_oe_ and Z_oo_. However, certain options may be impractical, excessively challenging to obtain, or unsuitable in terms of performance and physical implementation. In a stripline configuration, the width and vertical gap spacing of the two connected lines are the primary factors determining the (Z_oe_, Z_oo_) values. Through adjusting the vertical gap spacing to 100 μm and optimizing the width, it was determined that a width of 0.3 mm achieves the ideal combination of Z_oe_ = 186 Ω and Z_oo_ = 38 Ω. Furthermore, this line width provides a favorable margin of error to address any complications that may arise during the production process. Therefore, this proposal recommends the utilization of LCP bonded PCB multilayer technology to achieve the necessary vertical tight coupling and a minimal vertical distance of 100 μm.

**Figure 4:**
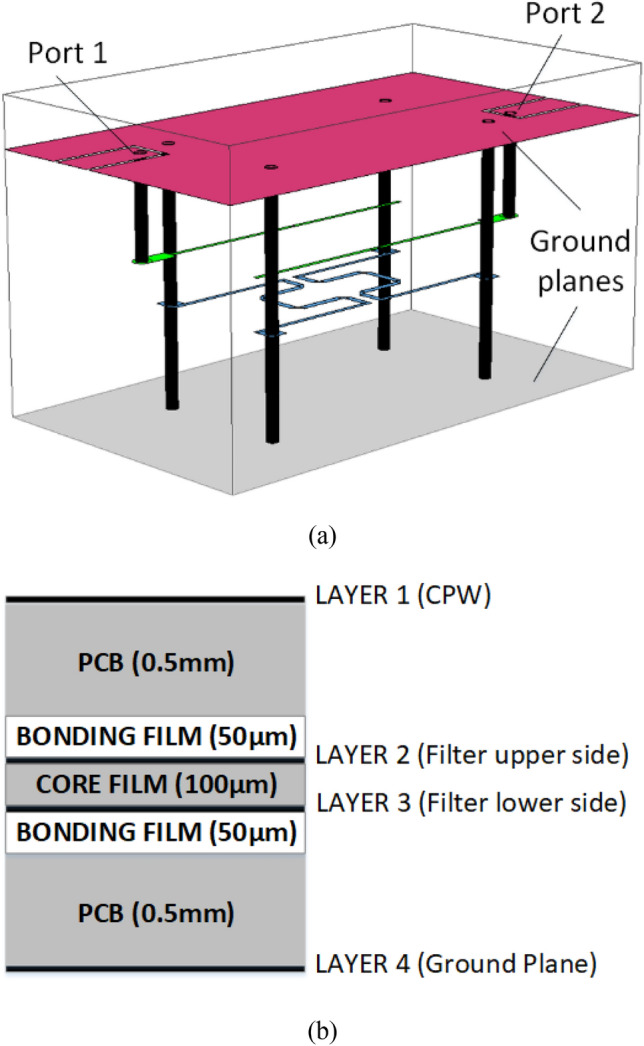
Multilayer cascaded filter (**a**) 3D view of the proposed design. (**b**) Cross-sectional view of the layer stack implemented.

The proposed work adopts a stripline configuration, where the entire layout is encapsulated within a custom-designed package integral to the production process of the hosted design. Figure 4 provides a three-dimensional perspective of the proposed design, revealing its layer stack. In Fig. 4a, Layer 1 functions as the interface between the enclosed design and the external system. It also serves as the first ground plane for the enclosed stripline arrangement. The device features input and output lines specifically designed to maintain a 50-Ω impedance in a CPW structure. In this configuration, the signal line and ground are situated at the same level with a small gap between them. Via holes connect the feeding lines to the hosted board and are employed for grounding purposes, as demonstrated in Layer 3 concerning the suggested design topology shown in Fig. 2.

Layers 2 and 3 illustrate the configuration of the proposed filter, highlighting the evident presence of broadside coupling between the two quarter-wavelength coupled-line segments. The etching process is employed to fabricate these structures on a single substrate with a thickness of 100 μm, aiming to achieve the desired high coupling factor. Layer 4 is entirely metallized and acts as the ground plane for the grounded coplanar waveguide (CPW) construction. It also serves as the second ground plane for the enclosed stripline arrangement.

Upon transmission over the unbalanced 50Ω input feed line from CPW to the stripline level, the signal is also linked to the lower quarter-wavelength lines at Layer 3. Although the magnitude of the signal remains unchanged, a 180° phase shift occurs. The differential signal is then transferred from the stripline level to the CPW level through via holes, ensuring a balanced signal output.

**Figure 5:**
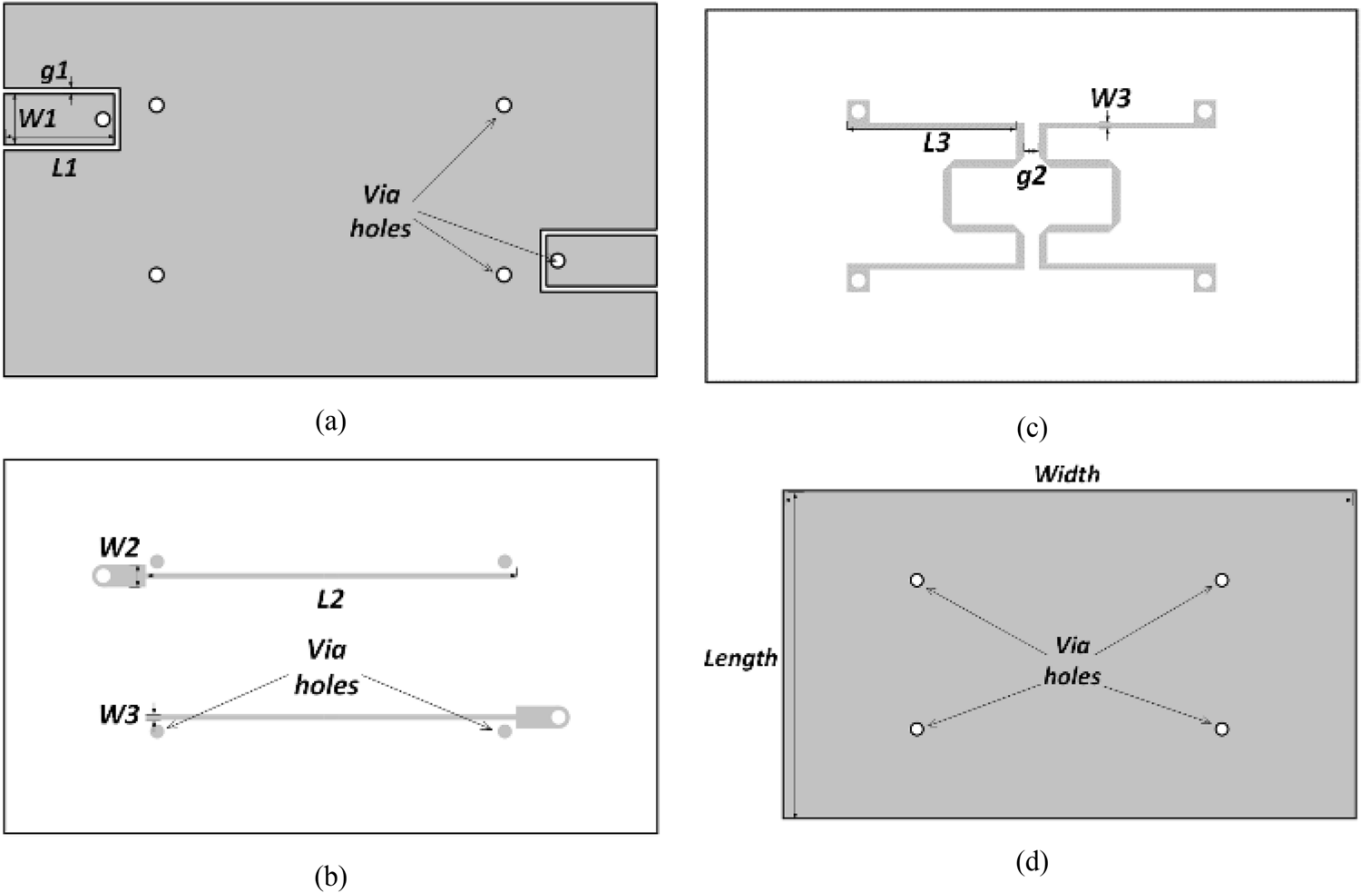
Filter’s layers dimensions (**a**) Layer 1. (**b**) Layer 2. (**c**) Layer 3. (**d**) Layer 4.

The investigations were conducted using two distinct substrates, as depicted in Fig. 4. PCB layers at Layers 1 and 4 are composed of Rogers RO3003 substrate, characterized by a dielectric constant of 3.0, a loss tangent of 0.0025, and a thickness of 0.5 mm. In Layers 2 and 3, both the core and bonding films of the LCP film share identical dielectric constants and loss tangents as the PCB. However, the LCP film has a thickness of 100 μm. The PCB and LCP were joined together using an LCP bonding film, possessing similar properties to the LCP core film but with a thickness of 50 μm, eliminating the need for additional adhesives. A multilayer lamination technique was devised to execute the proposed prototype. Metal tracks were etched on the LCP core film, with a melting temperature of 315 °C, while the LCP bonding film has a melting temperature of 280 °C. Figure 5 illustrates the distinct design layers and their respective dimensions employed in creating the prototype. The final dimensions of the objects are as follows: L1 = 3 mm, W1 = 1.8 mm, W2 = 0.8 mm, g1 = 0.2 mm, L2 = 13.1 mm, W3 = 0.2 mm, L3 = 6 mm, g2 = 0.5 mm. The width is 20 mm, and the length is 10 mm. The diameter of the via holes used for input and output feeding lines is 0.5 mm, whereas the diameter of the grounding via holes is 1.0 mm.

## Implementation and experiment

In consideration of the information provided, the proposed concept was developed, simulated, and fabricated, resulting in an ultra-wideband multilayer filter (UWB) operating at 6.9 GHz with a fractional bandwidth (FBW) greater than 100%.

**Figure 6:**
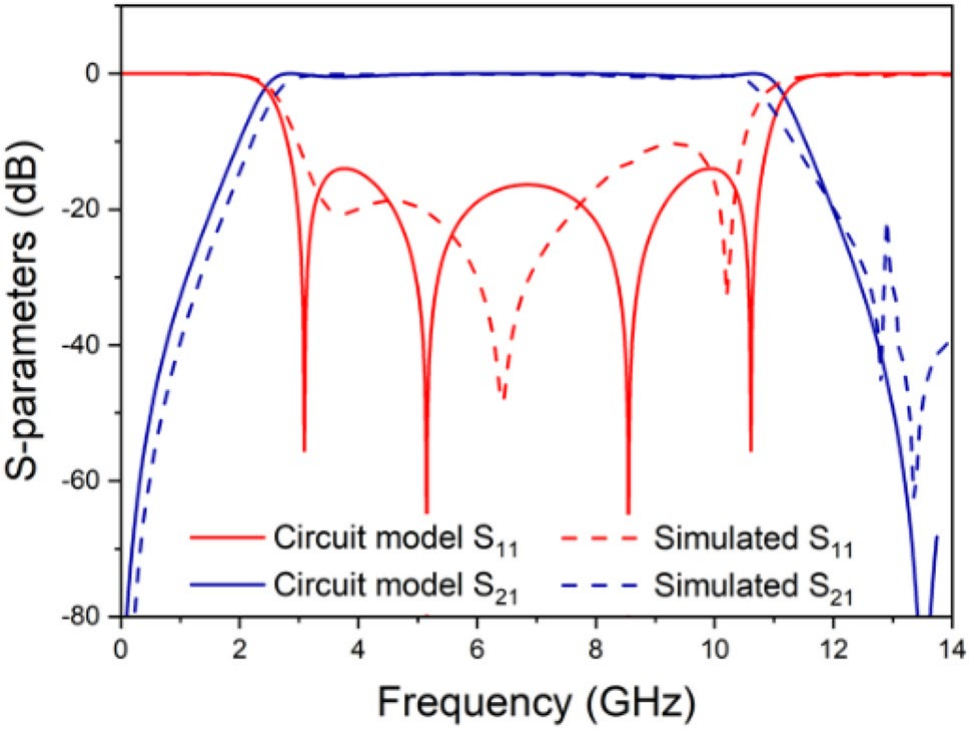
Circuit-model and simulated frequency responses.

The simulated results for the UWB filter are carried out using Sonnet Software and presented in Fig. 6. The insertion loss is maintained below 0.3 dB, and the return loss surpasses 12 dB. Furthermore, the group delay remains below 0.4 ns within the frequency range of 3.1–10.6 GHz.

**Figure 7:**
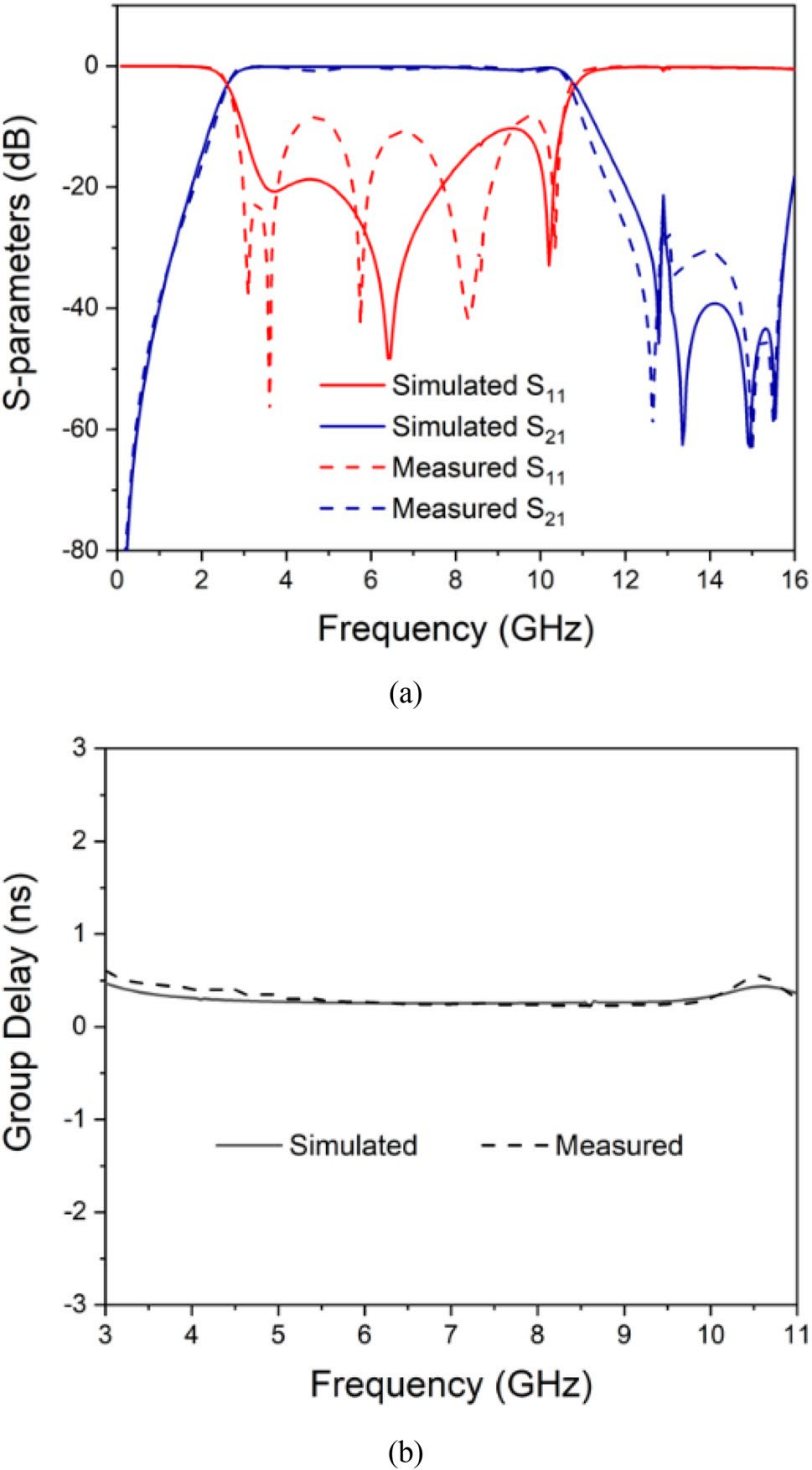
(**a**) Simulated and measured frequency response. (**b**) Group delay.

The measured results are illustrated in Fig. 7. The central frequency is determined to be 6.8 GHz, with a fractional bandwidth (FBW) of 110%, spanning from 3.1 to 10.7 GHz. In the considered passband, the insertion and return losses are measured at 1.2 dB and 10.8 dB, respectively, while the group delay is recorded to be less than 0.5 ns.

The slight frequency variation observed in the measured results may be attributed to the precision of the fabrication process and potential discrepancies in the measurement procedure. The final measurements were conducted using an N5225A PNA Microwave Network Analyzer, and the filter was manufactured in-house.

**Figure 8:**
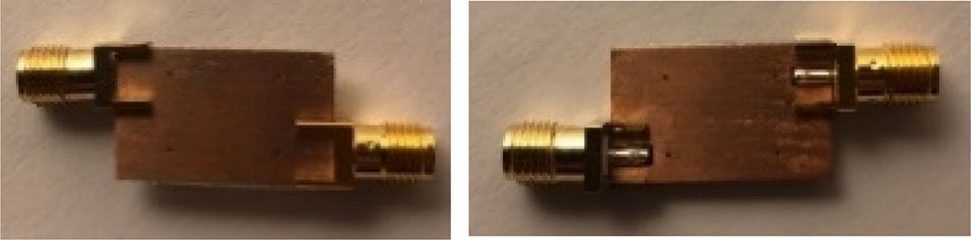
Photographs of the fabricated filter.

The images of the constructed filter prototype are presented in Fig. 8, showcasing both the front and back sides of the design.

## Conclusion

This paper introduces an innovative ultra-wideband filter leveraging the planar Marchand balun and grounded in the principles of transversal filters. The filter showcases the advantages of a straightforward topology, robust frequency performance, and consistent group delay, rendering the proposed UWB filter highly attractive for applications in UWB radio communication.

### Supplementary Information

Supplementary Information 1.

Supplementary Information 2.

Supplementary Information 3.

Supplementary Information 4.

## Data Availability

All data generated or analysed during this study are included in this published article [and its supplementary information files].
