# Supplementary material for: Multilayer UWB bandpass filter using liquid crystal polymer technology
Source: Sci Rep. 2024 Jul 8;14:15669. doi: 10.1038/s41598-024-66928-8 (PMC11231142; doi:10.1038/s41598-024-66928-8)
Supplement: Supplementary file 1 — Supplementary Information 1. [file 41598_2024_66928_MOESM1_ESM.pdf]

two baluns_60 ohms Data File

Frequency (	DB[S11]	DB[S21]	DB[S11]	DB[S21]	DB[S11]	DB[S21]
0	-0.01042	-99.7382	-0.01042	-100.085	-0.01042	-100.417
0.01	0	-96.9899	1.93E-15	-97.3362	-9.64E-16	-97.6692
0.02	-5.30E-14	-94.5031	-5.01E-14	-94.8495	-4.92E-14	-95.1825
0.03	-6.32E-13	-92.2323	-5.82E-13	-92.5788	-5.40E-13	-92.9118
0.04	-3.55E-12	-90.1428	-3.27E-12	-90.4893	-3.03E-12	-90.8224
0.05	-1.35E-11	-88.2076	-1.25E-11	-88.5542	-1.16E-11	-88.8873
0.06	-4.04E-11	-86.4054	-3.73E-11	-86.752	-3.46E-11	-87.0852
0.07	-1.02E-10	-84.7189	-9.42E-11	-85.0657	-8.72E-11	-85.3989
0.08	-2.27E-10	-83.1342	-2.10E-10	-83.481	-1.94E-10	-83.8143
0.09	-4.61E-10	-81.6394	-4.26E-10	-81.9863	-3.94E-10	-82.3197
0.1	-8.69E-10	-80.2249	-8.02E-10	-80.5719	-7.43E-10	-80.9053
0.11	-1.54E-09	-78.8824	-1.42E-09	-79.2295	-1.32E-09	-79.5629
0.12	-2.60E-09	-77.6047	-2.40E-09	-77.952	-2.22E-09	-78.2855
0.13	-4.20E-09	-76.386	-3.88E-09	-76.7333	-3.59E-09	-77.0669
0.14	-6.56E-09	-75.2208	-6.06E-09	-75.5683	-5.61E-09	-75.9019
0.15	-9.94E-09	-74.1046	-9.17E-09	-74.4522	-8.50E-09	-74.7859
0.16	-1.47E-08	-73.0334	-1.35E-08	-73.3811	-1.25E-08	-73.7149
0.17	-2.11E-08	-72.0036	-1.95E-08	-72.3515	-1.80E-08	-72.6853
0.18	-2.98E-08	-71.0121	-2.75E-08	-71.3601	-2.55E-08	-71.694
0.19	-4.12E-08	-70.056	-3.81E-08	-70.4041	-3.53E-08	-70.7382
0.2	-5.62E-08	-69.1329	-5.19E-08	-69.4812	-4.80E-08	-69.8153
0.21	-7.54E-08	-68.2404	-6.96E-08	-68.5889	-6.45E-08	-68.9231
0.22	-9.98E-08	-67.3767	-9.21E-08	-67.7253	-8.53E-08	-68.0596
0.23	-1.31E-07	-66.5397	-1.20E-07	-66.8885	-1.12E-07	-67.2229
0.24	-1.69E-07	-65.7279	-1.56E-07	-66.0769	-1.44E-07	-66.4114
0.25	-2.16E-07	-64.9397	-1.99E-07	-65.2889	-1.85E-07	-65.6235
0.26	-2.74E-07	-64.1738	-2.53E-07	-64.5231	-2.34E-07	-64.8578
0.27	-3.44E-07	-63.4288	-3.18E-07	-63.7783	-2.94E-07	-64.1132
0.28	-4.29E-07	-62.7036	-3.96E-07	-63.0534	-3.66E-07	-63.3883
0.29	-5.30E-07	-61.9972	-4.89E-07	-62.3471	-4.53E-07	-62.6822
0.3	-6.51E-07	-61.3085	-6.01E-07	-61.6586	-5.57E-07	-61.9938
0.31	-7.95E-07	-60.6366	-7.33E-07	-60.987	-6.79E-07	-61.3223
0.32	-9.63E-07	-59.9807	-8.89E-07	-60.3313	-8.23E-07	-60.6667
0.33	-1.16E-06	-59.3399	-1.07E-06	-59.6908	-9.92E-07	-60.0264
0.34	-1.39E-06	-58.7137	-1.28E-06	-59.0647	-1.19E-06	-59.4005
0.35	-1.66E-06	-58.1012	-1.53E-06	-58.4525	-1.42E-06	-58.7884
0.36	-1.97E-06	-57.5018	-1.82E-06	-57.8534	-1.68E-06	-58.1895
0.37	-2.33E-06	-56.915	-2.15E-06	-57.2669	-1.99E-06	-57.6031
0.38	-2.74E-06	-56.3402	-2.53E-06	-56.6924	-2.34E-06	-57.0287
0.39	-3.21E-06	-55.7769	-2.96E-06	-56.1293	-2.74E-06	-56.4658
0.4	-3.75E-06	-55.2246	-3.46E-06	-55.5773	-3.20E-06	-55.914
0.41	-4.36E-06	-54.6828	-4.02E-06	-55.0358	-3.72E-06	-55.3726
0.42	-5.06E-06	-54.1511	-4.66E-06	-54.5044	-4.32E-06	-54.8414
0.43	-5.84E-06	-53.6291	-5.39E-06	-53.9827	-4.99E-06	-54.3199
0.44	-6.72E-06	-53.1164	-6.20E-06	-53.4703	-5.74E-06	-53.8077
0.45	-7.72E-06	-52.6127	-7.12E-06	-52.9669	-6.59E-06	-53.3045

0.46	-8.84E-06	-52.1176	-8.15E-06	-52.4721	-7.54E-06	-52.8099
0.47	-1.01E-05	-51.6308	-9.30E-06	-51.9856	-8.61E-06	-52.3236
0.48	-1.15E-05	-51.1519	-1.06E-05	-51.5071	-9.80E-06	-51.8453
0.49	-1.30E-05	-50.6808	-1.20E-05	-51.0363	-1.11E-05	-51.3748
0.5	-1.48E-05	-50.217	-1.36E-05	-50.5729	-1.26E-05	-50.9116
0.51	-1.67E-05	-49.7604	-1.54E-05	-50.1167	-1.42E-05	-50.4556
0.52	-1.88E-05	-49.3108	-1.74E-05	-49.6674	-1.61E-05	-50.0065
0.53	-2.12E-05	-48.8678	-1.95E-05	-49.2248	-1.81E-05	-49.5642
0.54	-2.38E-05	-48.4312	-2.19E-05	-48.7887	-2.03E-05	-49.1282
0.55	-2.67E-05	-48.0009	-2.46E-05	-48.3587	-2.27E-05	-48.6986
0.56	-2.98E-05	-47.5766	-2.75E-05	-47.9349	-2.54E-05	-48.2749
0.57	-3.33E-05	-47.1581	-3.07E-05	-47.5168	-2.84E-05	-47.8571
0.58	-3.71E-05	-46.7453	-3.42E-05	-47.1044	-3.16E-05	-47.445
0.59	-4.13E-05	-46.338	-3.81E-05	-46.6975	-3.52E-05	-47.0384
0.6	-4.59E-05	-45.9359	-4.23E-05	-46.2959	-3.91E-05	-46.637
0.61	-5.09E-05	-45.539	-4.69E-05	-45.8994	-4.34E-05	-46.2409
0.62	-5.64E-05	-45.147	-5.19E-05	-45.508	-4.80E-05	-45.8497
0.63	-6.23E-05	-44.7599	-5.74E-05	-45.1213	-5.31E-05	-45.4633
0.64	-6.88E-05	-44.3774	-6.34E-05	-44.7394	-5.86E-05	-45.0817
0.65	-7.59E-05	-43.9996	-6.99E-05	-44.362	-6.46E-05	-44.7046
0.66	-8.36E-05	-43.6261	-7.69E-05	-43.989	-7.11E-05	-44.3319
0.67	-9.19E-05	-43.2569	-8.46E-05	-43.6203	-7.82E-05	-43.9636
0.68	-0.0001	-42.8918	-9.29E-05	-43.2558	-8.59E-05	-43.5994
0.69	-0.00011	-42.5308	-0.0001	-42.8954	-9.42E-05	-43.2393
0.7	-0.00012	-42.1738	-0.00011	-42.5389	-0.0001	-42.8831
0.71	-0.00013	-41.8205	-0.00012	-42.1862	-0.00011	-42.5308
0.72	-0.00015	-41.471	-0.00013	-41.8373	-0.00012	-42.1822
0.73	-0.00016	-41.1251	-0.00015	-41.4919	-0.00013	-41.8373
0.74	-0.00017	-40.7827	-0.00016	-41.1501	-0.00015	-41.4958
0.75	-0.00019	-40.4437	-0.00017	-40.8118	-0.00016	-41.1579
0.76	-0.00021	-40.1081	-0.00019	-40.4768	-0.00017	-40.8233
0.77	-0.00022	-39.7757	-0.00021	-40.145	-0.00019	-40.4919
0.78	-0.00024	-39.4465	-0.00022	-39.8165	-0.00021	-40.1638
0.79	-0.00026	-39.1203	-0.00024	-39.491	-0.00022	-39.8387
0.8	-0.00029	-38.7971	-0.00026	-39.1685	-0.00024	-39.5166
0.81	-0.00031	-38.4769	-0.00028	-38.849	-0.00026	-39.1975
0.82	-0.00034	-38.1595	-0.00031	-38.5323	-0.00028	-38.8813
0.83	-0.00036	-37.8449	-0.00033	-38.2184	-0.00031	-38.5679
0.84	-0.00039	-37.533	-0.00036	-37.9072	-0.00033	-38.2572
0.85	-0.00042	-37.2237	-0.00039	-37.5987	-0.00036	-37.9491
0.86	-0.00046	-36.917	-0.00042	-37.2928	-0.00039	-37.6437
0.87	-0.00049	-36.6128	-0.00045	-36.9894	-0.00042	-37.3408
0.88	-0.00053	-36.3111	-0.00049	-36.6885	-0.00045	-37.0404
0.89	-0.00057	-36.0117	-0.00053	-36.3899	-0.00049	-36.7423
0.9	-0.00062	-35.7147	-0.00057	-36.0937	-0.00052	-36.4467
0.91	-0.00066	-35.4199	-0.00061	-35.7998	-0.00056	-36.1533
0.92	-0.00071	-35.1274	-0.00065	-35.5082	-0.0006	-35.8622
0.93	-0.00077	-34.837	-0.0007	-35.2187	-0.00065	-35.5733

0.94	-0.00082	-34.5488	-0.00076	-34.9313	-0.0007	-35.2865
0.95	-0.00088	-34.2626	-0.00081	-34.6461	-0.00075	-35.0018
0.96	-0.00095	-33.9784	-0.00087	-34.3628	-0.0008	-34.7192
0.97	-0.00102	-33.6962	-0.00093	-34.0816	-0.00086	-34.4386
0.98	-0.00109	-33.4159	-0.001	-33.8023	-0.00092	-34.1599
0.99	-0.00117	-33.1375	-0.00107	-33.5249	-0.00098	-33.8831
1	-0.00125	-32.8609	-0.00114	-33.2493	-0.00105	-33.6082
1.01	-0.00133	-32.5861	-0.00122	-32.9755	-0.00113	-33.3351
1.02	-0.00143	-32.313	-0.00131	-32.7035	-0.0012	-33.0637
1.03	-0.00152	-32.0417	-0.0014	-32.4333	-0.00129	-32.7942
1.04	-0.00163	-31.772	-0.00149	-32.1647	-0.00137	-32.5263
1.05	-0.00174	-31.5039	-0.00159	-31.8977	-0.00147	-32.2601
1.06	-0.00185	-31.2375	-0.0017	-31.6324	-0.00156	-31.9955
1.07	-0.00198	-30.9725	-0.00181	-31.3687	-0.00167	-31.7325
1.08	-0.00211	-30.7091	-0.00193	-31.1065	-0.00178	-31.4711
1.09	-0.00225	-30.4472	-0.00206	-30.8458	-0.00189	-31.2111
1.1	-0.00239	-30.1867	-0.00219	-30.5865	-0.00202	-30.9527
1.11	-0.00255	-29.9277	-0.00233	-30.3287	-0.00215	-30.6957
1.12	-0.00271	-29.67	-0.00248	-30.0723	-0.00228	-30.4402
1.13	-0.00289	-29.4136	-0.00264	-29.8173	-0.00243	-30.186
1.14	-0.00307	-29.1586	-0.00281	-29.5636	-0.00258	-29.9332
1.15	-0.00327	-28.9049	-0.00298	-29.3113	-0.00274	-29.6817
1.16	-0.00347	-28.6524	-0.00317	-29.0602	-0.00292	-29.4315
1.17	-0.00369	-28.4011	-0.00337	-28.8103	-0.0031	-29.1826
1.18	-0.00392	-28.1511	-0.00358	-28.5617	-0.00329	-28.9349
1.19	-0.00416	-27.9022	-0.0038	-28.3143	-0.00349	-28.6885
1.2	-0.00442	-27.6544	-0.00403	-28.0681	-0.0037	-28.4432
1.21	-0.00469	-27.4078	-0.00427	-27.823	-0.00393	-28.1991
1.22	-0.00497	-27.1622	-0.00453	-27.579	-0.00416	-27.9561
1.23	-0.00527	-26.9177	-0.0048	-27.3361	-0.00441	-27.7143
1.24	-0.00559	-26.6743	-0.00509	-27.0942	-0.00468	-27.4735
1.25	-0.00593	-26.4318	-0.0054	-26.8535	-0.00495	-27.2338
1.26	-0.00628	-26.1903	-0.00572	-26.6137	-0.00525	-26.9952
1.27	-0.00665	-25.9498	-0.00605	-26.3749	-0.00555	-26.7575
1.28	-0.00705	-25.7103	-0.00641	-26.1371	-0.00588	-26.5209
1.29	-0.00746	-25.4716	-0.00678	-25.9003	-0.00622	-26.2852
1.3	-0.0079	-25.2338	-0.00718	-25.6643	-0.00658	-26.0505
1.31	-0.00836	-24.9969	-0.00759	-25.4293	-0.00696	-25.8167
1.32	-0.00884	-24.7609	-0.00803	-25.1952	-0.00736	-25.5838
1.33	-0.00935	-24.5257	-0.00849	-24.9619	-0.00778	-25.3518
1.34	-0.00989	-24.2912	-0.00897	-24.7295	-0.00822	-25.1207
1.35	-0.01045	-24.0576	-0.00948	-24.4978	-0.00868	-24.8904
1.36	-0.01105	-23.8247	-0.01002	-24.267	-0.00917	-24.6609
1.37	-0.01168	-23.5926	-0.01058	-24.037	-0.00969	-24.4323
1.38	-0.01234	-23.3612	-0.01118	-23.8078	-0.01023	-24.2045
1.39	-0.01303	-23.1305	-0.0118	-23.5793	-0.0108	-23.9774
1.4	-0.01377	-22.9005	-0.01246	-23.3515	-0.01139	-23.7511
1.41	-0.01454	-22.6711	-0.01315	-23.1244	-0.01202	-23.5255

1.42	-0.01535	-22.4424	-0.01388	-22.898	-0.01268	-23.3007
1.43	-0.0162	-22.2143	-0.01464	-22.6723	-0.01338	-23.0765
1.44	-0.0171	-21.9869	-0.01544	-22.4473	-0.01411	-22.8531
1.45	-0.01804	-21.76	-0.01629	-22.2229	-0.01487	-22.6303
1.46	-0.01903	-21.5337	-0.01718	-21.9991	-0.01568	-22.4082
1.47	-0.02008	-21.308	-0.01811	-21.7759	-0.01653	-22.1867
1.48	-0.02117	-21.0828	-0.01909	-21.5534	-0.01741	-21.9659
1.49	-0.02233	-20.8582	-0.02012	-21.3314	-0.01835	-21.7456
1.5	-0.02354	-20.6341	-0.0212	-21.11	-0.01933	-21.526
1.51	-0.02482	-20.4104	-0.02234	-20.8891	-0.02036	-21.307
1.52	-0.02616	-20.1873	-0.02354	-20.6688	-0.02144	-21.0885
1.53	-0.02757	-19.9646	-0.02479	-20.449	-0.02257	-20.8706
1.54	-0.02906	-19.7424	-0.02611	-20.2297	-0.02377	-20.6533
1.55	-0.03062	-19.5206	-0.02749	-20.0109	-0.02502	-20.4365
1.56	-0.03225	-19.2993	-0.02895	-19.7926	-0.02633	-20.2202
1.57	-0.03398	-19.0783	-0.03048	-19.5747	-0.02771	-20.0044
1.58	-0.03579	-18.8578	-0.03208	-19.3574	-0.02915	-19.7891
1.59	-0.03769	-18.6376	-0.03377	-19.1404	-0.03067	-19.5743
1.6	-0.03969	-18.4179	-0.03553	-18.9239	-0.03226	-19.36
1.61	-0.0418	-18.1985	-0.03739	-18.7079	-0.03393	-19.1461
1.62	-0.04401	-17.9794	-0.03934	-18.4922	-0.03569	-18.9327
1.63	-0.04633	-17.7607	-0.04139	-18.277	-0.03753	-18.7198
1.64	-0.04877	-17.5423	-0.04354	-18.0621	-0.03946	-18.5073
1.65	-0.05134	-17.3242	-0.04579	-17.8477	-0.04148	-18.2952
1.66	-0.05403	-17.1065	-0.04816	-17.6336	-0.0436	-18.0836
1.67	-0.05687	-16.8891	-0.05065	-17.4199	-0.04583	-17.8724
1.68	-0.05984	-16.6719	-0.05326	-17.2065	-0.04817	-17.6615
1.69	-0.06297	-16.455	-0.056	-16.9935	-0.05062	-17.4511
1.7	-0.06626	-16.2385	-0.05888	-16.7809	-0.05319	-17.2411
1.71	-0.06971	-16.0221	-0.06189	-16.5686	-0.05589	-17.0314
1.72	-0.07335	-15.8061	-0.06506	-16.3566	-0.05871	-16.8221
1.73	-0.07716	-15.5903	-0.06839	-16.1449	-0.06168	-16.6132
1.74	-0.08117	-15.3747	-0.07188	-15.9336	-0.06479	-16.4047
1.75	-0.08539	-15.1594	-0.07554	-15.7226	-0.06805	-16.1965
1.76	-0.08982	-14.9444	-0.07939	-15.5119	-0.07147	-15.9887
1.77	-0.09447	-14.7295	-0.08342	-15.3015	-0.07505	-15.7813
1.78	-0.09937	-14.5149	-0.08766	-15.0914	-0.07882	-15.5742
1.79	-0.10451	-14.3006	-0.09211	-14.8816	-0.08276	-15.3675
1.8	-0.10992	-14.0865	-0.09677	-14.6722	-0.08689	-15.1611
1.81	-0.1156	-13.8726	-0.10167	-14.463	-0.09123	-14.9551
1.82	-0.12157	-13.6589	-0.10681	-14.2541	-0.09577	-14.7494
1.83	-0.12785	-13.4455	-0.11221	-14.0455	-0.10054	-14.544
1.84	-0.13445	-13.2323	-0.11787	-13.8373	-0.10554	-14.339
1.85	-0.14138	-13.0193	-0.12381	-13.6293	-0.11077	-14.1344
1.86	-0.14868	-12.8066	-0.13005	-13.4217	-0.11627	-13.9301
1.87	-0.15635	-12.5941	-0.1366	-13.2143	-0.12203	-13.7262
1.88	-0.16441	-12.3819	-0.14347	-13.0073	-0.12806	-13.5226
1.89	-0.17288	-12.1699	-0.15069	-12.8006	-0.13439	-13.3194

1.9	-0.1818	-11.9582	-0.15826	-12.5942	-0.14103	-13.1166
1.91	-0.19117	-11.7468	-0.16621	-12.3882	-0.14799	-12.9142
1.92	-0.20103	-11.5357	-0.17455	-12.1825	-0.15528	-12.7121
1.93	-0.21139	-11.3249	-0.18331	-11.9772	-0.16293	-12.5104
1.94	-0.22229	-11.1144	-0.19251	-11.7722	-0.17095	-12.3092
1.95	-0.23376	-10.9042	-0.20216	-11.5677	-0.17935	-12.1083
1.96	-0.24582	-10.6944	-0.21229	-11.3635	-0.18817	-11.9079
1.97	-0.2585	-10.4849	-0.22292	-11.1597	-0.1974	-11.708
1.98	-0.27184	-10.2759	-0.23409	-10.9564	-0.20709	-11.5084
1.99	-0.28588	-10.0673	-0.24581	-10.7535	-0.21724	-11.3094
2	-0.30064	-9.85913	-0.25811	-10.5511	-0.22788	-11.1108
2.01	-0.31618	-9.65144	-0.27103	-10.3492	-0.23904	-10.9128
2.02	-0.33252	-9.44426	-0.28459	-10.1477	-0.25074	-10.7152
2.03	-0.34972	-9.23761	-0.29882	-9.94686	-0.263	-10.5182
2.04	-0.36781	-9.03155	-0.31377	-9.74655	-0.27585	-10.3218
2.05	-0.38685	-8.82609	-0.32946	-9.54683	-0.28932	-10.126
2.06	-0.40689	-8.62129	-0.34593	-9.34774	-0.30344	-9.93073
2.07	-0.42797	-8.41719	-0.36323	-9.1493	-0.31824	-9.73615
2.08	-0.45016	-8.21383	-0.38139	-8.95156	-0.33375	-9.54224
2.09	-0.47351	-8.01126	-0.40045	-8.75454	-0.35001	-9.34902
2.1	-0.49809	-7.80954	-0.42046	-8.55829	-0.36706	-9.15655
2.11	-0.52395	-7.60872	-0.44147	-8.36286	-0.38492	-8.96483
2.12	-0.55118	-7.40887	-0.46352	-8.16828	-0.40363	-8.77392
2.13	-0.57983	-7.21003	-0.48667	-7.9746	-0.42325	-8.58385
2.14	-0.60998	-7.01228	-0.51098	-7.78186	-0.4438	-8.39465
2.15	-0.64172	-6.81569	-0.53649	-7.59013	-0.46534	-8.20638
2.16	-0.67513	-6.62033	-0.56327	-7.39945	-0.48791	-8.01906
2.17	-0.71028	-6.42626	-0.59137	-7.20988	-0.51155	-7.83275
2.18	-0.74729	-6.23358	-0.62088	-7.02147	-0.53632	-7.6475
2.19	-0.78623	-6.04236	-0.65184	-6.83429	-0.56226	-7.46334
2.2	-0.82721	-5.85269	-0.68433	-6.6484	-0.58944	-7.28034
2.21	-0.87034	-5.66465	-0.71843	-6.46386	-0.6179	-7.09854
2.22	-0.91572	-5.47833	-0.75421	-6.28074	-0.64771	-6.918
2.23	-0.96347	-5.29384	-0.79174	-6.09912	-0.67893	-6.73878
2.24	-1.01371	-5.11126	-0.83112	-5.91906	-0.71161	-6.56092
2.25	-1.06657	-4.9307	-0.87243	-5.74064	-0.74582	-6.3845
2.26	-1.12217	-4.75225	-0.91576	-5.56393	-0.78164	-6.20957
2.27	-1.18065	-4.57603	-0.96119	-5.38901	-0.81912	-6.03619
2.28	-1.24216	-4.40213	-1.00884	-5.21596	-0.85834	-5.86444
2.29	-1.30683	-4.23065	-1.05879	-5.04486	-0.89938	-5.69436
2.3	-1.37483	-4.06172	-1.11115	-4.87579	-0.94231	-5.52604
2.31	-1.44631	-3.89543	-1.16602	-4.70884	-0.98721	-5.35953
2.32	-1.52144	-3.73189	-1.22353	-4.54408	-1.03416	-5.19491
2.33	-1.60038	-3.57122	-1.28377	-4.38161	-1.08325	-5.03224
2.34	-1.68331	-3.41351	-1.34687	-4.2215	-1.13456	-4.8716
2.35	-1.77042	-3.25887	-1.41294	-4.06384	-1.18818	-4.71305
2.36	-1.8619	-3.1074	-1.48212	-3.90872	-1.2442	-4.55666
2.37	-1.95793	-2.9592	-1.55453	-3.7562	-1.30272	-4.4025

2.38	-2.05871	-2.81437	-1.6303	-3.60639	-1.36382	-4.25064
2.39	-2.16446	-2.673	-1.70955	-3.45934	-1.4276	-4.10115
2.4	-2.27538	-2.53518	-1.79244	-3.31515	-1.49417	-3.95409
2.41	-2.39169	-2.40099	-1.87909	-3.17389	-1.56362	-3.80953
2.42	-2.51361	-2.2705	-1.96966	-3.03562	-1.63605	-3.66754
2.43	-2.64137	-2.14379	-2.06427	-2.90042	-1.71158	-3.52817
2.44	-2.77519	-2.02092	-2.16309	-2.76835	-1.79029	-3.39148
2.45	-2.91533	-1.90194	-2.26626	-2.63947	-1.8723	-3.25753
2.46	-3.06201	-1.78691	-2.37392	-2.51383	-1.95771	-3.12638
2.47	-3.21549	-1.67585	-2.48624	-2.39149	-2.04664	-2.99808
2.48	-3.37602	-1.5688	-2.60337	-2.27249	-2.13918	-2.87266
2.49	-3.54385	-1.46579	-2.72545	-2.15687	-2.23544	-2.75018
2.5	-3.71927	-1.36681	-2.85265	-2.04467	-2.33554	-2.63068
2.51	-3.90252	-1.27189	-2.98513	-1.93591	-2.43958	-2.51419
2.52	-4.09391	-1.181	-3.12303	-1.83061	-2.54766	-2.40074
2.53	-4.2937	-1.09414	-3.26653	-1.72879	-2.65989	-2.29035
2.54	-4.50219	-1.01127	-3.41578	-1.63046	-2.77639	-2.18306
2.55	-4.71969	-0.93237	-3.57094	-1.53562	-2.89724	-2.07887
2.56	-4.94651	-0.85739	-3.73217	-1.44426	-3.02256	-1.97779
2.57	-5.18298	-0.78628	-3.89964	-1.35637	-3.15244	-1.87983
2.58	-5.42944	-0.71899	-4.07349	-1.27193	-3.28699	-1.785
2.59	-5.68625	-0.65544	-4.25391	-1.19091	-3.42631	-1.69328
2.6	-5.95379	-0.59555	-4.44105	-1.1133	-3.57048	-1.60467
2.61	-6.23247	-0.53926	-4.63508	-1.03904	-3.71961	-1.51916
2.62	-6.52272	-0.48648	-4.83617	-0.96809	-3.87378	-1.43671
2.63	-6.82499	-0.4371	-5.04449	-0.90041	-4.03309	-1.35731
2.64	-7.1398	-0.39104	-5.26022	-0.83593	-4.19763	-1.28093
2.65	-7.46769	-0.3482	-5.48353	-0.77461	-4.36747	-1.20752
2.66	-7.80926	-0.30847	-5.71462	-0.71637	-4.54271	-1.13706
2.67	-8.16516	-0.27175	-5.95367	-0.66115	-4.72343	-1.0695
2.68	-8.53614	-0.23792	-6.20089	-0.60888	-4.90971	-1.00479
2.69	-8.92301	-0.20688	-6.45649	-0.55948	-5.10164	-0.94289
2.7	-9.32668	-0.17852	-6.7207	-0.51286	-5.29928	-0.88372
2.71	-9.7482	-0.15271	-6.99374	-0.46896	-5.50272	-0.82725
2.72	-10.1887	-0.12936	-7.27588	-0.42768	-5.71205	-0.7734
2.73	-10.6496	-0.10835	-7.56739	-0.38895	-5.92733	-0.72212
2.74	-11.1325	-0.08956	-7.86856	-0.35267	-6.14867	-0.67333
2.75	-11.639	-0.07289	-8.17971	-0.31877	-6.37613	-0.62698
2.76	-12.1713	-0.05824	-8.5012	-0.28714	-6.6098	-0.58299
2.77	-12.7319	-0.04548	-8.83341	-0.25771	-6.84979	-0.54129
2.78	-13.3237	-0.03453	-9.17677	-0.23038	-7.09617	-0.50181
2.79	-13.95	-0.02528	-9.53175	-0.20507	-7.34905	-0.46448
2.8	-14.6152	-0.01763	-9.89886	-0.1817	-7.60855	-0.42923
2.81	-15.3244	-0.01148	-10.2787	-0.16016	-7.87476	-0.39598
2.82	-16.0838	-0.00674	-10.6719	-0.14039	-8.14782	-0.36466
2.83	-16.9014	-0.00332	-11.0792	-0.1223	-8.42786	-0.33519
2.84	-17.7874	-0.00114	-11.5015	-0.1058	-8.71502	-0.30751
2.85	-18.755	-0.00011	-11.9397	-0.09082	-9.00947	-0.28154

2.86	-19.822	-0.00015	-12.3948	-0.07727	-9.31137	-0.25722
2.87	-21.013	-0.00118	-12.8681	-0.06509	-9.62091	-0.23447
2.88	-22.363	-0.00314	-13.3609	-0.05419	-9.93831	-0.21322
2.89	-23.9253	-0.00594	-13.875	-0.04451	-10.2638	-0.1934
2.9	-25.7856	-0.00953	-14.4121	-0.03597	-10.5976	-0.17496
2.91	-28.0955	-0.01383	-14.9745	-0.02852	-10.9401	-0.15782
2.92	-31.1651	-0.01879	-15.5647	-0.02207	-11.2914	-0.14192
2.93	-35.8072	-0.02435	-16.1859	-0.01658	-11.6521	-0.12721
2.94	-45.9699	-0.03046	-16.8416	-0.01198	-12.0224	-0.11361
2.95	-44.6208	-0.03705	-17.5363	-0.00821	-12.4028	-0.10108
2.96	-35.6462	-0.04409	-18.2755	-0.00522	-12.7938	-0.08956
2.97	-31.4137	-0.05153	-19.0658	-0.00296	-13.1958	-0.07899
2.98	-28.6424	-0.05932	-19.9157	-0.00137	-13.6096	-0.06931
2.99	-26.5933	-0.06741	-20.8361	-0.0004	-14.0357	-0.06049
3	-24.9765	-0.07578	-21.8411	-1.21E-05	-14.4749	-0.05247
3.01	-23.6477	-0.08439	-22.9501	-0.00016	-14.9281	-0.04521
3.02	-22.5248	-0.09319	-24.1898	-0.0008	-15.3962	-0.03865
3.03	-21.5564	-0.10217	-25.5988	-0.00189	-15.8803	-0.03276
3.04	-20.7082	-0.11128	-27.2365	-0.00339	-16.3816	-0.02749
3.05	-19.9563	-0.1205	-29.2005	-0.00528	-16.9016	-0.02281
3.06	-19.2832	-0.1298	-31.6685	-0.00751	-17.4418	-0.01868
3.07	-18.6758	-0.13917	-35.0214	-0.01005	-18.0042	-0.01505
3.08	-18.124	-0.14856	-40.3561	-0.01287	-18.591	-0.0119
3.09	-17.6199	-0.15797	-55.5372	-0.01594	-19.2047	-0.00919
3.1	-17.1571	-0.16738	-44.3736	-0.01924	-19.8483	-0.0069
3.11	-16.7304	-0.17675	-37.3605	-0.02274	-20.5256	-0.00499
3.12	-16.3357	-0.18609	-33.6166	-0.02641	-21.2408	-0.00342
3.13	-15.9692	-0.19537	-31.0715	-0.03023	-21.9994	-0.00219
3.14	-15.6281	-0.20457	-29.1551	-0.03419	-22.8078	-0.00125
3.15	-15.3097	-0.21369	-27.6269	-0.03826	-23.6745	-0.00059
3.16	-15.0119	-0.2227	-26.3625	-0.04241	-24.6099	-0.00019
3.17	-14.7328	-0.23161	-25.2891	-0.04664	-25.6279	-1.09E-05
3.18	-14.4708	-0.2404	-24.3604	-0.05093	-26.7471	-4.53E-05
3.19	-14.2244	-0.24905	-23.5454	-0.05526	-27.9933	-0.00027
3.2	-13.9923	-0.25757	-22.8217	-0.05962	-29.4035	-0.00067
3.21	-13.7734	-0.26594	-22.1733	-0.06399	-31.0343	-0.00122
3.22	-13.5667	-0.27415	-21.5879	-0.06837	-32.9774	-0.00191
3.23	-13.3713	-0.2822	-21.0559	-0.07273	-35.3983	-0.00273
3.24	-13.1865	-0.29009	-20.57	-0.07707	-38.6429	-0.00365
3.25	-13.0114	-0.2978	-20.1241	-0.08138	-43.6637	-0.00467
3.26	-12.8455	-0.30534	-19.7132	-0.08565	-55.995	-0.00577
3.27	-12.6882	-0.3127	-19.3333	-0.08987	-49.8151	-0.00695
3.28	-12.5389	-0.31987	-18.9811	-0.09404	-42.0558	-0.00818
3.29	-12.3971	-0.32686	-18.6536	-0.09814	-38.1292	-0.00946
3.3	-12.2623	-0.33365	-18.3485	-0.10217	-35.5115	-0.01078
3.31	-12.1343	-0.34025	-18.0636	-0.10613	-33.5628	-0.01213
3.32	-12.0126	-0.34667	-17.7971	-0.11	-32.0216	-0.01351
3.33	-11.8968	-0.35288	-17.5474	-0.11378	-30.755	-0.0149

3.34	-11.7866	-0.3589	-17.3132	-0.11748	-29.6862	-0.01629
3.35	-11.6818	-0.36472	-17.0932	-0.12108	-28.7667	-0.01769
3.36	-11.582	-0.37035	-16.8864	-0.12457	-27.9639	-0.01908
3.37	-11.4871	-0.37578	-16.6917	-0.12797	-27.255	-0.02046
3.38	-11.3967	-0.38101	-16.5084	-0.13126	-26.6233	-0.02182
3.39	-11.3107	-0.38604	-16.3356	-0.13445	-26.056	-0.02316
3.4	-11.2288	-0.39088	-16.1726	-0.13752	-25.5436	-0.02448
3.41	-11.1509	-0.39552	-16.0189	-0.14049	-25.0783	-0.02576
3.42	-11.0768	-0.39997	-15.8739	-0.14334	-24.6541	-0.02701
3.43	-11.0063	-0.40423	-15.7369	-0.14607	-24.2658	-0.02823
3.44	-10.9393	-0.4083	-15.6077	-0.14869	-23.9094	-0.0294
3.45	-10.8756	-0.41217	-15.4857	-0.1512	-23.5815	-0.03054
3.46	-10.8151	-0.41586	-15.3706	-0.15359	-23.2791	-0.03162
3.47	-10.7576	-0.41936	-15.2619	-0.15586	-22.9999	-0.03267
3.48	-10.7031	-0.42267	-15.1594	-0.15801	-22.7416	-0.03366
3.49	-10.6514	-0.4258	-15.0627	-0.16005	-22.5024	-0.0346
3.5	-10.6024	-0.42876	-14.9716	-0.16197	-22.2809	-0.03549
3.51	-10.556	-0.43153	-14.8858	-0.16378	-22.0755	-0.03633
3.52	-10.5121	-0.43413	-14.805	-0.16547	-21.8851	-0.03712
3.53	-10.4707	-0.43655	-14.7291	-0.16705	-21.7085	-0.03785
3.54	-10.4315	-0.4388	-14.6578	-0.16851	-21.5449	-0.03853
3.55	-10.3947	-0.44089	-14.5909	-0.16986	-21.3934	-0.03915
3.56	-10.36	-0.4428	-14.5283	-0.1711	-21.2532	-0.03972
3.57	-10.3274	-0.44456	-14.4697	-0.17223	-21.1237	-0.04023
3.58	-10.2969	-0.44615	-14.415	-0.17325	-21.0042	-0.04069
3.59	-10.2684	-0.44759	-14.3641	-0.17416	-20.8941	-0.04109
3.6	-10.2417	-0.44887	-14.3169	-0.17497	-20.7931	-0.04144
3.61	-10.2169	-0.44999	-14.2731	-0.17567	-20.7005	-0.04173
3.62	-10.1939	-0.45097	-14.2327	-0.17627	-20.6161	-0.04198
3.63	-10.1727	-0.4518	-14.1956	-0.17676	-20.5394	-0.04217
3.64	-10.1532	-0.45248	-14.1616	-0.17716	-20.4701	-0.0423
3.65	-10.1352	-0.45302	-14.1306	-0.17746	-20.4079	-0.04239
3.66	-10.1189	-0.45343	-14.1026	-0.17767	-20.3524	-0.04243
3.67	-10.1042	-0.45369	-14.0775	-0.17778	-20.3035	-0.04241
3.68	-10.091	-0.45383	-14.0551	-0.1778	-20.2609	-0.04235
3.69	-10.0792	-0.45383	-14.0355	-0.17773	-20.2244	-0.04225
3.7	-10.0689	-0.45371	-14.0185	-0.17757	-20.1937	-0.04209
3.71	-10.0599	-0.45346	-14.004	-0.17732	-20.1688	-0.0419
3.72	-10.0524	-0.45309	-13.992	-0.17699	-20.1493	-0.04166
3.73	-10.0461	-0.4526	-13.9824	-0.17658	-20.1353	-0.04137
3.74	-10.0412	-0.45199	-13.9752	-0.17609	-20.1264	-0.04105
3.75	-10.0375	-0.45127	-13.9703	-0.17552	-20.1227	-0.04069
3.76	-10.0351	-0.45044	-13.9677	-0.17488	-20.124	-0.04029
3.77	-10.0339	-0.4495	-13.9672	-0.17416	-20.1301	-0.03986
3.78	-10.0338	-0.44846	-13.9689	-0.17337	-20.1411	-0.03939
3.79	-10.035	-0.44731	-13.9727	-0.17251	-20.1567	-0.03888
3.8	-10.0372	-0.44606	-13.9786	-0.17158	-20.177	-0.03835
3.81	-10.0406	-0.44472	-13.9865	-0.17059	-20.2018	-0.03778

3.82	-10.045	-0.44328	-13.9964	-0.16954	-20.2312	-0.03719
3.83	-10.0506	-0.44175	-14.0083	-0.16842	-20.265	-0.03657
3.84	-10.0571	-0.44012	-14.022	-0.16725	-20.3032	-0.03592
3.85	-10.0647	-0.43842	-14.0377	-0.16602	-20.3458	-0.03525
3.86	-10.0733	-0.43662	-14.0552	-0.16473	-20.3927	-0.03456
3.87	-10.0829	-0.43475	-14.0745	-0.16339	-20.444	-0.03384
3.88	-10.0935	-0.43279	-14.0957	-0.162	-20.4996	-0.03311
3.89	-10.105	-0.43076	-14.1186	-0.16056	-20.5595	-0.03236
3.9	-10.1175	-0.42865	-14.1433	-0.15907	-20.6237	-0.03159
3.91	-10.1308	-0.42647	-14.1697	-0.15754	-20.6922	-0.0308
3.92	-10.1451	-0.42422	-14.1978	-0.15597	-20.765	-0.03001
3.93	-10.1603	-0.4219	-14.2276	-0.15435	-20.8422	-0.0292
3.94	-10.1764	-0.41952	-14.2591	-0.1527	-20.9238	-0.02838
3.95	-10.1933	-0.41708	-14.2923	-0.15101	-21.0097	-0.02755
3.96	-10.211	-0.41457	-14.327	-0.14928	-21.1001	-0.02671
3.97	-10.2297	-0.412	-14.3635	-0.14752	-21.195	-0.02587
3.98	-10.2491	-0.40938	-14.4015	-0.14573	-21.2945	-0.02502
3.99	-10.2694	-0.40671	-14.4412	-0.14391	-21.3986	-0.02416
4	-10.2904	-0.40398	-14.4824	-0.14206	-21.5073	-0.02331
4.01	-10.3123	-0.4012	-14.5252	-0.14018	-21.6209	-0.02246
4.02	-10.3349	-0.39837	-14.5696	-0.13828	-21.7393	-0.0216
4.03	-10.3584	-0.3955	-14.6156	-0.13635	-21.8626	-0.02075
4.04	-10.3826	-0.39258	-14.6631	-0.13441	-21.9911	-0.0199
4.05	-10.4075	-0.38962	-14.7122	-0.13244	-22.1247	-0.01905
4.06	-10.4332	-0.38662	-14.7629	-0.13046	-22.2636	-0.01821
4.07	-10.4596	-0.38358	-14.8151	-0.12846	-22.4081	-0.01738
4.08	-10.4868	-0.3805	-14.8688	-0.12644	-22.5582	-0.01655
4.09	-10.5147	-0.37739	-14.9241	-0.12441	-22.714	-0.01574
4.1	-10.5433	-0.37425	-14.9809	-0.12237	-22.8759	-0.01493
4.11	-10.5726	-0.37108	-15.0393	-0.12032	-23.044	-0.01414
4.12	-10.6027	-0.36787	-15.0992	-0.11826	-23.2185	-0.01336
4.13	-10.6334	-0.36464	-15.1607	-0.11619	-23.3997	-0.01259
4.14	-10.6648	-0.36138	-15.2237	-0.11411	-23.5878	-0.01183
4.15	-10.6969	-0.3581	-15.2883	-0.11203	-23.7831	-0.0111
4.16	-10.7297	-0.3548	-15.3544	-0.10995	-23.986	-0.01037
4.17	-10.7631	-0.35147	-15.4221	-0.10786	-24.1969	-0.00967
4.18	-10.7972	-0.34812	-15.4913	-0.10577	-24.416	-0.00898
4.19	-10.832	-0.34476	-15.5622	-0.10369	-24.6438	-0.00832
4.2	-10.8674	-0.34138	-15.6346	-0.1016	-24.8808	-0.00767
4.21	-10.9035	-0.33798	-15.7086	-0.09952	-25.1275	-0.00705
4.22	-10.9402	-0.33457	-15.7842	-0.09744	-25.3844	-0.00644
4.23	-10.9776	-0.33114	-15.8615	-0.09536	-25.6522	-0.00586
4.24	-11.0156	-0.32771	-15.9403	-0.09329	-25.9316	-0.0053
4.25	-11.0542	-0.32427	-16.0208	-0.09123	-26.2233	-0.00477
4.26	-11.0935	-0.32081	-16.103	-0.08918	-26.5283	-0.00426
4.27	-11.1334	-0.31735	-16.1868	-0.08713	-26.8474	-0.00378
4.28	-11.1739	-0.31388	-16.2723	-0.0851	-27.1819	-0.00332
4.29	-11.2151	-0.31041	-16.3596	-0.08307	-27.5328	-0.00289

4.3	-11.2568	-0.30694	-16.4485	-0.08106	-27.9018	-0.00249
4.31	-11.2992	-0.30346	-16.5392	-0.07906	-28.2903	-0.00212
4.32	-11.3422	-0.29998	-16.6316	-0.07708	-28.7002	-0.00177
4.33	-11.3858	-0.2965	-16.7259	-0.0751	-29.1338	-0.00146
4.34	-11.43	-0.29302	-16.8219	-0.07315	-29.5936	-0.00117
4.35	-11.4748	-0.28955	-16.9198	-0.07121	-30.0824	-0.00092
4.36	-11.5202	-0.28607	-17.0195	-0.06929	-30.6039	-0.00069
4.37	-11.5661	-0.2826	-17.1212	-0.06738	-31.1624	-0.0005
4.38	-11.6127	-0.27914	-17.2247	-0.06549	-31.763	-0.00033
4.39	-11.6599	-0.27568	-17.3302	-0.06363	-32.4121	-0.0002
4.4	-11.7077	-0.27223	-17.4377	-0.06178	-33.1176	-0.0001
4.41	-11.756	-0.26878	-17.5471	-0.05995	-33.8897	-3.72E-05
4.42	-11.805	-0.26535	-17.6587	-0.05815	-34.7417	-4.09E-06
4.43	-11.8545	-0.26192	-17.7723	-0.05636	-35.6912	-4.26E-06
4.44	-11.9046	-0.25851	-17.888	-0.0546	-36.7625	-3.80E-05
4.45	-11.9553	-0.2551	-18.0059	-0.05286	-37.9904	-0.00011
4.46	-12.0066	-0.25171	-18.1261	-0.05114	-39.4272	-0.00021
4.47	-12.0585	-0.24833	-18.2485	-0.04945	-41.1571	-0.00034
4.48	-12.1109	-0.24496	-18.3732	-0.04778	-43.3285	-0.00051
4.49	-12.1639	-0.24161	-18.5002	-0.04614	-46.2423	-0.00072
4.5	-12.2175	-0.23827	-18.6297	-0.04452	-50.6774	-0.00096
4.51	-12.2717	-0.23495	-18.7617	-0.04293	-60.2639	-0.00123
4.52	-12.3264	-0.23164	-18.8961	-0.04137	-60.0832	-0.00154
4.53	-12.3818	-0.22835	-19.0332	-0.03983	-50.5852	-0.00188
4.54	-12.4377	-0.22508	-19.1729	-0.03831	-46.1484	-0.00226
4.55	-12.4941	-0.22182	-19.3154	-0.03683	-43.2202	-0.00268
4.56	-12.5512	-0.21859	-19.4607	-0.03537	-41.0301	-0.00313
4.57	-12.6088	-0.21537	-19.6088	-0.03394	-39.2793	-0.00361
4.58	-12.667	-0.21218	-19.76	-0.03254	-37.8205	-0.00413
4.59	-12.7258	-0.209	-19.9141	-0.03117	-36.5699	-0.00469
4.6	-12.7851	-0.20584	-20.0715	-0.02982	-35.4754	-0.00528
4.61	-12.845	-0.20271	-20.232	-0.02851	-34.5023	-0.00591
4.62	-12.9055	-0.1996	-20.3959	-0.02722	-33.6264	-0.00657
4.63	-12.9666	-0.1965	-20.5633	-0.02596	-32.8299	-0.00726
4.64	-13.0282	-0.19344	-20.7343	-0.02474	-32.0998	-0.00799
4.65	-13.0904	-0.19039	-20.9089	-0.02354	-31.4259	-0.00876
4.66	-13.1532	-0.18737	-21.0874	-0.02237	-30.8001	-0.00956
4.67	-13.2165	-0.18437	-21.2698	-0.02123	-30.2162	-0.01039
4.68	-13.2805	-0.18139	-21.4564	-0.02012	-29.6689	-0.01126
4.69	-13.345	-0.17844	-21.6472	-0.01904	-29.1539	-0.01217
4.7	-13.4101	-0.17552	-21.8425	-0.018	-28.6678	-0.0131
4.71	-13.4758	-0.17262	-22.0425	-0.01698	-28.2075	-0.01407
4.72	-13.542	-0.16974	-22.2472	-0.01599	-27.7704	-0.01508
4.73	-13.6089	-0.16689	-22.457	-0.01503	-27.3545	-0.01612
4.74	-13.6763	-0.16407	-22.672	-0.01411	-26.9577	-0.01719
4.75	-13.7443	-0.16127	-22.8926	-0.01321	-26.5785	-0.01829
4.76	-13.8129	-0.1585	-23.1189	-0.01234	-26.2153	-0.01943
4.77	-13.882	-0.15575	-23.3512	-0.01151	-25.8671	-0.02059

4.78	-13.9518	-0.15303	-23.5899	-0.0107	-25.5326	-0.02179
4.79	-14.0221	-0.15034	-23.8352	-0.00993	-25.2108	-0.02303
4.8	-14.0931	-0.14767	-24.0876	-0.00918	-24.9009	-0.02429
4.81	-14.1646	-0.14504	-24.3474	-0.00847	-24.6021	-0.02558
4.82	-14.2367	-0.14243	-24.615	-0.00778	-24.3135	-0.02691
4.83	-14.3095	-0.13984	-24.8909	-0.00713	-24.0347	-0.02827
4.84	-14.3828	-0.13729	-25.1756	-0.0065	-23.765	-0.02965
4.85	-14.4567	-0.13476	-25.4697	-0.00591	-23.5038	-0.03107
4.86	-14.5312	-0.13226	-25.7737	-0.00534	-23.2508	-0.03251
4.87	-14.6064	-0.12979	-26.0884	-0.00481	-23.0053	-0.03399
4.88	-14.6821	-0.12735	-26.4145	-0.0043	-22.7671	-0.03549
4.89	-14.7584	-0.12493	-26.7529	-0.00383	-22.5357	-0.03702
4.9	-14.8354	-0.12255	-27.1044	-0.00338	-22.3109	-0.03858
4.91	-14.913	-0.12019	-27.4702	-0.00296	-22.0922	-0.04017
4.92	-14.9912	-0.11786	-27.8513	-0.00257	-21.8794	-0.04178
4.93	-15.07	-0.11555	-28.2493	-0.00221	-21.6722	-0.04342
4.94	-15.1494	-0.11328	-28.6655	-0.00187	-21.4703	-0.04509
4.95	-15.2295	-0.11103	-29.1019	-0.00157	-21.2736	-0.04678
4.96	-15.3102	-0.10882	-29.5603	-0.00129	-21.0818	-0.0485
4.97	-15.3915	-0.10663	-30.0432	-0.00104	-20.8946	-0.05024
4.98	-15.4734	-0.10447	-30.5533	-0.00082	-20.7119	-0.05201
4.99	-15.556	-0.10233	-31.094	-0.00062	-20.5336	-0.0538
5	-15.6393	-0.10023	-31.6691	-0.00046	-20.3594	-0.05561
5.01	-15.7231	-0.09815	-32.2833	-0.00031	-20.1891	-0.05745
5.02	-15.8077	-0.09611	-32.9426	-0.0002	-20.0227	-0.05931
5.03	-15.8929	-0.09409	-33.654	-0.00011	-19.86	-0.06119
5.04	-15.9787	-0.09209	-34.4266	-4.84E-05	-19.7008	-0.0631
5.05	-16.0652	-0.09013	-35.2721	-1.15E-05	-19.545	-0.06503
5.06	-16.1524	-0.08819	-36.206	-2.48E-08	-19.3926	-0.06697
5.07	-16.2402	-0.08628	-37.249	-1.36E-05	-19.2433	-0.06894
5.08	-16.3287	-0.0844	-38.4307	-5.21E-05	-19.0971	-0.07093
5.09	-16.4179	-0.08255	-39.794	-0.00012	-18.954	-0.07294
5.1	-16.5078	-0.08072	-41.406	-0.0002	-18.8136	-0.07496
5.11	-16.5983	-0.07893	-43.3792	-0.00031	-18.6761	-0.07701
5.12	-16.6896	-0.07715	-45.9264	-0.00045	-18.5413	-0.07907
5.13	-16.7815	-0.07541	-49.5298	-0.00061	-18.4091	-0.08115
5.14	-16.8742	-0.07369	-55.7663	-0.00079	-18.2795	-0.08325
5.15	-16.9675	-0.072	-82.4361	-0.00099	-18.1523	-0.08536
5.16	-17.0616	-0.07034	-55.0273	-0.00122	-18.0275	-0.08749
5.17	-17.1564	-0.0687	-49.2104	-0.00146	-17.9051	-0.08964
5.18	-17.2519	-0.06709	-45.7689	-0.00173	-17.7849	-0.0918
5.19	-17.3481	-0.0655	-43.3193	-0.00203	-17.6669	-0.09397
5.2	-17.4451	-0.06394	-41.4178	-0.00234	-17.5511	-0.09616
5.21	-17.5428	-0.06241	-39.8648	-0.00267	-17.4373	-0.09836
5.22	-17.6412	-0.0609	-38.553	-0.00302	-17.3255	-0.10058
5.23	-17.7404	-0.05942	-37.4183	-0.0034	-17.2158	-0.10281
5.24	-17.8404	-0.05796	-36.4191	-0.00379	-17.1079	-0.10505
5.25	-17.9411	-0.05653	-35.5269	-0.0042	-17.002	-0.1073

5.26	-18.0426	-0.05512	-34.7215	-0.00463	-16.8978	-0.10957
5.27	-18.1449	-0.05374	-33.9877	-0.00508	-16.7954	-0.11184
5.28	-18.2479	-0.05238	-33.3143	-0.00555	-16.6948	-0.11412
5.29	-18.3518	-0.05105	-32.6923	-0.00603	-16.5959	-0.11642
5.3	-18.4564	-0.04974	-32.1146	-0.00654	-16.4986	-0.11872
5.31	-18.5619	-0.04846	-31.5756	-0.00705	-16.403	-0.12103
5.32	-18.6681	-0.04719	-31.0706	-0.00759	-16.3089	-0.12336
5.33	-18.7752	-0.04596	-30.5957	-0.00814	-16.2163	-0.12568
5.34	-18.8832	-0.04474	-30.1478	-0.00871	-16.1253	-0.12802
5.35	-18.9919	-0.04355	-29.724	-0.0093	-16.0358	-0.13036
5.36	-19.1015	-0.04238	-29.3221	-0.0099	-15.9477	-0.13271
5.37	-19.212	-0.04123	-28.9401	-0.01051	-15.861	-0.13506
5.38	-19.3233	-0.04011	-28.5761	-0.01114	-15.7757	-0.13742
5.39	-19.4355	-0.039	-28.2287	-0.01179	-15.6918	-0.13979
5.4	-19.5486	-0.03792	-27.8965	-0.01244	-15.6091	-0.14216
5.41	-19.6626	-0.03686	-27.5783	-0.01312	-15.5278	-0.14453
5.42	-19.7775	-0.03583	-27.2732	-0.0138	-15.4478	-0.14691
5.43	-19.8933	-0.03481	-26.9802	-0.0145	-15.369	-0.14929
5.44	-20.01	-0.03381	-26.6984	-0.01521	-15.2914	-0.15167
5.45	-20.1276	-0.03284	-26.4271	-0.01593	-15.215	-0.15405
5.46	-20.2462	-0.03189	-26.1656	-0.01666	-15.1398	-0.15644
5.47	-20.3658	-0.03095	-25.9133	-0.01741	-15.0658	-0.15883
5.48	-20.4863	-0.03004	-25.6697	-0.01816	-14.9928	-0.16121
5.49	-20.6078	-0.02914	-25.4342	-0.01893	-14.921	-0.1636
5.5	-20.7302	-0.02827	-25.2064	-0.0197	-14.8503	-0.16599
5.51	-20.8537	-0.02741	-24.9858	-0.02049	-14.7807	-0.16838
5.52	-20.9782	-0.02658	-24.7721	-0.02129	-14.7121	-0.17076
5.53	-21.1037	-0.02576	-24.565	-0.02209	-14.6445	-0.17315
5.54	-21.2302	-0.02496	-24.364	-0.02291	-14.5779	-0.17553
5.55	-21.3578	-0.02418	-24.1689	-0.02373	-14.5124	-0.17791
5.56	-21.4865	-0.02341	-23.9794	-0.02456	-14.4478	-0.18029
5.57	-21.6162	-0.02267	-23.7953	-0.0254	-14.3842	-0.18266
5.58	-21.7471	-0.02194	-23.6162	-0.02624	-14.3215	-0.18503
5.59	-21.879	-0.02123	-23.442	-0.02709	-14.2598	-0.1874
5.6	-22.0121	-0.02054	-23.2725	-0.02795	-14.1989	-0.18976
5.61	-22.1463	-0.01986	-23.1074	-0.02882	-14.139	-0.19212
5.62	-22.2816	-0.0192	-22.9466	-0.02969	-14.08	-0.19447
5.63	-22.4182	-0.01856	-22.7899	-0.03057	-14.0218	-0.19682
5.64	-22.5559	-0.01793	-22.6372	-0.03145	-13.9645	-0.19916
5.65	-22.6948	-0.01732	-22.4882	-0.03234	-13.908	-0.20149
5.66	-22.8349	-0.01672	-22.3429	-0.03323	-13.8524	-0.20382
5.67	-22.9763	-0.01614	-22.2011	-0.03413	-13.7975	-0.20614
5.68	-23.1189	-0.01557	-22.0627	-0.03503	-13.7435	-0.20845
5.69	-23.2628	-0.01502	-21.9275	-0.03593	-13.6903	-0.21075
5.7	-23.408	-0.01448	-21.7955	-0.03684	-13.6378	-0.21305
5.71	-23.5545	-0.01396	-21.6665	-0.03775	-13.5862	-0.21534
5.72	-23.7023	-0.01345	-21.5405	-0.03866	-13.5352	-0.21761
5.73	-23.8515	-0.01296	-21.4173	-0.03958	-13.4851	-0.21988

5.74	-24.002	-0.01248	-21.2969	-0.0405	-13.4356	-0.22214
5.75	-24.154	-0.01201	-21.1791	-0.04142	-13.3869	-0.22439
5.76	-24.3074	-0.01156	-21.0639	-0.04234	-13.3389	-0.22662
5.77	-24.4622	-0.01111	-20.9512	-0.04326	-13.2916	-0.22885
5.78	-24.6185	-0.01069	-20.8409	-0.04419	-13.245	-0.23106
5.79	-24.7763	-0.01027	-20.733	-0.04511	-13.1991	-0.23327
5.8	-24.9355	-0.00986	-20.6274	-0.04603	-13.1539	-0.23546
5.81	-25.0964	-0.00947	-20.524	-0.04696	-13.1093	-0.23764
5.82	-25.2588	-0.00909	-20.4228	-0.04788	-13.0654	-0.2398
5.83	-25.4228	-0.00872	-20.3237	-0.0488	-13.0222	-0.24196
5.84	-25.5884	-0.00837	-20.2266	-0.04973	-12.9796	-0.2441
5.85	-25.7556	-0.00802	-20.1315	-0.05065	-12.9376	-0.24622
5.86	-25.9246	-0.00768	-20.0384	-0.05157	-12.8963	-0.24833
5.87	-26.0952	-0.00736	-19.9471	-0.05248	-12.8555	-0.25043
5.88	-26.2676	-0.00704	-19.8577	-0.0534	-12.8154	-0.25251
5.89	-26.4418	-0.00674	-19.7701	-0.05431	-12.7759	-0.25458
5.9	-26.6178	-0.00644	-19.6843	-0.05522	-12.737	-0.25663
5.91	-26.7956	-0.00616	-19.6001	-0.05613	-12.6987	-0.25867
5.92	-26.9753	-0.00588	-19.5177	-0.05704	-12.6609	-0.26069
5.93	-27.1569	-0.00562	-19.4368	-0.05794	-12.6237	-0.2627
5.94	-27.3405	-0.00536	-19.3576	-0.05883	-12.5871	-0.26469
5.95	-27.526	-0.00511	-19.2799	-0.05973	-12.5511	-0.26666
5.96	-27.7136	-0.00488	-19.2038	-0.06062	-12.5156	-0.26861
5.97	-27.9033	-0.00464	-19.1291	-0.0615	-12.4806	-0.27055
5.98	-28.095	-0.00442	-19.056	-0.06238	-12.4462	-0.27247
5.99	-28.2889	-0.00421	-18.9842	-0.06326	-12.4124	-0.27438
6	-28.485	-0.004	-18.9139	-0.06413	-12.3791	-0.27626
6.01	-28.6834	-0.0038	-18.8449	-0.06499	-12.3462	-0.27813
6.02	-28.884	-0.00361	-18.7772	-0.06585	-12.314	-0.27997
6.03	-29.087	-0.00343	-18.7109	-0.06671	-12.2822	-0.2818
6.04	-29.2924	-0.00325	-18.6459	-0.06755	-12.2509	-0.28361
6.05	-29.5002	-0.00308	-18.5822	-0.06839	-12.2202	-0.2854
6.06	-29.7106	-0.00292	-18.5197	-0.06923	-12.1899	-0.28717
6.07	-29.9235	-0.00276	-18.4584	-0.07005	-12.1601	-0.28893
6.08	-30.139	-0.00261	-18.3983	-0.07088	-12.1309	-0.29066
6.09	-30.3571	-0.00247	-18.3394	-0.07169	-12.1021	-0.29237
6.1	-30.5781	-0.00233	-18.2817	-0.07249	-12.0737	-0.29406
6.11	-30.8018	-0.0022	-18.2251	-0.07329	-12.0459	-0.29573
6.12	-31.0283	-0.00207	-18.1696	-0.07408	-12.0185	-0.29738
6.13	-31.2579	-0.00195	-18.1152	-0.07487	-11.9916	-0.299
6.14	-31.4904	-0.00184	-18.0619	-0.07564	-11.9652	-0.30061
6.15	-31.726	-0.00173	-18.0096	-0.0764	-11.9392	-0.3022
6.16	-31.9648	-0.00163	-17.9585	-0.07716	-11.9136	-0.30376
6.17	-32.2068	-0.00153	-17.9083	-0.07791	-11.8886	-0.3053
6.18	-32.4521	-0.00143	-17.8592	-0.07865	-11.8639	-0.30682
6.19	-32.7008	-0.00134	-17.811	-0.07938	-11.8397	-0.30831
6.2	-32.953	-0.00125	-17.7639	-0.0801	-11.8159	-0.30979
6.21	-33.2088	-0.00117	-17.7177	-0.08081	-11.7926	-0.31124

6.22	-33.4683	-0.00109	-17.6724	-0.08151	-11.7697	-0.31267
6.23	-33.7316	-0.00102	-17.6281	-0.0822	-11.7472	-0.31407
6.24	-33.9987	-0.00095	-17.5848	-0.08289	-11.7252	-0.31545
6.25	-34.2699	-0.00088	-17.5423	-0.08356	-11.7036	-0.31681
6.26	-34.5451	-0.00082	-17.5008	-0.08422	-11.6823	-0.31815
6.27	-34.8246	-0.00076	-17.4602	-0.08487	-11.6615	-0.31946
6.28	-35.1083	-0.00071	-17.4204	-0.08551	-11.6412	-0.32075
6.29	-35.3966	-0.00066	-17.3815	-0.08614	-11.6212	-0.32201
6.3	-35.6894	-0.00061	-17.3435	-0.08675	-11.6016	-0.32325
6.31	-35.9869	-0.00056	-17.3063	-0.08736	-11.5824	-0.32446
6.32	-36.2893	-0.00052	-17.27	-0.08796	-11.5636	-0.32565
6.33	-36.5967	-0.00047	-17.2344	-0.08854	-11.5453	-0.32682
6.34	-36.9093	-0.00044	-17.1997	-0.08911	-11.5273	-0.32796
6.35	-37.2272	-0.0004	-17.1659	-0.08967	-11.5097	-0.32907
6.36	-37.5505	-0.00037	-17.1328	-0.09022	-11.4925	-0.33016
6.37	-37.8795	-0.00034	-17.1005	-0.09076	-11.4756	-0.33123
6.38	-38.2143	-0.00031	-17.069	-0.09129	-11.4592	-0.33227
6.39	-38.5551	-0.00028	-17.0382	-0.0918	-11.4431	-0.33328
6.4	-38.9021	-0.00025	-17.0083	-0.0923	-11.4274	-0.33427
6.41	-39.2554	-0.00023	-16.9791	-0.09279	-11.4121	-0.33523
6.42	-39.6154	-0.00021	-16.9506	-0.09326	-11.3972	-0.33617
6.43	-39.9821	-0.00019	-16.9229	-0.09373	-11.3826	-0.33708
6.44	-40.3558	-0.00017	-16.8959	-0.09418	-11.3684	-0.33796
6.45	-40.7368	-0.00015	-16.8697	-0.09461	-11.3546	-0.33882
6.46	-41.1253	-0.00014	-16.8442	-0.09504	-11.3412	-0.33965
6.47	-41.5214	-0.00012	-16.8194	-0.09545	-11.3281	-0.34046
6.48	-41.9255	-0.00011	-16.7953	-0.09585	-11.3153	-0.34124
6.49	-42.3378	-9.82E-05	-16.7719	-0.09623	-11.303	-0.34199
6.5	-42.7585	-8.73E-05	-16.7493	-0.09661	-11.2909	-0.34272
6.51	-43.1879	-7.75E-05	-16.7273	-0.09696	-11.2793	-0.34342
6.52	-43.6262	-6.86E-05	-16.706	-0.09731	-11.268	-0.34409
6.53	-44.0738	-6.06E-05	-16.6854	-0.09764	-11.257	-0.34474
6.54	-44.5308	-5.34E-05	-16.6655	-0.09796	-11.2464	-0.34536
6.55	-44.9975	-4.69E-05	-16.6463	-0.09826	-11.2362	-0.34595
6.56	-45.4741	-4.12E-05	-16.6277	-0.09855	-11.2263	-0.34651
6.57	-45.9609	-3.60E-05	-16.6098	-0.09883	-11.2168	-0.34705
6.58	-46.458	-3.15E-05	-16.5926	-0.0991	-11.2076	-0.34756
6.59	-46.9655	-2.74E-05	-16.576	-0.09934	-11.1987	-0.34805
6.6	-47.4836	-2.39E-05	-16.5601	-0.09958	-11.1902	-0.3485
6.61	-48.0124	-2.08E-05	-16.5448	-0.0998	-11.182	-0.34893
6.62	-48.5518	-1.81E-05	-16.5302	-0.10001	-11.1742	-0.34933
6.63	-49.1017	-1.57E-05	-16.5163	-0.1002	-11.1667	-0.3497
6.64	-49.6618	-1.37E-05	-16.5029	-0.10038	-11.1596	-0.35005
6.65	-50.2318	-1.19E-05	-16.4902	-0.10055	-11.1528	-0.35037
6.66	-50.8111	-1.04E-05	-16.4782	-0.1007	-11.1463	-0.35066
6.67	-51.3989	-9.18E-06	-16.4668	-0.10083	-11.1402	-0.35092
6.68	-51.9941	-8.12E-06	-16.456	-0.10095	-11.1344	-0.35116
6.69	-52.5954	-7.24E-06	-16.4458	-0.10106	-11.1289	-0.35137

6.7	-53.2008	-6.51E-06	-16.4363	-0.10116	-11.1238	-0.35155
6.71	-53.8082	-5.93E-06	-16.4274	-0.10123	-11.119	-0.3517
6.72	-54.4147	-5.47E-06	-16.4191	-0.1013	-11.1145	-0.35183
6.73	-55.0167	-5.13E-06	-16.4115	-0.10135	-11.1104	-0.35192
6.74	-55.61	-4.89E-06	-16.4044	-0.10139	-11.1066	-0.35199
6.75	-56.1895	-4.75E-06	-16.398	-0.10141	-11.1031	-0.35203
6.76	-56.7495	-4.71E-06	-16.3922	-0.10141	-11.1	-0.35205
6.77	-57.2831	-4.75E-06	-16.387	-0.10141	-11.0972	-0.35203
6.78	-57.7827	-4.89E-06	-16.3824	-0.10139	-11.0947	-0.35199
6.79	-58.2403	-5.13E-06	-16.3784	-0.10135	-11.0926	-0.35192
6.8	-58.6473	-5.47E-06	-16.3751	-0.1013	-11.0908	-0.35183
6.81	-58.9951	-5.93E-06	-16.3723	-0.10123	-11.0893	-0.3517
6.82	-59.2755	-6.51E-06	-16.3702	-0.10116	-11.0881	-0.35155
6.83	-59.4815	-7.24E-06	-16.3687	-0.10106	-11.0873	-0.35137
6.84	-59.6075	-8.12E-06	-16.3678	-0.10095	-11.0868	-0.35116
6.85	-59.65	-9.18E-06	-16.3675	-0.10083	-11.0867	-0.35092
6.86	-59.6075	-1.04E-05	-16.3678	-0.1007	-11.0868	-0.35066
6.87	-59.4815	-1.19E-05	-16.3687	-0.10055	-11.0873	-0.35037
6.88	-59.2755	-1.37E-05	-16.3702	-0.10038	-11.0881	-0.35005
6.89	-58.9951	-1.57E-05	-16.3723	-0.1002	-11.0893	-0.3497
6.9	-58.6473	-1.81E-05	-16.3751	-0.10001	-11.0908	-0.34933
6.91	-58.2403	-2.08E-05	-16.3784	-0.0998	-11.0926	-0.34893
6.92	-57.7827	-2.39E-05	-16.3824	-0.09958	-11.0947	-0.3485
6.93	-57.2831	-2.74E-05	-16.387	-0.09934	-11.0972	-0.34805
6.94	-56.7495	-3.15E-05	-16.3922	-0.0991	-11.1	-0.34756
6.95	-56.1895	-3.60E-05	-16.398	-0.09883	-11.1031	-0.34705
6.96	-55.61	-4.12E-05	-16.4044	-0.09855	-11.1066	-0.34651
6.97	-55.0167	-4.69E-05	-16.4115	-0.09826	-11.1104	-0.34595
6.98	-54.4147	-5.34E-05	-16.4191	-0.09796	-11.1145	-0.34536
6.99	-53.8082	-6.06E-05	-16.4274	-0.09764	-11.119	-0.34474
7	-53.2008	-6.86E-05	-16.4363	-0.09731	-11.1238	-0.34409
7.01	-52.5954	-7.75E-05	-16.4458	-0.09696	-11.1289	-0.34342
7.02	-51.9941	-8.73E-05	-16.456	-0.09661	-11.1344	-0.34272
7.03	-51.3989	-9.82E-05	-16.4668	-0.09623	-11.1402	-0.34199
7.04	-50.8111	-0.00011	-16.4782	-0.09585	-11.1463	-0.34124
7.05	-50.2318	-0.00012	-16.4902	-0.09545	-11.1528	-0.34046
7.06	-49.6618	-0.00014	-16.5029	-0.09504	-11.1596	-0.33965
7.07	-49.1017	-0.00015	-16.5163	-0.09461	-11.1667	-0.33882
7.08	-48.5518	-0.00017	-16.5302	-0.09418	-11.1742	-0.33796
7.09	-48.0124	-0.00019	-16.5448	-0.09373	-11.182	-0.33708
7.1	-47.4836	-0.00021	-16.5601	-0.09326	-11.1902	-0.33617
7.11	-46.9655	-0.00023	-16.576	-0.09279	-11.1987	-0.33523
7.12	-46.458	-0.00025	-16.5926	-0.0923	-11.2076	-0.33427
7.13	-45.9609	-0.00028	-16.6098	-0.0918	-11.2168	-0.33328
7.14	-45.4741	-0.00031	-16.6277	-0.09129	-11.2263	-0.33227
7.15	-44.9975	-0.00034	-16.6463	-0.09076	-11.2362	-0.33123
7.16	-44.5308	-0.00037	-16.6655	-0.09022	-11.2464	-0.33016
7.17	-44.0738	-0.0004	-16.6854	-0.08967	-11.257	-0.32907

7.18	-43.6262	-0.00044	-16.706	-0.08911	-11.268	-0.32796
7.19	-43.1879	-0.00047	-16.7273	-0.08854	-11.2793	-0.32682
7.2	-42.7585	-0.00052	-16.7493	-0.08796	-11.2909	-0.32565
7.21	-42.3378	-0.00056	-16.7719	-0.08736	-11.303	-0.32446
7.22	-41.9255	-0.00061	-16.7953	-0.08675	-11.3153	-0.32325
7.23	-41.5214	-0.00066	-16.8194	-0.08614	-11.3281	-0.32201
7.24	-41.1253	-0.00071	-16.8442	-0.08551	-11.3412	-0.32075
7.25	-40.7368	-0.00076	-16.8697	-0.08487	-11.3546	-0.31946
7.26	-40.3558	-0.00082	-16.8959	-0.08422	-11.3684	-0.31815
7.27	-39.9821	-0.00088	-16.9229	-0.08356	-11.3826	-0.31681
7.28	-39.6154	-0.00095	-16.9506	-0.08289	-11.3972	-0.31545
7.29	-39.2554	-0.00102	-16.9791	-0.0822	-11.4121	-0.31407
7.3	-38.9021	-0.00109	-17.0083	-0.08151	-11.4274	-0.31267
7.31	-38.5551	-0.00117	-17.0382	-0.08081	-11.4431	-0.31124
7.32	-38.2143	-0.00125	-17.069	-0.0801	-11.4592	-0.30979
7.33	-37.8795	-0.00134	-17.1005	-0.07938	-11.4756	-0.30831
7.34	-37.5505	-0.00143	-17.1328	-0.07865	-11.4925	-0.30682
7.35	-37.2272	-0.00153	-17.1659	-0.07791	-11.5097	-0.3053
7.36	-36.9093	-0.00163	-17.1997	-0.07716	-11.5273	-0.30376
7.37	-36.5967	-0.00173	-17.2344	-0.0764	-11.5453	-0.3022
7.38	-36.2893	-0.00184	-17.27	-0.07564	-11.5636	-0.30061
7.39	-35.9869	-0.00195	-17.3063	-0.07487	-11.5824	-0.299
7.4	-35.6894	-0.00207	-17.3435	-0.07408	-11.6016	-0.29738
7.41	-35.3966	-0.0022	-17.3815	-0.07329	-11.6212	-0.29573
7.42	-35.1083	-0.00233	-17.4204	-0.07249	-11.6412	-0.29406
7.43	-34.8246	-0.00247	-17.4602	-0.07169	-11.6615	-0.29237
7.44	-34.5451	-0.00261	-17.5008	-0.07088	-11.6823	-0.29066
7.45	-34.2699	-0.00276	-17.5423	-0.07005	-11.7036	-0.28893
7.46	-33.9987	-0.00292	-17.5848	-0.06923	-11.7252	-0.28717
7.47	-33.7316	-0.00308	-17.6281	-0.06839	-11.7472	-0.2854
7.48	-33.4683	-0.00325	-17.6724	-0.06755	-11.7697	-0.28361
7.49	-33.2088	-0.00343	-17.7177	-0.06671	-11.7926	-0.2818
7.5	-32.953	-0.00361	-17.7639	-0.06585	-11.8159	-0.27997
7.51	-32.7008	-0.0038	-17.811	-0.06499	-11.8397	-0.27813
7.52	-32.4521	-0.004	-17.8592	-0.06413	-11.8639	-0.27626
7.53	-32.2068	-0.00421	-17.9083	-0.06326	-11.8886	-0.27438
7.54	-31.9648	-0.00442	-17.9585	-0.06238	-11.9136	-0.27247
7.55	-31.726	-0.00464	-18.0096	-0.0615	-11.9392	-0.27055
7.56	-31.4904	-0.00488	-18.0619	-0.06062	-11.9652	-0.26861
7.57	-31.2579	-0.00511	-18.1152	-0.05973	-11.9916	-0.26666
7.58	-31.0283	-0.00536	-18.1696	-0.05883	-12.0185	-0.26469
7.59	-30.8018	-0.00562	-18.2251	-0.05794	-12.0459	-0.2627
7.6	-30.5781	-0.00588	-18.2817	-0.05704	-12.0737	-0.26069
7.61	-30.3571	-0.00616	-18.3394	-0.05613	-12.1021	-0.25867
7.62	-30.139	-0.00644	-18.3983	-0.05522	-12.1309	-0.25663
7.63	-29.9235	-0.00674	-18.4584	-0.05431	-12.1601	-0.25458
7.64	-29.7106	-0.00704	-18.5197	-0.0534	-12.1899	-0.25251
7.65	-29.5002	-0.00736	-18.5822	-0.05248	-12.2202	-0.25043

7.66	-29.2924	-0.00768	-18.6459	-0.05157	-12.2509	-0.24833
7.67	-29.087	-0.00802	-18.7109	-0.05065	-12.2822	-0.24622
7.68	-28.884	-0.00837	-18.7772	-0.04973	-12.314	-0.2441
7.69	-28.6834	-0.00872	-18.8449	-0.0488	-12.3462	-0.24196
7.7	-28.485	-0.00909	-18.9139	-0.04788	-12.3791	-0.2398
7.71	-28.2889	-0.00947	-18.9842	-0.04696	-12.4124	-0.23764
7.72	-28.095	-0.00986	-19.056	-0.04603	-12.4462	-0.23546
7.73	-27.9033	-0.01027	-19.1291	-0.04511	-12.4806	-0.23327
7.74	-27.7136	-0.01069	-19.2038	-0.04419	-12.5156	-0.23106
7.75	-27.526	-0.01111	-19.2799	-0.04326	-12.5511	-0.22885
7.76	-27.3405	-0.01156	-19.3576	-0.04234	-12.5871	-0.22662
7.77	-27.1569	-0.01201	-19.4368	-0.04142	-12.6237	-0.22439
7.78	-26.9753	-0.01248	-19.5177	-0.0405	-12.6609	-0.22214
7.79	-26.7956	-0.01296	-19.6001	-0.03958	-12.6987	-0.21988
7.8	-26.6178	-0.01345	-19.6843	-0.03866	-12.737	-0.21761
7.81	-26.4418	-0.01396	-19.7701	-0.03775	-12.7759	-0.21534
7.82	-26.2676	-0.01448	-19.8577	-0.03684	-12.8154	-0.21305
7.83	-26.0952	-0.01502	-19.9471	-0.03593	-12.8555	-0.21075
7.84	-25.9246	-0.01557	-20.0384	-0.03503	-12.8963	-0.20845
7.85	-25.7556	-0.01614	-20.1315	-0.03413	-12.9376	-0.20614
7.86	-25.5884	-0.01672	-20.2266	-0.03323	-12.9796	-0.20382
7.87	-25.4228	-0.01732	-20.3237	-0.03234	-13.0222	-0.20149
7.88	-25.2588	-0.01793	-20.4228	-0.03145	-13.0654	-0.19916
7.89	-25.0964	-0.01856	-20.524	-0.03057	-13.1093	-0.19682
7.9	-24.9355	-0.0192	-20.6274	-0.02969	-13.1539	-0.19447
7.91	-24.7763	-0.01986	-20.733	-0.02882	-13.1991	-0.19212
7.92	-24.6185	-0.02054	-20.8409	-0.02795	-13.245	-0.18976
7.93	-24.4622	-0.02123	-20.9512	-0.02709	-13.2916	-0.1874
7.94	-24.3074	-0.02194	-21.0639	-0.02624	-13.3389	-0.18503
7.95	-24.154	-0.02267	-21.1791	-0.0254	-13.3869	-0.18266
7.96	-24.002	-0.02341	-21.2969	-0.02456	-13.4356	-0.18029
7.97	-23.8515	-0.02418	-21.4173	-0.02373	-13.4851	-0.17791
7.98	-23.7023	-0.02496	-21.5405	-0.02291	-13.5352	-0.17553
7.99	-23.5545	-0.02576	-21.6665	-0.02209	-13.5862	-0.17315
8	-23.408	-0.02658	-21.7955	-0.02129	-13.6378	-0.17076
8.01	-23.2628	-0.02741	-21.9275	-0.02049	-13.6903	-0.16838
8.02	-23.1189	-0.02827	-22.0627	-0.0197	-13.7435	-0.16599
8.03	-22.9763	-0.02914	-22.2011	-0.01893	-13.7975	-0.1636
8.04	-22.8349	-0.03004	-22.3429	-0.01816	-13.8524	-0.16121
8.05	-22.6948	-0.03095	-22.4882	-0.01741	-13.908	-0.15883
8.06	-22.5559	-0.03189	-22.6372	-0.01666	-13.9645	-0.15644
8.07	-22.4182	-0.03284	-22.7899	-0.01593	-14.0218	-0.15405
8.08	-22.2816	-0.03381	-22.9466	-0.01521	-14.08	-0.15167
8.09	-22.1463	-0.03481	-23.1074	-0.0145	-14.139	-0.14929
8.1	-22.0121	-0.03583	-23.2725	-0.0138	-14.1989	-0.14691
8.11	-21.879	-0.03686	-23.442	-0.01312	-14.2598	-0.14453
8.12	-21.7471	-0.03792	-23.6162	-0.01244	-14.3215	-0.14216
8.13	-21.6162	-0.039	-23.7953	-0.01179	-14.3842	-0.13979

8.14	-21.4865	-0.04011	-23.9794	-0.01114	-14.4478	-0.13742
8.15	-21.3578	-0.04123	-24.1689	-0.01051	-14.5124	-0.13506
8.16	-21.2302	-0.04238	-24.364	-0.0099	-14.5779	-0.13271
8.17	-21.1037	-0.04355	-24.565	-0.0093	-14.6445	-0.13036
8.18	-20.9782	-0.04474	-24.7721	-0.00871	-14.7121	-0.12802
8.19	-20.8537	-0.04596	-24.9858	-0.00814	-14.7807	-0.12568
8.2	-20.7302	-0.04719	-25.2064	-0.00759	-14.8503	-0.12336
8.21	-20.6078	-0.04846	-25.4342	-0.00705	-14.921	-0.12103
8.22	-20.4863	-0.04974	-25.6697	-0.00654	-14.9928	-0.11872
8.23	-20.3658	-0.05105	-25.9133	-0.00603	-15.0658	-0.11642
8.24	-20.2462	-0.05238	-26.1656	-0.00555	-15.1398	-0.11412
8.25	-20.1276	-0.05374	-26.4271	-0.00508	-15.215	-0.11184
8.26	-20.01	-0.05512	-26.6984	-0.00463	-15.2914	-0.10957
8.27	-19.8933	-0.05653	-26.9802	-0.0042	-15.369	-0.1073
8.28	-19.7775	-0.05796	-27.2732	-0.00379	-15.4478	-0.10505
8.29	-19.6626	-0.05942	-27.5783	-0.0034	-15.5278	-0.10281
8.3	-19.5486	-0.0609	-27.8965	-0.00302	-15.6091	-0.10058
8.31	-19.4355	-0.06241	-28.2287	-0.00267	-15.6918	-0.09836
8.32	-19.3233	-0.06394	-28.5761	-0.00234	-15.7757	-0.09616
8.33	-19.212	-0.0655	-28.9401	-0.00203	-15.861	-0.09397
8.34	-19.1015	-0.06709	-29.3221	-0.00173	-15.9477	-0.0918
8.35	-18.9919	-0.0687	-29.724	-0.00146	-16.0358	-0.08964
8.36	-18.8832	-0.07034	-30.1478	-0.00122	-16.1253	-0.08749
8.37	-18.7752	-0.072	-30.5957	-0.00099	-16.2163	-0.08536
8.38	-18.6681	-0.07369	-31.0706	-0.00079	-16.3089	-0.08325
8.39	-18.5619	-0.07541	-31.5756	-0.00061	-16.403	-0.08115
8.4	-18.4564	-0.07715	-32.1146	-0.00045	-16.4986	-0.07907
8.41	-18.3518	-0.07893	-32.6923	-0.00031	-16.5959	-0.07701
8.42	-18.2479	-0.08072	-33.3143	-0.0002	-16.6948	-0.07496
8.43	-18.1449	-0.08255	-33.9877	-0.00012	-16.7954	-0.07294
8.44	-18.0426	-0.0844	-34.7215	-5.21E-05	-16.8978	-0.07093
8.45	-17.9411	-0.08628	-35.5269	-1.36E-05	-17.002	-0.06894
8.46	-17.8404	-0.08819	-36.4191	-2.48E-08	-17.1079	-0.06697
8.47	-17.7404	-0.09013	-37.4183	-1.15E-05	-17.2158	-0.06503
8.48	-17.6412	-0.09209	-38.553	-4.84E-05	-17.3255	-0.0631
8.49	-17.5428	-0.09409	-39.8648	-0.00011	-17.4373	-0.06119
8.5	-17.4451	-0.09611	-41.4178	-0.0002	-17.5511	-0.05931
8.51	-17.3481	-0.09815	-43.3193	-0.00031	-17.6669	-0.05745
8.52	-17.2519	-0.10023	-45.7689	-0.00046	-17.7849	-0.05561
8.53	-17.1564	-0.10233	-49.2104	-0.00062	-17.9051	-0.0538
8.54	-17.0616	-0.10447	-55.0273	-0.00082	-18.0275	-0.05201
8.55	-16.9675	-0.10663	-82.4361	-0.00104	-18.1523	-0.05024
8.56	-16.8742	-0.10882	-55.7663	-0.00129	-18.2795	-0.0485
8.57	-16.7815	-0.11103	-49.5298	-0.00157	-18.4091	-0.04678
8.58	-16.6896	-0.11328	-45.9264	-0.00187	-18.5413	-0.04509
8.59	-16.5983	-0.11555	-43.3792	-0.00221	-18.6761	-0.04342
8.6	-16.5078	-0.11786	-41.406	-0.00257	-18.8136	-0.04178
8.61	-16.4179	-0.12019	-39.794	-0.00296	-18.954	-0.04017

8.62	-16.3287	-0.12255	-38.4307	-0.00338	-19.0971	-0.03858
8.63	-16.2402	-0.12493	-37.249	-0.00383	-19.2433	-0.03702
8.64	-16.1524	-0.12735	-36.206	-0.0043	-19.3926	-0.03549
8.65	-16.0652	-0.12979	-35.2721	-0.00481	-19.545	-0.03399
8.66	-15.9787	-0.13226	-34.4266	-0.00534	-19.7008	-0.03251
8.67	-15.8929	-0.13476	-33.654	-0.00591	-19.86	-0.03107
8.68	-15.8077	-0.13729	-32.9426	-0.0065	-20.0227	-0.02965
8.69	-15.7231	-0.13984	-32.2833	-0.00713	-20.1891	-0.02827
8.7	-15.6393	-0.14243	-31.6691	-0.00778	-20.3594	-0.02691
8.71	-15.556	-0.14504	-31.094	-0.00847	-20.5336	-0.02558
8.72	-15.4734	-0.14767	-30.5533	-0.00918	-20.7119	-0.02429
8.73	-15.3915	-0.15034	-30.0432	-0.00993	-20.8946	-0.02303
8.74	-15.3102	-0.15303	-29.5603	-0.0107	-21.0818	-0.02179
8.75	-15.2295	-0.15575	-29.1019	-0.01151	-21.2736	-0.02059
8.76	-15.1494	-0.1585	-28.6655	-0.01234	-21.4703	-0.01943
8.77	-15.07	-0.16127	-28.2493	-0.01321	-21.6722	-0.01829
8.78	-14.9912	-0.16407	-27.8513	-0.01411	-21.8794	-0.01719
8.79	-14.913	-0.16689	-27.4702	-0.01503	-22.0922	-0.01612
8.8	-14.8354	-0.16974	-27.1044	-0.01599	-22.3109	-0.01508
8.81	-14.7584	-0.17262	-26.7529	-0.01698	-22.5357	-0.01407
8.82	-14.6821	-0.17552	-26.4145	-0.018	-22.7671	-0.0131
8.83	-14.6064	-0.17844	-26.0884	-0.01904	-23.0053	-0.01217
8.84	-14.5312	-0.18139	-25.7737	-0.02012	-23.2508	-0.01126
8.85	-14.4567	-0.18437	-25.4697	-0.02123	-23.5038	-0.01039
8.86	-14.3828	-0.18737	-25.1756	-0.02237	-23.765	-0.00956
8.87	-14.3095	-0.19039	-24.8909	-0.02354	-24.0347	-0.00876
8.88	-14.2367	-0.19344	-24.615	-0.02474	-24.3135	-0.00799
8.89	-14.1646	-0.1965	-24.3474	-0.02596	-24.6021	-0.00726
8.9	-14.0931	-0.1996	-24.0876	-0.02722	-24.9009	-0.00657
8.91	-14.0221	-0.20271	-23.8352	-0.02851	-25.2108	-0.00591
8.92	-13.9518	-0.20584	-23.5899	-0.02982	-25.5326	-0.00528
8.93	-13.882	-0.209	-23.3512	-0.03117	-25.8671	-0.00469
8.94	-13.8129	-0.21218	-23.1189	-0.03254	-26.2153	-0.00413
8.95	-13.7443	-0.21537	-22.8926	-0.03394	-26.5785	-0.00361
8.96	-13.6763	-0.21859	-22.672	-0.03537	-26.9577	-0.00313
8.97	-13.6089	-0.22182	-22.457	-0.03683	-27.3545	-0.00268
8.98	-13.542	-0.22508	-22.2472	-0.03831	-27.7704	-0.00226
8.99	-13.4758	-0.22835	-22.0425	-0.03983	-28.2075	-0.00188
9	-13.4101	-0.23164	-21.8425	-0.04137	-28.6678	-0.00154
9.01	-13.345	-0.23495	-21.6472	-0.04293	-29.1539	-0.00123
9.02	-13.2805	-0.23827	-21.4564	-0.04452	-29.6689	-0.00096
9.03	-13.2165	-0.24161	-21.2698	-0.04614	-30.2162	-0.00072
9.04	-13.1532	-0.24496	-21.0874	-0.04778	-30.8001	-0.00051
9.05	-13.0904	-0.24833	-20.9089	-0.04945	-31.4259	-0.00034
9.06	-13.0282	-0.25171	-20.7343	-0.05114	-32.0998	-0.00021
9.07	-12.9666	-0.2551	-20.5633	-0.05286	-32.8299	-0.00011
9.08	-12.9055	-0.25851	-20.3959	-0.0546	-33.6264	-3.80E-05
9.09	-12.845	-0.26192	-20.232	-0.05636	-34.5023	-4.26E-06

9.1	-12.7851	-0.26535	-20.0715	-0.05815	-35.4754	-4.09E-06
9.11	-12.7258	-0.26878	-19.9141	-0.05995	-36.5699	-3.72E-05
9.12	-12.667	-0.27223	-19.76	-0.06178	-37.8205	-0.0001
9.13	-12.6088	-0.27568	-19.6088	-0.06363	-39.2793	-0.0002
9.14	-12.5512	-0.27914	-19.4607	-0.06549	-41.0301	-0.00033
9.15	-12.4941	-0.2826	-19.3154	-0.06738	-43.2202	-0.0005
9.16	-12.4377	-0.28607	-19.1729	-0.06929	-46.1484	-0.00069
9.17	-12.3818	-0.28955	-19.0332	-0.07121	-50.5852	-0.00092
9.18	-12.3264	-0.29302	-18.8961	-0.07315	-60.0832	-0.00117
9.19	-12.2717	-0.2965	-18.7617	-0.0751	-60.2639	-0.00146
9.2	-12.2175	-0.29998	-18.6297	-0.07708	-50.6774	-0.00177
9.21	-12.1639	-0.30346	-18.5002	-0.07906	-46.2423	-0.00212
9.22	-12.1109	-0.30694	-18.3732	-0.08106	-43.3285	-0.00249
9.23	-12.0585	-0.31041	-18.2485	-0.08307	-41.1571	-0.00289
9.24	-12.0066	-0.31388	-18.1261	-0.0851	-39.4272	-0.00332
9.25	-11.9553	-0.31735	-18.0059	-0.08713	-37.9904	-0.00378
9.26	-11.9046	-0.32081	-17.888	-0.08918	-36.7625	-0.00426
9.27	-11.8545	-0.32427	-17.7723	-0.09123	-35.6912	-0.00477
9.28	-11.805	-0.32771	-17.6587	-0.09329	-34.7417	-0.0053
9.29	-11.756	-0.33114	-17.5471	-0.09536	-33.8897	-0.00586
9.3	-11.7077	-0.33457	-17.4377	-0.09744	-33.1176	-0.00644
9.31	-11.6599	-0.33798	-17.3302	-0.09952	-32.4121	-0.00705
9.32	-11.6127	-0.34138	-17.2247	-0.1016	-31.763	-0.00767
9.33	-11.5661	-0.34476	-17.1212	-0.10369	-31.1624	-0.00832
9.34	-11.5202	-0.34812	-17.0195	-0.10577	-30.6039	-0.00898
9.35	-11.4748	-0.35147	-16.9198	-0.10786	-30.0824	-0.00967
9.36	-11.43	-0.3548	-16.8219	-0.10995	-29.5936	-0.01037
9.37	-11.3858	-0.3581	-16.7259	-0.11203	-29.1338	-0.0111
9.38	-11.3422	-0.36138	-16.6316	-0.11411	-28.7002	-0.01183
9.39	-11.2992	-0.36464	-16.5392	-0.11619	-28.2903	-0.01259
9.4	-11.2568	-0.36787	-16.4485	-0.11826	-27.9018	-0.01336
9.41	-11.2151	-0.37108	-16.3596	-0.12032	-27.5328	-0.01414
9.42	-11.1739	-0.37425	-16.2723	-0.12237	-27.1819	-0.01493
9.43	-11.1334	-0.37739	-16.1868	-0.12441	-26.8474	-0.01574
9.44	-11.0935	-0.3805	-16.103	-0.12644	-26.5283	-0.01655
9.45	-11.0542	-0.38358	-16.0208	-0.12846	-26.2233	-0.01738
9.46	-11.0156	-0.38662	-15.9403	-0.13046	-25.9316	-0.01821
9.47	-10.9776	-0.38962	-15.8615	-0.13244	-25.6522	-0.01905
9.48	-10.9402	-0.39258	-15.7842	-0.13441	-25.3844	-0.0199
9.49	-10.9035	-0.3955	-15.7086	-0.13635	-25.1275	-0.02075
9.5	-10.8674	-0.39837	-15.6346	-0.13828	-24.8808	-0.0216
9.51	-10.832	-0.4012	-15.5622	-0.14018	-24.6438	-0.02246
9.52	-10.7972	-0.40398	-15.4913	-0.14206	-24.416	-0.02331
9.53	-10.7631	-0.40671	-15.4221	-0.14391	-24.1969	-0.02416
9.54	-10.7297	-0.40938	-15.3544	-0.14573	-23.986	-0.02502
9.55	-10.6969	-0.412	-15.2883	-0.14752	-23.7831	-0.02587
9.56	-10.6648	-0.41457	-15.2237	-0.14928	-23.5878	-0.02671
9.57	-10.6334	-0.41708	-15.1607	-0.15101	-23.3997	-0.02755

9.58	-10.6027	-0.41952	-15.0992	-0.1527	-23.2185	-0.02838
9.59	-10.5726	-0.4219	-15.0393	-0.15435	-23.044	-0.0292
9.6	-10.5433	-0.42422	-14.9809	-0.15597	-22.8759	-0.03001
9.61	-10.5147	-0.42647	-14.9241	-0.15754	-22.714	-0.0308
9.62	-10.4868	-0.42865	-14.8688	-0.15907	-22.5582	-0.03159
9.63	-10.4596	-0.43076	-14.8151	-0.16056	-22.4081	-0.03236
9.64	-10.4332	-0.43279	-14.7629	-0.162	-22.2636	-0.03311
9.65	-10.4075	-0.43475	-14.7122	-0.16339	-22.1247	-0.03384
9.66	-10.3826	-0.43662	-14.6631	-0.16473	-21.9911	-0.03456
9.67	-10.3584	-0.43842	-14.6156	-0.16602	-21.8626	-0.03525
9.68	-10.3349	-0.44012	-14.5696	-0.16725	-21.7393	-0.03592
9.69	-10.3123	-0.44175	-14.5252	-0.16842	-21.6209	-0.03657
9.7	-10.2904	-0.44328	-14.4824	-0.16954	-21.5073	-0.03719
9.71	-10.2694	-0.44472	-14.4412	-0.17059	-21.3986	-0.03778
9.72	-10.2491	-0.44606	-14.4015	-0.17158	-21.2945	-0.03835
9.73	-10.2297	-0.44731	-14.3635	-0.17251	-21.195	-0.03888
9.74	-10.211	-0.44846	-14.327	-0.17337	-21.1001	-0.03939
9.75	-10.1933	-0.4495	-14.2923	-0.17416	-21.0097	-0.03986
9.76	-10.1764	-0.45044	-14.2591	-0.17488	-20.9238	-0.04029
9.77	-10.1603	-0.45127	-14.2276	-0.17552	-20.8422	-0.04069
9.78	-10.1451	-0.45199	-14.1978	-0.17609	-20.765	-0.04105
9.79	-10.1308	-0.4526	-14.1697	-0.17658	-20.6922	-0.04137
9.8	-10.1175	-0.45309	-14.1433	-0.17699	-20.6237	-0.04166
9.81	-10.105	-0.45346	-14.1186	-0.17732	-20.5595	-0.0419
9.82	-10.0935	-0.45371	-14.0957	-0.17757	-20.4996	-0.04209
9.83	-10.0829	-0.45383	-14.0745	-0.17773	-20.444	-0.04225
9.84	-10.0733	-0.45383	-14.0552	-0.1778	-20.3927	-0.04235
9.85	-10.0647	-0.45369	-14.0377	-0.17778	-20.3458	-0.04241
9.86	-10.0571	-0.45343	-14.022	-0.17767	-20.3032	-0.04243
9.87	-10.0506	-0.45302	-14.0083	-0.17746	-20.265	-0.04239
9.88	-10.045	-0.45248	-13.9964	-0.17716	-20.2312	-0.0423
9.89	-10.0406	-0.4518	-13.9865	-0.17676	-20.2018	-0.04217
9.9	-10.0372	-0.45097	-13.9786	-0.17627	-20.177	-0.04198
9.91	-10.035	-0.44999	-13.9727	-0.17567	-20.1567	-0.04173
9.92	-10.0338	-0.44887	-13.9689	-0.17497	-20.1411	-0.04144
9.93	-10.0339	-0.44759	-13.9672	-0.17416	-20.1301	-0.04109
9.94	-10.0351	-0.44615	-13.9677	-0.17325	-20.124	-0.04069
9.95	-10.0375	-0.44456	-13.9703	-0.17223	-20.1227	-0.04023
9.96	-10.0412	-0.4428	-13.9752	-0.1711	-20.1264	-0.03972
9.97	-10.0461	-0.44089	-13.9824	-0.16986	-20.1353	-0.03915
9.98	-10.0524	-0.4388	-13.992	-0.16851	-20.1493	-0.03853
9.99	-10.0599	-0.43655	-14.004	-0.16705	-20.1688	-0.03785
10	-10.0689	-0.43413	-14.0185	-0.16547	-20.1937	-0.03712
10.01	-10.0792	-0.43153	-14.0355	-0.16378	-20.2244	-0.03633
10.02	-10.091	-0.42876	-14.0551	-0.16197	-20.2609	-0.03549
10.03	-10.1042	-0.4258	-14.0775	-0.16005	-20.3035	-0.0346
10.04	-10.1189	-0.42267	-14.1026	-0.15801	-20.3524	-0.03366
10.05	-10.1352	-0.41936	-14.1306	-0.15586	-20.4079	-0.03267

10.06	-10.1532	-0.41586	-14.1616	-0.15359	-20.4701	-0.03162
10.07	-10.1727	-0.41217	-14.1956	-0.1512	-20.5394	-0.03054
10.08	-10.1939	-0.4083	-14.2327	-0.14869	-20.6161	-0.0294
10.09	-10.2169	-0.40423	-14.2731	-0.14607	-20.7005	-0.02823
10.1	-10.2417	-0.39997	-14.3169	-0.14334	-20.7931	-0.02701
10.11	-10.2684	-0.39552	-14.3641	-0.14049	-20.8941	-0.02576
10.12	-10.2969	-0.39088	-14.415	-0.13752	-21.0042	-0.02448
10.13	-10.3274	-0.38604	-14.4697	-0.13445	-21.1237	-0.02316
10.14	-10.36	-0.38101	-14.5283	-0.13126	-21.2532	-0.02182
10.15	-10.3947	-0.37578	-14.5909	-0.12797	-21.3934	-0.02046
10.16	-10.4315	-0.37035	-14.6578	-0.12457	-21.5449	-0.01908
10.17	-10.4707	-0.36472	-14.7291	-0.12108	-21.7085	-0.01769
10.18	-10.5121	-0.3589	-14.805	-0.11748	-21.8851	-0.01629
10.19	-10.556	-0.35288	-14.8858	-0.11378	-22.0755	-0.0149
10.2	-10.6024	-0.34667	-14.9716	-0.11	-22.2809	-0.01351
10.21	-10.6514	-0.34025	-15.0627	-0.10613	-22.5024	-0.01213
10.22	-10.7031	-0.33365	-15.1594	-0.10217	-22.7416	-0.01078
10.23	-10.7576	-0.32686	-15.2619	-0.09814	-22.9999	-0.00946
10.24	-10.8151	-0.31987	-15.3706	-0.09404	-23.2791	-0.00818
10.25	-10.8756	-0.3127	-15.4857	-0.08987	-23.5815	-0.00695
10.26	-10.9393	-0.30534	-15.6077	-0.08565	-23.9094	-0.00577
10.27	-11.0063	-0.2978	-15.7369	-0.08138	-24.2658	-0.00467
10.28	-11.0768	-0.29009	-15.8739	-0.07707	-24.6541	-0.00365
10.29	-11.1509	-0.2822	-16.0189	-0.07273	-25.0783	-0.00273
10.3	-11.2288	-0.27415	-16.1726	-0.06837	-25.5436	-0.00191
10.31	-11.3107	-0.26594	-16.3356	-0.06399	-26.056	-0.00122
10.32	-11.3967	-0.25757	-16.5084	-0.05962	-26.6233	-0.00067
10.33	-11.4871	-0.24905	-16.6917	-0.05526	-27.255	-0.00027
10.34	-11.582	-0.2404	-16.8864	-0.05093	-27.9639	-4.53E-05
10.35	-11.6818	-0.23161	-17.0932	-0.04664	-28.7667	-1.09E-05
10.36	-11.7866	-0.2227	-17.3132	-0.04241	-29.6862	-0.00019
10.37	-11.8968	-0.21369	-17.5474	-0.03826	-30.755	-0.00059
10.38	-12.0126	-0.20457	-17.7971	-0.03419	-32.0216	-0.00125
10.39	-12.1343	-0.19537	-18.0636	-0.03023	-33.5628	-0.00219
10.4	-12.2623	-0.18609	-18.3485	-0.02641	-35.5115	-0.00342
10.41	-12.3971	-0.17675	-18.6536	-0.02274	-38.1292	-0.00499
10.42	-12.5389	-0.16738	-18.9811	-0.01924	-42.0558	-0.0069
10.43	-12.6882	-0.15797	-19.3333	-0.01594	-49.8151	-0.00919
10.44	-12.8455	-0.14856	-19.7132	-0.01287	-55.995	-0.0119
10.45	-13.0114	-0.13917	-20.1241	-0.01005	-43.6637	-0.01505
10.46	-13.1865	-0.1298	-20.57	-0.00751	-38.6429	-0.01868
10.47	-13.3713	-0.1205	-21.0559	-0.00528	-35.3983	-0.02281
10.48	-13.5667	-0.11128	-21.5879	-0.00339	-32.9774	-0.02749
10.49	-13.7734	-0.10217	-22.1733	-0.00189	-31.0343	-0.03276
10.5	-13.9923	-0.09319	-22.8217	-0.0008	-29.4035	-0.03865
10.51	-14.2244	-0.08439	-23.5454	-0.00016	-27.9933	-0.04521
10.52	-14.4708	-0.07578	-24.3604	-1.21E-05	-26.7471	-0.05247
10.53	-14.7328	-0.06741	-25.2891	-0.0004	-25.6279	-0.06049

10.54	-15.0119	-0.05932	-26.3625	-0.00137	-24.6099	-0.06931
10.55	-15.3097	-0.05153	-27.6269	-0.00296	-23.6745	-0.07899
10.56	-15.6281	-0.04409	-29.1551	-0.00522	-22.8078	-0.08956
10.57	-15.9692	-0.03705	-31.0715	-0.00821	-21.9994	-0.10108
10.58	-16.3357	-0.03046	-33.6166	-0.01198	-21.2408	-0.11361
10.59	-16.7304	-0.02435	-37.3605	-0.01658	-20.5256	-0.12721
10.6	-17.1571	-0.01879	-44.3736	-0.02207	-19.8483	-0.14192
10.61	-17.6199	-0.01383	-55.5372	-0.02852	-19.2047	-0.15782
10.62	-18.124	-0.00953	-40.3561	-0.03597	-18.591	-0.17496
10.63	-18.6758	-0.00594	-35.0214	-0.04451	-18.0042	-0.1934
10.64	-19.2832	-0.00314	-31.6685	-0.05419	-17.4418	-0.21322
10.65	-19.9563	-0.00118	-29.2005	-0.06509	-16.9016	-0.23447
10.66	-20.7082	-0.00015	-27.2365	-0.07727	-16.3816	-0.25722
10.67	-21.5564	-0.00011	-25.5988	-0.09082	-15.8803	-0.28154
10.68	-22.5248	-0.00114	-24.1898	-0.1058	-15.3962	-0.30751
10.69	-23.6477	-0.00332	-22.9501	-0.1223	-14.9281	-0.33519
10.7	-24.9765	-0.00674	-21.8411	-0.14039	-14.4749	-0.36466
10.71	-26.5933	-0.01148	-20.8361	-0.16016	-14.0357	-0.39598
10.72	-28.6424	-0.01763	-19.9157	-0.1817	-13.6096	-0.42923
10.73	-31.4137	-0.02528	-19.0658	-0.20507	-13.1958	-0.46448
10.74	-35.6462	-0.03453	-18.2755	-0.23038	-12.7938	-0.50181
10.75	-44.6208	-0.04548	-17.5363	-0.25771	-12.4028	-0.54129
10.76	-45.9699	-0.05824	-16.8416	-0.28714	-12.0224	-0.58299
10.77	-35.8072	-0.07289	-16.1859	-0.31877	-11.6521	-0.62698
10.78	-31.1651	-0.08956	-15.5647	-0.35267	-11.2914	-0.67333
10.79	-28.0955	-0.10835	-14.9745	-0.38895	-10.9401	-0.72212
10.8	-25.7856	-0.12936	-14.4121	-0.42768	-10.5976	-0.7734
10.81	-23.9253	-0.15271	-13.875	-0.46896	-10.2638	-0.82725
10.82	-22.363	-0.17852	-13.3609	-0.51286	-9.93831	-0.88372
10.83	-21.013	-0.20688	-12.8681	-0.55948	-9.62091	-0.94289
10.84	-19.822	-0.23792	-12.3948	-0.60888	-9.31137	-1.00479
10.85	-18.755	-0.27175	-11.9397	-0.66115	-9.00947	-1.0695
10.86	-17.7874	-0.30847	-11.5015	-0.71637	-8.71502	-1.13706
10.87	-16.9014	-0.3482	-11.0792	-0.77461	-8.42786	-1.20752
10.88	-16.0838	-0.39104	-10.6719	-0.83593	-8.14782	-1.28093
10.89	-15.3244	-0.4371	-10.2787	-0.90041	-7.87476	-1.35731
10.9	-14.6152	-0.48648	-9.89886	-0.96809	-7.60855	-1.43671
10.91	-13.95	-0.53926	-9.53175	-1.03904	-7.34905	-1.51916
10.92	-13.3237	-0.59555	-9.17677	-1.1133	-7.09617	-1.60467
10.93	-12.7319	-0.65544	-8.83341	-1.19091	-6.84979	-1.69328
10.94	-12.1713	-0.71899	-8.5012	-1.27193	-6.6098	-1.785
10.95	-11.639	-0.78628	-8.17971	-1.35637	-6.37613	-1.87983
10.96	-11.1325	-0.85739	-7.86856	-1.44426	-6.14867	-1.97779
10.97	-10.6496	-0.93237	-7.56739	-1.53562	-5.92733	-2.07887
10.98	-10.1887	-1.01127	-7.27588	-1.63046	-5.71205	-2.18306
10.99	-9.7482	-1.09414	-6.99374	-1.72879	-5.50272	-2.29035
11	-9.32668	-1.181	-6.7207	-1.83061	-5.29928	-2.40074
11.01	-8.92301	-1.27189	-6.45649	-1.93591	-5.10164	-2.51419

11.02	-8.53614	-1.36681	-6.20089	-2.04467	-4.90971	-2.63068
11.03	-8.16516	-1.46579	-5.95367	-2.15687	-4.72343	-2.75018
11.04	-7.80926	-1.5688	-5.71462	-2.27249	-4.54271	-2.87266
11.05	-7.46769	-1.67585	-5.48353	-2.39149	-4.36747	-2.99808
11.06	-7.1398	-1.78691	-5.26022	-2.51383	-4.19763	-3.12638
11.07	-6.82499	-1.90194	-5.04449	-2.63947	-4.03309	-3.25753
11.08	-6.52272	-2.02092	-4.83617	-2.76835	-3.87378	-3.39148
11.09	-6.23247	-2.14379	-4.63508	-2.90042	-3.71961	-3.52817
11.1	-5.95379	-2.2705	-4.44105	-3.03562	-3.57048	-3.66754
11.11	-5.68625	-2.40099	-4.25391	-3.17389	-3.42631	-3.80953
11.12	-5.42944	-2.53518	-4.07349	-3.31515	-3.28699	-3.95409
11.13	-5.18298	-2.673	-3.89964	-3.45934	-3.15244	-4.10115
11.14	-4.94651	-2.81437	-3.73217	-3.60639	-3.02256	-4.25064
11.15	-4.71969	-2.9592	-3.57094	-3.7562	-2.89724	-4.4025
11.16	-4.50219	-3.1074	-3.41578	-3.90872	-2.77639	-4.55666
11.17	-4.2937	-3.25887	-3.26653	-4.06384	-2.65989	-4.71305
11.18	-4.09391	-3.41351	-3.12303	-4.2215	-2.54766	-4.8716
11.19	-3.90252	-3.57122	-2.98513	-4.38161	-2.43958	-5.03224
11.2	-3.71927	-3.73189	-2.85265	-4.54408	-2.33554	-5.19491
11.21	-3.54385	-3.89543	-2.72545	-4.70884	-2.23544	-5.35953
11.22	-3.37602	-4.06172	-2.60337	-4.87579	-2.13918	-5.52604
11.23	-3.21549	-4.23065	-2.48624	-5.04486	-2.04664	-5.69436
11.24	-3.06201	-4.40213	-2.37392	-5.21596	-1.95771	-5.86444
11.25	-2.91533	-4.57603	-2.26626	-5.38901	-1.8723	-6.03619
11.26	-2.77519	-4.75225	-2.16309	-5.56393	-1.79029	-6.20957
11.27	-2.64137	-4.9307	-2.06427	-5.74064	-1.71158	-6.3845
11.28	-2.51361	-5.11126	-1.96966	-5.91906	-1.63605	-6.56092
11.29	-2.39169	-5.29384	-1.87909	-6.09912	-1.56362	-6.73878
11.3	-2.27538	-5.47833	-1.79244	-6.28074	-1.49417	-6.918
11.31	-2.16446	-5.66465	-1.70955	-6.46386	-1.4276	-7.09854
11.32	-2.05871	-5.85269	-1.6303	-6.6484	-1.36382	-7.28034
11.33	-1.95793	-6.04236	-1.55453	-6.83429	-1.30272	-7.46334
11.34	-1.8619	-6.23358	-1.48212	-7.02147	-1.2442	-7.6475
11.35	-1.77042	-6.42626	-1.41294	-7.20988	-1.18818	-7.83275
11.36	-1.68331	-6.62033	-1.34687	-7.39945	-1.13456	-8.01906
11.37	-1.60038	-6.81569	-1.28377	-7.59013	-1.08325	-8.20638
11.38	-1.52144	-7.01228	-1.22353	-7.78186	-1.03416	-8.39465
11.39	-1.44631	-7.21003	-1.16602	-7.9746	-0.98721	-8.58385
11.4	-1.37483	-7.40887	-1.11115	-8.16828	-0.94231	-8.77392
11.41	-1.30683	-7.60872	-1.05879	-8.36286	-0.89938	-8.96483
11.42	-1.24216	-7.80954	-1.00884	-8.55829	-0.85834	-9.15655
11.43	-1.18065	-8.01126	-0.96119	-8.75454	-0.81912	-9.34902
11.44	-1.12217	-8.21383	-0.91576	-8.95156	-0.78164	-9.54224
11.45	-1.06657	-8.41719	-0.87243	-9.1493	-0.74582	-9.73615
11.46	-1.01371	-8.62129	-0.83112	-9.34774	-0.71161	-9.93073
11.47	-0.96347	-8.82609	-0.79174	-9.54683	-0.67893	-10.126
11.48	-0.91572	-9.03155	-0.75421	-9.74655	-0.64771	-10.3218
11.49	-0.87034	-9.23761	-0.71843	-9.94686	-0.6179	-10.5182

11.5	-0.82721	-9.44426	-0.68433	-10.1477	-0.58944	-10.7152
11.51	-0.78623	-9.65144	-0.65184	-10.3492	-0.56226	-10.9128
11.52	-0.74729	-9.85913	-0.62088	-10.5511	-0.53632	-11.1108
11.53	-0.71028	-10.0673	-0.59137	-10.7535	-0.51155	-11.3094
11.54	-0.67513	-10.2759	-0.56327	-10.9564	-0.48791	-11.5084
11.55	-0.64172	-10.4849	-0.53649	-11.1597	-0.46534	-11.708
11.56	-0.60998	-10.6944	-0.51098	-11.3635	-0.4438	-11.9079
11.57	-0.57983	-10.9042	-0.48667	-11.5677	-0.42325	-12.1083
11.58	-0.55118	-11.1144	-0.46352	-11.7722	-0.40363	-12.3092
11.59	-0.52395	-11.3249	-0.44147	-11.9772	-0.38492	-12.5104
11.6	-0.49809	-11.5357	-0.42046	-12.1825	-0.36706	-12.7121
11.61	-0.47351	-11.7468	-0.40045	-12.3882	-0.35001	-12.9142
11.62	-0.45016	-11.9582	-0.38139	-12.5942	-0.33375	-13.1166
11.63	-0.42797	-12.1699	-0.36323	-12.8006	-0.31824	-13.3194
11.64	-0.40689	-12.3819	-0.34593	-13.0073	-0.30344	-13.5226
11.65	-0.38685	-12.5941	-0.32946	-13.2143	-0.28932	-13.7262
11.66	-0.36781	-12.8066	-0.31377	-13.4217	-0.27585	-13.9301
11.67	-0.34972	-13.0193	-0.29882	-13.6293	-0.263	-14.1344
11.68	-0.33252	-13.2323	-0.28459	-13.8373	-0.25074	-14.339
11.69	-0.31618	-13.4455	-0.27103	-14.0455	-0.23904	-14.544
11.7	-0.30064	-13.6589	-0.25811	-14.2541	-0.22788	-14.7494
11.71	-0.28588	-13.8726	-0.24581	-14.463	-0.21724	-14.9551
11.72	-0.27184	-14.0865	-0.23409	-14.6722	-0.20709	-15.1611
11.73	-0.2585	-14.3006	-0.22292	-14.8816	-0.1974	-15.3675
11.74	-0.24582	-14.5149	-0.21229	-15.0914	-0.18817	-15.5742
11.75	-0.23376	-14.7295	-0.20216	-15.3015	-0.17935	-15.7813
11.76	-0.22229	-14.9444	-0.19251	-15.5119	-0.17095	-15.9887
11.77	-0.21139	-15.1594	-0.18331	-15.7226	-0.16293	-16.1965
11.78	-0.20103	-15.3747	-0.17455	-15.9336	-0.15528	-16.4047
11.79	-0.19117	-15.5903	-0.16621	-16.1449	-0.14799	-16.6132
11.8	-0.1818	-15.8061	-0.15826	-16.3566	-0.14103	-16.8221
11.81	-0.17288	-16.0221	-0.15069	-16.5686	-0.13439	-17.0314
11.82	-0.16441	-16.2385	-0.14347	-16.7809	-0.12806	-17.2411
11.83	-0.15635	-16.455	-0.1366	-16.9935	-0.12203	-17.4511
11.84	-0.14868	-16.6719	-0.13005	-17.2065	-0.11627	-17.6615
11.85	-0.14138	-16.8891	-0.12381	-17.4199	-0.11077	-17.8724
11.86	-0.13445	-17.1065	-0.11787	-17.6336	-0.10554	-18.0836
11.87	-0.12785	-17.3242	-0.11221	-17.8477	-0.10054	-18.2952
11.88	-0.12157	-17.5423	-0.10681	-18.0621	-0.09577	-18.5073
11.89	-0.1156	-17.7607	-0.10167	-18.277	-0.09123	-18.7198
11.9	-0.10992	-17.9794	-0.09677	-18.4922	-0.08689	-18.9327
11.91	-0.10451	-18.1985	-0.09211	-18.7079	-0.08276	-19.1461
11.92	-0.09937	-18.4179	-0.08766	-18.9239	-0.07882	-19.36
11.93	-0.09447	-18.6376	-0.08342	-19.1404	-0.07505	-19.5743
11.94	-0.08982	-18.8578	-0.07939	-19.3574	-0.07147	-19.7891
11.95	-0.08539	-19.0783	-0.07554	-19.5747	-0.06805	-20.0044
11.96	-0.08117	-19.2993	-0.07188	-19.7926	-0.06479	-20.2202
11.97	-0.07716	-19.5206	-0.06839	-20.0109	-0.06168	-20.4365

11.98	-0.07335	-19.7424	-0.06506	-20.2297	-0.05871	-20.6533
11.99	-0.06971	-19.9646	-0.06189	-20.449	-0.05589	-20.8706
12	-0.06626	-20.1873	-0.05888	-20.6688	-0.05319	-21.0885
12.01	-0.06297	-20.4104	-0.056	-20.8891	-0.05062	-21.307
12.02	-0.05984	-20.6341	-0.05326	-21.11	-0.04817	-21.526
12.03	-0.05687	-20.8582	-0.05065	-21.3314	-0.04583	-21.7456
12.04	-0.05403	-21.0828	-0.04816	-21.5534	-0.0436	-21.9659
12.05	-0.05134	-21.308	-0.04579	-21.7759	-0.04148	-22.1867
12.06	-0.04877	-21.5337	-0.04354	-21.9991	-0.03946	-22.4082
12.07	-0.04633	-21.76	-0.04139	-22.2229	-0.03753	-22.6303
12.08	-0.04401	-21.9869	-0.03934	-22.4473	-0.03569	-22.8531
12.09	-0.0418	-22.2143	-0.03739	-22.6723	-0.03393	-23.0765
12.1	-0.03969	-22.4424	-0.03553	-22.898	-0.03226	-23.3007
12.11	-0.03769	-22.6711	-0.03377	-23.1244	-0.03067	-23.5255
12.12	-0.03579	-22.9005	-0.03208	-23.3515	-0.02915	-23.7511
12.13	-0.03398	-23.1305	-0.03048	-23.5792	-0.02771	-23.9774
12.14	-0.03225	-23.3612	-0.02895	-23.8078	-0.02633	-24.2045
12.15	-0.03062	-23.5926	-0.02749	-24.037	-0.02502	-24.4323
12.16	-0.02906	-23.8247	-0.02611	-24.267	-0.02377	-24.6609
12.17	-0.02757	-24.0576	-0.02479	-24.4978	-0.02257	-24.8904
12.18	-0.02616	-24.2912	-0.02354	-24.7295	-0.02144	-25.1207
12.19	-0.02482	-24.5257	-0.02234	-24.9619	-0.02036	-25.3518
12.2	-0.02354	-24.7609	-0.0212	-25.1952	-0.01933	-25.5838
12.21	-0.02233	-24.9969	-0.02012	-25.4293	-0.01835	-25.8167
12.22	-0.02117	-25.2338	-0.01909	-25.6643	-0.01741	-26.0505
12.23	-0.02008	-25.4716	-0.01811	-25.9003	-0.01653	-26.2852
12.24	-0.01903	-25.7103	-0.01718	-26.1371	-0.01568	-26.5209
12.25	-0.01804	-25.9498	-0.01629	-26.3749	-0.01487	-26.7575
12.26	-0.0171	-26.1903	-0.01544	-26.6137	-0.01411	-26.9952
12.27	-0.0162	-26.4318	-0.01464	-26.8535	-0.01338	-27.2338
12.28	-0.01535	-26.6743	-0.01388	-27.0942	-0.01268	-27.4735
12.29	-0.01454	-26.9177	-0.01315	-27.3361	-0.01202	-27.7143
12.3	-0.01377	-27.1622	-0.01246	-27.579	-0.01139	-27.9561
12.31	-0.01303	-27.4078	-0.0118	-27.823	-0.0108	-28.1991
12.32	-0.01234	-27.6544	-0.01118	-28.0681	-0.01023	-28.4432
12.33	-0.01168	-27.9022	-0.01058	-28.3143	-0.00969	-28.6885
12.34	-0.01105	-28.1511	-0.01002	-28.5617	-0.00917	-28.9349
12.35	-0.01045	-28.4011	-0.00948	-28.8103	-0.00868	-29.1826
12.36	-0.00989	-28.6524	-0.00897	-29.0602	-0.00822	-29.4315
12.37	-0.00935	-28.9049	-0.00849	-29.3113	-0.00778	-29.6817
12.38	-0.00884	-29.1586	-0.00803	-29.5636	-0.00736	-29.9332
12.39	-0.00836	-29.4136	-0.00759	-29.8173	-0.00696	-30.186
12.4	-0.0079	-29.67	-0.00718	-30.0723	-0.00658	-30.4402
12.41	-0.00746	-29.9277	-0.00678	-30.3287	-0.00622	-30.6957
12.42	-0.00705	-30.1867	-0.00641	-30.5865	-0.00588	-30.9527
12.43	-0.00665	-30.4472	-0.00605	-30.8458	-0.00555	-31.2111
12.44	-0.00628	-30.7091	-0.00572	-31.1065	-0.00525	-31.4711
12.45	-0.00593	-30.9725	-0.0054	-31.3687	-0.00495	-31.7325

12.46	-0.00559	-31.2375	-0.00509	-31.6324	-0.00468	-31.9955
12.47	-0.00527	-31.5039	-0.0048	-31.8977	-0.00441	-32.2601
12.48	-0.00497	-31.772	-0.00453	-32.1647	-0.00416	-32.5263
12.49	-0.00469	-32.0417	-0.00427	-32.4333	-0.00393	-32.7942
12.5	-0.00442	-32.313	-0.00403	-32.7035	-0.0037	-33.0637
12.51	-0.00416	-32.5861	-0.0038	-32.9755	-0.00349	-33.3351
12.52	-0.00392	-32.8609	-0.00358	-33.2493	-0.00329	-33.6082
12.53	-0.00369	-33.1375	-0.00337	-33.5249	-0.0031	-33.8831
12.54	-0.00347	-33.4159	-0.00317	-33.8023	-0.00292	-34.1599
12.55	-0.00327	-33.6962	-0.00298	-34.0816	-0.00274	-34.4386
12.56	-0.00307	-33.9784	-0.00281	-34.3628	-0.00258	-34.7192
12.57	-0.00289	-34.2626	-0.00264	-34.6461	-0.00243	-35.0018
12.58	-0.00271	-34.5488	-0.00248	-34.9313	-0.00228	-35.2865
12.59	-0.00255	-34.837	-0.00233	-35.2187	-0.00215	-35.5733
12.6	-0.00239	-35.1274	-0.00219	-35.5082	-0.00202	-35.8622
12.61	-0.00225	-35.4199	-0.00206	-35.7998	-0.00189	-36.1533
12.62	-0.00211	-35.7147	-0.00193	-36.0937	-0.00178	-36.4467
12.63	-0.00198	-36.0117	-0.00181	-36.3899	-0.00167	-36.7423
12.64	-0.00185	-36.3111	-0.0017	-36.6885	-0.00156	-37.0404
12.65	-0.00174	-36.6128	-0.00159	-36.9894	-0.00147	-37.3408
12.66	-0.00163	-36.917	-0.00149	-37.2928	-0.00137	-37.6437
12.67	-0.00152	-37.2237	-0.0014	-37.5987	-0.00129	-37.9491
12.68	-0.00143	-37.533	-0.00131	-37.9072	-0.0012	-38.2572
12.69	-0.00133	-37.8449	-0.00122	-38.2184	-0.00113	-38.5679
12.7	-0.00125	-38.1595	-0.00114	-38.5323	-0.00105	-38.8813
12.71	-0.00117	-38.4769	-0.00107	-38.849	-0.00098	-39.1975
12.72	-0.00109	-38.7971	-0.001	-39.1685	-0.00092	-39.5166
12.73	-0.00102	-39.1203	-0.00093	-39.491	-0.00086	-39.8387
12.74	-0.00095	-39.4465	-0.00087	-39.8165	-0.0008	-40.1638
12.75	-0.00088	-39.7757	-0.00081	-40.145	-0.00075	-40.4919
12.76	-0.00082	-40.1081	-0.00076	-40.4768	-0.0007	-40.8233
12.77	-0.00077	-40.4437	-0.0007	-40.8118	-0.00065	-41.1579
12.78	-0.00071	-40.7827	-0.00065	-41.1501	-0.0006	-41.4958
12.79	-0.00066	-41.1251	-0.00061	-41.4919	-0.00056	-41.8373
12.8	-0.00062	-41.471	-0.00057	-41.8373	-0.00052	-42.1822
12.81	-0.00057	-41.8205	-0.00053	-42.1862	-0.00049	-42.5308
12.82	-0.00053	-42.1738	-0.00049	-42.5389	-0.00045	-42.8831
12.83	-0.00049	-42.5308	-0.00045	-42.8954	-0.00042	-43.2393
12.84	-0.00046	-42.8918	-0.00042	-43.2558	-0.00039	-43.5994
12.85	-0.00042	-43.2569	-0.00039	-43.6203	-0.00036	-43.9636
12.86	-0.00039	-43.6261	-0.00036	-43.989	-0.00033	-44.3319
12.87	-0.00036	-43.9996	-0.00033	-44.362	-0.00031	-44.7046
12.88	-0.00034	-44.3774	-0.00031	-44.7394	-0.00028	-45.0817
12.89	-0.00031	-44.7599	-0.00028	-45.1213	-0.00026	-45.4633
12.9	-0.00029	-45.147	-0.00026	-45.508	-0.00024	-45.8497
12.91	-0.00026	-45.539	-0.00024	-45.8994	-0.00022	-46.2409
12.92	-0.00024	-45.9359	-0.00022	-46.2959	-0.00021	-46.637
12.93	-0.00022	-46.338	-0.00021	-46.6975	-0.00019	-47.0384

12.94	-0.00021	-46.7453	-0.00019	-47.1044	-0.00017	-47.445
12.95	-0.00019	-47.1581	-0.00017	-47.5168	-0.00016	-47.8571
12.96	-0.00017	-47.5766	-0.00016	-47.9349	-0.00015	-48.2749
12.97	-0.00016	-48.0009	-0.00015	-48.3587	-0.00013	-48.6986
12.98	-0.00015	-48.4312	-0.00013	-48.7887	-0.00012	-49.1282
12.99	-0.00013	-48.8678	-0.00012	-49.2248	-0.00011	-49.5642
13	-0.00012	-49.3108	-0.00011	-49.6674	-0.0001	-50.0065
13.01	-0.00011	-49.7604	-0.0001	-50.1167	-9.42E-05	-50.4556
13.02	-0.0001	-50.217	-9.29E-05	-50.5729	-8.59E-05	-50.9116
13.03	-9.19E-05	-50.6808	-8.46E-05	-51.0363	-7.82E-05	-51.3748
13.04	-8.36E-05	-51.1519	-7.69E-05	-51.5071	-7.11E-05	-51.8453
13.05	-7.59E-05	-51.6308	-6.99E-05	-51.9856	-6.46E-05	-52.3236
13.06	-6.88E-05	-52.1176	-6.34E-05	-52.4721	-5.86E-05	-52.8099
13.07	-6.23E-05	-52.6127	-5.74E-05	-52.9669	-5.31E-05	-53.3045
13.08	-5.64E-05	-53.1164	-5.19E-05	-53.4703	-4.80E-05	-53.8077
13.09	-5.09E-05	-53.6291	-4.69E-05	-53.9827	-4.34E-05	-54.3199
13.1	-4.59E-05	-54.1511	-4.23E-05	-54.5044	-3.91E-05	-54.8414
13.11	-4.13E-05	-54.6828	-3.81E-05	-55.0358	-3.52E-05	-55.3726
13.12	-3.71E-05	-55.2246	-3.42E-05	-55.5773	-3.16E-05	-55.914
13.13	-3.33E-05	-55.7769	-3.07E-05	-56.1293	-2.84E-05	-56.4658
13.14	-2.98E-05	-56.3402	-2.75E-05	-56.6924	-2.54E-05	-57.0287
13.15	-2.67E-05	-56.915	-2.46E-05	-57.2669	-2.27E-05	-57.6031
13.16	-2.38E-05	-57.5018	-2.19E-05	-57.8534	-2.03E-05	-58.1895
13.17	-2.12E-05	-58.1012	-1.95E-05	-58.4525	-1.81E-05	-58.7884
13.18	-1.88E-05	-58.7137	-1.74E-05	-59.0647	-1.61E-05	-59.4005
13.19	-1.67E-05	-59.3399	-1.54E-05	-59.6908	-1.42E-05	-60.0264
13.2	-1.48E-05	-59.9807	-1.36E-05	-60.3313	-1.26E-05	-60.6667
13.21	-1.30E-05	-60.6366	-1.20E-05	-60.987	-1.11E-05	-61.3223
13.22	-1.15E-05	-61.3085	-1.06E-05	-61.6586	-9.80E-06	-61.9938
13.23	-1.01E-05	-61.9972	-9.30E-06	-62.3471	-8.61E-06	-62.6822
13.24	-8.84E-06	-62.7036	-8.15E-06	-63.0534	-7.54E-06	-63.3883
13.25	-7.72E-06	-63.4288	-7.12E-06	-63.7783	-6.59E-06	-64.1132
13.26	-6.72E-06	-64.1738	-6.20E-06	-64.5231	-5.74E-06	-64.8578
13.27	-5.84E-06	-64.9397	-5.39E-06	-65.2889	-4.99E-06	-65.6235
13.28	-5.06E-06	-65.7279	-4.66E-06	-66.0769	-4.32E-06	-66.4114
13.29	-4.36E-06	-66.5397	-4.02E-06	-66.8885	-3.72E-06	-67.2229
13.3	-3.75E-06	-67.3767	-3.46E-06	-67.7253	-3.20E-06	-68.0596
13.31	-3.21E-06	-68.2404	-2.96E-06	-68.5889	-2.74E-06	-68.9231
13.32	-2.74E-06	-69.1329	-2.53E-06	-69.4812	-2.34E-06	-69.8153
13.33	-2.33E-06	-70.056	-2.15E-06	-70.4041	-1.99E-06	-70.7382
13.34	-1.97E-06	-71.0121	-1.82E-06	-71.3601	-1.68E-06	-71.694
13.35	-1.66E-06	-72.0036	-1.53E-06	-72.3515	-1.42E-06	-72.6853
13.36	-1.39E-06	-73.0334	-1.28E-06	-73.3811	-1.19E-06	-73.7149
13.37	-1.16E-06	-74.1046	-1.07E-06	-74.4522	-9.92E-07	-74.7859
13.38	-9.63E-07	-75.2208	-8.89E-07	-75.5683	-8.23E-07	-75.9019
13.39	-7.95E-07	-76.386	-7.33E-07	-76.7333	-6.79E-07	-77.0669
13.4	-6.51E-07	-77.6047	-6.01E-07	-77.952	-5.57E-07	-78.2855
13.41	-5.30E-07	-78.8824	-4.89E-07	-79.2295	-4.53E-07	-79.5629

13.42	-4.29E-07	-80.2249	-3.96E-07	-80.5719	-3.66E-07	-80.9053
13.43	-3.44E-07	-81.6394	-3.18E-07	-81.9863	-2.94E-07	-82.3197
13.44	-2.74E-07	-83.1342	-2.53E-07	-83.481	-2.34E-07	-83.8143
13.45	-2.16E-07	-84.7189	-1.99E-07	-85.0657	-1.85E-07	-85.3989
13.46	-1.69E-07	-86.4054	-1.56E-07	-86.752	-1.44E-07	-87.0852
13.47	-1.31E-07	-88.2076	-1.20E-07	-88.5542	-1.12E-07	-88.8873
13.48	-9.98E-08	-90.1428	-9.21E-08	-90.4893	-8.53E-08	-90.8224
13.49	-7.54E-08	-92.2323	-6.96E-08	-92.5788	-6.45E-08	-92.9118
13.5	-5.62E-08	-94.5031	-5.19E-08	-94.8495	-4.80E-08	-95.1825
13.51	-4.12E-08	-96.9899	-3.81E-08	-97.3362	-3.53E-08	-97.6692
13.52	-2.98E-08	-99.7382	-2.75E-08	-100.085	-2.55E-08	-100.417
13.53	-2.11E-08	-99.7382	-1.95E-08	-100.085	-1.80E-08	-100.417
13.54	-1.47E-08	-96.9899	-1.35E-08	-97.3362	-1.25E-08	-97.6692
13.55	-9.94E-09	-94.5031	-9.17E-09	-94.8495	-8.50E-09	-95.1825
13.56	-6.56E-09	-92.2323	-6.06E-09	-92.5788	-5.61E-09	-92.9118
13.57	-4.20E-09	-90.1428	-3.88E-09	-90.4893	-3.59E-09	-90.8224
13.58	-2.60E-09	-88.2076	-2.40E-09	-88.5542	-2.22E-09	-88.8873
13.59	-1.54E-09	-86.4054	-1.42E-09	-86.752	-1.32E-09	-87.0852
13.6	-8.69E-10	-84.7189	-8.02E-10	-85.0657	-7.43E-10	-85.3989
13.61	-4.61E-10	-83.1342	-4.26E-10	-83.481	-3.94E-10	-83.8143
13.62	-2.27E-10	-81.6394	-2.10E-10	-81.9863	-1.94E-10	-82.3197
13.63	-1.02E-10	-80.2249	-9.42E-11	-80.5719	-8.72E-11	-80.9053
13.64	-4.04E-11	-78.8824	-3.73E-11	-79.2295	-3.46E-11	-79.5629
13.65	-1.35E-11	-77.6047	-1.25E-11	-77.952	-1.16E-11	-78.2855
13.66	-3.55E-12	-76.386	-3.27E-12	-76.7333	-3.03E-12	-77.0669
13.67	-6.31E-13	-75.2208	-5.83E-13	-75.5683	-5.41E-13	-75.9019
13.68	-5.40E-14	-74.1046	-5.01E-14	-74.4522	-4.82E-14	-74.7859
13.69	0	-73.0334	0	-73.3811	0	-73.7149
13.7	0	-72.0036	0	-72.3515	0	-72.6853
13.71	0	-71.0121	-9.64E-16	-71.3601	0	-71.694
13.72	-5.40E-14	-70.056	-5.01E-14	-70.4041	-4.92E-14	-70.7382
13.73	-6.30E-13	-69.1329	-5.82E-13	-69.4812	-5.41E-13	-69.8153
13.74	-3.55E-12	-68.2404	-3.28E-12	-68.5889	-3.03E-12	-68.9231
13.75	-1.35E-11		-1.25E-11		-1.16E-11	
13.76	-4.04E-11		-3.73E-11		-3.46E-11	
13.77	-1.02E-10		-9.42E-11		-8.72E-11	
13.78	-2.27E-10		-2.10E-10		-1.94E-10	
13.79	-4.61E-10		-4.26E-10		-3.94E-10	
13.8	-8.69E-10		-8.02E-10		-7.43E-10	
13.81	-1.54E-09		-1.42E-09		-1.32E-09	
13.82	-2.60E-09		-2.40E-09		-2.22E-09	
13.83	-4.20E-09		-3.88E-09		-3.59E-09	
13.84	-6.56E-09		-6.06E-09		-5.61E-09	
13.85	-9.94E-09		-9.17E-09		-8.50E-09	
13.86	-1.47E-08		-1.35E-08		-1.25E-08	
13.87	-2.11E-08		-1.95E-08		-1.80E-08	
13.88	-2.98E-08		-2.75E-08		-2.55E-08	
13.89	-4.12E-08		-3.81E-08		-3.53E-08	

13.9	-5.62E-08	-5.19E-08	-4.80E-08
13.91	-7.54E-08	-6.96E-08	-6.45E-08
13.92	-9.98E-08	-9.21E-08	-8.53E-08
13.93	-1.31E-07	-1.20E-07	-1.12E-07
13.94	-1.69E-07	-1.56E-07	-1.44E-07
13.95	-2.16E-07	-1.99E-07	-1.85E-07
13.96	-2.74E-07	-2.53E-07	-2.34E-07
13.97	-3.44E-07	-3.18E-07	-2.94E-07
13.98	-4.29E-07	-3.96E-07	-3.66E-07
13.99	-5.30E-07	-4.89E-07	-4.53E-07
14	-6.51E-07	-6.01E-07	-5.57E-07
