# Supplementary material for: Multilayer UWB bandpass filter using liquid crystal polymer technology
Source: Sci Rep. 2024 Jul 8;14:15669. doi: 10.1038/s41598-024-66928-8 (PMC11231142; doi:10.1038/s41598-024-66928-8)
Supplement: Supplementary file 2 — Supplementary Information 2. [file 41598_2024_66928_MOESM2_ESM.pdf]

one balun_60 ohms Data File

Frequency (	DB[S11]
0	-0.01042
0.01	-5.49E-11
0.02	-8.78E-10
0.03	-4.45E-09
0.04	-1.41E-08
0.05	-3.43E-08
0.06	-7.11E-08
0.07	-1.32E-07
0.08	-2.25E-07
0.09	-3.60E-07
0.1	-5.49E-07
0.11	-8.04E-07
0.12	-1.14E-06
0.13	-1.57E-06
0.14	-2.11E-06
0.15	-2.78E-06
0.16	-3.60E-06
0.17	-4.59E-06
0.18	-5.77E-06
0.19	-7.16E-06
0.2	-8.79E-06
0.21	-1.07E-05
0.22	-1.29E-05
0.23	-1.54E-05
0.24	-1.82E-05
0.25	-2.15E-05
0.26	-2.51E-05
0.27	-2.92E-05
0.28	-3.38E-05
0.29	-3.89E-05
0.3	-4.45E-05
0.31	-5.08E-05
0.32	-5.77E-05
0.33	-6.52E-05
0.34	-7.35E-05
0.35	-8.25E-05
0.36	-9.24E-05
0.37	-0.0001
0.38	-0.00011
0.39	-0.00013
0.4	-0.00014
0.41	-0.00016
0.42	-0.00017
0.43	-0.00019
0.44	-0.00021
0.45	-0.00023

0.46	-0.00025
0.47	-0.00027
0.48	-0.00029
0.49	-0.00032
0.5	-0.00034
0.51	-0.00037
0.52	-0.0004
0.53	-0.00043
0.54	-0.00047
0.55	-0.0005
0.56	-0.00054
0.57	-0.00058
0.58	-0.00062
0.59	-0.00067
0.6	-0.00071
0.61	-0.00076
0.62	-0.00081
0.63	-0.00086
0.64	-0.00092
0.65	-0.00098
0.66	-0.00104
0.67	-0.0011
0.68	-0.00117
0.69	-0.00124
0.7	-0.00131
0.71	-0.00139
0.72	-0.00147
0.73	-0.00155
0.74	-0.00164
0.75	-0.00172
0.76	-0.00182
0.77	-0.00191
0.78	-0.00201
0.79	-0.00212
0.8	-0.00222
0.81	-0.00233
0.82	-0.00245
0.83	-0.00257
0.84	-0.00269
0.85	-0.00282
0.86	-0.00295
0.87	-0.00308
0.88	-0.00322
0.89	-0.00336
0.9	-0.00351
0.91	-0.00366
0.92	-0.00382
0.93	-0.00398

0.94	-0.00415
0.95	-0.00432
0.96	-0.00449
0.97	-0.00467
0.98	-0.00486
0.99	-0.00504
1	-0.00524
1.01	-0.00544
1.02	-0.00564
1.03	-0.00584
1.04	-0.00606
1.05	-0.00627
1.06	-0.00649
1.07	-0.00672
1.08	-0.00694
1.09	-0.00718
1.1	-0.00741
1.11	-0.00765
1.12	-0.0079
1.13	-0.00815
1.14	-0.0084
1.15	-0.00866
1.16	-0.00891
1.17	-0.00918
1.18	-0.00944
1.19	-0.00971
1.2	-0.00998
1.21	-0.01025
1.22	-0.01052
1.23	-0.0108
1.24	-0.01108
1.25	-0.01135
1.26	-0.01163
1.27	-0.01191
1.28	-0.01219
1.29	-0.01247
1.3	-0.01275
1.31	-0.01302
1.32	-0.01329
1.33	-0.01357
1.34	-0.01383
1.35	-0.0141
1.36	-0.01436
1.37	-0.01461
1.38	-0.01486
1.39	-0.01511
1.4	-0.01534
1.41	-0.01557

1.42	-0.0158
1.43	-0.01601
1.44	-0.01621
1.45	-0.0164
1.46	-0.01658
1.47	-0.01675
1.48	-0.01691
1.49	-0.01705
1.5	-0.01718
1.51	-0.01729
1.52	-0.01738
1.53	-0.01746
1.54	-0.01752
1.55	-0.01755
1.56	-0.01757
1.57	-0.01757
1.58	-0.01755
1.59	-0.0175
1.6	-0.01743
1.61	-0.01733
1.62	-0.01721
1.63	-0.01706
1.64	-0.01689
1.65	-0.01669
1.66	-0.01646
1.67	-0.01621
1.68	-0.01592
1.69	-0.01561
1.7	-0.01527
1.71	-0.0149
1.72	-0.0145
1.73	-0.01407
1.74	-0.01362
1.75	-0.01313
1.76	-0.01263
1.77	-0.01209
1.78	-0.01154
1.79	-0.01096
1.8	-0.01036
1.81	-0.00974
1.82	-0.00911
1.83	-0.00846
1.84	-0.0078
1.85	-0.00714
1.86	-0.00647
1.87	-0.00581
1.88	-0.00515
1.89	-0.0045

1.9	-0.00387
1.91	-0.00326
1.92	-0.00268
1.93	-0.00213
1.94	-0.00163
1.95	-0.00118
1.96	-0.00079
1.97	-0.00046
1.98	-0.00022
1.99	-5.83E-05
2	-1.07E-07
2.01	-5.25E-05
2.02	-0.00023
2.03	-0.00054
2.04	-0.001
2.05	-0.00162
2.06	-0.00242
2.07	-0.00341
2.08	-0.00461
2.09	-0.00604
2.1	-0.00771
2.11	-0.00964
2.12	-0.01185
2.13	-0.01435
2.14	-0.01718
2.15	-0.02035
2.16	-0.02387
2.17	-0.02778
2.18	-0.03209
2.19	-0.03683
2.2	-0.04202
2.21	-0.04769
2.22	-0.05386
2.23	-0.06056
2.24	-0.06781
2.25	-0.07564
2.26	-0.08407
2.27	-0.09314
2.28	-0.10288
2.29	-0.1133
2.3	-0.12445
2.31	-0.13634
2.32	-0.14902
2.33	-0.16251
2.34	-0.17685
2.35	-0.19206
2.36	-0.20818
2.37	-0.22524

2.38	-0.24327
2.39	-0.2623
2.4	-0.28238
2.41	-0.30354
2.42	-0.3258
2.43	-0.3492
2.44	-0.37378
2.45	-0.39958
2.46	-0.42663
2.47	-0.45496
2.48	-0.48461
2.49	-0.51562
2.5	-0.54802
2.51	-0.58184
2.52	-0.61714
2.53	-0.65393
2.54	-0.69226
2.55	-0.73217
2.56	-0.77368
2.57	-0.81683
2.58	-0.86167
2.59	-0.90822
2.6	-0.95651
2.61	-1.00659
2.62	-1.05849
2.63	-1.11223
2.64	-1.16786
2.65	-1.2254
2.66	-1.28488
2.67	-1.34633
2.68	-1.40978
2.69	-1.47526
2.7	-1.5428
2.71	-1.61241
2.72	-1.68413
2.73	-1.75797
2.74	-1.83396
2.75	-1.91211
2.76	-1.99244
2.77	-2.07497
2.78	-2.1597
2.79	-2.24666
2.8	-2.33585
2.81	-2.42727
2.82	-2.52093
2.83	-2.61684
2.84	-2.71498
2.85	-2.81537

2.86	-2.91799
2.87	-3.02283
2.88	-3.12988
2.89	-3.23913
2.9	-3.35055
2.91	-3.46413
2.92	-3.57985
2.93	-3.69766
2.94	-3.81755
2.95	-3.93947
2.96	-4.06339
2.97	-4.18925
2.98	-4.31703
2.99	-4.44665
3	-4.57807
3.01	-4.71122
3.02	-4.84604
3.03	-4.98246
3.04	-5.12039
3.05	-5.25977
3.06	-5.40051
3.07	-5.54251
3.08	-5.68567
3.09	-5.82989
3.1	-5.97507
3.11	-6.12109
3.12	-6.26783
3.13	-6.41515
3.14	-6.56293
3.15	-6.71102
3.16	-6.85928
3.17	-7.00755
3.18	-7.15567
3.19	-7.30348
3.2	-7.4508
3.21	-7.59745
3.22	-7.74325
3.23	-7.888
3.24	-8.03151
3.25	-8.17358
3.26	-8.314
3.27	-8.45257
3.28	-8.58907
3.29	-8.72329
3.3	-8.85502
3.31	-8.98406
3.32	-9.11017
3.33	-9.23317

3.34	-9.35285
3.35	-9.46901
3.36	-9.58146
3.37	-9.69003
3.38	-9.79454
3.39	-9.89483
3.4	-9.99076
3.41	-10.0822
3.42	-10.169
3.43	-10.2512
3.44	-10.3286
3.45	-10.4011
3.46	-10.4687
3.47	-10.5314
3.48	-10.5893
3.49	-10.6422
3.5	-10.6902
3.51	-10.7335
3.52	-10.772
3.53	-10.8059
3.54	-10.8352
3.55	-10.8601
3.56	-10.8806
3.57	-10.897
3.58	-10.9094
3.59	-10.9179
3.6	-10.9227
3.61	-10.9239
3.62	-10.9218
3.63	-10.9165
3.64	-10.9082
3.65	-10.897
3.66	-10.8832
3.67	-10.8669
3.68	-10.8483
3.69	-10.8275
3.7	-10.8047
3.71	-10.7801
3.72	-10.7538
3.73	-10.726
3.74	-10.6968
3.75	-10.6663
3.76	-10.6347
3.77	-10.6021
3.78	-10.5686
3.79	-10.5343
3.8	-10.4993
3.81	-10.4638

3.82	-10.4277
3.83	-10.3913
3.84	-10.3545
3.85	-10.3175
3.86	-10.2803
3.87	-10.243
3.88	-10.2057
3.89	-10.1684
3.9	-10.1311
3.91	-10.094
3.92	-10.057
3.93	-10.0202
3.94	-9.98364
3.95	-9.94735
3.96	-9.91137
3.97	-9.87572
3.98	-9.84043
3.99	-9.80552
4	-9.77102
4.01	-9.73694
4.02	-9.70331
4.03	-9.67013
4.04	-9.63743
4.05	-9.60521
4.06	-9.57349
4.07	-9.54228
4.08	-9.51158
4.09	-9.48141
4.1	-9.45176
4.11	-9.42265
4.12	-9.39408
4.13	-9.36605
4.14	-9.33857
4.15	-9.31164
4.16	-9.28525
4.17	-9.25942
4.18	-9.23413
4.19	-9.2094
4.2	-9.18521
4.21	-9.16158
4.22	-9.13849
4.23	-9.11594
4.24	-9.09394
4.25	-9.07248
4.26	-9.05156
4.27	-9.03117
4.28	-9.01131
4.29	-8.99198

4.3	-8.97318
4.31	-8.9549
4.32	-8.93713
4.33	-8.91988
4.34	-8.90314
4.35	-8.88691
4.36	-8.87117
4.37	-8.85593
4.38	-8.84119
4.39	-8.82693
4.4	-8.81316
4.41	-8.79987
4.42	-8.78706
4.43	-8.77471
4.44	-8.76284
4.45	-8.75142
4.46	-8.74047
4.47	-8.72996
4.48	-8.71991
4.49	-8.71031
4.5	-8.70114
4.51	-8.69241
4.52	-8.68411
4.53	-8.67625
4.54	-8.6688
4.55	-8.66178
4.56	-8.65517
4.57	-8.64898
4.58	-8.64319
4.59	-8.63781
4.6	-8.63283
4.61	-8.62824
4.62	-8.62405
4.63	-8.62025
4.64	-8.61683
4.65	-8.6138
4.66	-8.61114
4.67	-8.60886
4.68	-8.60695
4.69	-8.6054
4.7	-8.60422
4.71	-8.60341
4.72	-8.60295
4.73	-8.60284
4.74	-8.60309
4.75	-8.60368
4.76	-8.60462
4.77	-8.60591

4.78	-8.60753
4.79	-8.60948
4.8	-8.61178
4.81	-8.6144
4.82	-8.61735
4.83	-8.62062
4.84	-8.62422
4.85	-8.62813
4.86	-8.63236
4.87	-8.63691
4.88	-8.64177
4.89	-8.64693
4.9	-8.65241
4.91	-8.65818
4.92	-8.66426
4.93	-8.67064
4.94	-8.67731
4.95	-8.68428
4.96	-8.69154
4.97	-8.69909
4.98	-8.70692
4.99	-8.71505
5	-8.72345
5.01	-8.73214
5.02	-8.7411
5.03	-8.75034
5.04	-8.75985
5.05	-8.76964
5.06	-8.7797
5.07	-8.79002
5.08	-8.80061
5.09	-8.81146
5.1	-8.82258
5.11	-8.83396
5.12	-8.84559
5.13	-8.85748
5.14	-8.86962
5.15	-8.88202
5.16	-8.89466
5.17	-8.90755
5.18	-8.92069
5.19	-8.93408
5.2	-8.9477
5.21	-8.96157
5.22	-8.97567
5.23	-8.99001
5.24	-9.00458
5.25	-9.01939

5.26	-9.03443
5.27	-9.0497
5.28	-9.06519
5.29	-9.08091
5.3	-9.09685
5.31	-9.11301
5.32	-9.1294
5.33	-9.14599
5.34	-9.16281
5.35	-9.17984
5.36	-9.19707
5.37	-9.21452
5.38	-9.23218
5.39	-9.25004
5.4	-9.2681
5.41	-9.28636
5.42	-9.30482
5.43	-9.32348
5.44	-9.34234
5.45	-9.36138
5.46	-9.38062
5.47	-9.40004
5.48	-9.41965
5.49	-9.43944
5.5	-9.45942
5.51	-9.47957
5.52	-9.4999
5.53	-9.5204
5.54	-9.54108
5.55	-9.56192
5.56	-9.58293
5.57	-9.60411
5.58	-9.62544
5.59	-9.64694
5.6	-9.66859
5.61	-9.69039
5.62	-9.71234
5.63	-9.73444
5.64	-9.75669
5.65	-9.77908
5.66	-9.80161
5.67	-9.82427
5.68	-9.84706
5.69	-9.86999
5.7	-9.89304
5.71	-9.91622
5.72	-9.93951
5.73	-9.96292

5.74	-9.98645
5.75	-10.0101
5.76	-10.0338
5.77	-10.0577
5.78	-10.0816
5.79	-10.1056
5.8	-10.1298
5.81	-10.154
5.82	-10.1783
5.83	-10.2026
5.84	-10.2271
5.85	-10.2516
5.86	-10.2762
5.87	-10.3008
5.88	-10.3256
5.89	-10.3503
5.9	-10.3751
5.91	-10.4
5.92	-10.4249
5.93	-10.4498
5.94	-10.4747
5.95	-10.4997
5.96	-10.5247
5.97	-10.5497
5.98	-10.5747
5.99	-10.5997
6	-10.6248
6.01	-10.6498
6.02	-10.6748
6.03	-10.6997
6.04	-10.7247
6.05	-10.7496
6.06	-10.7745
6.07	-10.7993
6.08	-10.8241
6.09	-10.8488
6.1	-10.8735
6.11	-10.8981
6.12	-10.9226
6.13	-10.9471
6.14	-10.9714
6.15	-10.9957
6.16	-11.0198
6.17	-11.0439
6.18	-11.0678
6.19	-11.0916
6.2	-11.1153
6.21	-11.1389

6.22	-11.1623
6.23	-11.1855
6.24	-11.2086
6.25	-11.2315
6.26	-11.2543
6.27	-11.2768
6.28	-11.2992
6.29	-11.3214
6.3	-11.3433
6.31	-11.3651
6.32	-11.3866
6.33	-11.408
6.34	-11.429
6.35	-11.4499
6.36	-11.4704
6.37	-11.4908
6.38	-11.5108
6.39	-11.5306
6.4	-11.5501
6.41	-11.5693
6.42	-11.5882
6.43	-11.6068
6.44	-11.6251
6.45	-11.6431
6.46	-11.6607
6.47	-11.678
6.48	-11.695
6.49	-11.7116
6.5	-11.7279
6.51	-11.7437
6.52	-11.7593
6.53	-11.7744
6.54	-11.7891
6.55	-11.8035
6.56	-11.8174
6.57	-11.831
6.58	-11.8441
6.59	-11.8568
6.6	-11.8691
6.61	-11.881
6.62	-11.8924
6.63	-11.9034
6.64	-11.9139
6.65	-11.924
6.66	-11.9336
6.67	-11.9428
6.68	-11.9515
6.69	-11.9597

6.7	-11.9674
6.71	-11.9747
6.72	-11.9815
6.73	-11.9878
6.74	-11.9936
6.75	-11.9989
6.76	-12.0037
6.77	-12.008
6.78	-12.0118
6.79	-12.0151
6.8	-12.0179
6.81	-12.0202
6.82	-12.022
6.83	-12.0232
6.84	-12.024
6.85	-12.0243
6.86	-12.024
6.87	-12.0232
6.88	-12.022
6.89	-12.0202
6.9	-12.0179
6.91	-12.0151
6.92	-12.0118
6.93	-12.008
6.94	-12.0037
6.95	-11.9989
6.96	-11.9936
6.97	-11.9878
6.98	-11.9815
6.99	-11.9747
7	-11.9674
7.01	-11.9597
7.02	-11.9515
7.03	-11.9428
7.04	-11.9336
7.05	-11.924
7.06	-11.9139
7.07	-11.9034
7.08	-11.8924
7.09	-11.881
7.1	-11.8691
7.11	-11.8568
7.12	-11.8441
7.13	-11.831
7.14	-11.8174
7.15	-11.8035
7.16	-11.7891
7.17	-11.7744

7.18	-11.7593
7.19	-11.7437
7.2	-11.7279
7.21	-11.7116
7.22	-11.695
7.23	-11.678
7.24	-11.6607
7.25	-11.6431
7.26	-11.6251
7.27	-11.6068
7.28	-11.5882
7.29	-11.5693
7.3	-11.5501
7.31	-11.5306
7.32	-11.5108
7.33	-11.4908
7.34	-11.4704
7.35	-11.4499
7.36	-11.429
7.37	-11.408
7.38	-11.3866
7.39	-11.3651
7.4	-11.3433
7.41	-11.3214
7.42	-11.2992
7.43	-11.2768
7.44	-11.2543
7.45	-11.2315
7.46	-11.2086
7.47	-11.1855
7.48	-11.1623
7.49	-11.1389
7.5	-11.1153
7.51	-11.0916
7.52	-11.0678
7.53	-11.0439
7.54	-11.0198
7.55	-10.9957
7.56	-10.9714
7.57	-10.9471
7.58	-10.9226
7.59	-10.8981
7.6	-10.8735
7.61	-10.8488
7.62	-10.8241
7.63	-10.7993
7.64	-10.7745
7.65	-10.7496

7.66	-10.7247
7.67	-10.6997
7.68	-10.6748
7.69	-10.6498
7.7	-10.6248
7.71	-10.5997
7.72	-10.5747
7.73	-10.5497
7.74	-10.5247
7.75	-10.4997
7.76	-10.4747
7.77	-10.4498
7.78	-10.4249
7.79	-10.4
7.8	-10.3751
7.81	-10.3503
7.82	-10.3256
7.83	-10.3008
7.84	-10.2762
7.85	-10.2516
7.86	-10.2271
7.87	-10.2026
7.88	-10.1783
7.89	-10.154
7.9	-10.1298
7.91	-10.1056
7.92	-10.0816
7.93	-10.0577
7.94	-10.0338
7.95	-10.0101
7.96	-9.98645
7.97	-9.96292
7.98	-9.93951
7.99	-9.91622
8	-9.89304
8.01	-9.86999
8.02	-9.84706
8.03	-9.82427
8.04	-9.80161
8.05	-9.77908
8.06	-9.75669
8.07	-9.73444
8.08	-9.71234
8.09	-9.69039
8.1	-9.66859
8.11	-9.64694
8.12	-9.62544
8.13	-9.60411

8.14	-9.58293
8.15	-9.56192
8.16	-9.54108
8.17	-9.5204
8.18	-9.4999
8.19	-9.47957
8.2	-9.45942
8.21	-9.43944
8.22	-9.41965
8.23	-9.40004
8.24	-9.38062
8.25	-9.36138
8.26	-9.34234
8.27	-9.32348
8.28	-9.30482
8.29	-9.28636
8.3	-9.2681
8.31	-9.25004
8.32	-9.23218
8.33	-9.21452
8.34	-9.19707
8.35	-9.17984
8.36	-9.16281
8.37	-9.14599
8.38	-9.1294
8.39	-9.11301
8.4	-9.09685
8.41	-9.08091
8.42	-9.06519
8.43	-9.0497
8.44	-9.03443
8.45	-9.01939
8.46	-9.00458
8.47	-8.99001
8.48	-8.97567
8.49	-8.96157
8.5	-8.9477
8.51	-8.93408
8.52	-8.92069
8.53	-8.90755
8.54	-8.89466
8.55	-8.88202
8.56	-8.86962
8.57	-8.85748
8.58	-8.84559
8.59	-8.83396
8.6	-8.82258
8.61	-8.81146

8.62	-8.80061
8.63	-8.79002
8.64	-8.7797
8.65	-8.76964
8.66	-8.75985
8.67	-8.75034
8.68	-8.7411
8.69	-8.73214
8.7	-8.72345
8.71	-8.71505
8.72	-8.70692
8.73	-8.69909
8.74	-8.69154
8.75	-8.68428
8.76	-8.67731
8.77	-8.67064
8.78	-8.66426
8.79	-8.65818
8.8	-8.65241
8.81	-8.64693
8.82	-8.64177
8.83	-8.63691
8.84	-8.63236
8.85	-8.62813
8.86	-8.62422
8.87	-8.62062
8.88	-8.61735
8.89	-8.6144
8.9	-8.61178
8.91	-8.60948
8.92	-8.60753
8.93	-8.60591
8.94	-8.60462
8.95	-8.60368
8.96	-8.60309
8.97	-8.60284
8.98	-8.60295
8.99	-8.60341
9	-8.60422
9.01	-8.6054
9.02	-8.60695
9.03	-8.60886
9.04	-8.61114
9.05	-8.6138
9.06	-8.61683
9.07	-8.62025
9.08	-8.62405
9.09	-8.62824

9.1	-8.63283
9.11	-8.63781
9.12	-8.64319
9.13	-8.64898
9.14	-8.65517
9.15	-8.66178
9.16	-8.6688
9.17	-8.67625
9.18	-8.68411
9.19	-8.69241
9.2	-8.70114
9.21	-8.71031
9.22	-8.71991
9.23	-8.72996
9.24	-8.74047
9.25	-8.75142
9.26	-8.76284
9.27	-8.77471
9.28	-8.78706
9.29	-8.79987
9.3	-8.81316
9.31	-8.82693
9.32	-8.84119
9.33	-8.85593
9.34	-8.87117
9.35	-8.88691
9.36	-8.90314
9.37	-8.91988
9.38	-8.93713
9.39	-8.9549
9.4	-8.97318
9.41	-8.99198
9.42	-9.01131
9.43	-9.03117
9.44	-9.05156
9.45	-9.07248
9.46	-9.09394
9.47	-9.11594
9.48	-9.13849
9.49	-9.16158
9.5	-9.18521
9.51	-9.2094
9.52	-9.23413
9.53	-9.25942
9.54	-9.28525
9.55	-9.31164
9.56	-9.33857
9.57	-9.36605

9.58	-9.39408
9.59	-9.42265
9.6	-9.45176
9.61	-9.48141
9.62	-9.51158
9.63	-9.54228
9.64	-9.57349
9.65	-9.60521
9.66	-9.63743
9.67	-9.67013
9.68	-9.70331
9.69	-9.73694
9.7	-9.77102
9.71	-9.80552
9.72	-9.84043
9.73	-9.87572
9.74	-9.91137
9.75	-9.94735
9.76	-9.98364
9.77	-10.0202
9.78	-10.057
9.79	-10.094
9.8	-10.1311
9.81	-10.1684
9.82	-10.2057
9.83	-10.243
9.84	-10.2803
9.85	-10.3175
9.86	-10.3545
9.87	-10.3913
9.88	-10.4277
9.89	-10.4638
9.9	-10.4993
9.91	-10.5343
9.92	-10.5686
9.93	-10.6021
9.94	-10.6347
9.95	-10.6663
9.96	-10.6968
9.97	-10.726
9.98	-10.7538
9.99	-10.7801
10	-10.8047
10.01	-10.8275
10.02	-10.8483
10.03	-10.8669
10.04	-10.8832
10.05	-10.897

10.06	-10.9082
10.07	-10.9165
10.08	-10.9218
10.09	-10.9239
10.1	-10.9227
10.11	-10.9179
10.12	-10.9094
10.13	-10.897
10.14	-10.8806
10.15	-10.8601
10.16	-10.8352
10.17	-10.8059
10.18	-10.772
10.19	-10.7335
10.2	-10.6902
10.21	-10.6422
10.22	-10.5893
10.23	-10.5314
10.24	-10.4687
10.25	-10.4011
10.26	-10.3286
10.27	-10.2512
10.28	-10.169
10.29	-10.0822
10.3	-9.99076
10.31	-9.89483
10.32	-9.79454
10.33	-9.69003
10.34	-9.58146
10.35	-9.46901
10.36	-9.35285
10.37	-9.23317
10.38	-9.11017
10.39	-8.98406
10.4	-8.85502
10.41	-8.72329
10.42	-8.58907
10.43	-8.45257
10.44	-8.314
10.45	-8.17358
10.46	-8.03151
10.47	-7.888
10.48	-7.74325
10.49	-7.59745
10.5	-7.4508
10.51	-7.30348
10.52	-7.15567
10.53	-7.00755

10.54	-6.85928
10.55	-6.71102
10.56	-6.56293
10.57	-6.41515
10.58	-6.26783
10.59	-6.12109
10.6	-5.97507
10.61	-5.82989
10.62	-5.68567
10.63	-5.54251
10.64	-5.40051
10.65	-5.25977
10.66	-5.12039
10.67	-4.98246
10.68	-4.84604
10.69	-4.71122
10.7	-4.57807
10.71	-4.44665
10.72	-4.31703
10.73	-4.18925
10.74	-4.06339
10.75	-3.93947
10.76	-3.81755
10.77	-3.69766
10.78	-3.57985
10.79	-3.46413
10.8	-3.35055
10.81	-3.23913
10.82	-3.12988
10.83	-3.02283
10.84	-2.91799
10.85	-2.81537
10.86	-2.71498
10.87	-2.61684
10.88	-2.52093
10.89	-2.42727
10.9	-2.33585
10.91	-2.24666
10.92	-2.1597
10.93	-2.07497
10.94	-1.99244
10.95	-1.91211
10.96	-1.83396
10.97	-1.75797
10.98	-1.68413
10.99	-1.61241
11	-1.5428
11.01	-1.47526

11.02	-1.40978
11.03	-1.34633
11.04	-1.28488
11.05	-1.2254
11.06	-1.16786
11.07	-1.11223
11.08	-1.05849
11.09	-1.00659
11.1	-0.95651
11.11	-0.90822
11.12	-0.86167
11.13	-0.81683
11.14	-0.77368
11.15	-0.73217
11.16	-0.69226
11.17	-0.65393
11.18	-0.61714
11.19	-0.58184
11.2	-0.54802
11.21	-0.51562
11.22	-0.48461
11.23	-0.45496
11.24	-0.42663
11.25	-0.39958
11.26	-0.37378
11.27	-0.3492
11.28	-0.3258
11.29	-0.30354
11.3	-0.28238
11.31	-0.2623
11.32	-0.24327
11.33	-0.22524
11.34	-0.20818
11.35	-0.19206
11.36	-0.17685
11.37	-0.16251
11.38	-0.14902
11.39	-0.13634
11.4	-0.12445
11.41	-0.1133
11.42	-0.10288
11.43	-0.09314
11.44	-0.08407
11.45	-0.07564
11.46	-0.06781
11.47	-0.06056
11.48	-0.05386
11.49	-0.04769

11.5	-0.04202
11.51	-0.03683
11.52	-0.03209
11.53	-0.02778
11.54	-0.02387
11.55	-0.02035
11.56	-0.01718
11.57	-0.01435
11.58	-0.01185
11.59	-0.00964
11.6	-0.00771
11.61	-0.00604
11.62	-0.00461
11.63	-0.00341
11.64	-0.00242
11.65	-0.00162
11.66	-0.001
11.67	-0.00054
11.68	-0.00023
11.69	-5.25E-05
11.7	-1.07E-07
11.71	-5.83E-05
11.72	-0.00022
11.73	-0.00046
11.74	-0.00079
11.75	-0.00118
11.76	-0.00163
11.77	-0.00213
11.78	-0.00268
11.79	-0.00326
11.8	-0.00387
11.81	-0.0045
11.82	-0.00515
11.83	-0.00581
11.84	-0.00647
11.85	-0.00714
11.86	-0.0078
11.87	-0.00846
11.88	-0.00911
11.89	-0.00974
11.9	-0.01036
11.91	-0.01096
11.92	-0.01154
11.93	-0.01209
11.94	-0.01263
11.95	-0.01313
11.96	-0.01362
11.97	-0.01407

11.98	-0.0145
11.99	-0.0149
12	-0.01527
12.01	-0.01561
12.02	-0.01592
12.03	-0.01621
12.04	-0.01646
12.05	-0.01669
12.06	-0.01689
12.07	-0.01706
12.08	-0.01721
12.09	-0.01733
12.1	-0.01743
12.11	-0.0175
12.12	-0.01755
12.13	-0.01757
12.14	-0.01757
12.15	-0.01755
12.16	-0.01752
12.17	-0.01746
12.18	-0.01738
12.19	-0.01729
12.2	-0.01718
12.21	-0.01705
12.22	-0.01691
12.23	-0.01675
12.24	-0.01658
12.25	-0.0164
12.26	-0.01621
12.27	-0.01601
12.28	-0.0158
12.29	-0.01557
12.3	-0.01534
12.31	-0.01511
12.32	-0.01486
12.33	-0.01461
12.34	-0.01436
12.35	-0.0141
12.36	-0.01383
12.37	-0.01357
12.38	-0.01329
12.39	-0.01302
12.4	-0.01275
12.41	-0.01247
12.42	-0.01219
12.43	-0.01191
12.44	-0.01163
12.45	-0.01135

12.46	-0.01108
12.47	-0.0108
12.48	-0.01052
12.49	-0.01025
12.5	-0.00998
12.51	-0.00971
12.52	-0.00944
12.53	-0.00918
12.54	-0.00891
12.55	-0.00866
12.56	-0.0084
12.57	-0.00815
12.58	-0.0079
12.59	-0.00765
12.6	-0.00741
12.61	-0.00718
12.62	-0.00694
12.63	-0.00672
12.64	-0.00649
12.65	-0.00627
12.66	-0.00606
12.67	-0.00584
12.68	-0.00564
12.69	-0.00544
12.7	-0.00524
12.71	-0.00504
12.72	-0.00486
12.73	-0.00467
12.74	-0.00449
12.75	-0.00432
12.76	-0.00415
12.77	-0.00398
12.78	-0.00382
12.79	-0.00366
12.8	-0.00351
12.81	-0.00336
12.82	-0.00322
12.83	-0.00308
12.84	-0.00295
12.85	-0.00282
12.86	-0.00269
12.87	-0.00257
12.88	-0.00245
12.89	-0.00233
12.9	-0.00222
12.91	-0.00212
12.92	-0.00201
12.93	-0.00191

12.94	-0.00182
12.95	-0.00172
12.96	-0.00164
12.97	-0.00155
12.98	-0.00147
12.99	-0.00139
13	-0.00131
13.01	-0.00124
13.02	-0.00117
13.03	-0.0011
13.04	-0.00104
13.05	-0.00098
13.06	-0.00092
13.07	-0.00086
13.08	-0.00081
13.09	-0.00076
13.1	-0.00071
13.11	-0.00067
13.12	-0.00062
13.13	-0.00058
13.14	-0.00054
13.15	-0.0005
13.16	-0.00047
13.17	-0.00043
13.18	-0.0004
13.19	-0.00037
13.2	-0.00034
13.21	-0.00032
13.22	-0.00029
13.23	-0.00027
13.24	-0.00025
13.25	-0.00023
13.26	-0.00021
13.27	-0.00019
13.28	-0.00017
13.29	-0.00016
13.3	-0.00014
13.31	-0.00013
13.32	-0.00011
13.33	-0.0001
13.34	-9.24E-05
13.35	-8.25E-05
13.36	-7.35E-05
13.37	-6.52E-05
13.38	-5.77E-05
13.39	-5.08E-05
13.4	-4.45E-05
13.41	-3.89E-05

13.42	-3.38E-05
13.43	-2.92E-05
13.44	-2.51E-05
13.45	-2.15E-05
13.46	-1.82E-05
13.47	-1.54E-05
13.48	-1.29E-05
13.49	-1.07E-05
13.5	-8.79E-06
13.51	-7.16E-06
13.52	-5.77E-06
13.53	-4.59E-06
13.54	-3.60E-06
13.55	-2.78E-06
13.56	-2.11E-06
13.57	-1.57E-06
13.58	-1.14E-06
13.59	-8.04E-07
13.6	-5.49E-07
13.61	-3.60E-07
13.62	-2.25E-07
13.63	-1.32E-07
13.64	-7.11E-08
13.65	-3.43E-08
13.66	-1.41E-08
13.67	-4.45E-09
13.68	-8.78E-10
13.69	-5.49E-11
13.7	0
13.71	-5.49E-11
13.72	-8.78E-10
13.73	-4.45E-09
13.74	-1.41E-08
13.75	-3.43E-08
13.76	-7.11E-08
13.77	-1.32E-07
13.78	-2.25E-07
13.79	-3.60E-07
13.8	-5.49E-07
13.81	-8.04E-07
13.82	-1.14E-06
13.83	-1.57E-06
13.84	-2.11E-06
13.85	-2.78E-06
13.86	-3.60E-06
13.87	-4.59E-06
13.88	-5.77E-06
13.89	-7.16E-06

13.9	-8.79E-06
13.91	-1.07E-05
13.92	-1.29E-05
13.93	-1.54E-05
13.94	-1.82E-05
13.95	-2.15E-05
13.96	-2.51E-05
13.97	-2.92E-05
13.98	-3.38E-05
13.99	-3.89E-05
14	-4.45E-05
14.01	-5.08E-05
14.02	-5.77E-05
14.03	-6.52E-05
14.04	-7.35E-05
14.05	-8.25E-05
14.06	-9.24E-05
14.07	-0.0001
14.08	-0.00011
14.09	-0.00013
14.1	-0.00014
14.11	-0.00016
14.12	-0.00017
14.13	-0.00019
14.14	-0.00021
14.15	-0.00023
14.16	-0.00025
14.17	-0.00027
14.18	-0.00029
14.19	-0.00032
14.2	-0.00034
14.21	-0.00037
14.22	-0.0004
14.23	-0.00043
14.24	-0.00047
14.25	-0.0005
14.26	-0.00054
14.27	-0.00058
14.28	-0.00062
14.29	-0.00067
14.3	-0.00071
14.31	-0.00076
14.32	-0.00081
14.33	-0.00086
14.34	-0.00092
14.35	-0.00098
14.36	-0.00104
14.37	-0.0011

14.38	-0.00117
14.39	-0.00124
14.4	-0.00131
14.41	-0.00139
14.42	-0.00147
14.43	-0.00155
14.44	-0.00164
14.45	-0.00172
14.46	-0.00182
14.47	-0.00191
14.48	-0.00201
14.49	-0.00212
14.5	-0.00222
14.51	-0.00233
14.52	-0.00245
14.53	-0.00257
14.54	-0.00269
14.55	-0.00282
14.56	-0.00295
14.57	-0.00308
14.58	-0.00322
14.59	-0.00336
14.6	-0.00351
14.61	-0.00366
14.62	-0.00382
14.63	-0.00398
14.64	-0.00415
14.65	-0.00432
14.66	-0.00449
14.67	-0.00467
14.68	-0.00486
14.69	-0.00504
14.7	-0.00524
14.71	-0.00544
14.72	-0.00564
14.73	-0.00584
14.74	-0.00606
14.75	-0.00627
14.76	-0.00649
14.77	-0.00672
14.78	-0.00694
14.79	-0.00718
14.8	-0.00741
14.81	-0.00765
14.82	-0.0079
14.83	-0.00815
14.84	-0.0084
14.85	-0.00866

14.86	-0.00891
14.87	-0.00918
14.88	-0.00944
14.89	-0.00971
14.9	-0.00998
14.91	-0.01025
14.92	-0.01052
14.93	-0.0108
14.94	-0.01108
14.95	-0.01135
14.96	-0.01163
14.97	-0.01191
14.98	-0.01219
14.99	-0.01247
15	-0.01275
15.01	-0.01302
15.02	-0.01329
15.03	-0.01357
15.04	-0.01383
15.05	-0.0141
15.06	-0.01436
15.07	-0.01461
15.08	-0.01486
15.09	-0.01511
15.1	-0.01534
15.11	-0.01557
15.12	-0.0158
15.13	-0.01601
15.14	-0.01621
15.15	-0.0164
15.16	-0.01658
15.17	-0.01675
15.18	-0.01691
15.19	-0.01705
15.2	-0.01718
15.21	-0.01729
15.22	-0.01738
15.23	-0.01746
15.24	-0.01752
15.25	-0.01755
15.26	-0.01757
15.27	-0.01757
15.28	-0.01755
15.29	-0.0175
15.3	-0.01743
15.31	-0.01733
15.32	-0.01721
15.33	-0.01706

15.34	-0.01689
15.35	-0.01669
15.36	-0.01646
15.37	-0.01621
15.38	-0.01592
15.39	-0.01561
15.4	-0.01527
15.41	-0.0149
15.42	-0.0145
15.43	-0.01407
15.44	-0.01362
15.45	-0.01313
15.46	-0.01263
15.47	-0.01209
15.48	-0.01154
15.49	-0.01096
15.5	-0.01036
15.51	-0.00974
15.52	-0.00911
15.53	-0.00846
15.54	-0.0078
15.55	-0.00714
15.56	-0.00647
15.57	-0.00581
15.58	-0.00515
15.59	-0.0045
15.6	-0.00387
15.61	-0.00326
15.62	-0.00268
15.63	-0.00213
15.64	-0.00163
15.65	-0.00118
15.66	-0.00079
15.67	-0.00046
15.68	-0.00022
15.69	-5.83E-05
15.7	-1.07E-07
15.71	-5.25E-05
15.72	-0.00023
15.73	-0.00054
15.74	-0.001
15.75	-0.00162
15.76	-0.00242
15.77	-0.00341
15.78	-0.00461
15.79	-0.00604
15.8	-0.00771
15.81	-0.00964

15.82	-0.01185
15.83	-0.01435
15.84	-0.01718
15.85	-0.02035
15.86	-0.02387
15.87	-0.02778
15.88	-0.03209
15.89	-0.03683
15.9	-0.04202
15.91	-0.04769
15.92	-0.05386
15.93	-0.06056
15.94	-0.06781
15.95	-0.07564
15.96	-0.08407
15.97	-0.09314
15.98	-0.10288
15.99	-0.1133
16	-0.12445

one balun_60 ohms Data File

Frequency (	DB[S21]
0.05	-81.0241
0.06	-77.8568
0.07	-75.1788
0.08	-72.859
0.09	-70.8127
0.1	-68.9822
0.11	-67.3263
0.12	-65.8146
0.13	-64.4239
0.14	-63.1363
0.15	-61.9375
0.16	-60.8161
0.17	-59.7626
0.18	-58.7694
0.19	-57.8299
0.2	-56.9385
0.21	-56.0906
0.22	-55.2822
0.23	-54.5097
0.24	-53.77
0.25	-53.0605
0.26	-52.3789
0.27	-51.7229
0.28	-51.0908
0.29	-50.4809
0.3	-49.8916

0.31	-49.3217
0.32	-48.7699
0.33	-48.235
0.34	-47.7162
0.35	-47.2123
0.36	-46.7227
0.37	-46.2465
0.38	-45.783
0.39	-45.3316
0.4	-44.8916
0.41	-44.4626
0.42	-44.0439
0.43	-43.635
0.44	-43.2357
0.45	-42.8453
0.46	-42.4635
0.47	-42.0901
0.48	-41.7245
0.49	-41.3665
0.5	-41.0159
0.51	-40.6722
0.52	-40.3353
0.53	-40.0049
0.54	-39.6807
0.55	-39.3626
0.56	-39.0503
0.57	-38.7436
0.58	-38.4424
0.59	-38.1464
0.6	-37.8555
0.61	-37.5696
0.62	-37.2884
0.63	-37.0118
0.64	-36.7398
0.65	-36.4721
0.66	-36.2087
0.67	-35.9494
0.68	-35.6942
0.69	-35.4428
0.7	-35.1952
0.71	-34.9514
0.72	-34.7112
0.73	-34.4745
0.74	-34.2412
0.75	-34.0114
0.76	-33.7848
0.77	-33.5614
0.78	-33.3412

0.79	-33.1241
0.8	-32.91
0.81	-32.6988
0.82	-32.4906
0.83	-32.2852
0.84	-32.0826
0.85	-31.8828
0.86	-31.6857
0.87	-31.4912
0.88	-31.2994
0.89	-31.1101
0.9	-30.9233
0.91	-30.7391
0.92	-30.5574
0.93	-30.3781
0.94	-30.2012
0.95	-30.0266
0.96	-29.8545
0.97	-29.6847
0.98	-29.5171
0.99	-29.3519
1	-29.189
1.01	-29.0283
1.02	-28.8698
1.03	-28.7135
1.04	-28.5595
1.05	-28.4077
1.06	-28.2581
1.07	-28.1106
1.08	-27.9654
1.09	-27.8223
1.1	-27.6813
1.11	-27.5426
1.12	-27.406
1.13	-27.2716
1.14	-27.1394
1.15	-27.0094
1.16	-26.8815
1.17	-26.7559
1.18	-26.6324
1.19	-26.5112
1.2	-26.3923
1.21	-26.2756
1.22	-26.1612
1.23	-26.049
1.24	-25.9392
1.25	-25.8318
1.26	-25.7267

1.27	-25.624
1.28	-25.5237
1.29	-25.426
1.3	-25.3307
1.31	-25.238
1.32	-25.1478
1.33	-25.0603
1.34	-24.9755
1.35	-24.8934
1.36	-24.8141
1.37	-24.7376
1.38	-24.6641
1.39	-24.5935
1.4	-24.526
1.41	-24.4616
1.42	-24.4004
1.43	-24.3425
1.44	-24.288
1.45	-24.2369
1.46	-24.1894
1.47	-24.1456
1.48	-24.1056
1.49	-24.0695
1.5	-24.0374
1.51	-24.0095
1.52	-23.9859
1.53	-23.9669
1.54	-23.9524
1.55	-23.9428
1.56	-23.9382
1.57	-23.9389
1.58	-23.9449
1.59	-23.9567
1.6	-23.9743
1.61	-23.9981
1.62	-24.0285
1.63	-24.0656
1.64	-24.1098
1.65	-24.1616
1.66	-24.2212
1.67	-24.2892
1.68	-24.3659
1.69	-24.452
1.7	-24.548
1.71	-24.6545
1.72	-24.7722
1.73	-24.9019
1.74	-25.0444

1.75	-25.2006
1.76	-25.3717
1.77	-25.5587
1.78	-25.7632
1.79	-25.9866
1.8	-26.2306
1.81	-26.4973
1.82	-26.7891
1.83	-27.1086
1.84	-27.4593
1.85	-27.8448
1.86	-28.27
1.87	-28.7404
1.88	-29.2633
1.89	-29.8474
1.9	-30.5042
1.91	-31.2489
1.92	-32.1016
1.93	-33.0909
1.94	-34.2583
1.95	-35.6681
1.96	-37.4275
1.97	-39.7364
1.98	-43.0386
1.99	-48.7187
2	-76.0816
2.01	-49.175
2.02	-42.8056
2.03	-39.0689
2.04	-36.3892
2.05	-34.2846
2.06	-32.5425
2.07	-31.0502
2.08	-29.7409
2.09	-28.5714
2.1	-27.5124
2.11	-26.543
2.12	-25.6479
2.13	-24.8153
2.14	-24.0362
2.15	-23.3034
2.16	-22.6111
2.17	-21.9546
2.18	-21.33
2.19	-20.734
2.2	-20.1638
2.21	-19.6171
2.22	-19.0919

2.23	-18.5863
2.24	-18.0989
2.25	-17.6283
2.26	-17.1733
2.27	-16.7329
2.28	-16.3061
2.29	-15.8921
2.3	-15.4901
2.31	-15.0995
2.32	-14.7196
2.33	-14.3499
2.34	-13.9899
2.35	-13.6392
2.36	-13.2972
2.37	-12.9636
2.38	-12.6381
2.39	-12.3203
2.4	-12.0099
2.41	-11.7066
2.42	-11.4103
2.43	-11.1205
2.44	-10.8372
2.45	-10.5601
2.46	-10.2889
2.47	-10.0236
2.48	-9.76399
2.49	-9.50985
2.5	-9.26105
2.51	-9.01746
2.52	-8.77895
2.53	-8.54539
2.54	-8.31668
2.55	-8.0927
2.56	-7.87335
2.57	-7.65853
2.58	-7.44816
2.59	-7.24215
2.6	-7.04041
2.61	-6.84288
2.62	-6.64948
2.63	-6.46014
2.64	-6.27479
2.65	-6.09338
2.66	-5.91584
2.67	-5.74212
2.68	-5.57216
2.69	-5.40592
2.7	-5.24334

2.71	-5.08438
2.72	-4.92898
2.73	-4.77711
2.74	-4.62871
2.75	-4.48376
2.76	-4.34219
2.77	-4.20399
2.78	-4.0691
2.79	-3.93748
2.8	-3.8091
2.81	-3.68392
2.82	-3.5619
2.83	-3.44299
2.84	-3.32717
2.85	-3.21439
2.86	-3.10462
2.87	-2.99781
2.88	-2.89393
2.89	-2.79293
2.9	-2.69478
2.91	-2.59944
2.92	-2.50686
2.93	-2.41701
2.94	-2.32984
2.95	-2.24531
2.96	-2.16339
2.97	-2.08401
2.98	-2.00716
2.99	-1.93277
3	-1.86081
3.01	-1.79122
3.02	-1.72398
3.03	-1.65902
3.04	-1.59631
3.05	-1.53581
3.06	-1.47745
3.07	-1.42121
3.08	-1.36703
3.09	-1.31486
3.1	-1.26467
3.11	-1.2164
3.12	-1.17001
3.13	-1.12545
3.14	-1.08268
3.15	-1.04166
3.16	-1.00233
3.17	-0.96465
3.18	-0.92858

3.19	-0.89407
3.2	-0.86109
3.21	-0.82958
3.22	-0.79951
3.23	-0.77083
3.24	-0.7435
3.25	-0.71748
3.26	-0.69272
3.27	-0.6692
3.28	-0.64687
3.29	-0.6257
3.3	-0.60564
3.31	-0.58665
3.32	-0.56871
3.33	-0.55178
3.34	-0.53581
3.35	-0.52079
3.36	-0.50667
3.37	-0.49342
3.38	-0.48102
3.39	-0.46942
3.4	-0.4586
3.41	-0.44854
3.42	-0.43919
3.43	-0.43054
3.44	-0.42256
3.45	-0.41521
3.46	-0.40849
3.47	-0.40235
3.48	-0.39678
3.49	-0.39175
3.5	-0.38724
3.51	-0.38323
3.52	-0.37969
3.53	-0.37661
3.54	-0.37396
3.55	-0.37173
3.56	-0.3699
3.57	-0.36845
3.58	-0.36735
3.59	-0.36661
3.6	-0.36618
3.61	-0.36607
3.62	-0.36626
3.63	-0.36673
3.64	-0.36746
3.65	-0.36845
3.66	-0.36967

3.67	-0.37112
3.68	-0.37279
3.69	-0.37466
3.7	-0.37671
3.71	-0.37895
3.72	-0.38135
3.73	-0.38392
3.74	-0.38663
3.75	-0.38948
3.76	-0.39245
3.77	-0.39555
3.78	-0.39876
3.79	-0.40207
3.8	-0.40548
3.81	-0.40898
3.82	-0.41255
3.83	-0.4162
3.84	-0.41991
3.85	-0.42369
3.86	-0.42752
3.87	-0.43139
3.88	-0.43531
3.89	-0.43926
3.9	-0.44325
3.91	-0.44726
3.92	-0.45129
3.93	-0.45534
3.94	-0.4594
3.95	-0.46346
3.96	-0.46754
3.97	-0.47161
3.98	-0.47567
3.99	-0.47973
4	-0.48378
4.01	-0.48781
4.02	-0.49183
4.03	-0.49582
4.04	-0.4998
4.05	-0.50374
4.06	-0.50766
4.07	-0.51154
4.08	-0.5154
4.09	-0.51921
4.1	-0.52299
4.11	-0.52673
4.12	-0.53043
4.13	-0.53408
4.14	-0.53769

4.15	-0.54126
4.16	-0.54477
4.17	-0.54823
4.18	-0.55165
4.19	-0.55501
4.2	-0.55832
4.21	-0.56157
4.22	-0.56477
4.23	-0.56791
4.24	-0.57099
4.25	-0.57401
4.26	-0.57698
4.27	-0.57988
4.28	-0.58273
4.29	-0.58551
4.3	-0.58823
4.31	-0.59089
4.32	-0.59348
4.33	-0.59601
4.34	-0.59848
4.35	-0.60089
4.36	-0.60323
4.37	-0.6055
4.38	-0.60771
4.39	-0.60986
4.4	-0.61194
4.41	-0.61395
4.42	-0.6159
4.43	-0.61778
4.44	-0.6196
4.45	-0.62135
4.46	-0.62304
4.47	-0.62466
4.48	-0.62622
4.49	-0.62771
4.5	-0.62914
4.51	-0.6305
4.52	-0.6318
4.53	-0.63303
4.54	-0.6342
4.55	-0.63531
4.56	-0.63635
4.57	-0.63733
4.58	-0.63824
4.59	-0.6391
4.6	-0.63989
4.61	-0.64062
4.62	-0.64128

4.63	-0.64189
4.64	-0.64243
4.65	-0.64292
4.66	-0.64334
4.67	-0.6437
4.68	-0.64401
4.69	-0.64426
4.7	-0.64445
4.71	-0.64458
4.72	-0.64465
4.73	-0.64467
4.74	-0.64463
4.75	-0.64453
4.76	-0.64438
4.77	-0.64418
4.78	-0.64392
4.79	-0.6436
4.8	-0.64324
4.81	-0.64282
4.82	-0.64235
4.83	-0.64183
4.84	-0.64126
4.85	-0.64063
4.86	-0.63996
4.87	-0.63924
4.88	-0.63847
4.89	-0.63765
4.9	-0.63679
4.91	-0.63588
4.92	-0.63492
4.93	-0.63392
4.94	-0.63287
4.95	-0.63178
4.96	-0.63064
4.97	-0.62946
4.98	-0.62824
4.99	-0.62698
5	-0.62567
5.01	-0.62433
5.02	-0.62295
5.03	-0.62152
5.04	-0.62006
5.05	-0.61856
5.06	-0.61702
5.07	-0.61545
5.08	-0.61384
5.09	-0.61219
5.1	-0.61051

5.11	-0.6088
5.12	-0.60705
5.13	-0.60527
5.14	-0.60346
5.15	-0.60161
5.16	-0.59974
5.17	-0.59783
5.18	-0.59589
5.19	-0.59393
5.2	-0.59194
5.21	-0.58992
5.22	-0.58787
5.23	-0.58579
5.24	-0.58369
5.25	-0.58157
5.26	-0.57942
5.27	-0.57724
5.28	-0.57504
5.29	-0.57282
5.3	-0.57058
5.31	-0.56832
5.32	-0.56603
5.33	-0.56372
5.34	-0.5614
5.35	-0.55905
5.36	-0.55669
5.37	-0.55431
5.38	-0.55191
5.39	-0.5495
5.4	-0.54707
5.41	-0.54462
5.42	-0.54216
5.43	-0.53969
5.44	-0.5372
5.45	-0.5347
5.46	-0.53218
5.47	-0.52966
5.48	-0.52712
5.49	-0.52457
5.5	-0.52201
5.51	-0.51945
5.52	-0.51687
5.53	-0.51429
5.54	-0.51169
5.55	-0.50909
5.56	-0.50649
5.57	-0.50388
5.58	-0.50126

5.59	-0.49864
5.6	-0.49601
5.61	-0.49338
5.62	-0.49075
5.63	-0.48811
5.64	-0.48547
5.65	-0.48283
5.66	-0.48019
5.67	-0.47755
5.68	-0.47491
5.69	-0.47226
5.7	-0.46962
5.71	-0.46699
5.72	-0.46435
5.73	-0.46171
5.74	-0.45908
5.75	-0.45646
5.76	-0.45383
5.77	-0.45122
5.78	-0.4486
5.79	-0.446
5.8	-0.44339
5.81	-0.4408
5.82	-0.43821
5.83	-0.43563
5.84	-0.43306
5.85	-0.4305
5.86	-0.42794
5.87	-0.4254
5.88	-0.42286
5.89	-0.42034
5.9	-0.41783
5.91	-0.41533
5.92	-0.41284
5.93	-0.41036
5.94	-0.40789
5.95	-0.40544
5.96	-0.403
5.97	-0.40058
5.98	-0.39817
5.99	-0.39578
6	-0.3934
6.01	-0.39103
6.02	-0.38869
6.03	-0.38635
6.04	-0.38404
6.05	-0.38174
6.06	-0.37946

6.07	-0.3772
6.08	-0.37496
6.09	-0.37274
6.1	-0.37053
6.11	-0.36835
6.12	-0.36619
6.13	-0.36404
6.14	-0.36192
6.15	-0.35982
6.16	-0.35774
6.17	-0.35568
6.18	-0.35364
6.19	-0.35163
6.2	-0.34964
6.21	-0.34767
6.22	-0.34572
6.23	-0.3438
6.24	-0.34191
6.25	-0.34004
6.26	-0.33819
6.27	-0.33637
6.28	-0.33457
6.29	-0.3328
6.3	-0.33105
6.31	-0.32933
6.32	-0.32764
6.33	-0.32598
6.34	-0.32434
6.35	-0.32273
6.36	-0.32114
6.37	-0.31959
6.38	-0.31806
6.39	-0.31656
6.4	-0.31509
6.41	-0.31365
6.42	-0.31223
6.43	-0.31085
6.44	-0.3095
6.45	-0.30817
6.46	-0.30688
6.47	-0.30561
6.48	-0.30438
6.49	-0.30318
6.5	-0.302
6.51	-0.30086
6.52	-0.29975
6.53	-0.29867
6.54	-0.29762

6.55	-0.29661
6.56	-0.29562
6.57	-0.29467
6.58	-0.29375
6.59	-0.29286
6.6	-0.292
6.61	-0.29118
6.62	-0.29039
6.63	-0.28963
6.64	-0.28891
6.65	-0.28821
6.66	-0.28755
6.67	-0.28693
6.68	-0.28634
6.69	-0.28578
6.7	-0.28525
6.71	-0.28476
6.72	-0.2843
6.73	-0.28387
6.74	-0.28348
6.75	-0.28312
6.76	-0.2828
6.77	-0.28251
6.78	-0.28226
6.79	-0.28203
6.8	-0.28185
6.81	-0.28169
6.82	-0.28157
6.83	-0.28149
6.84	-0.28144
6.85	-0.28142
6.86	-0.28144
6.87	-0.28149
6.88	-0.28157
6.89	-0.28169
6.9	-0.28185
6.91	-0.28203
6.92	-0.28226
6.93	-0.28251
6.94	-0.2828
6.95	-0.28312
6.96	-0.28348
6.97	-0.28387
6.98	-0.2843
6.99	-0.28476
7	-0.28525
7.01	-0.28578
7.02	-0.28634

7.03	-0.28693
7.04	-0.28755
7.05	-0.28821
7.06	-0.28891
7.07	-0.28963
7.08	-0.29039
7.09	-0.29118
7.1	-0.292
7.11	-0.29286
7.12	-0.29375
7.13	-0.29467
7.14	-0.29562
7.15	-0.29661
7.16	-0.29762
7.17	-0.29867
7.18	-0.29975
7.19	-0.30086
7.2	-0.302
7.21	-0.30318
7.22	-0.30438
7.23	-0.30561
7.24	-0.30688
7.25	-0.30817
7.26	-0.3095
7.27	-0.31085
7.28	-0.31223
7.29	-0.31365
7.3	-0.31509
7.31	-0.31656
7.32	-0.31806
7.33	-0.31959
7.34	-0.32114
7.35	-0.32273
7.36	-0.32434
7.37	-0.32598
7.38	-0.32764
7.39	-0.32933
7.4	-0.33105
7.41	-0.3328
7.42	-0.33457
7.43	-0.33637
7.44	-0.33819
7.45	-0.34004
7.46	-0.34191
7.47	-0.3438
7.48	-0.34572
7.49	-0.34767
7.5	-0.34964

7.51	-0.35163
7.52	-0.35364
7.53	-0.35568
7.54	-0.35774
7.55	-0.35982
7.56	-0.36192
7.57	-0.36404
7.58	-0.36619
7.59	-0.36835
7.6	-0.37053
7.61	-0.37274
7.62	-0.37496
7.63	-0.3772
7.64	-0.37946
7.65	-0.38174
7.66	-0.38404
7.67	-0.38635
7.68	-0.38869
7.69	-0.39103
7.7	-0.3934
7.71	-0.39578
7.72	-0.39817
7.73	-0.40058
7.74	-0.403
7.75	-0.40544
7.76	-0.40789
7.77	-0.41036
7.78	-0.41284
7.79	-0.41533
7.8	-0.41783
7.81	-0.42034
7.82	-0.42286
7.83	-0.4254
7.84	-0.42794
7.85	-0.4305
7.86	-0.43306
7.87	-0.43563
7.88	-0.43821
7.89	-0.4408
7.9	-0.44339
7.91	-0.446
7.92	-0.4486
7.93	-0.45122
7.94	-0.45383
7.95	-0.45646
7.96	-0.45908
7.97	-0.46171
7.98	-0.46435

7.99	-0.46699
8	-0.46962
8.01	-0.47226
8.02	-0.47491
8.03	-0.47755
8.04	-0.48019
8.05	-0.48283
8.06	-0.48547
8.07	-0.48811
8.08	-0.49075
8.09	-0.49338
8.1	-0.49601
8.11	-0.49864
8.12	-0.50126
8.13	-0.50388
8.14	-0.50649
8.15	-0.50909
8.16	-0.51169
8.17	-0.51429
8.18	-0.51687
8.19	-0.51945
8.2	-0.52201
8.21	-0.52457
8.22	-0.52712
8.23	-0.52966
8.24	-0.53218
8.25	-0.5347
8.26	-0.5372
8.27	-0.53969
8.28	-0.54216
8.29	-0.54462
8.3	-0.54707
8.31	-0.5495
8.32	-0.55191
8.33	-0.55431
8.34	-0.55669
8.35	-0.55905
8.36	-0.5614
8.37	-0.56372
8.38	-0.56603
8.39	-0.56832
8.4	-0.57058
8.41	-0.57282
8.42	-0.57504
8.43	-0.57724
8.44	-0.57942
8.45	-0.58157
8.46	-0.58369

8.47	-0.58579
8.48	-0.58787
8.49	-0.58992
8.5	-0.59194
8.51	-0.59393
8.52	-0.59589
8.53	-0.59783
8.54	-0.59974
8.55	-0.60161
8.56	-0.60346
8.57	-0.60527
8.58	-0.60705
8.59	-0.6088
8.6	-0.61051
8.61	-0.61219
8.62	-0.61384
8.63	-0.61545
8.64	-0.61702
8.65	-0.61856
8.66	-0.62006
8.67	-0.62152
8.68	-0.62295
8.69	-0.62433
8.7	-0.62567
8.71	-0.62698
8.72	-0.62824
8.73	-0.62946
8.74	-0.63064
8.75	-0.63178
8.76	-0.63287
8.77	-0.63392
8.78	-0.63492
8.79	-0.63588
8.8	-0.63679
8.81	-0.63765
8.82	-0.63847
8.83	-0.63924
8.84	-0.63996
8.85	-0.64063
8.86	-0.64126
8.87	-0.64183
8.88	-0.64235
8.89	-0.64282
8.9	-0.64324
8.91	-0.6436
8.92	-0.64392
8.93	-0.64418
8.94	-0.64438

8.95	-0.64453
8.96	-0.64463
8.97	-0.64467
8.98	-0.64465
8.99	-0.64458
9	-0.64445
9.01	-0.64426
9.02	-0.64401
9.03	-0.6437
9.04	-0.64334
9.05	-0.64292
9.06	-0.64243
9.07	-0.64189
9.08	-0.64128
9.09	-0.64062
9.1	-0.63989
9.11	-0.6391
9.12	-0.63824
9.13	-0.63733
9.14	-0.63635
9.15	-0.63531
9.16	-0.6342
9.17	-0.63303
9.18	-0.6318
9.19	-0.6305
9.2	-0.62914
9.21	-0.62771
9.22	-0.62622
9.23	-0.62466
9.24	-0.62304
9.25	-0.62135
9.26	-0.6196
9.27	-0.61778
9.28	-0.6159
9.29	-0.61395
9.3	-0.61194
9.31	-0.60986
9.32	-0.60771
9.33	-0.6055
9.34	-0.60323
9.35	-0.60089
9.36	-0.59848
9.37	-0.59601
9.38	-0.59348
9.39	-0.59089
9.4	-0.58823
9.41	-0.58551
9.42	-0.58273

9.43	-0.57988
9.44	-0.57698
9.45	-0.57401
9.46	-0.57099
9.47	-0.56791
9.48	-0.56477
9.49	-0.56157
9.5	-0.55832
9.51	-0.55501
9.52	-0.55165
9.53	-0.54823
9.54	-0.54477
9.55	-0.54126
9.56	-0.53769
9.57	-0.53408
9.58	-0.53043
9.59	-0.52673
9.6	-0.52299
9.61	-0.51921
9.62	-0.5154
9.63	-0.51154
9.64	-0.50766
9.65	-0.50374
9.66	-0.4998
9.67	-0.49582
9.68	-0.49183
9.69	-0.48781
9.7	-0.48378
9.71	-0.47973
9.72	-0.47567
9.73	-0.47161
9.74	-0.46754
9.75	-0.46346
9.76	-0.4594
9.77	-0.45534
9.78	-0.45129
9.79	-0.44726
9.8	-0.44325
9.81	-0.43926
9.82	-0.43531
9.83	-0.43139
9.84	-0.42752
9.85	-0.42369
9.86	-0.41991
9.87	-0.4162
9.88	-0.41255
9.89	-0.40898
9.9	-0.40548

9.91	-0.40207
9.92	-0.39876
9.93	-0.39555
9.94	-0.39245
9.95	-0.38948
9.96	-0.38663
9.97	-0.38392
9.98	-0.38135
9.99	-0.37895
10	-0.37671
10.01	-0.37466
10.02	-0.37279
10.03	-0.37112
10.04	-0.36967
10.05	-0.36845
10.06	-0.36746
10.07	-0.36673
10.08	-0.36626
10.09	-0.36607
10.1	-0.36618
10.11	-0.36661
10.12	-0.36735
10.13	-0.36845
10.14	-0.3699
10.15	-0.37173
10.16	-0.37396
10.17	-0.37661
10.18	-0.37969
10.19	-0.38323
10.2	-0.38724
10.21	-0.39175
10.22	-0.39678
10.23	-0.40235
10.24	-0.40849
10.25	-0.41521
10.26	-0.42256
10.27	-0.43054
10.28	-0.43919
10.29	-0.44854
10.3	-0.4586
10.31	-0.46942
10.32	-0.48102
10.33	-0.49342
10.34	-0.50667
10.35	-0.52079
10.36	-0.53581
10.37	-0.55178
10.38	-0.56871

10.39	-0.58665
10.4	-0.60564
10.41	-0.6257
10.42	-0.64687
10.43	-0.6692
10.44	-0.69272
10.45	-0.71748
10.46	-0.7435
10.47	-0.77083
10.48	-0.79951
10.49	-0.82958
10.5	-0.86109
10.51	-0.89407
10.52	-0.92858
10.53	-0.96465
10.54	-1.00233
10.55	-1.04166
10.56	-1.08268
10.57	-1.12545
10.58	-1.17001
10.59	-1.2164
10.6	-1.26467
10.61	-1.31486
10.62	-1.36703
10.63	-1.42121
10.64	-1.47745
10.65	-1.53581
10.66	-1.59631
10.67	-1.65902
10.68	-1.72398
10.69	-1.79122
10.7	-1.86081
10.71	-1.93277
10.72	-2.00716
10.73	-2.08401
10.74	-2.16339
10.75	-2.24531
10.76	-2.32984
10.77	-2.41701
10.78	-2.50686
10.79	-2.59944
10.8	-2.69478
10.81	-2.79293
10.82	-2.89393
10.83	-2.99781
10.84	-3.10462
10.85	-3.21439
10.86	-3.32717

10.87	-3.44299
10.88	-3.5619
10.89	-3.68392
10.9	-3.8091
10.91	-3.93748
10.92	-4.0691
10.93	-4.20399
10.94	-4.34219
10.95	-4.48376
10.96	-4.62871
10.97	-4.77711
10.98	-4.92898
10.99	-5.08438
11	-5.24334
11.01	-5.40592
11.02	-5.57216
11.03	-5.74212
11.04	-5.91584
11.05	-6.09338
11.06	-6.27479
11.07	-6.46014
11.08	-6.64948
11.09	-6.84288
11.1	-7.04041
11.11	-7.24215
11.12	-7.44816
11.13	-7.65853
11.14	-7.87335
11.15	-8.0927
11.16	-8.31668
11.17	-8.54539
11.18	-8.77895
11.19	-9.01746
11.2	-9.26105
11.21	-9.50985
11.22	-9.76399
11.23	-10.0236
11.24	-10.2889
11.25	-10.5601
11.26	-10.8372
11.27	-11.1205
11.28	-11.4103
11.29	-11.7066
11.3	-12.0099
11.31	-12.3203
11.32	-12.6381
11.33	-12.9636
11.34	-13.2972

11.35	-13.6392
11.36	-13.9899
11.37	-14.3499
11.38	-14.7196
11.39	-15.0995
11.4	-15.4901
11.41	-15.8921
11.42	-16.3061
11.43	-16.7329
11.44	-17.1733
11.45	-17.6283
11.46	-18.0989
11.47	-18.5863
11.48	-19.0919
11.49	-19.6171
11.5	-20.1638
11.51	-20.734
11.52	-21.33
11.53	-21.9546
11.54	-22.6111
11.55	-23.3034
11.56	-24.0362
11.57	-24.8153
11.58	-25.6479
11.59	-26.543
11.6	-27.5124
11.61	-28.5714
11.62	-29.7409
11.63	-31.0502
11.64	-32.5425
11.65	-34.2846
11.66	-36.3892
11.67	-39.0689
11.68	-42.8056
11.69	-49.175
11.7	-76.0816
11.71	-48.7187
11.72	-43.0386
11.73	-39.7364
11.74	-37.4275
11.75	-35.6681
11.76	-34.2583
11.77	-33.0909
11.78	-32.1016
11.79	-31.2489
11.8	-30.5042
11.81	-29.8474
11.82	-29.2633

11.83	-28.7404
11.84	-28.27
11.85	-27.8448
11.86	-27.4593
11.87	-27.1086
11.88	-26.7891
11.89	-26.4973
11.9	-26.2306
11.91	-25.9866
11.92	-25.7632
11.93	-25.5587
11.94	-25.3717
11.95	-25.2006
11.96	-25.0444
11.97	-24.9019
11.98	-24.7722
11.99	-24.6545
12	-24.548
12.01	-24.452
12.02	-24.3659
12.03	-24.2892
12.04	-24.2212
12.05	-24.1616
12.06	-24.1098
12.07	-24.0656
12.08	-24.0285
12.09	-23.9981
12.1	-23.9743
12.11	-23.9567
12.12	-23.9449
12.13	-23.9389
12.14	-23.9382
12.15	-23.9428
12.16	-23.9524
12.17	-23.9669
12.18	-23.9859
12.19	-24.0095
12.2	-24.0374
12.21	-24.0695
12.22	-24.1056
12.23	-24.1456
12.24	-24.1894
12.25	-24.2369
12.26	-24.288
12.27	-24.3425
12.28	-24.4004
12.29	-24.4616
12.3	-24.526

12.31	-24.5935
12.32	-24.6641
12.33	-24.7376
12.34	-24.8141
12.35	-24.8934
12.36	-24.9755
12.37	-25.0603
12.38	-25.1478
12.39	-25.238
12.4	-25.3307
12.41	-25.426
12.42	-25.5237
12.43	-25.624
12.44	-25.7267
12.45	-25.8318
12.46	-25.9392
12.47	-26.049
12.48	-26.1612
12.49	-26.2756
12.5	-26.3923
12.51	-26.5112
12.52	-26.6324
12.53	-26.7559
12.54	-26.8815
12.55	-27.0094
12.56	-27.1394
12.57	-27.2716
12.58	-27.406
12.59	-27.5426
12.6	-27.6813
12.61	-27.8223
12.62	-27.9654
12.63	-28.1106
12.64	-28.2581
12.65	-28.4077
12.66	-28.5595
12.67	-28.7135
12.68	-28.8698
12.69	-29.0283
12.7	-29.189
12.71	-29.3519
12.72	-29.5171
12.73	-29.6847
12.74	-29.8545
12.75	-30.0266
12.76	-30.2012
12.77	-30.3781
12.78	-30.5574

12.79	-30.7391
12.8	-30.9233
12.81	-31.1101
12.82	-31.2994
12.83	-31.4912
12.84	-31.6857
12.85	-31.8828
12.86	-32.0826
12.87	-32.2852
12.88	-32.4906
12.89	-32.6988
12.9	-32.91
12.91	-33.1241
12.92	-33.3412
12.93	-33.5614
12.94	-33.7848
12.95	-34.0114
12.96	-34.2412
12.97	-34.4745
12.98	-34.7112
12.99	-34.9514
13	-35.1952
13.01	-35.4428
13.02	-35.6942
13.03	-35.9494
13.04	-36.2087
13.05	-36.4721
13.06	-36.7398
13.07	-37.0118
13.08	-37.2884
13.09	-37.5696
13.1	-37.8555
13.11	-38.1464
13.12	-38.4424
13.13	-38.7436
13.14	-39.0503
13.15	-39.3626
13.16	-39.6807
13.17	-40.0049
13.18	-40.3353
13.19	-40.6722
13.2	-41.0159
13.21	-41.3665
13.22	-41.7245
13.23	-42.0901
13.24	-42.4635
13.25	-42.8453
13.26	-43.2357

13.27	-43.635
13.28	-44.0439
13.29	-44.4626
13.3	-44.8916
13.31	-45.3316
13.32	-45.783
13.33	-46.2465
13.34	-46.7227
13.35	-47.2123
13.36	-47.7162
13.37	-48.235
13.38	-48.7699
13.39	-49.3217
13.4	-49.8916
13.41	-50.4809
13.42	-51.0908
13.43	-51.7229
13.44	-52.3789
13.45	-53.0605
13.46	-53.77
13.47	-54.5097
13.48	-55.2822
13.49	-56.0906
13.5	-56.9385
13.51	-57.8299
13.52	-58.7694
13.53	-59.7626
13.54	-60.8161
13.55	-61.9375
13.56	-63.1363
13.57	-64.4239
13.58	-65.8146
13.59	-67.3263
13.6	-68.9822
13.61	-70.8127
13.62	-72.859
13.63	-75.1788
13.64	-77.8568
13.65	-81.0241
13.75	-81.0241
13.76	-77.8568
13.77	-75.1788
13.78	-72.859
13.79	-70.8127
13.8	-68.9822
13.81	-67.3263
13.82	-65.8146
13.83	-64.4239

13.84	-63.1363
13.85	-61.9375
13.86	-60.8161
13.87	-59.7626
13.88	-58.7694
13.89	-57.8299
13.9	-56.9385
13.91	-56.0906
13.92	-55.2822
13.93	-54.5097
13.94	-53.77
13.95	-53.0605
13.96	-52.3789
13.97	-51.7229
13.98	-51.0908
13.99	-50.4809
14	-49.8916
14.01	-49.3217
14.02	-48.7699
14.03	-48.235
14.04	-47.7162
14.05	-47.2123
14.06	-46.7227
14.07	-46.2465
14.08	-45.783
14.09	-45.3316
14.1	-44.8916
14.11	-44.4626
14.12	-44.0439
14.13	-43.635
14.14	-43.2357
14.15	-42.8453
14.16	-42.4635
14.17	-42.0901
14.18	-41.7245
14.19	-41.3665
14.2	-41.0159
14.21	-40.6722
14.22	-40.3353
14.23	-40.0049
14.24	-39.6807
14.25	-39.3626
14.26	-39.0503
14.27	-38.7436
14.28	-38.4424
14.29	-38.1464
14.3	-37.8555
14.31	-37.5696

14.32	-37.2884
14.33	-37.0118
14.34	-36.7398
14.35	-36.4721
14.36	-36.2087
14.37	-35.9494
14.38	-35.6942
14.39	-35.4428
14.4	-35.1952
14.41	-34.9514
14.42	-34.7112
14.43	-34.4745
14.44	-34.2412
14.45	-34.0114
14.46	-33.7848
14.47	-33.5614
14.48	-33.3412
14.49	-33.1241
14.5	-32.91
14.51	-32.6988
14.52	-32.4906
14.53	-32.2852
14.54	-32.0826
14.55	-31.8828
14.56	-31.6857
14.57	-31.4912
14.58	-31.2994
14.59	-31.1101
14.6	-30.9233
14.61	-30.7391
14.62	-30.5574
14.63	-30.3781
14.64	-30.2012
14.65	-30.0266
14.66	-29.8545
14.67	-29.6847
14.68	-29.5171
14.69	-29.3519
14.7	-29.189
14.71	-29.0283
14.72	-28.8698
14.73	-28.7135
14.74	-28.5595
14.75	-28.4077
14.76	-28.2581
14.77	-28.1106
14.78	-27.9654
14.79	-27.8223

14.8	-27.6813
14.81	-27.5426
14.82	-27.406
14.83	-27.2716
14.84	-27.1394
14.85	-27.0094
14.86	-26.8815
14.87	-26.7559
14.88	-26.6324
14.89	-26.5112
14.9	-26.3923
14.91	-26.2756
14.92	-26.1612
14.93	-26.049
14.94	-25.9392
14.95	-25.8318
14.96	-25.7267
14.97	-25.624
14.98	-25.5237
14.99	-25.426
15	-25.3307
15.01	-25.238
15.02	-25.1478
15.03	-25.0603
15.04	-24.9755
15.05	-24.8934
15.06	-24.8141
15.07	-24.7376
15.08	-24.6641
15.09	-24.5935
15.1	-24.526
15.11	-24.4616
15.12	-24.4004
15.13	-24.3425
15.14	-24.288
15.15	-24.2369
15.16	-24.1894
15.17	-24.1456
15.18	-24.1056
15.19	-24.0695
15.2	-24.0374
15.21	-24.0095
15.22	-23.9859
15.23	-23.9669
15.24	-23.9524
15.25	-23.9428
15.26	-23.9382
15.27	-23.9389

15.28	-23.9449
15.29	-23.9567
15.3	-23.9743
15.31	-23.9981
15.32	-24.0285
15.33	-24.0656
15.34	-24.1098
15.35	-24.1616
15.36	-24.2212
15.37	-24.2892
15.38	-24.3659
15.39	-24.452
15.4	-24.548
15.41	-24.6545
15.42	-24.7722
15.43	-24.9019
15.44	-25.0444
15.45	-25.2006
15.46	-25.3717
15.47	-25.5587
15.48	-25.7632
15.49	-25.9866
15.5	-26.2306
15.51	-26.4973
15.52	-26.7891
15.53	-27.1086
15.54	-27.4593
15.55	-27.8448
15.56	-28.27
15.57	-28.7404
15.58	-29.2633
15.59	-29.8474
15.6	-30.5042
15.61	-31.2489
15.62	-32.1016
15.63	-33.0909
15.64	-34.2583
15.65	-35.6681
15.66	-37.4275
15.67	-39.7364
15.68	-43.0386
15.69	-48.7187
15.7	-76.0816
15.71	-49.175
15.72	-42.8056
15.73	-39.0689
15.74	-36.3892
15.75	-34.2846

15.76	-32.5425
15.77	-31.0502
15.78	-29.7409
15.79	-28.5714
15.8	-27.5124
15.81	-26.543
15.82	-25.6479
15.83	-24.8153
15.84	-24.0362
15.85	-23.3034
15.86	-22.6111
15.87	-21.9546
15.88	-21.33
15.89	-20.734
15.9	-20.1638
15.91	-19.6171
15.92	-19.0919
15.93	-18.5863
15.94	-18.0989
15.95	-17.6283
15.96	-17.1733
15.97	-16.7329
15.98	-16.3061
15.99	-15.8921
16	-15.4901

one balun_70 ohms Data File

Frequency (	DB[S11]
0	-0.01042
0.01	-6.24E-11
0.02	-9.99E-10
0.03	-5.06E-09
0.04	-1.60E-08
0.05	-3.90E-08
0.06	-8.09E-08
0.07	-1.50E-07
0.08	-2.56E-07
0.09	-4.10E-07
0.1	-6.24E-07
0.11	-9.14E-07
0.12	-1.29E-06
0.13	-1.78E-06
0.14	-2.40E-06
0.15	-3.16E-06
0.16	-4.09E-06
0.17	-5.21E-06
0.18	-6.55E-06
0.19	-8.13E-06

0.2	-9.98E-06
0.21	-1.21E-05
0.22	-1.46E-05
0.23	-1.75E-05
0.24	-2.07E-05
0.25	-2.44E-05
0.26	-2.85E-05
0.27	-3.31E-05
0.28	-3.83E-05
0.29	-4.41E-05
0.3	-5.05E-05
0.31	-5.76E-05
0.32	-6.54E-05
0.33	-7.39E-05
0.34	-8.33E-05
0.35	-9.35E-05
0.36	-0.0001
0.37	-0.00012
0.38	-0.00013
0.39	-0.00014
0.4	-0.00016
0.41	-0.00018
0.42	-0.00019
0.43	-0.00021
0.44	-0.00023
0.45	-0.00025
0.46	-0.00028
0.47	-0.0003
0.48	-0.00033
0.49	-0.00036
0.5	-0.00039
0.51	-0.00042
0.52	-0.00045
0.53	-0.00049
0.54	-0.00053
0.55	-0.00057
0.56	-0.00061
0.57	-0.00065
0.58	-0.0007
0.59	-0.00075
0.6	-0.0008
0.61	-0.00086
0.62	-0.00091
0.63	-0.00097
0.64	-0.00103
0.65	-0.0011
0.66	-0.00117
0.67	-0.00124

0.68	-0.00131
0.69	-0.00139
0.7	-0.00147
0.71	-0.00156
0.72	-0.00165
0.73	-0.00174
0.74	-0.00183
0.75	-0.00193
0.76	-0.00203
0.77	-0.00214
0.78	-0.00225
0.79	-0.00237
0.8	-0.00248
0.81	-0.00261
0.82	-0.00273
0.83	-0.00287
0.84	-0.003
0.85	-0.00314
0.86	-0.00329
0.87	-0.00344
0.88	-0.00359
0.89	-0.00375
0.9	-0.00391
0.91	-0.00408
0.92	-0.00426
0.93	-0.00443
0.94	-0.00462
0.95	-0.0048
0.96	-0.005
0.97	-0.0052
0.98	-0.0054
0.99	-0.00561
1	-0.00582
1.01	-0.00604
1.02	-0.00626
1.03	-0.00649
1.04	-0.00673
1.05	-0.00697
1.06	-0.00721
1.07	-0.00746
1.08	-0.00771
1.09	-0.00797
1.1	-0.00824
1.11	-0.0085
1.12	-0.00878
1.13	-0.00905
1.14	-0.00934
1.15	-0.00962

1.16	-0.00991
1.17	-0.01021
1.18	-0.01051
1.19	-0.01081
1.2	-0.01111
1.21	-0.01142
1.22	-0.01173
1.23	-0.01205
1.24	-0.01237
1.25	-0.01269
1.26	-0.01301
1.27	-0.01333
1.28	-0.01366
1.29	-0.01398
1.3	-0.01431
1.31	-0.01464
1.32	-0.01497
1.33	-0.01529
1.34	-0.01562
1.35	-0.01594
1.36	-0.01627
1.37	-0.01659
1.38	-0.01691
1.39	-0.01722
1.4	-0.01753
1.41	-0.01784
1.42	-0.01814
1.43	-0.01843
1.44	-0.01872
1.45	-0.01901
1.46	-0.01928
1.47	-0.01955
1.48	-0.0198
1.49	-0.02005
1.5	-0.02028
1.51	-0.02051
1.52	-0.02072
1.53	-0.02092
1.54	-0.0211
1.55	-0.02127
1.56	-0.02143
1.57	-0.02157
1.58	-0.02169
1.59	-0.02179
1.6	-0.02187
1.61	-0.02193
1.62	-0.02197
1.63	-0.02199

1.64	-0.02199
1.65	-0.02196
1.66	-0.02191
1.67	-0.02184
1.68	-0.02173
1.69	-0.0216
1.7	-0.02145
1.71	-0.02126
1.72	-0.02105
1.73	-0.0208
1.74	-0.02053
1.75	-0.02023
1.76	-0.01989
1.77	-0.01953
1.78	-0.01913
1.79	-0.0187
1.8	-0.01825
1.81	-0.01776
1.82	-0.01724
1.83	-0.01669
1.84	-0.01612
1.85	-0.01551
1.86	-0.01488
1.87	-0.01422
1.88	-0.01354
1.89	-0.01284
1.9	-0.01212
1.91	-0.01138
1.92	-0.01062
1.93	-0.00986
1.94	-0.00908
1.95	-0.0083
1.96	-0.00752
1.97	-0.00674
1.98	-0.00598
1.99	-0.00522
2	-0.00449
2.01	-0.00379
2.02	-0.00311
2.03	-0.00248
2.04	-0.0019
2.05	-0.00138
2.06	-0.00093
2.07	-0.00055
2.08	-0.00026
2.09	-7.53E-05
2.1	-7.61E-07
2.11	-5.21E-05

2.12	-0.00024
2.13	-0.00059
2.14	-0.00111
2.15	-0.00181
2.16	-0.00272
2.17	-0.00385
2.18	-0.00523
2.19	-0.00686
2.2	-0.00879
2.21	-0.01102
2.22	-0.01359
2.23	-0.01651
2.24	-0.01982
2.25	-0.02354
2.26	-0.02771
2.27	-0.03234
2.28	-0.03748
2.29	-0.04315
2.3	-0.04939
2.31	-0.05624
2.32	-0.06372
2.33	-0.07189
2.34	-0.08078
2.35	-0.09043
2.36	-0.10088
2.37	-0.11217
2.38	-0.12436
2.39	-0.13748
2.4	-0.15159
2.41	-0.16673
2.42	-0.18295
2.43	-0.2003
2.44	-0.21883
2.45	-0.23861
2.46	-0.25967
2.47	-0.28207
2.48	-0.30588
2.49	-0.33114
2.5	-0.35792
2.51	-0.38626
2.52	-0.41623
2.53	-0.44788
2.54	-0.48127
2.55	-0.51646
2.56	-0.5535
2.57	-0.59245
2.58	-0.63337
2.59	-0.67631

2.6	-0.72132
2.61	-0.76845
2.62	-0.81777
2.63	-0.8693
2.64	-0.92312
2.65	-0.97925
2.66	-1.03775
2.67	-1.09865
2.68	-1.16199
2.69	-1.2278
2.7	-1.29613
2.71	-1.36699
2.72	-1.44042
2.73	-1.51644
2.74	-1.59505
2.75	-1.67629
2.76	-1.76015
2.77	-1.84665
2.78	-1.93579
2.79	-2.02756
2.8	-2.12195
2.81	-2.21896
2.82	-2.31858
2.83	-2.42077
2.84	-2.52551
2.85	-2.63278
2.86	-2.74253
2.87	-2.85473
2.88	-2.96934
2.89	-3.0863
2.9	-3.20557
2.91	-3.32708
2.92	-3.45076
2.93	-3.57657
2.94	-3.70441
2.95	-3.83422
2.96	-3.96591
2.97	-4.0994
2.98	-4.23461
2.99	-4.37143
3	-4.50977
3.01	-4.64952
3.02	-4.79059
3.03	-4.93286
3.04	-5.07622
3.05	-5.22054
3.06	-5.36572
3.07	-5.51162

3.08	-5.65811
3.09	-5.80507
3.1	-5.95235
3.11	-6.09981
3.12	-6.24732
3.13	-6.39473
3.14	-6.54188
3.15	-6.68863
3.16	-6.83483
3.17	-6.9803
3.18	-7.12491
3.19	-7.26849
3.2	-7.41087
3.21	-7.55189
3.22	-7.6914
3.23	-7.82923
3.24	-7.96521
3.25	-8.09919
3.26	-8.23101
3.27	-8.36051
3.28	-8.48754
3.29	-8.61195
3.3	-8.7336
3.31	-8.85234
3.32	-8.96806
3.33	-9.08061
3.34	-9.18988
3.35	-9.29577
3.36	-9.39817
3.37	-9.49699
3.38	-9.59215
3.39	-9.68359
3.4	-9.77124
3.41	-9.85506
3.42	-9.93501
3.43	-10.0111
3.44	-10.0832
3.45	-10.1515
3.46	-10.2158
3.47	-10.2763
3.48	-10.333
3.49	-10.3859
3.5	-10.435
3.51	-10.4805
3.52	-10.5224
3.53	-10.5608
3.54	-10.5957
3.55	-10.6274

3.56	-10.6558
3.57	-10.681
3.58	-10.7033
3.59	-10.7227
3.6	-10.7392
3.61	-10.7532
3.62	-10.7645
3.63	-10.7735
3.64	-10.7801
3.65	-10.7846
3.66	-10.787
3.67	-10.7874
3.68	-10.786
3.69	-10.7829
3.7	-10.7781
3.71	-10.7719
3.72	-10.7643
3.73	-10.7553
3.74	-10.7452
3.75	-10.7339
3.76	-10.7216
3.77	-10.7084
3.78	-10.6944
3.79	-10.6795
3.8	-10.664
3.81	-10.6479
3.82	-10.6312
3.83	-10.614
3.84	-10.5964
3.85	-10.5784
3.86	-10.5601
3.87	-10.5415
3.88	-10.5228
3.89	-10.5038
3.9	-10.4848
3.91	-10.4656
3.92	-10.4465
3.93	-10.4273
3.94	-10.4081
3.95	-10.389
3.96	-10.37
3.97	-10.3512
3.98	-10.3324
3.99	-10.3139
4	-10.2955
4.01	-10.2773
4.02	-10.2594
4.03	-10.2417

4.04	-10.2243
4.05	-10.2072
4.06	-10.1904
4.07	-10.1738
4.08	-10.1576
4.09	-10.1418
4.1	-10.1262
4.11	-10.1111
4.12	-10.0963
4.13	-10.0818
4.14	-10.0678
4.15	-10.0541
4.16	-10.0408
4.17	-10.0279
4.18	-10.0154
4.19	-10.0033
4.2	-9.99157
4.21	-9.98029
4.22	-9.96941
4.23	-9.95896
4.24	-9.94892
4.25	-9.9393
4.26	-9.9301
4.27	-9.92132
4.28	-9.91297
4.29	-9.90504
4.3	-9.89754
4.31	-9.89046
4.32	-9.88381
4.33	-9.87759
4.34	-9.87179
4.35	-9.86641
4.36	-9.86146
4.37	-9.85694
4.38	-9.85284
4.39	-9.84916
4.4	-9.84591
4.41	-9.84308
4.42	-9.84067
4.43	-9.83868
4.44	-9.83711
4.45	-9.83595
4.46	-9.83522
4.47	-9.8349
4.48	-9.83499
4.49	-9.8355
4.5	-9.83642
4.51	-9.83775

4.52	-9.8395
4.53	-9.84165
4.54	-9.84421
4.55	-9.84717
4.56	-9.85054
4.57	-9.85432
4.58	-9.85849
4.59	-9.86307
4.6	-9.86805
4.61	-9.87343
4.62	-9.8792
4.63	-9.88538
4.64	-9.89195
4.65	-9.89891
4.66	-9.90627
4.67	-9.91402
4.68	-9.92216
4.69	-9.93069
4.7	-9.93962
4.71	-9.94893
4.72	-9.95863
4.73	-9.96871
4.74	-9.97919
4.75	-9.99005
4.76	-10.0013
4.77	-10.0129
4.78	-10.0249
4.79	-10.0373
4.8	-10.0501
4.81	-10.0633
4.82	-10.0768
4.83	-10.0907
4.84	-10.105
4.85	-10.1197
4.86	-10.1348
4.87	-10.1502
4.88	-10.166
4.89	-10.1822
4.9	-10.1988
4.91	-10.2158
4.92	-10.2331
4.93	-10.2508
4.94	-10.2689
4.95	-10.2874
4.96	-10.3063
4.97	-10.3255
4.98	-10.3451
4.99	-10.3651

5	-10.3855
5.01	-10.4062
5.02	-10.4273
5.03	-10.4488
5.04	-10.4707
5.05	-10.493
5.06	-10.5156
5.07	-10.5387
5.08	-10.5621
5.09	-10.5859
5.1	-10.6101
5.11	-10.6346
5.12	-10.6596
5.13	-10.6849
5.14	-10.7107
5.15	-10.7368
5.16	-10.7633
5.17	-10.7902
5.18	-10.8175
5.19	-10.8451
5.2	-10.8732
5.21	-10.9017
5.22	-10.9305
5.23	-10.9598
5.24	-10.9894
5.25	-11.0195
5.26	-11.0499
5.27	-11.0807
5.28	-11.112
5.29	-11.1436
5.3	-11.1757
5.31	-11.2082
5.32	-11.241
5.33	-11.2743
5.34	-11.308
5.35	-11.3421
5.36	-11.3766
5.37	-11.4116
5.38	-11.4469
5.39	-11.4827
5.4	-11.5189
5.41	-11.5555
5.42	-11.5925
5.43	-11.63
5.44	-11.6679
5.45	-11.7062
5.46	-11.745
5.47	-11.7842

5.48	-11.8238
5.49	-11.8639
5.5	-11.9044
5.51	-11.9453
5.52	-11.9867
5.53	-12.0285
5.54	-12.0708
5.55	-12.1136
5.56	-12.1568
5.57	-12.2004
5.58	-12.2445
5.59	-12.2891
5.6	-12.3341
5.61	-12.3796
5.62	-12.4256
5.63	-12.472
5.64	-12.5189
5.65	-12.5663
5.66	-12.6142
5.67	-12.6625
5.68	-12.7113
5.69	-12.7606
5.7	-12.8104
5.71	-12.8607
5.72	-12.9114
5.73	-12.9627
5.74	-13.0144
5.75	-13.0667
5.76	-13.1194
5.77	-13.1727
5.78	-13.2264
5.79	-13.2807
5.8	-13.3355
5.81	-13.3908
5.82	-13.4465
5.83	-13.5028
5.84	-13.5597
5.85	-13.617
5.86	-13.6749
5.87	-13.7332
5.88	-13.7921
5.89	-13.8515
5.9	-13.9115
5.91	-13.972
5.92	-14.033
5.93	-14.0945
5.94	-14.1565
5.95	-14.2191

5.96	-14.2822
5.97	-14.3458
5.98	-14.41
5.99	-14.4747
6	-14.5399
6.01	-14.6056
6.02	-14.6719
6.03	-14.7387
6.04	-14.806
6.05	-14.8738
6.06	-14.9421
6.07	-15.011
6.08	-15.0803
6.09	-15.1502
6.1	-15.2206
6.11	-15.2914
6.12	-15.3628
6.13	-15.4346
6.14	-15.5069
6.15	-15.5797
6.16	-15.6529
6.17	-15.7266
6.18	-15.8007
6.19	-15.8752
6.2	-15.9502
6.21	-16.0256
6.22	-16.1013
6.23	-16.1775
6.24	-16.254
6.25	-16.3308
6.26	-16.408
6.27	-16.4855
6.28	-16.5633
6.29	-16.6413
6.3	-16.7196
6.31	-16.7981
6.32	-16.8768
6.33	-16.9557
6.34	-17.0346
6.35	-17.1137
6.36	-17.1929
6.37	-17.2721
6.38	-17.3513
6.39	-17.4304
6.4	-17.5094
6.41	-17.5883
6.42	-17.6671
6.43	-17.7456

6.44	-17.8238
6.45	-17.9017
6.46	-17.9792
6.47	-18.0562
6.48	-18.1328
6.49	-18.2087
6.5	-18.2841
6.51	-18.3587
6.52	-18.4325
6.53	-18.5055
6.54	-18.5775
6.55	-18.6485
6.56	-18.7184
6.57	-18.7872
6.58	-18.8546
6.59	-18.9208
6.6	-18.9854
6.61	-19.0486
6.62	-19.1101
6.63	-19.1699
6.64	-19.2279
6.65	-19.284
6.66	-19.3381
6.67	-19.39
6.68	-19.4399
6.69	-19.4874
6.7	-19.5326
6.71	-19.5753
6.72	-19.6155
6.73	-19.653
6.74	-19.6879
6.75	-19.72
6.76	-19.7493
6.77	-19.7756
6.78	-19.799
6.79	-19.8194
6.8	-19.8368
6.81	-19.851
6.82	-19.8621
6.83	-19.8701
6.84	-19.8748
6.85	-19.8764
6.86	-19.8748
6.87	-19.8701
6.88	-19.8621
6.89	-19.851
6.9	-19.8368
6.91	-19.8194

6.92	-19.799
6.93	-19.7756
6.94	-19.7493
6.95	-19.72
6.96	-19.6879
6.97	-19.653
6.98	-19.6155
6.99	-19.5753
7	-19.5326
7.01	-19.4874
7.02	-19.4399
7.03	-19.39
7.04	-19.3381
7.05	-19.284
7.06	-19.2279
7.07	-19.1699
7.08	-19.1101
7.09	-19.0486
7.1	-18.9854
7.11	-18.9208
7.12	-18.8546
7.13	-18.7872
7.14	-18.7184
7.15	-18.6485
7.16	-18.5775
7.17	-18.5055
7.18	-18.4325
7.19	-18.3587
7.2	-18.2841
7.21	-18.2087
7.22	-18.1328
7.23	-18.0562
7.24	-17.9792
7.25	-17.9017
7.26	-17.8238
7.27	-17.7456
7.28	-17.6671
7.29	-17.5883
7.3	-17.5094
7.31	-17.4304
7.32	-17.3513
7.33	-17.2721
7.34	-17.1929
7.35	-17.1137
7.36	-17.0346
7.37	-16.9557
7.38	-16.8768
7.39	-16.7981

7.4	-16.7196
7.41	-16.6413
7.42	-16.5633
7.43	-16.4855
7.44	-16.408
7.45	-16.3308
7.46	-16.254
7.47	-16.1775
7.48	-16.1013
7.49	-16.0256
7.5	-15.9502
7.51	-15.8752
7.52	-15.8007
7.53	-15.7266
7.54	-15.6529
7.55	-15.5797
7.56	-15.5069
7.57	-15.4346
7.58	-15.3628
7.59	-15.2914
7.6	-15.2206
7.61	-15.1502
7.62	-15.0803
7.63	-15.011
7.64	-14.9421
7.65	-14.8738
7.66	-14.806
7.67	-14.7387
7.68	-14.6719
7.69	-14.6056
7.7	-14.5399
7.71	-14.4747
7.72	-14.41
7.73	-14.3458
7.74	-14.2822
7.75	-14.2191
7.76	-14.1565
7.77	-14.0945
7.78	-14.033
7.79	-13.972
7.8	-13.9115
7.81	-13.8515
7.82	-13.7921
7.83	-13.7332
7.84	-13.6749
7.85	-13.617
7.86	-13.5597
7.87	-13.5028

7.88	-13.4465
7.89	-13.3908
7.9	-13.3355
7.91	-13.2807
7.92	-13.2264
7.93	-13.1727
7.94	-13.1194
7.95	-13.0667
7.96	-13.0144
7.97	-12.9627
7.98	-12.9114
7.99	-12.8607
8	-12.8104
8.01	-12.7606
8.02	-12.7113
8.03	-12.6625
8.04	-12.6142
8.05	-12.5663
8.06	-12.5189
8.07	-12.472
8.08	-12.4256
8.09	-12.3796
8.1	-12.3341
8.11	-12.2891
8.12	-12.2445
8.13	-12.2004
8.14	-12.1568
8.15	-12.1136
8.16	-12.0708
8.17	-12.0285
8.18	-11.9867
8.19	-11.9453
8.2	-11.9044
8.21	-11.8639
8.22	-11.8238
8.23	-11.7842
8.24	-11.745
8.25	-11.7062
8.26	-11.6679
8.27	-11.63
8.28	-11.5925
8.29	-11.5555
8.3	-11.5189
8.31	-11.4827
8.32	-11.4469
8.33	-11.4116
8.34	-11.3766
8.35	-11.3421

8.36	-11.308
8.37	-11.2743
8.38	-11.241
8.39	-11.2082
8.4	-11.1757
8.41	-11.1436
8.42	-11.112
8.43	-11.0807
8.44	-11.0499
8.45	-11.0195
8.46	-10.9894
8.47	-10.9598
8.48	-10.9305
8.49	-10.9017
8.5	-10.8732
8.51	-10.8451
8.52	-10.8175
8.53	-10.7902
8.54	-10.7633
8.55	-10.7368
8.56	-10.7107
8.57	-10.6849
8.58	-10.6596
8.59	-10.6346
8.6	-10.6101
8.61	-10.5859
8.62	-10.5621
8.63	-10.5387
8.64	-10.5156
8.65	-10.493
8.66	-10.4707
8.67	-10.4488
8.68	-10.4273
8.69	-10.4062
8.7	-10.3855
8.71	-10.3651
8.72	-10.3451
8.73	-10.3255
8.74	-10.3063
8.75	-10.2874
8.76	-10.2689
8.77	-10.2508
8.78	-10.2331
8.79	-10.2158
8.8	-10.1988
8.81	-10.1822
8.82	-10.166
8.83	-10.1502

8.84	-10.1348
8.85	-10.1197
8.86	-10.105
8.87	-10.0907
8.88	-10.0768
8.89	-10.0633
8.9	-10.0501
8.91	-10.0373
8.92	-10.0249
8.93	-10.0129
8.94	-10.0013
8.95	-9.99005
8.96	-9.97919
8.97	-9.96871
8.98	-9.95863
8.99	-9.94893
9	-9.93962
9.01	-9.93069
9.02	-9.92216
9.03	-9.91402
9.04	-9.90627
9.05	-9.89891
9.06	-9.89195
9.07	-9.88538
9.08	-9.8792
9.09	-9.87343
9.1	-9.86805
9.11	-9.86307
9.12	-9.85849
9.13	-9.85432
9.14	-9.85054
9.15	-9.84717
9.16	-9.84421
9.17	-9.84165
9.18	-9.8395
9.19	-9.83775
9.2	-9.83642
9.21	-9.8355
9.22	-9.83499
9.23	-9.8349
9.24	-9.83522
9.25	-9.83595
9.26	-9.83711
9.27	-9.83868
9.28	-9.84067
9.29	-9.84308
9.3	-9.84591
9.31	-9.84916

9.32	-9.85284
9.33	-9.85694
9.34	-9.86146
9.35	-9.86641
9.36	-9.87179
9.37	-9.87759
9.38	-9.88381
9.39	-9.89046
9.4	-9.89754
9.41	-9.90504
9.42	-9.91297
9.43	-9.92132
9.44	-9.9301
9.45	-9.9393
9.46	-9.94892
9.47	-9.95896
9.48	-9.96941
9.49	-9.98029
9.5	-9.99157
9.51	-10.0033
9.52	-10.0154
9.53	-10.0279
9.54	-10.0408
9.55	-10.0541
9.56	-10.0678
9.57	-10.0818
9.58	-10.0963
9.59	-10.1111
9.6	-10.1262
9.61	-10.1418
9.62	-10.1576
9.63	-10.1738
9.64	-10.1904
9.65	-10.2072
9.66	-10.2243
9.67	-10.2417
9.68	-10.2594
9.69	-10.2773
9.7	-10.2955
9.71	-10.3139
9.72	-10.3324
9.73	-10.3512
9.74	-10.37
9.75	-10.389
9.76	-10.4081
9.77	-10.4273
9.78	-10.4465
9.79	-10.4656

9.8	-10.4848
9.81	-10.5038
9.82	-10.5228
9.83	-10.5415
9.84	-10.5601
9.85	-10.5784
9.86	-10.5964
9.87	-10.614
9.88	-10.6312
9.89	-10.6479
9.9	-10.664
9.91	-10.6795
9.92	-10.6944
9.93	-10.7084
9.94	-10.7216
9.95	-10.7339
9.96	-10.7452
9.97	-10.7553
9.98	-10.7643
9.99	-10.7719
10	-10.7781
10.01	-10.7829
10.02	-10.786
10.03	-10.7874
10.04	-10.787
10.05	-10.7846
10.06	-10.7801
10.07	-10.7735
10.08	-10.7645
10.09	-10.7532
10.1	-10.7392
10.11	-10.7227
10.12	-10.7033
10.13	-10.681
10.14	-10.6558
10.15	-10.6274
10.16	-10.5957
10.17	-10.5608
10.18	-10.5224
10.19	-10.4805
10.2	-10.435
10.21	-10.3859
10.22	-10.333
10.23	-10.2763
10.24	-10.2158
10.25	-10.1515
10.26	-10.0832
10.27	-10.0111

10.28	-9.93501
10.29	-9.85506
10.3	-9.77124
10.31	-9.68359
10.32	-9.59215
10.33	-9.49699
10.34	-9.39817
10.35	-9.29577
10.36	-9.18988
10.37	-9.08061
10.38	-8.96806
10.39	-8.85234
10.4	-8.7336
10.41	-8.61195
10.42	-8.48754
10.43	-8.36051
10.44	-8.23101
10.45	-8.09919
10.46	-7.96521
10.47	-7.82923
10.48	-7.6914
10.49	-7.55189
10.5	-7.41087
10.51	-7.26849
10.52	-7.12491
10.53	-6.9803
10.54	-6.83483
10.55	-6.68863
10.56	-6.54188
10.57	-6.39473
10.58	-6.24732
10.59	-6.09981
10.6	-5.95235
10.61	-5.80507
10.62	-5.65811
10.63	-5.51162
10.64	-5.36572
10.65	-5.22054
10.66	-5.07622
10.67	-4.93286
10.68	-4.79059
10.69	-4.64952
10.7	-4.50977
10.71	-4.37143
10.72	-4.23461
10.73	-4.0994
10.74	-3.96591
10.75	-3.83422

10.76	-3.70441
10.77	-3.57657
10.78	-3.45076
10.79	-3.32708
10.8	-3.20557
10.81	-3.0863
10.82	-2.96934
10.83	-2.85473
10.84	-2.74253
10.85	-2.63278
10.86	-2.52551
10.87	-2.42077
10.88	-2.31858
10.89	-2.21896
10.9	-2.12195
10.91	-2.02756
10.92	-1.93579
10.93	-1.84665
10.94	-1.76015
10.95	-1.67629
10.96	-1.59505
10.97	-1.51644
10.98	-1.44042
10.99	-1.36699
11	-1.29613
11.01	-1.2278
11.02	-1.16199
11.03	-1.09865
11.04	-1.03775
11.05	-0.97925
11.06	-0.92312
11.07	-0.8693
11.08	-0.81777
11.09	-0.76845
11.1	-0.72132
11.11	-0.67631
11.12	-0.63337
11.13	-0.59245
11.14	-0.5535
11.15	-0.51646
11.16	-0.48127
11.17	-0.44788
11.18	-0.41623
11.19	-0.38626
11.2	-0.35792
11.21	-0.33114
11.22	-0.30588
11.23	-0.28207

11.24	-0.25967
11.25	-0.23861
11.26	-0.21883
11.27	-0.2003
11.28	-0.18295
11.29	-0.16673
11.3	-0.15159
11.31	-0.13748
11.32	-0.12436
11.33	-0.11217
11.34	-0.10088
11.35	-0.09043
11.36	-0.08078
11.37	-0.07189
11.38	-0.06372
11.39	-0.05624
11.4	-0.04939
11.41	-0.04315
11.42	-0.03748
11.43	-0.03234
11.44	-0.02771
11.45	-0.02354
11.46	-0.01982
11.47	-0.01651
11.48	-0.01359
11.49	-0.01102
11.5	-0.00879
11.51	-0.00686
11.52	-0.00523
11.53	-0.00385
11.54	-0.00272
11.55	-0.00181
11.56	-0.00111
11.57	-0.00059
11.58	-0.00024
11.59	-5.21E-05
11.6	-7.61E-07
11.61	-7.53E-05
11.62	-0.00026
11.63	-0.00055
11.64	-0.00093
11.65	-0.00138
11.66	-0.0019
11.67	-0.00248
11.68	-0.00311
11.69	-0.00379
11.7	-0.00449
11.71	-0.00522

11.72	-0.00598
11.73	-0.00674
11.74	-0.00752
11.75	-0.0083
11.76	-0.00908
11.77	-0.00986
11.78	-0.01062
11.79	-0.01138
11.8	-0.01212
11.81	-0.01284
11.82	-0.01354
11.83	-0.01422
11.84	-0.01488
11.85	-0.01551
11.86	-0.01612
11.87	-0.01669
11.88	-0.01724
11.89	-0.01776
11.9	-0.01825
11.91	-0.0187
11.92	-0.01913
11.93	-0.01953
11.94	-0.01989
11.95	-0.02023
11.96	-0.02053
11.97	-0.0208
11.98	-0.02105
11.99	-0.02126
12	-0.02145
12.01	-0.0216
12.02	-0.02173
12.03	-0.02184
12.04	-0.02191
12.05	-0.02196
12.06	-0.02199
12.07	-0.02199
12.08	-0.02197
12.09	-0.02193
12.1	-0.02187
12.11	-0.02179
12.12	-0.02169
12.13	-0.02157
12.14	-0.02143
12.15	-0.02127
12.16	-0.0211
12.17	-0.02092
12.18	-0.02072
12.19	-0.02051

12.2	-0.02028
12.21	-0.02005
12.22	-0.0198
12.23	-0.01955
12.24	-0.01928
12.25	-0.01901
12.26	-0.01872
12.27	-0.01843
12.28	-0.01814
12.29	-0.01784
12.3	-0.01753
12.31	-0.01722
12.32	-0.01691
12.33	-0.01659
12.34	-0.01627
12.35	-0.01594
12.36	-0.01562
12.37	-0.01529
12.38	-0.01497
12.39	-0.01464
12.4	-0.01431
12.41	-0.01398
12.42	-0.01366
12.43	-0.01333
12.44	-0.01301
12.45	-0.01269
12.46	-0.01237
12.47	-0.01205
12.48	-0.01173
12.49	-0.01142
12.5	-0.01111
12.51	-0.01081
12.52	-0.01051
12.53	-0.01021
12.54	-0.00991
12.55	-0.00962
12.56	-0.00934
12.57	-0.00905
12.58	-0.00878
12.59	-0.0085
12.6	-0.00824
12.61	-0.00797
12.62	-0.00771
12.63	-0.00746
12.64	-0.00721
12.65	-0.00697
12.66	-0.00673
12.67	-0.00649

12.68	-0.00626
12.69	-0.00604
12.7	-0.00582
12.71	-0.00561
12.72	-0.0054
12.73	-0.0052
12.74	-0.005
12.75	-0.0048
12.76	-0.00462
12.77	-0.00443
12.78	-0.00426
12.79	-0.00408
12.8	-0.00391
12.81	-0.00375
12.82	-0.00359
12.83	-0.00344
12.84	-0.00329
12.85	-0.00314
12.86	-0.003
12.87	-0.00287
12.88	-0.00273
12.89	-0.00261
12.9	-0.00248
12.91	-0.00237
12.92	-0.00225
12.93	-0.00214
12.94	-0.00203
12.95	-0.00193
12.96	-0.00183
12.97	-0.00174
12.98	-0.00165
12.99	-0.00156
13	-0.00147
13.01	-0.00139
13.02	-0.00131
13.03	-0.00124
13.04	-0.00117
13.05	-0.0011
13.06	-0.00103
13.07	-0.00097
13.08	-0.00091
13.09	-0.00086
13.1	-0.0008
13.11	-0.00075
13.12	-0.0007
13.13	-0.00065
13.14	-0.00061
13.15	-0.00057

13.16	-0.00053
13.17	-0.00049
13.18	-0.00045
13.19	-0.00042
13.2	-0.00039
13.21	-0.00036
13.22	-0.00033
13.23	-0.0003
13.24	-0.00028
13.25	-0.00025
13.26	-0.00023
13.27	-0.00021
13.28	-0.00019
13.29	-0.00018
13.3	-0.00016
13.31	-0.00014
13.32	-0.00013
13.33	-0.00012
13.34	-0.0001
13.35	-9.35E-05
13.36	-8.33E-05
13.37	-7.39E-05
13.38	-6.54E-05
13.39	-5.76E-05
13.4	-5.05E-05
13.41	-4.41E-05
13.42	-3.83E-05
13.43	-3.31E-05
13.44	-2.85E-05
13.45	-2.44E-05
13.46	-2.07E-05
13.47	-1.75E-05
13.48	-1.46E-05
13.49	-1.21E-05
13.5	-9.98E-06
13.51	-8.13E-06
13.52	-6.55E-06
13.53	-5.21E-06
13.54	-4.09E-06
13.55	-3.16E-06
13.56	-2.40E-06
13.57	-1.78E-06
13.58	-1.29E-06
13.59	-9.14E-07
13.6	-6.24E-07
13.61	-4.10E-07
13.62	-2.56E-07
13.63	-1.50E-07

13.64	-8.09E-08
13.65	-3.90E-08
13.66	-1.60E-08
13.67	-5.06E-09
13.68	-9.99E-10
13.69	-6.24E-11
13.7	0
13.71	-6.24E-11
13.72	-9.99E-10
13.73	-5.06E-09
13.74	-1.60E-08
13.75	-3.90E-08
13.76	-8.09E-08
13.77	-1.50E-07
13.78	-2.56E-07
13.79	-4.10E-07
13.8	-6.24E-07
13.81	-9.14E-07
13.82	-1.29E-06
13.83	-1.78E-06
13.84	-2.40E-06
13.85	-3.16E-06
13.86	-4.09E-06
13.87	-5.21E-06
13.88	-6.55E-06
13.89	-8.13E-06
13.9	-9.98E-06
13.91	-1.21E-05
13.92	-1.46E-05
13.93	-1.75E-05
13.94	-2.07E-05
13.95	-2.44E-05
13.96	-2.85E-05
13.97	-3.31E-05
13.98	-3.83E-05
13.99	-4.41E-05
14	-5.05E-05
14.01	-5.76E-05
14.02	-6.54E-05
14.03	-7.39E-05
14.04	-8.33E-05
14.05	-9.35E-05
14.06	-0.0001
14.07	-0.00012
14.08	-0.00013
14.09	-0.00014
14.1	-0.00016
14.11	-0.00018

14.12	-0.00019
14.13	-0.00021
14.14	-0.00023
14.15	-0.00025
14.16	-0.00028
14.17	-0.0003
14.18	-0.00033
14.19	-0.00036
14.2	-0.00039
14.21	-0.00042
14.22	-0.00045
14.23	-0.00049
14.24	-0.00053
14.25	-0.00057
14.26	-0.00061
14.27	-0.00065
14.28	-0.0007
14.29	-0.00075
14.3	-0.0008
14.31	-0.00086
14.32	-0.00091
14.33	-0.00097
14.34	-0.00103
14.35	-0.0011
14.36	-0.00117
14.37	-0.00124
14.38	-0.00131
14.39	-0.00139
14.4	-0.00147
14.41	-0.00156
14.42	-0.00165
14.43	-0.00174
14.44	-0.00183
14.45	-0.00193
14.46	-0.00203
14.47	-0.00214
14.48	-0.00225
14.49	-0.00237
14.5	-0.00248
14.51	-0.00261
14.52	-0.00273
14.53	-0.00287
14.54	-0.003
14.55	-0.00314
14.56	-0.00329
14.57	-0.00344
14.58	-0.00359
14.59	-0.00375

14.6	-0.00391
14.61	-0.00408
14.62	-0.00426
14.63	-0.00443
14.64	-0.00462
14.65	-0.0048
14.66	-0.005
14.67	-0.0052
14.68	-0.0054
14.69	-0.00561
14.7	-0.00582
14.71	-0.00604
14.72	-0.00626
14.73	-0.00649
14.74	-0.00673
14.75	-0.00697
14.76	-0.00721
14.77	-0.00746
14.78	-0.00771
14.79	-0.00797
14.8	-0.00824
14.81	-0.0085
14.82	-0.00878
14.83	-0.00905
14.84	-0.00934
14.85	-0.00962
14.86	-0.00991
14.87	-0.01021
14.88	-0.01051
14.89	-0.01081
14.9	-0.01111
14.91	-0.01142
14.92	-0.01173
14.93	-0.01205
14.94	-0.01237
14.95	-0.01269
14.96	-0.01301
14.97	-0.01333
14.98	-0.01366
14.99	-0.01398
15	-0.01431
15.01	-0.01464
15.02	-0.01497
15.03	-0.01529
15.04	-0.01562
15.05	-0.01594
15.06	-0.01627
15.07	-0.01659

15.08	-0.01691
15.09	-0.01722
15.1	-0.01753
15.11	-0.01784
15.12	-0.01814
15.13	-0.01843
15.14	-0.01872
15.15	-0.01901
15.16	-0.01928
15.17	-0.01955
15.18	-0.0198
15.19	-0.02005
15.2	-0.02028
15.21	-0.02051
15.22	-0.02072
15.23	-0.02092
15.24	-0.0211
15.25	-0.02127
15.26	-0.02143
15.27	-0.02157
15.28	-0.02169
15.29	-0.02179
15.3	-0.02187
15.31	-0.02193
15.32	-0.02197
15.33	-0.02199
15.34	-0.02199
15.35	-0.02196
15.36	-0.02191
15.37	-0.02184
15.38	-0.02173
15.39	-0.0216
15.4	-0.02145
15.41	-0.02126
15.42	-0.02105
15.43	-0.0208
15.44	-0.02053
15.45	-0.02023
15.46	-0.01989
15.47	-0.01953
15.48	-0.01913
15.49	-0.0187
15.5	-0.01825
15.51	-0.01776
15.52	-0.01724
15.53	-0.01669
15.54	-0.01612
15.55	-0.01551

15.56	-0.01488
15.57	-0.01422
15.58	-0.01354
15.59	-0.01284
15.6	-0.01212
15.61	-0.01138
15.62	-0.01062
15.63	-0.00986
15.64	-0.00908
15.65	-0.0083
15.66	-0.00752
15.67	-0.00674
15.68	-0.00598
15.69	-0.00522
15.7	-0.00449
15.71	-0.00379
15.72	-0.00311
15.73	-0.00248
15.74	-0.0019
15.75	-0.00138
15.76	-0.00093
15.77	-0.00055
15.78	-0.00026
15.79	-7.53E-05
15.8	-7.61E-07
15.81	-5.21E-05
15.82	-0.00024
15.83	-0.00059
15.84	-0.00111
15.85	-0.00181
15.86	-0.00272
15.87	-0.00385
15.88	-0.00523
15.89	-0.00686
15.9	-0.00879
15.91	-0.01102
15.92	-0.01359
15.93	-0.01651
15.94	-0.01982
15.95	-0.02354
15.96	-0.02771
15.97	-0.03234
15.98	-0.03748
15.99	-0.04315
16	-0.04939

one balun_70 ohms Data File

Frequency (	DB[S21]

0.05	-80.4651
0.06	-77.2979
0.07	-74.6201
0.08	-72.3004
0.09	-70.2544
0.1	-68.4242
0.11	-66.7686
0.12	-65.2572
0.13	-63.8668
0.14	-62.5795
0.15	-61.3812
0.16	-60.2602
0.17	-59.2072
0.18	-58.2145
0.19	-57.2754
0.2	-56.3846
0.21	-55.5373
0.22	-54.7294
0.23	-53.9575
0.24	-53.2185
0.25	-52.5096
0.26	-51.8287
0.27	-51.1734
0.28	-50.5421
0.29	-49.9329
0.3	-49.3445
0.31	-48.7754
0.32	-48.2244
0.33	-47.6904
0.34	-47.1724
0.35	-46.6695
0.36	-46.1808
0.37	-45.7056
0.38	-45.2431
0.39	-44.7927
0.4	-44.3538
0.41	-43.9258
0.42	-43.5081
0.43	-43.1004
0.44	-42.7022
0.45	-42.313
0.46	-41.9324
0.47	-41.5601
0.48	-41.1957
0.49	-40.839
0.5	-40.4896
0.51	-40.1472
0.52	-39.8116

0.53	-39.4825
0.54	-39.1596
0.55	-38.8428
0.56	-38.5319
0.57	-38.2266
0.58	-37.9268
0.59	-37.6322
0.6	-37.3428
0.61	-37.0582
0.62	-36.7785
0.63	-36.5035
0.64	-36.2329
0.65	-35.9667
0.66	-35.7048
0.67	-35.447
0.68	-35.1932
0.69	-34.9434
0.7	-34.6973
0.71	-34.455
0.72	-34.2163
0.73	-33.9811
0.74	-33.7494
0.75	-33.5211
0.76	-33.296
0.77	-33.0742
0.78	-32.8554
0.79	-32.6398
0.8	-32.4272
0.81	-32.2175
0.82	-32.0108
0.83	-31.8068
0.84	-31.6057
0.85	-31.4073
0.86	-31.2115
0.87	-31.0184
0.88	-30.8279
0.89	-30.6399
0.9	-30.4545
0.91	-30.2715
0.92	-30.0909
0.93	-29.9128
0.94	-29.737
0.95	-29.5635
0.96	-29.3924
0.97	-29.2235
0.98	-29.0569
0.99	-28.8925
1	-28.7303

1.01	-28.5703
1.02	-28.4124
1.03	-28.2567
1.04	-28.1031
1.05	-27.9516
1.06	-27.8022
1.07	-27.6549
1.08	-27.5096
1.09	-27.3664
1.1	-27.2252
1.11	-27.0861
1.12	-26.949
1.13	-26.814
1.14	-26.6809
1.15	-26.5499
1.16	-26.4209
1.17	-26.2939
1.18	-26.1689
1.19	-26.0459
1.2	-25.9249
1.21	-25.806
1.22	-25.6891
1.23	-25.5743
1.24	-25.4615
1.25	-25.3507
1.26	-25.242
1.27	-25.1354
1.28	-25.0309
1.29	-24.9285
1.3	-24.8283
1.31	-24.7302
1.32	-24.6343
1.33	-24.5405
1.34	-24.449
1.35	-24.3598
1.36	-24.2728
1.37	-24.1882
1.38	-24.1058
1.39	-24.0259
1.4	-23.9484
1.41	-23.8733
1.42	-23.8008
1.43	-23.7307
1.44	-23.6633
1.45	-23.5985
1.46	-23.5364
1.47	-23.4771
1.48	-23.4206

1.49	-23.367
1.5	-23.3163
1.51	-23.2686
1.52	-23.2241
1.53	-23.1827
1.54	-23.1446
1.55	-23.1098
1.56	-23.0786
1.57	-23.0508
1.58	-23.0268
1.59	-23.0065
1.6	-22.9901
1.61	-22.9778
1.62	-22.9696
1.63	-22.9658
1.64	-22.9665
1.65	-22.9718
1.66	-22.982
1.67	-22.9972
1.68	-23.0176
1.69	-23.0436
1.7	-23.0752
1.71	-23.1128
1.72	-23.1567
1.73	-23.2071
1.74	-23.2644
1.75	-23.329
1.76	-23.4012
1.77	-23.4814
1.78	-23.5702
1.79	-23.6679
1.8	-23.7753
1.81	-23.8928
1.82	-24.0211
1.83	-24.1611
1.84	-24.3135
1.85	-24.4792
1.86	-24.6594
1.87	-24.8551
1.88	-25.0678
1.89	-25.2988
1.9	-25.55
1.91	-25.8234
1.92	-26.1212
1.93	-26.4462
1.94	-26.8016
1.95	-27.1912
1.96	-27.6196

1.97	-28.0924
1.98	-28.6166
1.99	-29.2009
2	-29.8566
2.01	-30.5985
2.02	-31.4464
2.03	-32.4281
2.04	-33.5841
2.05	-34.9768
2.06	-36.7095
2.07	-38.9731
2.08	-42.1858
2.09	-47.6101
2.1	-67.5666
2.11	-49.2095
2.12	-42.5163
2.13	-38.6751
2.14	-35.942
2.15	-33.8035
2.16	-32.0369
2.17	-30.5253
2.18	-29.1995
2.19	-28.0154
2.2	-26.9428
2.21	-25.9605
2.22	-25.0527
2.23	-24.2077
2.24	-23.4162
2.25	-22.6709
2.26	-21.966
2.27	-21.2967
2.28	-20.6591
2.29	-20.0498
2.3	-19.4662
2.31	-18.9058
2.32	-18.3666
2.33	-17.8469
2.34	-17.3451
2.35	-16.8599
2.36	-16.3902
2.37	-15.9349
2.38	-15.4931
2.39	-15.0639
2.4	-14.6468
2.41	-14.2409
2.42	-13.8458
2.43	-13.4609
2.44	-13.0857

2.45	-12.7198
2.46	-12.3629
2.47	-12.0145
2.48	-11.6744
2.49	-11.3422
2.5	-11.0177
2.51	-10.7007
2.52	-10.391
2.53	-10.0882
2.54	-9.79236
2.55	-9.50316
2.56	-9.22048
2.57	-8.94417
2.58	-8.67412
2.59	-8.41018
2.6	-8.15226
2.61	-7.90024
2.62	-7.65403
2.63	-7.41353
2.64	-7.17866
2.65	-6.94933
2.66	-6.72548
2.67	-6.50702
2.68	-6.29388
2.69	-6.086
2.7	-5.88332
2.71	-5.68576
2.72	-5.49327
2.73	-5.30578
2.74	-5.12324
2.75	-4.94557
2.76	-4.77273
2.77	-4.60465
2.78	-4.44127
2.79	-4.28252
2.8	-4.12835
2.81	-3.97868
2.82	-3.83346
2.83	-3.69262
2.84	-3.55609
2.85	-3.42379
2.86	-3.29567
2.87	-3.17165
2.88	-3.05165
2.89	-2.9356
2.9	-2.82344
2.91	-2.71507
2.92	-2.61042

2.93	-2.50942
2.94	-2.41199
2.95	-2.31804
2.96	-2.2275
2.97	-2.14029
2.98	-2.05632
2.99	-1.97551
3	-1.89778
3.01	-1.82306
3.02	-1.75126
3.03	-1.68229
3.04	-1.61608
3.05	-1.55256
3.06	-1.49163
3.07	-1.43323
3.08	-1.37727
3.09	-1.32368
3.1	-1.27238
3.11	-1.2233
3.12	-1.17637
3.13	-1.13151
3.14	-1.08866
3.15	-1.04775
3.16	-1.0087
3.17	-0.97145
3.18	-0.93595
3.19	-0.90211
3.2	-0.86989
3.21	-0.83923
3.22	-0.81006
3.23	-0.78233
3.24	-0.75599
3.25	-0.73097
3.26	-0.70724
3.27	-0.68473
3.28	-0.6634
3.29	-0.64321
3.3	-0.6241
3.31	-0.60604
3.32	-0.58897
3.33	-0.57287
3.34	-0.55768
3.35	-0.54337
3.36	-0.5299
3.37	-0.51724
3.38	-0.50535
3.39	-0.4942
3.4	-0.48375

3.41	-0.47398
3.42	-0.46486
3.43	-0.45635
3.44	-0.44843
3.45	-0.44107
3.46	-0.43424
3.47	-0.42793
3.48	-0.4221
3.49	-0.41674
3.5	-0.41182
3.51	-0.40732
3.52	-0.40323
3.53	-0.39951
3.54	-0.39616
3.55	-0.39315
3.56	-0.39047
3.57	-0.3881
3.58	-0.38602
3.59	-0.38423
3.6	-0.3827
3.61	-0.38142
3.62	-0.38038
3.63	-0.37956
3.64	-0.37895
3.65	-0.37855
3.66	-0.37833
3.67	-0.37829
3.68	-0.37842
3.69	-0.3787
3.7	-0.37913
3.71	-0.3797
3.72	-0.3804
3.73	-0.38122
3.74	-0.38215
3.75	-0.38319
3.76	-0.38432
3.77	-0.38555
3.78	-0.38685
3.79	-0.38824
3.8	-0.38969
3.81	-0.39121
3.82	-0.39279
3.83	-0.39442
3.84	-0.3961
3.85	-0.39782
3.86	-0.39958
3.87	-0.40137
3.88	-0.40319

3.89	-0.40504
3.9	-0.40691
3.91	-0.40879
3.92	-0.41069
3.93	-0.41259
3.94	-0.41451
3.95	-0.41642
3.96	-0.41834
3.97	-0.42025
3.98	-0.42216
3.99	-0.42406
4	-0.42595
4.01	-0.42782
4.02	-0.42969
4.03	-0.43153
4.04	-0.43335
4.05	-0.43515
4.06	-0.43693
4.07	-0.43868
4.08	-0.44041
4.09	-0.4421
4.1	-0.44377
4.11	-0.44541
4.12	-0.44701
4.13	-0.44858
4.14	-0.45011
4.15	-0.45161
4.16	-0.45307
4.17	-0.45449
4.18	-0.45587
4.19	-0.45721
4.2	-0.45851
4.21	-0.45977
4.22	-0.46099
4.23	-0.46216
4.24	-0.46329
4.25	-0.46437
4.26	-0.46541
4.27	-0.46641
4.28	-0.46735
4.29	-0.46826
4.3	-0.46911
4.31	-0.46992
4.32	-0.47068
4.33	-0.47139
4.34	-0.47206
4.35	-0.47268
4.36	-0.47324

4.37	-0.47377
4.38	-0.47424
4.39	-0.47466
4.4	-0.47504
4.41	-0.47537
4.42	-0.47565
4.43	-0.47588
4.44	-0.47606
4.45	-0.47619
4.46	-0.47628
4.47	-0.47631
4.48	-0.4763
4.49	-0.47624
4.5	-0.47614
4.51	-0.47598
4.52	-0.47578
4.53	-0.47553
4.54	-0.47524
4.55	-0.47489
4.56	-0.4745
4.57	-0.47407
4.58	-0.47359
4.59	-0.47306
4.6	-0.47249
4.61	-0.47187
4.62	-0.47121
4.63	-0.4705
4.64	-0.46975
4.65	-0.46895
4.66	-0.46812
4.67	-0.46723
4.68	-0.46631
4.69	-0.46534
4.7	-0.46434
4.71	-0.46329
4.72	-0.4622
4.73	-0.46107
4.74	-0.45989
4.75	-0.45868
4.76	-0.45743
4.77	-0.45614
4.78	-0.45481
4.79	-0.45345
4.8	-0.45204
4.81	-0.4506
4.82	-0.44912
4.83	-0.44761
4.84	-0.44606

4.85	-0.44448
4.86	-0.44285
4.87	-0.4412
4.88	-0.43951
4.89	-0.43779
4.9	-0.43604
4.91	-0.43425
4.92	-0.43243
4.93	-0.43058
4.94	-0.4287
4.95	-0.42678
4.96	-0.42484
4.97	-0.42287
4.98	-0.42087
4.99	-0.41884
5	-0.41679
5.01	-0.4147
5.02	-0.41259
5.03	-0.41045
5.04	-0.40829
5.05	-0.4061
5.06	-0.40389
5.07	-0.40165
5.08	-0.39938
5.09	-0.3971
5.1	-0.39479
5.11	-0.39246
5.12	-0.39011
5.13	-0.38773
5.14	-0.38534
5.15	-0.38292
5.16	-0.38049
5.17	-0.37804
5.18	-0.37556
5.19	-0.37307
5.2	-0.37056
5.21	-0.36804
5.22	-0.36549
5.23	-0.36294
5.24	-0.36036
5.25	-0.35777
5.26	-0.35517
5.27	-0.35255
5.28	-0.34992
5.29	-0.34727
5.3	-0.34461
5.31	-0.34194
5.32	-0.33926

5.33	-0.33657
5.34	-0.33386
5.35	-0.33115
5.36	-0.32843
5.37	-0.3257
5.38	-0.32295
5.39	-0.32021
5.4	-0.31745
5.41	-0.31468
5.42	-0.31191
5.43	-0.30914
5.44	-0.30635
5.45	-0.30357
5.46	-0.30077
5.47	-0.29798
5.48	-0.29518
5.49	-0.29237
5.5	-0.28957
5.51	-0.28676
5.52	-0.28395
5.53	-0.28113
5.54	-0.27832
5.55	-0.27551
5.56	-0.27269
5.57	-0.26988
5.58	-0.26707
5.59	-0.26425
5.6	-0.26144
5.61	-0.25864
5.62	-0.25583
5.63	-0.25303
5.64	-0.25023
5.65	-0.24744
5.66	-0.24465
5.67	-0.24186
5.68	-0.23909
5.69	-0.23631
5.7	-0.23354
5.71	-0.23078
5.72	-0.22803
5.73	-0.22528
5.74	-0.22255
5.75	-0.21982
5.76	-0.2171
5.77	-0.21438
5.78	-0.21168
5.79	-0.20899
5.8	-0.20631

5.81	-0.20363
5.82	-0.20097
5.83	-0.19833
5.84	-0.19569
5.85	-0.19306
5.86	-0.19045
5.87	-0.18785
5.88	-0.18527
5.89	-0.1827
5.9	-0.18014
5.91	-0.1776
5.92	-0.17507
5.93	-0.17256
5.94	-0.17006
5.95	-0.16758
5.96	-0.16512
5.97	-0.16267
5.98	-0.16024
5.99	-0.15783
6	-0.15543
6.01	-0.15306
6.02	-0.1507
6.03	-0.14836
6.04	-0.14604
6.05	-0.14374
6.06	-0.14146
6.07	-0.1392
6.08	-0.13696
6.09	-0.13474
6.1	-0.13254
6.11	-0.13036
6.12	-0.1282
6.13	-0.12607
6.14	-0.12396
6.15	-0.12187
6.16	-0.1198
6.17	-0.11776
6.18	-0.11574
6.19	-0.11375
6.2	-0.11177
6.21	-0.10983
6.22	-0.1079
6.23	-0.10601
6.24	-0.10413
6.25	-0.10228
6.26	-0.10046
6.27	-0.09866
6.28	-0.09689

6.29	-0.09515
6.3	-0.09343
6.31	-0.09174
6.32	-0.09007
6.33	-0.08844
6.34	-0.08683
6.35	-0.08524
6.36	-0.08369
6.37	-0.08216
6.38	-0.08067
6.39	-0.0792
6.4	-0.07775
6.41	-0.07634
6.42	-0.07496
6.43	-0.0736
6.44	-0.07228
6.45	-0.07098
6.46	-0.06972
6.47	-0.06848
6.48	-0.06728
6.49	-0.0661
6.5	-0.06496
6.51	-0.06384
6.52	-0.06276
6.53	-0.0617
6.54	-0.06068
6.55	-0.05969
6.56	-0.05873
6.57	-0.0578
6.58	-0.05691
6.59	-0.05604
6.6	-0.05521
6.61	-0.05441
6.62	-0.05364
6.63	-0.0529
6.64	-0.05219
6.65	-0.05152
6.66	-0.05088
6.67	-0.05027
6.68	-0.04969
6.69	-0.04915
6.7	-0.04864
6.71	-0.04816
6.72	-0.04771
6.73	-0.0473
6.74	-0.04692
6.75	-0.04657
6.76	-0.04626

6.77	-0.04597
6.78	-0.04573
6.79	-0.04551
6.8	-0.04533
6.81	-0.04518
6.82	-0.04506
6.83	-0.04498
6.84	-0.04493
6.85	-0.04491
6.86	-0.04493
6.87	-0.04498
6.88	-0.04506
6.89	-0.04518
6.9	-0.04533
6.91	-0.04551
6.92	-0.04573
6.93	-0.04597
6.94	-0.04626
6.95	-0.04657
6.96	-0.04692
6.97	-0.0473
6.98	-0.04771
6.99	-0.04816
7	-0.04864
7.01	-0.04915
7.02	-0.04969
7.03	-0.05027
7.04	-0.05088
7.05	-0.05152
7.06	-0.05219
7.07	-0.0529
7.08	-0.05364
7.09	-0.05441
7.1	-0.05521
7.11	-0.05604
7.12	-0.05691
7.13	-0.0578
7.14	-0.05873
7.15	-0.05969
7.16	-0.06068
7.17	-0.0617
7.18	-0.06276
7.19	-0.06384
7.2	-0.06496
7.21	-0.0661
7.22	-0.06728
7.23	-0.06848
7.24	-0.06972

7.25	-0.07098
7.26	-0.07228
7.27	-0.0736
7.28	-0.07496
7.29	-0.07634
7.3	-0.07775
7.31	-0.0792
7.32	-0.08067
7.33	-0.08216
7.34	-0.08369
7.35	-0.08524
7.36	-0.08683
7.37	-0.08844
7.38	-0.09007
7.39	-0.09174
7.4	-0.09343
7.41	-0.09515
7.42	-0.09689
7.43	-0.09866
7.44	-0.10046
7.45	-0.10228
7.46	-0.10413
7.47	-0.10601
7.48	-0.1079
7.49	-0.10983
7.5	-0.11177
7.51	-0.11375
7.52	-0.11574
7.53	-0.11776
7.54	-0.1198
7.55	-0.12187
7.56	-0.12396
7.57	-0.12607
7.58	-0.1282
7.59	-0.13036
7.6	-0.13254
7.61	-0.13474
7.62	-0.13696
7.63	-0.1392
7.64	-0.14146
7.65	-0.14374
7.66	-0.14604
7.67	-0.14836
7.68	-0.1507
7.69	-0.15306
7.7	-0.15543
7.71	-0.15783
7.72	-0.16024

7.73	-0.16267
7.74	-0.16512
7.75	-0.16758
7.76	-0.17006
7.77	-0.17256
7.78	-0.17507
7.79	-0.1776
7.8	-0.18014
7.81	-0.1827
7.82	-0.18527
7.83	-0.18785
7.84	-0.19045
7.85	-0.19306
7.86	-0.19569
7.87	-0.19833
7.88	-0.20097
7.89	-0.20363
7.9	-0.20631
7.91	-0.20899
7.92	-0.21168
7.93	-0.21438
7.94	-0.2171
7.95	-0.21982
7.96	-0.22255
7.97	-0.22528
7.98	-0.22803
7.99	-0.23078
8	-0.23354
8.01	-0.23631
8.02	-0.23909
8.03	-0.24186
8.04	-0.24465
8.05	-0.24744
8.06	-0.25023
8.07	-0.25303
8.08	-0.25583
8.09	-0.25864
8.1	-0.26144
8.11	-0.26425
8.12	-0.26707
8.13	-0.26988
8.14	-0.27269
8.15	-0.27551
8.16	-0.27832
8.17	-0.28113
8.18	-0.28395
8.19	-0.28676
8.2	-0.28957

8.21	-0.29237
8.22	-0.29518
8.23	-0.29798
8.24	-0.30077
8.25	-0.30357
8.26	-0.30635
8.27	-0.30914
8.28	-0.31191
8.29	-0.31468
8.3	-0.31745
8.31	-0.32021
8.32	-0.32295
8.33	-0.3257
8.34	-0.32843
8.35	-0.33115
8.36	-0.33386
8.37	-0.33657
8.38	-0.33926
8.39	-0.34194
8.4	-0.34461
8.41	-0.34727
8.42	-0.34992
8.43	-0.35255
8.44	-0.35517
8.45	-0.35777
8.46	-0.36036
8.47	-0.36294
8.48	-0.36549
8.49	-0.36804
8.5	-0.37056
8.51	-0.37307
8.52	-0.37556
8.53	-0.37804
8.54	-0.38049
8.55	-0.38292
8.56	-0.38534
8.57	-0.38773
8.58	-0.39011
8.59	-0.39246
8.6	-0.39479
8.61	-0.3971
8.62	-0.39938
8.63	-0.40165
8.64	-0.40389
8.65	-0.4061
8.66	-0.40829
8.67	-0.41045
8.68	-0.41259

8.69	-0.4147
8.7	-0.41679
8.71	-0.41884
8.72	-0.42087
8.73	-0.42287
8.74	-0.42484
8.75	-0.42678
8.76	-0.4287
8.77	-0.43058
8.78	-0.43243
8.79	-0.43425
8.8	-0.43604
8.81	-0.43779
8.82	-0.43951
8.83	-0.4412
8.84	-0.44285
8.85	-0.44448
8.86	-0.44606
8.87	-0.44761
8.88	-0.44912
8.89	-0.4506
8.9	-0.45204
8.91	-0.45345
8.92	-0.45481
8.93	-0.45614
8.94	-0.45743
8.95	-0.45868
8.96	-0.45989
8.97	-0.46107
8.98	-0.4622
8.99	-0.46329
9	-0.46434
9.01	-0.46534
9.02	-0.46631
9.03	-0.46723
9.04	-0.46812
9.05	-0.46895
9.06	-0.46975
9.07	-0.4705
9.08	-0.47121
9.09	-0.47187
9.1	-0.47249
9.11	-0.47306
9.12	-0.47359
9.13	-0.47407
9.14	-0.4745
9.15	-0.47489
9.16	-0.47524

9.17	-0.47553
9.18	-0.47578
9.19	-0.47598
9.2	-0.47614
9.21	-0.47624
9.22	-0.4763
9.23	-0.47631
9.24	-0.47628
9.25	-0.47619
9.26	-0.47606
9.27	-0.47588
9.28	-0.47565
9.29	-0.47537
9.3	-0.47504
9.31	-0.47466
9.32	-0.47424
9.33	-0.47377
9.34	-0.47324
9.35	-0.47268
9.36	-0.47206
9.37	-0.47139
9.38	-0.47068
9.39	-0.46992
9.4	-0.46911
9.41	-0.46826
9.42	-0.46735
9.43	-0.46641
9.44	-0.46541
9.45	-0.46437
9.46	-0.46329
9.47	-0.46216
9.48	-0.46099
9.49	-0.45977
9.5	-0.45851
9.51	-0.45721
9.52	-0.45587
9.53	-0.45449
9.54	-0.45307
9.55	-0.45161
9.56	-0.45011
9.57	-0.44858
9.58	-0.44701
9.59	-0.44541
9.6	-0.44377
9.61	-0.4421
9.62	-0.44041
9.63	-0.43868
9.64	-0.43693

9.65	-0.43515
9.66	-0.43335
9.67	-0.43153
9.68	-0.42969
9.69	-0.42782
9.7	-0.42595
9.71	-0.42406
9.72	-0.42216
9.73	-0.42025
9.74	-0.41834
9.75	-0.41642
9.76	-0.41451
9.77	-0.41259
9.78	-0.41069
9.79	-0.40879
9.8	-0.40691
9.81	-0.40504
9.82	-0.40319
9.83	-0.40137
9.84	-0.39958
9.85	-0.39782
9.86	-0.3961
9.87	-0.39442
9.88	-0.39279
9.89	-0.39121
9.9	-0.38969
9.91	-0.38824
9.92	-0.38685
9.93	-0.38555
9.94	-0.38432
9.95	-0.38319
9.96	-0.38215
9.97	-0.38122
9.98	-0.3804
9.99	-0.3797
10	-0.37913
10.01	-0.3787
10.02	-0.37842
10.03	-0.37829
10.04	-0.37833
10.05	-0.37855
10.06	-0.37895
10.07	-0.37956
10.08	-0.38038
10.09	-0.38142
10.1	-0.3827
10.11	-0.38423
10.12	-0.38602

10.13	-0.3881
10.14	-0.39047
10.15	-0.39315
10.16	-0.39616
10.17	-0.39951
10.18	-0.40323
10.19	-0.40732
10.2	-0.41182
10.21	-0.41674
10.22	-0.4221
10.23	-0.42793
10.24	-0.43424
10.25	-0.44107
10.26	-0.44843
10.27	-0.45635
10.28	-0.46486
10.29	-0.47398
10.3	-0.48375
10.31	-0.4942
10.32	-0.50535
10.33	-0.51724
10.34	-0.5299
10.35	-0.54337
10.36	-0.55768
10.37	-0.57287
10.38	-0.58897
10.39	-0.60604
10.4	-0.6241
10.41	-0.64321
10.42	-0.6634
10.43	-0.68473
10.44	-0.70724
10.45	-0.73097
10.46	-0.75599
10.47	-0.78233
10.48	-0.81006
10.49	-0.83923
10.5	-0.86989
10.51	-0.90211
10.52	-0.93595
10.53	-0.97145
10.54	-1.0087
10.55	-1.04775
10.56	-1.08866
10.57	-1.13151
10.58	-1.17637
10.59	-1.2233
10.6	-1.27238

10.61	-1.32368
10.62	-1.37727
10.63	-1.43323
10.64	-1.49163
10.65	-1.55256
10.66	-1.61608
10.67	-1.68229
10.68	-1.75126
10.69	-1.82306
10.7	-1.89778
10.71	-1.97551
10.72	-2.05632
10.73	-2.14029
10.74	-2.2275
10.75	-2.31804
10.76	-2.41199
10.77	-2.50942
10.78	-2.61042
10.79	-2.71507
10.8	-2.82344
10.81	-2.9356
10.82	-3.05165
10.83	-3.17165
10.84	-3.29567
10.85	-3.42379
10.86	-3.55609
10.87	-3.69262
10.88	-3.83346
10.89	-3.97868
10.9	-4.12835
10.91	-4.28252
10.92	-4.44127
10.93	-4.60465
10.94	-4.77273
10.95	-4.94557
10.96	-5.12324
10.97	-5.30578
10.98	-5.49327
10.99	-5.68576
11	-5.88332
11.01	-6.086
11.02	-6.29388
11.03	-6.50702
11.04	-6.72548
11.05	-6.94933
11.06	-7.17866
11.07	-7.41353
11.08	-7.65403

11.09	-7.90024
11.1	-8.15226
11.11	-8.41018
11.12	-8.67412
11.13	-8.94417
11.14	-9.22048
11.15	-9.50316
11.16	-9.79236
11.17	-10.0882
11.18	-10.391
11.19	-10.7007
11.2	-11.0177
11.21	-11.3422
11.22	-11.6744
11.23	-12.0145
11.24	-12.3629
11.25	-12.7198
11.26	-13.0857
11.27	-13.4609
11.28	-13.8458
11.29	-14.2409
11.3	-14.6468
11.31	-15.0639
11.32	-15.4931
11.33	-15.9349
11.34	-16.3902
11.35	-16.8599
11.36	-17.3451
11.37	-17.8469
11.38	-18.3666
11.39	-18.9058
11.4	-19.4662
11.41	-20.0498
11.42	-20.6591
11.43	-21.2967
11.44	-21.966
11.45	-22.6709
11.46	-23.4162
11.47	-24.2077
11.48	-25.0527
11.49	-25.9605
11.5	-26.9428
11.51	-28.0154
11.52	-29.1995
11.53	-30.5253
11.54	-32.0369
11.55	-33.8035
11.56	-35.942

11.57	-38.6751
11.58	-42.5163
11.59	-49.2095
11.6	-67.5666
11.61	-47.6101
11.62	-42.1858
11.63	-38.9731
11.64	-36.7095
11.65	-34.9768
11.66	-33.5841
11.67	-32.4281
11.68	-31.4464
11.69	-30.5985
11.7	-29.8566
11.71	-29.2009
11.72	-28.6166
11.73	-28.0924
11.74	-27.6196
11.75	-27.1912
11.76	-26.8016
11.77	-26.4462
11.78	-26.1212
11.79	-25.8234
11.8	-25.55
11.81	-25.2988
11.82	-25.0678
11.83	-24.8551
11.84	-24.6594
11.85	-24.4792
11.86	-24.3135
11.87	-24.1611
11.88	-24.0211
11.89	-23.8928
11.9	-23.7753
11.91	-23.6679
11.92	-23.5702
11.93	-23.4814
11.94	-23.4012
11.95	-23.329
11.96	-23.2644
11.97	-23.2071
11.98	-23.1567
11.99	-23.1128
12	-23.0752
12.01	-23.0436
12.02	-23.0176
12.03	-22.9972
12.04	-22.982

12.05	-22.9718
12.06	-22.9665
12.07	-22.9658
12.08	-22.9696
12.09	-22.9778
12.1	-22.9901
12.11	-23.0065
12.12	-23.0268
12.13	-23.0508
12.14	-23.0786
12.15	-23.1098
12.16	-23.1446
12.17	-23.1827
12.18	-23.2241
12.19	-23.2686
12.2	-23.3163
12.21	-23.367
12.22	-23.4206
12.23	-23.4771
12.24	-23.5364
12.25	-23.5985
12.26	-23.6633
12.27	-23.7307
12.28	-23.8008
12.29	-23.8733
12.3	-23.9484
12.31	-24.0259
12.32	-24.1058
12.33	-24.1882
12.34	-24.2728
12.35	-24.3598
12.36	-24.449
12.37	-24.5405
12.38	-24.6343
12.39	-24.7302
12.4	-24.8283
12.41	-24.9285
12.42	-25.0309
12.43	-25.1354
12.44	-25.242
12.45	-25.3507
12.46	-25.4615
12.47	-25.5743
12.48	-25.6891
12.49	-25.806
12.5	-25.9249
12.51	-26.0459
12.52	-26.1689

12.53	-26.2939
12.54	-26.4209
12.55	-26.5499
12.56	-26.6809
12.57	-26.814
12.58	-26.949
12.59	-27.0861
12.6	-27.2252
12.61	-27.3664
12.62	-27.5096
12.63	-27.6549
12.64	-27.8022
12.65	-27.9516
12.66	-28.1031
12.67	-28.2567
12.68	-28.4124
12.69	-28.5703
12.7	-28.7303
12.71	-28.8925
12.72	-29.0569
12.73	-29.2235
12.74	-29.3924
12.75	-29.5635
12.76	-29.737
12.77	-29.9128
12.78	-30.0909
12.79	-30.2715
12.8	-30.4545
12.81	-30.6399
12.82	-30.8279
12.83	-31.0184
12.84	-31.2115
12.85	-31.4073
12.86	-31.6057
12.87	-31.8068
12.88	-32.0108
12.89	-32.2175
12.9	-32.4272
12.91	-32.6398
12.92	-32.8554
12.93	-33.0742
12.94	-33.296
12.95	-33.5211
12.96	-33.7494
12.97	-33.9811
12.98	-34.2163
12.99	-34.455
13	-34.6973

13.01	-34.9434
13.02	-35.1932
13.03	-35.447
13.04	-35.7048
13.05	-35.9667
13.06	-36.2329
13.07	-36.5035
13.08	-36.7785
13.09	-37.0582
13.1	-37.3428
13.11	-37.6322
13.12	-37.9268
13.13	-38.2266
13.14	-38.5319
13.15	-38.8428
13.16	-39.1596
13.17	-39.4825
13.18	-39.8116
13.19	-40.1472
13.2	-40.4896
13.21	-40.839
13.22	-41.1957
13.23	-41.5601
13.24	-41.9324
13.25	-42.313
13.26	-42.7022
13.27	-43.1004
13.28	-43.5081
13.29	-43.9258
13.3	-44.3538
13.31	-44.7927
13.32	-45.2431
13.33	-45.7056
13.34	-46.1808
13.35	-46.6695
13.36	-47.1724
13.37	-47.6904
13.38	-48.2244
13.39	-48.7754
13.4	-49.3445
13.41	-49.9329
13.42	-50.5421
13.43	-51.1734
13.44	-51.8287
13.45	-52.5096
13.46	-53.2185
13.47	-53.9575
13.48	-54.7294

13.49	-55.5373
13.5	-56.3846
13.51	-57.2754
13.52	-58.2145
13.53	-59.2072
13.54	-60.2602
13.55	-61.3812
13.56	-62.5795
13.57	-63.8668
13.58	-65.2572
13.59	-66.7686
13.6	-68.4242
13.61	-70.2544
13.62	-72.3004
13.63	-74.6201
13.64	-77.2979
13.65	-80.4651
13.75	-80.4651
13.76	-77.2979
13.77	-74.6201
13.78	-72.3004
13.79	-70.2544
13.8	-68.4242
13.81	-66.7686
13.82	-65.2572
13.83	-63.8668
13.84	-62.5795
13.85	-61.3812
13.86	-60.2602
13.87	-59.2072
13.88	-58.2145
13.89	-57.2754
13.9	-56.3846
13.91	-55.5373
13.92	-54.7294
13.93	-53.9575
13.94	-53.2185
13.95	-52.5096
13.96	-51.8287
13.97	-51.1734
13.98	-50.5421
13.99	-49.9329
14	-49.3445
14.01	-48.7754
14.02	-48.2244
14.03	-47.6904
14.04	-47.1724
14.05	-46.6695

14.06	-46.1808
14.07	-45.7056
14.08	-45.2431
14.09	-44.7927
14.1	-44.3538
14.11	-43.9258
14.12	-43.5081
14.13	-43.1004
14.14	-42.7022
14.15	-42.313
14.16	-41.9324
14.17	-41.5601
14.18	-41.1957
14.19	-40.839
14.2	-40.4896
14.21	-40.1472
14.22	-39.8116
14.23	-39.4825
14.24	-39.1596
14.25	-38.8428
14.26	-38.5319
14.27	-38.2266
14.28	-37.9268
14.29	-37.6322
14.3	-37.3428
14.31	-37.0582
14.32	-36.7785
14.33	-36.5035
14.34	-36.2329
14.35	-35.9667
14.36	-35.7048
14.37	-35.447
14.38	-35.1932
14.39	-34.9434
14.4	-34.6973
14.41	-34.455
14.42	-34.2163
14.43	-33.9811
14.44	-33.7494
14.45	-33.5211
14.46	-33.296
14.47	-33.0742
14.48	-32.8554
14.49	-32.6398
14.5	-32.4272
14.51	-32.2175
14.52	-32.0108
14.53	-31.8068

14.54	-31.6057
14.55	-31.4073
14.56	-31.2115
14.57	-31.0184
14.58	-30.8279
14.59	-30.6399
14.6	-30.4545
14.61	-30.2715
14.62	-30.0909
14.63	-29.9128
14.64	-29.737
14.65	-29.5635
14.66	-29.3924
14.67	-29.2235
14.68	-29.0569
14.69	-28.8925
14.7	-28.7303
14.71	-28.5703
14.72	-28.4124
14.73	-28.2567
14.74	-28.1031
14.75	-27.9516
14.76	-27.8022
14.77	-27.6549
14.78	-27.5096
14.79	-27.3664
14.8	-27.2252
14.81	-27.0861
14.82	-26.949
14.83	-26.814
14.84	-26.6809
14.85	-26.5499
14.86	-26.4209
14.87	-26.2939
14.88	-26.1689
14.89	-26.0459
14.9	-25.9249
14.91	-25.806
14.92	-25.6891
14.93	-25.5743
14.94	-25.4615
14.95	-25.3507
14.96	-25.242
14.97	-25.1354
14.98	-25.0309
14.99	-24.9285
15	-24.8283
15.01	-24.7302

15.02	-24.6343
15.03	-24.5405
15.04	-24.449
15.05	-24.3598
15.06	-24.2728
15.07	-24.1882
15.08	-24.1058
15.09	-24.0259
15.1	-23.9484
15.11	-23.8733
15.12	-23.8008
15.13	-23.7307
15.14	-23.6633
15.15	-23.5985
15.16	-23.5364
15.17	-23.4771
15.18	-23.4206
15.19	-23.367
15.2	-23.3163
15.21	-23.2686
15.22	-23.2241
15.23	-23.1827
15.24	-23.1446
15.25	-23.1098
15.26	-23.0786
15.27	-23.0508
15.28	-23.0268
15.29	-23.0065
15.3	-22.9901
15.31	-22.9778
15.32	-22.9696
15.33	-22.9658
15.34	-22.9665
15.35	-22.9718
15.36	-22.982
15.37	-22.9972
15.38	-23.0176
15.39	-23.0436
15.4	-23.0752
15.41	-23.1128
15.42	-23.1567
15.43	-23.2071
15.44	-23.2644
15.45	-23.329
15.46	-23.4012
15.47	-23.4814
15.48	-23.5702
15.49	-23.6679

15.5	-23.7753
15.51	-23.8928
15.52	-24.0211
15.53	-24.1611
15.54	-24.3135
15.55	-24.4792
15.56	-24.6594
15.57	-24.8551
15.58	-25.0678
15.59	-25.2988
15.6	-25.55
15.61	-25.8234
15.62	-26.1212
15.63	-26.4462
15.64	-26.8016
15.65	-27.1912
15.66	-27.6196
15.67	-28.0924
15.68	-28.6166
15.69	-29.2009
15.7	-29.8566
15.71	-30.5985
15.72	-31.4464
15.73	-32.4281
15.74	-33.5841
15.75	-34.9768
15.76	-36.7095
15.77	-38.9731
15.78	-42.1858
15.79	-47.6101
15.8	-67.5666
15.81	-49.2095
15.82	-42.5163
15.83	-38.6751
15.84	-35.942
15.85	-33.8035
15.86	-32.0369
15.87	-30.5253
15.88	-29.1995
15.89	-28.0154
15.9	-26.9428
15.91	-25.9605
15.92	-25.0527
15.93	-24.2077
15.94	-23.4162
15.95	-22.6709
15.96	-21.966
15.97	-21.2967

15.98	-20.6591
15.99	-20.0498
16	-19.4662

one balun_80 ohms Data File

Frequency (	DB[S11]
0	-0.01042
0.01	-6.92E-11
0.02	-1.11E-09
0.03	-5.60E-09
0.04	-1.77E-08
0.05	-4.32E-08
0.06	-8.96E-08
0.07	-1.66E-07
0.08	-2.83E-07
0.09	-4.54E-07
0.1	-6.91E-07
0.11	-1.01E-06
0.12	-1.43E-06
0.13	-1.97E-06
0.14	-2.65E-06
0.15	-3.50E-06
0.16	-4.53E-06
0.17	-5.77E-06
0.18	-7.25E-06
0.19	-9.00E-06
0.2	-1.10E-05
0.21	-1.34E-05
0.22	-1.62E-05
0.23	-1.93E-05
0.24	-2.29E-05
0.25	-2.69E-05
0.26	-3.15E-05
0.27	-3.66E-05
0.28	-4.24E-05
0.29	-4.87E-05
0.3	-5.58E-05
0.31	-6.36E-05
0.32	-7.22E-05
0.33	-8.16E-05
0.34	-9.19E-05
0.35	-0.0001
0.36	-0.00012
0.37	-0.00013
0.38	-0.00014
0.39	-0.00016
0.4	-0.00018
0.41	-0.00019

0.42	-0.00021
0.43	-0.00023
0.44	-0.00026
0.45	-0.00028
0.46	-0.00031
0.47	-0.00033
0.48	-0.00036
0.49	-0.00039
0.5	-0.00043
0.51	-0.00046
0.52	-0.0005
0.53	-0.00054
0.54	-0.00058
0.55	-0.00062
0.56	-0.00067
0.57	-0.00072
0.58	-0.00077
0.59	-0.00082
0.6	-0.00088
0.61	-0.00094
0.62	-0.001
0.63	-0.00106
0.64	-0.00113
0.65	-0.0012
0.66	-0.00128
0.67	-0.00135
0.68	-0.00143
0.69	-0.00152
0.7	-0.00161
0.71	-0.0017
0.72	-0.00179
0.73	-0.00189
0.74	-0.002
0.75	-0.0021
0.76	-0.00221
0.77	-0.00233
0.78	-0.00245
0.79	-0.00257
0.8	-0.0027
0.81	-0.00283
0.82	-0.00297
0.83	-0.00311
0.84	-0.00326
0.85	-0.00341
0.86	-0.00356
0.87	-0.00372
0.88	-0.00389
0.89	-0.00406

0.9	-0.00424
0.91	-0.00442
0.92	-0.0046
0.93	-0.00479
0.94	-0.00499
0.95	-0.00519
0.96	-0.0054
0.97	-0.00561
0.98	-0.00583
0.99	-0.00605
1	-0.00628
1.01	-0.00652
1.02	-0.00676
1.03	-0.007
1.04	-0.00725
1.05	-0.00751
1.06	-0.00777
1.07	-0.00804
1.08	-0.00831
1.09	-0.00859
1.1	-0.00887
1.11	-0.00916
1.12	-0.00945
1.13	-0.00975
1.14	-0.01005
1.15	-0.01036
1.16	-0.01067
1.17	-0.01099
1.18	-0.01131
1.19	-0.01164
1.2	-0.01197
1.21	-0.0123
1.22	-0.01264
1.23	-0.01298
1.24	-0.01333
1.25	-0.01368
1.26	-0.01403
1.27	-0.01439
1.28	-0.01474
1.29	-0.0151
1.3	-0.01546
1.31	-0.01583
1.32	-0.01619
1.33	-0.01656
1.34	-0.01692
1.35	-0.01729
1.36	-0.01766
1.37	-0.01802

1.38	-0.01839
1.39	-0.01875
1.4	-0.01911
1.41	-0.01947
1.42	-0.01983
1.43	-0.02018
1.44	-0.02053
1.45	-0.02088
1.46	-0.02122
1.47	-0.02155
1.48	-0.02188
1.49	-0.0222
1.5	-0.02251
1.51	-0.02282
1.52	-0.02311
1.53	-0.0234
1.54	-0.02367
1.55	-0.02394
1.56	-0.02419
1.57	-0.02443
1.58	-0.02465
1.59	-0.02487
1.6	-0.02506
1.61	-0.02524
1.62	-0.02541
1.63	-0.02555
1.64	-0.02568
1.65	-0.02579
1.66	-0.02587
1.67	-0.02594
1.68	-0.02599
1.69	-0.02601
1.7	-0.026
1.71	-0.02598
1.72	-0.02593
1.73	-0.02585
1.74	-0.02574
1.75	-0.02561
1.76	-0.02545
1.77	-0.02526
1.78	-0.02504
1.79	-0.02479
1.8	-0.02451
1.81	-0.02419
1.82	-0.02385
1.83	-0.02347
1.84	-0.02306
1.85	-0.02262

1.86	-0.02215
1.87	-0.02164
1.88	-0.0211
1.89	-0.02053
1.9	-0.01993
1.91	-0.0193
1.92	-0.01864
1.93	-0.01794
1.94	-0.01722
1.95	-0.01648
1.96	-0.01571
1.97	-0.01491
1.98	-0.0141
1.99	-0.01326
2	-0.01241
2.01	-0.01155
2.02	-0.01068
2.03	-0.00981
2.04	-0.00893
2.05	-0.00805
2.06	-0.00719
2.07	-0.00633
2.08	-0.0055
2.09	-0.0047
2.1	-0.00393
2.11	-0.00319
2.12	-0.00251
2.13	-0.00189
2.14	-0.00134
2.15	-0.00087
2.16	-0.00048
2.17	-0.0002
2.18	-3.85E-05
2.19	-4.49E-06
2.2	-0.00012
2.21	-0.00039
2.22	-0.00084
2.23	-0.00148
2.24	-0.00235
2.25	-0.00345
2.26	-0.0048
2.27	-0.00644
2.28	-0.00839
2.29	-0.01067
2.3	-0.01331
2.31	-0.01634
2.32	-0.01979
2.33	-0.02369

2.34	-0.02808
2.35	-0.033
2.36	-0.03847
2.37	-0.04455
2.38	-0.05127
2.39	-0.05868
2.4	-0.06682
2.41	-0.07574
2.42	-0.08549
2.43	-0.09613
2.44	-0.1077
2.45	-0.12026
2.46	-0.13388
2.47	-0.1486
2.48	-0.1645
2.49	-0.18163
2.5	-0.20007
2.51	-0.21988
2.52	-0.24113
2.53	-0.26389
2.54	-0.28823
2.55	-0.31423
2.56	-0.34196
2.57	-0.3715
2.58	-0.40293
2.59	-0.43631
2.6	-0.47174
2.61	-0.50928
2.62	-0.54901
2.63	-0.59101
2.64	-0.63536
2.65	-0.68211
2.66	-0.73136
2.67	-0.78316
2.68	-0.83759
2.69	-0.8947
2.7	-0.95456
2.71	-1.01722
2.72	-1.08274
2.73	-1.15116
2.74	-1.22252
2.75	-1.29686
2.76	-1.37421
2.77	-1.4546
2.78	-1.53804
2.79	-1.62455
2.8	-1.71412
2.81	-1.80676

2.82	-1.90246
2.83	-2.00119
2.84	-2.10293
2.85	-2.20765
2.86	-2.3153
2.87	-2.42584
2.88	-2.53921
2.89	-2.65535
2.9	-2.77417
2.91	-2.89562
2.92	-3.01959
2.93	-3.146
2.94	-3.27474
2.95	-3.40572
2.96	-3.53881
2.97	-3.67391
2.98	-3.81089
2.99	-3.94962
3	-4.08996
3.01	-4.2318
3.02	-4.37497
3.03	-4.51935
3.04	-4.66478
3.05	-4.81111
3.06	-4.95819
3.07	-5.10587
3.08	-5.25398
3.09	-5.40238
3.1	-5.55089
3.11	-5.69937
3.12	-5.84765
3.13	-5.99557
3.14	-6.14297
3.15	-6.28969
3.16	-6.43557
3.17	-6.58046
3.18	-6.72419
3.19	-6.86663
3.2	-7.0076
3.21	-7.14698
3.22	-7.28461
3.23	-7.42036
3.24	-7.55409
3.25	-7.68567
3.26	-7.81497
3.27	-7.94187
3.28	-8.06626
3.29	-8.18804

3.3	-8.3071
3.31	-8.42334
3.32	-8.5367
3.33	-8.64707
3.34	-8.75441
3.35	-8.85863
3.36	-8.9597
3.37	-9.05757
3.38	-9.1522
3.39	-9.24357
3.4	-9.33165
3.41	-9.41645
3.42	-9.49795
3.43	-9.57616
3.44	-9.65111
3.45	-9.7228
3.46	-9.79129
3.47	-9.85659
3.48	-9.91876
3.49	-9.97784
3.5	-10.0339
3.51	-10.087
3.52	-10.1372
3.53	-10.1845
3.54	-10.2291
3.55	-10.271
3.56	-10.3104
3.57	-10.3472
3.58	-10.3816
3.59	-10.4136
3.6	-10.4434
3.61	-10.471
3.62	-10.4965
3.63	-10.52
3.64	-10.5417
3.65	-10.5615
3.66	-10.5796
3.67	-10.596
3.68	-10.6109
3.69	-10.6242
3.7	-10.6362
3.71	-10.6469
3.72	-10.6563
3.73	-10.6645
3.74	-10.6716
3.75	-10.6777
3.76	-10.6828
3.77	-10.6871

3.78	-10.6905
3.79	-10.6931
3.8	-10.6949
3.81	-10.6961
3.82	-10.6967
3.83	-10.6968
3.84	-10.6963
3.85	-10.6954
3.86	-10.694
3.87	-10.6922
3.88	-10.6901
3.89	-10.6877
3.9	-10.6851
3.91	-10.6822
3.92	-10.6791
3.93	-10.6759
3.94	-10.6725
3.95	-10.669
3.96	-10.6654
3.97	-10.6617
3.98	-10.6581
3.99	-10.6544
4	-10.6507
4.01	-10.6471
4.02	-10.6435
4.03	-10.64
4.04	-10.6365
4.05	-10.6332
4.06	-10.63
4.07	-10.6269
4.08	-10.624
4.09	-10.6212
4.1	-10.6186
4.11	-10.6162
4.12	-10.6139
4.13	-10.6119
4.14	-10.6101
4.15	-10.6085
4.16	-10.6071
4.17	-10.6059
4.18	-10.605
4.19	-10.6044
4.2	-10.604
4.21	-10.6039
4.22	-10.604
4.23	-10.6044
4.24	-10.6051
4.25	-10.6061

4.26	-10.6073
4.27	-10.6089
4.28	-10.6107
4.29	-10.6129
4.3	-10.6153
4.31	-10.618
4.32	-10.6211
4.33	-10.6245
4.34	-10.6281
4.35	-10.6321
4.36	-10.6364
4.37	-10.6411
4.38	-10.646
4.39	-10.6513
4.4	-10.6569
4.41	-10.6628
4.42	-10.6691
4.43	-10.6757
4.44	-10.6826
4.45	-10.6898
4.46	-10.6974
4.47	-10.7053
4.48	-10.7136
4.49	-10.7222
4.5	-10.7311
4.51	-10.7403
4.52	-10.75
4.53	-10.7599
4.54	-10.7702
4.55	-10.7808
4.56	-10.7918
4.57	-10.8031
4.58	-10.8147
4.59	-10.8267
4.6	-10.8391
4.61	-10.8518
4.62	-10.8648
4.63	-10.8782
4.64	-10.8919
4.65	-10.906
4.66	-10.9204
4.67	-10.9352
4.68	-10.9503
4.69	-10.9658
4.7	-10.9816
4.71	-10.9978
4.72	-11.0144
4.73	-11.0312

4.74	-11.0485
4.75	-11.0661
4.76	-11.0841
4.77	-11.1024
4.78	-11.121
4.79	-11.1401
4.8	-11.1595
4.81	-11.1792
4.82	-11.1994
4.83	-11.2198
4.84	-11.2407
4.85	-11.2619
4.86	-11.2835
4.87	-11.3054
4.88	-11.3277
4.89	-11.3504
4.9	-11.3735
4.91	-11.3969
4.92	-11.4207
4.93	-11.4449
4.94	-11.4694
4.95	-11.4944
4.96	-11.5197
4.97	-11.5454
4.98	-11.5714
4.99	-11.5979
5	-11.6247
5.01	-11.652
5.02	-11.6796
5.03	-11.7076
5.04	-11.736
5.05	-11.7648
5.06	-11.794
5.07	-11.8236
5.08	-11.8536
5.09	-11.884
5.1	-11.9148
5.11	-11.9461
5.12	-11.9777
5.13	-12.0097
5.14	-12.0422
5.15	-12.0751
5.16	-12.1084
5.17	-12.1421
5.18	-12.1762
5.19	-12.2108
5.2	-12.2458
5.21	-12.2812

5.22	-12.3171
5.23	-12.3534
5.24	-12.3902
5.25	-12.4274
5.26	-12.465
5.27	-12.5032
5.28	-12.5417
5.29	-12.5807
5.3	-12.6202
5.31	-12.6602
5.32	-12.7006
5.33	-12.7415
5.34	-12.7829
5.35	-12.8248
5.36	-12.8671
5.37	-12.9099
5.38	-12.9533
5.39	-12.9971
5.4	-13.0415
5.41	-13.0863
5.42	-13.1317
5.43	-13.1776
5.44	-13.224
5.45	-13.2709
5.46	-13.3184
5.47	-13.3664
5.48	-13.4149
5.49	-13.464
5.5	-13.5137
5.51	-13.5639
5.52	-13.6147
5.53	-13.6661
5.54	-13.718
5.55	-13.7706
5.56	-13.8237
5.57	-13.8774
5.58	-13.9317
5.59	-13.9867
5.6	-14.0422
5.61	-14.0984
5.62	-14.1553
5.63	-14.2127
5.64	-14.2709
5.65	-14.3296
5.66	-14.3891
5.67	-14.4492
5.68	-14.51
5.69	-14.5715

5.7	-14.6338
5.71	-14.6967
5.72	-14.7603
5.73	-14.8247
5.74	-14.8898
5.75	-14.9557
5.76	-15.0223
5.77	-15.0897
5.78	-15.1579
5.79	-15.2269
5.8	-15.2967
5.81	-15.3673
5.82	-15.4388
5.83	-15.511
5.84	-15.5842
5.85	-15.6582
5.86	-15.7331
5.87	-15.8089
5.88	-15.8856
5.89	-15.9633
5.9	-16.0419
5.91	-16.1214
5.92	-16.2019
5.93	-16.2834
5.94	-16.366
5.95	-16.4495
5.96	-16.5341
5.97	-16.6198
5.98	-16.7065
5.99	-16.7943
6	-16.8833
6.01	-16.9734
6.02	-17.0646
6.03	-17.1571
6.04	-17.2507
6.05	-17.3456
6.06	-17.4418
6.07	-17.5392
6.08	-17.6379
6.09	-17.7379
6.1	-17.8393
6.11	-17.9421
6.12	-18.0463
6.13	-18.152
6.14	-18.2591
6.15	-18.3677
6.16	-18.4779
6.17	-18.5896

6.18	-18.7029
6.19	-18.8179
6.2	-18.9345
6.21	-19.0528
6.22	-19.1729
6.23	-19.2948
6.24	-19.4185
6.25	-19.5441
6.26	-19.6716
6.27	-19.801
6.28	-19.9325
6.29	-20.066
6.3	-20.2016
6.31	-20.3393
6.32	-20.4793
6.33	-20.6214
6.34	-20.7659
6.35	-20.9128
6.36	-21.062
6.37	-21.2137
6.38	-21.368
6.39	-21.5248
6.4	-21.6843
6.41	-21.8464
6.42	-22.0113
6.43	-22.179
6.44	-22.3496
6.45	-22.5231
6.46	-22.6996
6.47	-22.8791
6.48	-23.0617
6.49	-23.2474
6.5	-23.4363
6.51	-23.6284
6.52	-23.8237
6.53	-24.0223
6.54	-24.2241
6.55	-24.4291
6.56	-24.6374
6.57	-24.8488
6.58	-25.0633
6.59	-25.2808
6.6	-25.5012
6.61	-25.7242
6.62	-25.9497
6.63	-26.1774
6.64	-26.407
6.65	-26.638

6.66	-26.87
6.67	-27.1023
6.68	-27.3344
6.69	-27.5653
6.7	-27.7942
6.71	-28.0199
6.72	-28.2414
6.73	-28.4571
6.74	-28.6656
6.75	-28.8653
6.76	-29.0543
6.77	-29.2307
6.78	-29.3926
6.79	-29.538
6.8	-29.665
6.81	-29.7718
6.82	-29.8567
6.83	-29.9184
6.84	-29.9558
6.85	-29.9684
6.86	-29.9558
6.87	-29.9184
6.88	-29.8567
6.89	-29.7718
6.9	-29.665
6.91	-29.538
6.92	-29.3926
6.93	-29.2307
6.94	-29.0543
6.95	-28.8653
6.96	-28.6656
6.97	-28.4571
6.98	-28.2414
6.99	-28.0199
7	-27.7942
7.01	-27.5653
7.02	-27.3344
7.03	-27.1023
7.04	-26.87
7.05	-26.638
7.06	-26.407
7.07	-26.1774
7.08	-25.9497
7.09	-25.7242
7.1	-25.5012
7.11	-25.2808
7.12	-25.0633
7.13	-24.8488

7.14	-24.6374
7.15	-24.4291
7.16	-24.2241
7.17	-24.0223
7.18	-23.8237
7.19	-23.6284
7.2	-23.4363
7.21	-23.2474
7.22	-23.0617
7.23	-22.8791
7.24	-22.6996
7.25	-22.5231
7.26	-22.3496
7.27	-22.179
7.28	-22.0113
7.29	-21.8464
7.3	-21.6843
7.31	-21.5248
7.32	-21.368
7.33	-21.2137
7.34	-21.062
7.35	-20.9128
7.36	-20.7659
7.37	-20.6214
7.38	-20.4793
7.39	-20.3393
7.4	-20.2016
7.41	-20.066
7.42	-19.9325
7.43	-19.801
7.44	-19.6716
7.45	-19.5441
7.46	-19.4185
7.47	-19.2948
7.48	-19.1729
7.49	-19.0528
7.5	-18.9345
7.51	-18.8179
7.52	-18.7029
7.53	-18.5896
7.54	-18.4779
7.55	-18.3677
7.56	-18.2591
7.57	-18.152
7.58	-18.0463
7.59	-17.9421
7.6	-17.8393
7.61	-17.7379

7.62	-17.6379
7.63	-17.5392
7.64	-17.4418
7.65	-17.3456
7.66	-17.2507
7.67	-17.1571
7.68	-17.0646
7.69	-16.9734
7.7	-16.8833
7.71	-16.7943
7.72	-16.7065
7.73	-16.6198
7.74	-16.5341
7.75	-16.4495
7.76	-16.366
7.77	-16.2834
7.78	-16.2019
7.79	-16.1214
7.8	-16.0419
7.81	-15.9633
7.82	-15.8856
7.83	-15.8089
7.84	-15.7331
7.85	-15.6582
7.86	-15.5842
7.87	-15.511
7.88	-15.4388
7.89	-15.3673
7.9	-15.2967
7.91	-15.2269
7.92	-15.1579
7.93	-15.0897
7.94	-15.0223
7.95	-14.9557
7.96	-14.8898
7.97	-14.8247
7.98	-14.7603
7.99	-14.6967
8	-14.6338
8.01	-14.5715
8.02	-14.51
8.03	-14.4492
8.04	-14.3891
8.05	-14.3296
8.06	-14.2709
8.07	-14.2127
8.08	-14.1553
8.09	-14.0984

8.1	-14.0422
8.11	-13.9867
8.12	-13.9317
8.13	-13.8774
8.14	-13.8237
8.15	-13.7706
8.16	-13.718
8.17	-13.6661
8.18	-13.6147
8.19	-13.5639
8.2	-13.5137
8.21	-13.464
8.22	-13.4149
8.23	-13.3664
8.24	-13.3184
8.25	-13.2709
8.26	-13.224
8.27	-13.1776
8.28	-13.1317
8.29	-13.0863
8.3	-13.0415
8.31	-12.9971
8.32	-12.9533
8.33	-12.9099
8.34	-12.8671
8.35	-12.8248
8.36	-12.7829
8.37	-12.7415
8.38	-12.7006
8.39	-12.6602
8.4	-12.6202
8.41	-12.5807
8.42	-12.5417
8.43	-12.5032
8.44	-12.465
8.45	-12.4274
8.46	-12.3902
8.47	-12.3534
8.48	-12.3171
8.49	-12.2812
8.5	-12.2458
8.51	-12.2108
8.52	-12.1762
8.53	-12.1421
8.54	-12.1084
8.55	-12.0751
8.56	-12.0422
8.57	-12.0097

8.58	-11.9777
8.59	-11.9461
8.6	-11.9148
8.61	-11.884
8.62	-11.8536
8.63	-11.8236
8.64	-11.794
8.65	-11.7648
8.66	-11.736
8.67	-11.7076
8.68	-11.6796
8.69	-11.652
8.7	-11.6247
8.71	-11.5979
8.72	-11.5714
8.73	-11.5454
8.74	-11.5197
8.75	-11.4944
8.76	-11.4694
8.77	-11.4449
8.78	-11.4207
8.79	-11.3969
8.8	-11.3735
8.81	-11.3504
8.82	-11.3277
8.83	-11.3054
8.84	-11.2835
8.85	-11.2619
8.86	-11.2407
8.87	-11.2198
8.88	-11.1994
8.89	-11.1792
8.9	-11.1595
8.91	-11.1401
8.92	-11.121
8.93	-11.1024
8.94	-11.0841
8.95	-11.0661
8.96	-11.0485
8.97	-11.0312
8.98	-11.0144
8.99	-10.9978
9	-10.9816
9.01	-10.9658
9.02	-10.9503
9.03	-10.9352
9.04	-10.9204
9.05	-10.906

9.06	-10.8919
9.07	-10.8782
9.08	-10.8648
9.09	-10.8518
9.1	-10.8391
9.11	-10.8267
9.12	-10.8147
9.13	-10.8031
9.14	-10.7918
9.15	-10.7808
9.16	-10.7702
9.17	-10.7599
9.18	-10.75
9.19	-10.7403
9.2	-10.7311
9.21	-10.7222
9.22	-10.7136
9.23	-10.7053
9.24	-10.6974
9.25	-10.6898
9.26	-10.6826
9.27	-10.6757
9.28	-10.6691
9.29	-10.6628
9.3	-10.6569
9.31	-10.6513
9.32	-10.646
9.33	-10.6411
9.34	-10.6364
9.35	-10.6321
9.36	-10.6281
9.37	-10.6245
9.38	-10.6211
9.39	-10.618
9.4	-10.6153
9.41	-10.6129
9.42	-10.6107
9.43	-10.6089
9.44	-10.6073
9.45	-10.6061
9.46	-10.6051
9.47	-10.6044
9.48	-10.604
9.49	-10.6039
9.5	-10.604
9.51	-10.6044
9.52	-10.605
9.53	-10.6059

9.54	-10.6071
9.55	-10.6085
9.56	-10.6101
9.57	-10.6119
9.58	-10.6139
9.59	-10.6162
9.6	-10.6186
9.61	-10.6212
9.62	-10.624
9.63	-10.6269
9.64	-10.63
9.65	-10.6332
9.66	-10.6365
9.67	-10.64
9.68	-10.6435
9.69	-10.6471
9.7	-10.6507
9.71	-10.6544
9.72	-10.6581
9.73	-10.6617
9.74	-10.6654
9.75	-10.669
9.76	-10.6725
9.77	-10.6759
9.78	-10.6791
9.79	-10.6822
9.8	-10.6851
9.81	-10.6877
9.82	-10.6901
9.83	-10.6922
9.84	-10.694
9.85	-10.6954
9.86	-10.6963
9.87	-10.6968
9.88	-10.6967
9.89	-10.6961
9.9	-10.6949
9.91	-10.6931
9.92	-10.6905
9.93	-10.6871
9.94	-10.6828
9.95	-10.6777
9.96	-10.6716
9.97	-10.6645
9.98	-10.6563
9.99	-10.6469
10	-10.6362
10.01	-10.6242

10.02	-10.6109
10.03	-10.596
10.04	-10.5796
10.05	-10.5615
10.06	-10.5417
10.07	-10.52
10.08	-10.4965
10.09	-10.471
10.1	-10.4434
10.11	-10.4136
10.12	-10.3816
10.13	-10.3472
10.14	-10.3104
10.15	-10.271
10.16	-10.2291
10.17	-10.1845
10.18	-10.1372
10.19	-10.087
10.2	-10.0339
10.21	-9.97784
10.22	-9.91876
10.23	-9.85659
10.24	-9.79129
10.25	-9.7228
10.26	-9.65111
10.27	-9.57616
10.28	-9.49795
10.29	-9.41645
10.3	-9.33165
10.31	-9.24357
10.32	-9.1522
10.33	-9.05757
10.34	-8.9597
10.35	-8.85863
10.36	-8.75441
10.37	-8.64707
10.38	-8.5367
10.39	-8.42334
10.4	-8.3071
10.41	-8.18804
10.42	-8.06626
10.43	-7.94187
10.44	-7.81497
10.45	-7.68567
10.46	-7.55409
10.47	-7.42036
10.48	-7.28461
10.49	-7.14698

10.5	-7.0076
10.51	-6.86663
10.52	-6.72419
10.53	-6.58046
10.54	-6.43557
10.55	-6.28969
10.56	-6.14297
10.57	-5.99557
10.58	-5.84765
10.59	-5.69937
10.6	-5.55089
10.61	-5.40238
10.62	-5.25398
10.63	-5.10587
10.64	-4.95819
10.65	-4.81111
10.66	-4.66478
10.67	-4.51935
10.68	-4.37497
10.69	-4.2318
10.7	-4.08996
10.71	-3.94962
10.72	-3.81089
10.73	-3.67391
10.74	-3.53881
10.75	-3.40572
10.76	-3.27474
10.77	-3.146
10.78	-3.01959
10.79	-2.89562
10.8	-2.77417
10.81	-2.65535
10.82	-2.53921
10.83	-2.42584
10.84	-2.3153
10.85	-2.20765
10.86	-2.10293
10.87	-2.00119
10.88	-1.90246
10.89	-1.80676
10.9	-1.71412
10.91	-1.62455
10.92	-1.53804
10.93	-1.4546
10.94	-1.37421
10.95	-1.29686
10.96	-1.22252
10.97	-1.15116

10.98	-1.08274
10.99	-1.01722
11	-0.95456
11.01	-0.8947
11.02	-0.83759
11.03	-0.78316
11.04	-0.73136
11.05	-0.68211
11.06	-0.63536
11.07	-0.59101
11.08	-0.54901
11.09	-0.50928
11.1	-0.47174
11.11	-0.43631
11.12	-0.40293
11.13	-0.3715
11.14	-0.34196
11.15	-0.31423
11.16	-0.28823
11.17	-0.26389
11.18	-0.24113
11.19	-0.21988
11.2	-0.20007
11.21	-0.18163
11.22	-0.1645
11.23	-0.1486
11.24	-0.13388
11.25	-0.12026
11.26	-0.1077
11.27	-0.09613
11.28	-0.08549
11.29	-0.07574
11.3	-0.06682
11.31	-0.05868
11.32	-0.05127
11.33	-0.04455
11.34	-0.03847
11.35	-0.033
11.36	-0.02808
11.37	-0.02369
11.38	-0.01979
11.39	-0.01634
11.4	-0.01331
11.41	-0.01067
11.42	-0.00839
11.43	-0.00644
11.44	-0.0048
11.45	-0.00345

11.46	-0.00235
11.47	-0.00148
11.48	-0.00084
11.49	-0.00039
11.5	-0.00012
11.51	-4.49E-06
11.52	-3.85E-05
11.53	-0.0002
11.54	-0.00048
11.55	-0.00087
11.56	-0.00134
11.57	-0.00189
11.58	-0.00251
11.59	-0.00319
11.6	-0.00393
11.61	-0.0047
11.62	-0.0055
11.63	-0.00633
11.64	-0.00719
11.65	-0.00805
11.66	-0.00893
11.67	-0.00981
11.68	-0.01068
11.69	-0.01155
11.7	-0.01241
11.71	-0.01326
11.72	-0.0141
11.73	-0.01491
11.74	-0.01571
11.75	-0.01648
11.76	-0.01722
11.77	-0.01794
11.78	-0.01864
11.79	-0.0193
11.8	-0.01993
11.81	-0.02053
11.82	-0.0211
11.83	-0.02164
11.84	-0.02215
11.85	-0.02262
11.86	-0.02306
11.87	-0.02347
11.88	-0.02385
11.89	-0.02419
11.9	-0.02451
11.91	-0.02479
11.92	-0.02504
11.93	-0.02526

11.94	-0.02545
11.95	-0.02561
11.96	-0.02574
11.97	-0.02585
11.98	-0.02593
11.99	-0.02598
12	-0.026
12.01	-0.02601
12.02	-0.02599
12.03	-0.02594
12.04	-0.02587
12.05	-0.02579
12.06	-0.02568
12.07	-0.02555
12.08	-0.02541
12.09	-0.02524
12.1	-0.02506
12.11	-0.02487
12.12	-0.02465
12.13	-0.02443
12.14	-0.02419
12.15	-0.02394
12.16	-0.02367
12.17	-0.0234
12.18	-0.02311
12.19	-0.02282
12.2	-0.02251
12.21	-0.0222
12.22	-0.02188
12.23	-0.02155
12.24	-0.02122
12.25	-0.02088
12.26	-0.02053
12.27	-0.02018
12.28	-0.01983
12.29	-0.01947
12.3	-0.01911
12.31	-0.01875
12.32	-0.01839
12.33	-0.01802
12.34	-0.01766
12.35	-0.01729
12.36	-0.01692
12.37	-0.01656
12.38	-0.01619
12.39	-0.01583
12.4	-0.01546
12.41	-0.0151

12.42	-0.01474
12.43	-0.01439
12.44	-0.01403
12.45	-0.01368
12.46	-0.01333
12.47	-0.01298
12.48	-0.01264
12.49	-0.0123
12.5	-0.01197
12.51	-0.01164
12.52	-0.01131
12.53	-0.01099
12.54	-0.01067
12.55	-0.01036
12.56	-0.01005
12.57	-0.00975
12.58	-0.00945
12.59	-0.00916
12.6	-0.00887
12.61	-0.00859
12.62	-0.00831
12.63	-0.00804
12.64	-0.00777
12.65	-0.00751
12.66	-0.00725
12.67	-0.007
12.68	-0.00676
12.69	-0.00652
12.7	-0.00628
12.71	-0.00605
12.72	-0.00583
12.73	-0.00561
12.74	-0.0054
12.75	-0.00519
12.76	-0.00499
12.77	-0.00479
12.78	-0.0046
12.79	-0.00442
12.8	-0.00424
12.81	-0.00406
12.82	-0.00389
12.83	-0.00372
12.84	-0.00356
12.85	-0.00341
12.86	-0.00326
12.87	-0.00311
12.88	-0.00297
12.89	-0.00283

12.9	-0.0027
12.91	-0.00257
12.92	-0.00245
12.93	-0.00233
12.94	-0.00221
12.95	-0.0021
12.96	-0.002
12.97	-0.00189
12.98	-0.00179
12.99	-0.0017
13	-0.00161
13.01	-0.00152
13.02	-0.00143
13.03	-0.00135
13.04	-0.00128
13.05	-0.0012
13.06	-0.00113
13.07	-0.00106
13.08	-0.001
13.09	-0.00094
13.1	-0.00088
13.11	-0.00082
13.12	-0.00077
13.13	-0.00072
13.14	-0.00067
13.15	-0.00062
13.16	-0.00058
13.17	-0.00054
13.18	-0.0005
13.19	-0.00046
13.2	-0.00043
13.21	-0.00039
13.22	-0.00036
13.23	-0.00033
13.24	-0.00031
13.25	-0.00028
13.26	-0.00026
13.27	-0.00023
13.28	-0.00021
13.29	-0.00019
13.3	-0.00018
13.31	-0.00016
13.32	-0.00014
13.33	-0.00013
13.34	-0.00012
13.35	-0.0001
13.36	-9.19E-05
13.37	-8.16E-05

13.38	-7.22E-05
13.39	-6.36E-05
13.4	-5.58E-05
13.41	-4.87E-05
13.42	-4.24E-05
13.43	-3.66E-05
13.44	-3.15E-05
13.45	-2.69E-05
13.46	-2.29E-05
13.47	-1.93E-05
13.48	-1.62E-05
13.49	-1.34E-05
13.5	-1.10E-05
13.51	-9.00E-06
13.52	-7.25E-06
13.53	-5.77E-06
13.54	-4.53E-06
13.55	-3.50E-06
13.56	-2.65E-06
13.57	-1.97E-06
13.58	-1.43E-06
13.59	-1.01E-06
13.6	-6.91E-07
13.61	-4.54E-07
13.62	-2.83E-07
13.63	-1.66E-07
13.64	-8.96E-08
13.65	-4.32E-08
13.66	-1.77E-08
13.67	-5.60E-09
13.68	-1.11E-09
13.69	-6.92E-11
13.7	0
13.71	-6.92E-11
13.72	-1.11E-09
13.73	-5.60E-09
13.74	-1.77E-08
13.75	-4.32E-08
13.76	-8.96E-08
13.77	-1.66E-07
13.78	-2.83E-07
13.79	-4.54E-07
13.8	-6.91E-07
13.81	-1.01E-06
13.82	-1.43E-06
13.83	-1.97E-06
13.84	-2.65E-06
13.85	-3.50E-06

13.86	-4.53E-06
13.87	-5.77E-06
13.88	-7.25E-06
13.89	-9.00E-06
13.9	-1.10E-05
13.91	-1.34E-05
13.92	-1.62E-05
13.93	-1.93E-05
13.94	-2.29E-05
13.95	-2.69E-05
13.96	-3.15E-05
13.97	-3.66E-05
13.98	-4.24E-05
13.99	-4.87E-05
14	-5.58E-05
14.01	-6.36E-05
14.02	-7.22E-05
14.03	-8.16E-05
14.04	-9.19E-05
14.05	-0.0001
14.06	-0.00012
14.07	-0.00013
14.08	-0.00014
14.09	-0.00016
14.1	-0.00018
14.11	-0.00019
14.12	-0.00021
14.13	-0.00023
14.14	-0.00026
14.15	-0.00028
14.16	-0.00031
14.17	-0.00033
14.18	-0.00036
14.19	-0.00039
14.2	-0.00043
14.21	-0.00046
14.22	-0.0005
14.23	-0.00054
14.24	-0.00058
14.25	-0.00062
14.26	-0.00067
14.27	-0.00072
14.28	-0.00077
14.29	-0.00082
14.3	-0.00088
14.31	-0.00094
14.32	-0.001
14.33	-0.00106

14.34	-0.00113
14.35	-0.0012
14.36	-0.00128
14.37	-0.00135
14.38	-0.00143
14.39	-0.00152
14.4	-0.00161
14.41	-0.0017
14.42	-0.00179
14.43	-0.00189
14.44	-0.002
14.45	-0.0021
14.46	-0.00221
14.47	-0.00233
14.48	-0.00245
14.49	-0.00257
14.5	-0.0027
14.51	-0.00283
14.52	-0.00297
14.53	-0.00311
14.54	-0.00326
14.55	-0.00341
14.56	-0.00356
14.57	-0.00372
14.58	-0.00389
14.59	-0.00406
14.6	-0.00424
14.61	-0.00442
14.62	-0.0046
14.63	-0.00479
14.64	-0.00499
14.65	-0.00519
14.66	-0.0054
14.67	-0.00561
14.68	-0.00583
14.69	-0.00605
14.7	-0.00628
14.71	-0.00652
14.72	-0.00676
14.73	-0.007
14.74	-0.00725
14.75	-0.00751
14.76	-0.00777
14.77	-0.00804
14.78	-0.00831
14.79	-0.00859
14.8	-0.00887
14.81	-0.00916

14.82	-0.00945
14.83	-0.00975
14.84	-0.01005
14.85	-0.01036
14.86	-0.01067
14.87	-0.01099
14.88	-0.01131
14.89	-0.01164
14.9	-0.01197
14.91	-0.0123
14.92	-0.01264
14.93	-0.01298
14.94	-0.01333
14.95	-0.01368
14.96	-0.01403
14.97	-0.01439
14.98	-0.01474
14.99	-0.0151
15	-0.01546
15.01	-0.01583
15.02	-0.01619
15.03	-0.01656
15.04	-0.01692
15.05	-0.01729
15.06	-0.01766
15.07	-0.01802
15.08	-0.01839
15.09	-0.01875
15.1	-0.01911
15.11	-0.01947
15.12	-0.01983
15.13	-0.02018
15.14	-0.02053
15.15	-0.02088
15.16	-0.02122
15.17	-0.02155
15.18	-0.02188
15.19	-0.0222
15.2	-0.02251
15.21	-0.02282
15.22	-0.02311
15.23	-0.0234
15.24	-0.02367
15.25	-0.02394
15.26	-0.02419
15.27	-0.02443
15.28	-0.02465
15.29	-0.02487

15.3	-0.02506
15.31	-0.02524
15.32	-0.02541
15.33	-0.02555
15.34	-0.02568
15.35	-0.02579
15.36	-0.02587
15.37	-0.02594
15.38	-0.02599
15.39	-0.02601
15.4	-0.026
15.41	-0.02598
15.42	-0.02593
15.43	-0.02585
15.44	-0.02574
15.45	-0.02561
15.46	-0.02545
15.47	-0.02526
15.48	-0.02504
15.49	-0.02479
15.5	-0.02451
15.51	-0.02419
15.52	-0.02385
15.53	-0.02347
15.54	-0.02306
15.55	-0.02262
15.56	-0.02215
15.57	-0.02164
15.58	-0.0211
15.59	-0.02053
15.6	-0.01993
15.61	-0.0193
15.62	-0.01864
15.63	-0.01794
15.64	-0.01722
15.65	-0.01648
15.66	-0.01571
15.67	-0.01491
15.68	-0.0141
15.69	-0.01326
15.7	-0.01241
15.71	-0.01155
15.72	-0.01068
15.73	-0.00981
15.74	-0.00893
15.75	-0.00805
15.76	-0.00719
15.77	-0.00633

15.78	-0.0055
15.79	-0.0047
15.8	-0.00393
15.81	-0.00319
15.82	-0.00251
15.83	-0.00189
15.84	-0.00134
15.85	-0.00087
15.86	-0.00048
15.87	-0.0002
15.88	-3.85E-05
15.89	-4.49E-06
15.9	-0.00012
15.91	-0.00039
15.92	-0.00084
15.93	-0.00148
15.94	-0.00235
15.95	-0.00345
15.96	-0.0048
15.97	-0.00644
15.98	-0.00839
15.99	-0.01067
16	-0.01331

one balun_80 ohms Data File

Frequency (	DB[S21]
0.05	-80.0209
0.06	-76.8539
0.07	-74.1762
0.08	-71.8568
0.09	-69.8111
0.1	-67.9811
0.11	-66.3259
0.12	-64.8148
0.13	-63.4248
0.14	-62.1379
0.15	-60.94
0.16	-59.8194
0.17	-58.767
0.18	-57.7747
0.19	-56.8362
0.2	-55.946
0.21	-55.0993
0.22	-54.292
0.23	-53.5208
0.24	-52.7824
0.25	-52.0743
0.26	-51.3941

0.27	-50.7397
0.28	-50.1091
0.29	-49.5008
0.3	-48.9132
0.31	-48.345
0.32	-47.7949
0.33	-47.2619
0.34	-46.7448
0.35	-46.2429
0.36	-45.7553
0.37	-45.2811
0.38	-44.8197
0.39	-44.3704
0.4	-43.9325
0.41	-43.5057
0.42	-43.0892
0.43	-42.6827
0.44	-42.2857
0.45	-41.8977
0.46	-41.5184
0.47	-41.1474
0.48	-40.7844
0.49	-40.429
0.5	-40.0809
0.51	-39.7399
0.52	-39.4056
0.53	-39.078
0.54	-38.7566
0.55	-38.4412
0.56	-38.1318
0.57	-37.828
0.58	-37.5296
0.59	-37.2366
0.6	-36.9487
0.61	-36.6657
0.62	-36.3876
0.63	-36.1141
0.64	-35.8452
0.65	-35.5806
0.66	-35.3203
0.67	-35.0641
0.68	-34.812
0.69	-34.5638
0.7	-34.3195
0.71	-34.0788
0.72	-33.8418
0.73	-33.6083
0.74	-33.3783

0.75	-33.1516
0.76	-32.9283
0.77	-32.7081
0.78	-32.4911
0.79	-32.2772
0.8	-32.0662
0.81	-31.8583
0.82	-31.6532
0.83	-31.4509
0.84	-31.2514
0.85	-31.0547
0.86	-30.8606
0.87	-30.6691
0.88	-30.4802
0.89	-30.2938
0.9	-30.1099
0.91	-29.9285
0.92	-29.7494
0.93	-29.5728
0.94	-29.3985
0.95	-29.2264
0.96	-29.0567
0.97	-28.8892
0.98	-28.7239
0.99	-28.5607
1	-28.3998
1.01	-28.2409
1.02	-28.0842
1.03	-27.9296
1.04	-27.777
1.05	-27.6265
1.06	-27.4779
1.07	-27.3314
1.08	-27.1869
1.09	-27.0444
1.1	-26.9038
1.11	-26.7652
1.12	-26.6285
1.13	-26.4937
1.14	-26.3609
1.15	-26.23
1.16	-26.1009
1.17	-25.9738
1.18	-25.8485
1.19	-25.7252
1.2	-25.6037
1.21	-25.4841
1.22	-25.3664

1.23	-25.2505
1.24	-25.1365
1.25	-25.0245
1.26	-24.9143
1.27	-24.8059
1.28	-24.6995
1.29	-24.595
1.3	-24.4924
1.31	-24.3917
1.32	-24.293
1.33	-24.1961
1.34	-24.1013
1.35	-24.0084
1.36	-23.9175
1.37	-23.8285
1.38	-23.7416
1.39	-23.6568
1.4	-23.574
1.41	-23.4933
1.42	-23.4147
1.43	-23.3382
1.44	-23.2639
1.45	-23.1918
1.46	-23.1219
1.47	-23.0542
1.48	-22.9889
1.49	-22.9259
1.5	-22.8652
1.51	-22.807
1.52	-22.7512
1.53	-22.698
1.54	-22.6473
1.55	-22.5992
1.56	-22.5538
1.57	-22.5111
1.58	-22.4712
1.59	-22.4342
1.6	-22.4002
1.61	-22.3691
1.62	-22.3411
1.63	-22.3163
1.64	-22.2948
1.65	-22.2767
1.66	-22.262
1.67	-22.2509
1.68	-22.2435
1.69	-22.2399
1.7	-22.2403

1.71	-22.2448
1.72	-22.2535
1.73	-22.2666
1.74	-22.2842
1.75	-22.3067
1.76	-22.334
1.77	-22.3666
1.78	-22.4045
1.79	-22.4481
1.8	-22.4975
1.81	-22.5531
1.82	-22.6153
1.83	-22.6842
1.84	-22.7603
1.85	-22.844
1.86	-22.9357
1.87	-23.0358
1.88	-23.145
1.89	-23.2637
1.9	-23.3926
1.91	-23.5324
1.92	-23.6839
1.93	-23.8478
1.94	-24.0253
1.95	-24.2172
1.96	-24.425
1.97	-24.6499
1.98	-24.8936
1.99	-25.1578
2	-25.4447
2.01	-25.7567
2.02	-26.0967
2.03	-26.4681
2.04	-26.8749
2.05	-27.3222
2.06	-27.8159
2.07	-28.3636
2.08	-28.9748
2.09	-29.6619
2.1	-30.4413
2.11	-31.3353
2.12	-32.3756
2.13	-33.6092
2.14	-35.11
2.15	-37.0053
2.16	-39.542
2.17	-43.3122
2.18	-50.5215

2.19	-59.8587
2.2	-45.7523
2.21	-40.4944
2.22	-37.1465
2.23	-34.6624
2.24	-32.6734
2.25	-31.0058
2.26	-29.564
2.27	-28.2896
2.28	-27.1444
2.29	-26.1019
2.3	-25.1432
2.31	-24.2541
2.32	-23.4238
2.33	-22.6438
2.34	-21.9076
2.35	-21.2097
2.36	-20.5456
2.37	-19.9116
2.38	-19.3047
2.39	-18.7223
2.4	-18.1621
2.41	-17.6223
2.42	-17.1011
2.43	-16.5973
2.44	-16.1094
2.45	-15.6365
2.46	-15.1776
2.47	-14.7317
2.48	-14.2982
2.49	-13.8764
2.5	-13.4657
2.51	-13.0655
2.52	-12.6754
2.53	-12.295
2.54	-11.9238
2.55	-11.5616
2.56	-11.2079
2.57	-10.8627
2.58	-10.5255
2.59	-10.1962
2.6	-9.87457
2.61	-9.56044
2.62	-9.25364
2.63	-8.95404
2.64	-8.66148
2.65	-8.37587
2.66	-8.09708

2.67	-7.82502
2.68	-7.5596
2.69	-7.30073
2.7	-7.04835
2.71	-6.80237
2.72	-6.56273
2.73	-6.32937
2.74	-6.10222
2.75	-5.88122
2.76	-5.66631
2.77	-5.45742
2.78	-5.25451
2.79	-5.0575
2.8	-4.86633
2.81	-4.68094
2.82	-4.50126
2.83	-4.32721
2.84	-4.15872
2.85	-3.99571
2.86	-3.83811
2.87	-3.68583
2.88	-3.53878
2.89	-3.39687
2.9	-3.26001
2.91	-3.12809
2.92	-3.00103
2.93	-2.87871
2.94	-2.76104
2.95	-2.6479
2.96	-2.53918
2.97	-2.43478
2.98	-2.33458
2.99	-2.23846
3	-2.14631
3.01	-2.05802
3.02	-1.97347
3.03	-1.89254
3.04	-1.81512
3.05	-1.7411
3.06	-1.67036
3.07	-1.60278
3.08	-1.53827
3.09	-1.4767
3.1	-1.41797
3.11	-1.36197
3.12	-1.3086
3.13	-1.25776
3.14	-1.20935

3.15	-1.16327
3.16	-1.11943
3.17	-1.07773
3.18	-1.03809
3.19	-1.00042
3.2	-0.96464
3.21	-0.93065
3.22	-0.8984
3.23	-0.86779
3.24	-0.83876
3.25	-0.81124
3.26	-0.78515
3.27	-0.76044
3.28	-0.73704
3.29	-0.71488
3.3	-0.69392
3.31	-0.67409
3.32	-0.65535
3.33	-0.63763
3.34	-0.6209
3.35	-0.6051
3.36	-0.59019
3.37	-0.57612
3.38	-0.56286
3.39	-0.55037
3.4	-0.53861
3.41	-0.52753
3.42	-0.51712
3.43	-0.50733
3.44	-0.49813
3.45	-0.4895
3.46	-0.4814
3.47	-0.47381
3.48	-0.4667
3.49	-0.46004
3.5	-0.45383
3.51	-0.44802
3.52	-0.4426
3.53	-0.43755
3.54	-0.43285
3.55	-0.42848
3.56	-0.42442
3.57	-0.42066
3.58	-0.41718
3.59	-0.41396
3.6	-0.41099
3.61	-0.40826
3.62	-0.40575

3.63	-0.40346
3.64	-0.40136
3.65	-0.39944
3.66	-0.39771
3.67	-0.39613
3.68	-0.39472
3.69	-0.39345
3.7	-0.39231
3.71	-0.39131
3.72	-0.39042
3.73	-0.38965
3.74	-0.38898
3.75	-0.38841
3.76	-0.38793
3.77	-0.38753
3.78	-0.38722
3.79	-0.38698
3.8	-0.3868
3.81	-0.38669
3.82	-0.38663
3.83	-0.38663
3.84	-0.38667
3.85	-0.38676
3.86	-0.38689
3.87	-0.38705
3.88	-0.38725
3.89	-0.38747
3.9	-0.38772
3.91	-0.38799
3.92	-0.38828
3.93	-0.38858
3.94	-0.3889
3.95	-0.38923
3.96	-0.38957
3.97	-0.38991
3.98	-0.39025
3.99	-0.3906
4	-0.39094
4.01	-0.39129
4.02	-0.39163
4.03	-0.39196
4.04	-0.39228
4.05	-0.3926
4.06	-0.3929
4.07	-0.39319
4.08	-0.39347
4.09	-0.39373
4.1	-0.39398

4.11	-0.39421
4.12	-0.39443
4.13	-0.39462
4.14	-0.39479
4.15	-0.39495
4.16	-0.39508
4.17	-0.39519
4.18	-0.39527
4.19	-0.39533
4.2	-0.39537
4.21	-0.39538
4.22	-0.39537
4.23	-0.39533
4.24	-0.39527
4.25	-0.39517
4.26	-0.39505
4.27	-0.39491
4.28	-0.39473
4.29	-0.39453
4.3	-0.39429
4.31	-0.39403
4.32	-0.39374
4.33	-0.39342
4.34	-0.39308
4.35	-0.3927
4.36	-0.39229
4.37	-0.39185
4.38	-0.39139
4.39	-0.39089
4.4	-0.39036
4.41	-0.3898
4.42	-0.38922
4.43	-0.3886
4.44	-0.38795
4.45	-0.38728
4.46	-0.38657
4.47	-0.38583
4.48	-0.38507
4.49	-0.38427
4.5	-0.38345
4.51	-0.38259
4.52	-0.38171
4.53	-0.3808
4.54	-0.37986
4.55	-0.37889
4.56	-0.37789
4.57	-0.37686
4.58	-0.37581

4.59	-0.37473
4.6	-0.37362
4.61	-0.37248
4.62	-0.37131
4.63	-0.37012
4.64	-0.3689
4.65	-0.36765
4.66	-0.36638
4.67	-0.36508
4.68	-0.36376
4.69	-0.36241
4.7	-0.36103
4.71	-0.35963
4.72	-0.35821
4.73	-0.35676
4.74	-0.35529
4.75	-0.35379
4.76	-0.35227
4.77	-0.35072
4.78	-0.34916
4.79	-0.34757
4.8	-0.34596
4.81	-0.34432
4.82	-0.34267
4.83	-0.34099
4.84	-0.33929
4.85	-0.33757
4.86	-0.33583
4.87	-0.33407
4.88	-0.33229
4.89	-0.33049
4.9	-0.32868
4.91	-0.32684
4.92	-0.32498
4.93	-0.32311
4.94	-0.32122
4.95	-0.31931
4.96	-0.31739
4.97	-0.31545
4.98	-0.31349
4.99	-0.31151
5	-0.30952
5.01	-0.30752
5.02	-0.3055
5.03	-0.30346
5.04	-0.30142
5.05	-0.29935
5.06	-0.29728

5.07	-0.29519
5.08	-0.29309
5.09	-0.29097
5.1	-0.28884
5.11	-0.2867
5.12	-0.28455
5.13	-0.28239
5.14	-0.28022
5.15	-0.27804
5.16	-0.27585
5.17	-0.27364
5.18	-0.27143
5.19	-0.26921
5.2	-0.26698
5.21	-0.26475
5.22	-0.2625
5.23	-0.26025
5.24	-0.25799
5.25	-0.25572
5.26	-0.25345
5.27	-0.25117
5.28	-0.24888
5.29	-0.24659
5.3	-0.2443
5.31	-0.242
5.32	-0.23969
5.33	-0.23738
5.34	-0.23507
5.35	-0.23275
5.36	-0.23043
5.37	-0.22811
5.38	-0.22579
5.39	-0.22346
5.4	-0.22113
5.41	-0.2188
5.42	-0.21647
5.43	-0.21414
5.44	-0.2118
5.45	-0.20947
5.46	-0.20714
5.47	-0.20481
5.48	-0.20248
5.49	-0.20015
5.5	-0.19782
5.51	-0.19549
5.52	-0.19317
5.53	-0.19085
5.54	-0.18853

5.55	-0.18621
5.56	-0.1839
5.57	-0.18159
5.58	-0.17929
5.59	-0.17699
5.6	-0.17469
5.61	-0.1724
5.62	-0.17011
5.63	-0.16783
5.64	-0.16556
5.65	-0.16329
5.66	-0.16103
5.67	-0.15877
5.68	-0.15653
5.69	-0.15428
5.7	-0.15205
5.71	-0.14983
5.72	-0.14761
5.73	-0.1454
5.74	-0.1432
5.75	-0.14101
5.76	-0.13883
5.77	-0.13666
5.78	-0.13449
5.79	-0.13234
5.8	-0.1302
5.81	-0.12807
5.82	-0.12595
5.83	-0.12384
5.84	-0.12174
5.85	-0.11966
5.86	-0.11758
5.87	-0.11552
5.88	-0.11347
5.89	-0.11143
5.9	-0.10941
5.91	-0.1074
5.92	-0.1054
5.93	-0.10342
5.94	-0.10145
5.95	-0.09949
5.96	-0.09755
5.97	-0.09563
5.98	-0.09372
5.99	-0.09182
6	-0.08994
6.01	-0.08807
6.02	-0.08622

6.03	-0.08439
6.04	-0.08257
6.05	-0.08077
6.06	-0.07899
6.07	-0.07722
6.08	-0.07547
6.09	-0.07373
6.1	-0.07202
6.11	-0.07032
6.12	-0.06864
6.13	-0.06698
6.14	-0.06533
6.15	-0.06371
6.16	-0.0621
6.17	-0.06051
6.18	-0.05894
6.19	-0.05739
6.2	-0.05586
6.21	-0.05435
6.22	-0.05286
6.23	-0.05139
6.24	-0.04994
6.25	-0.04851
6.26	-0.0471
6.27	-0.04571
6.28	-0.04434
6.29	-0.04299
6.3	-0.04166
6.31	-0.04035
6.32	-0.03907
6.33	-0.0378
6.34	-0.03656
6.35	-0.03534
6.36	-0.03414
6.37	-0.03297
6.38	-0.03181
6.39	-0.03068
6.4	-0.02957
6.41	-0.02848
6.42	-0.02742
6.43	-0.02638
6.44	-0.02536
6.45	-0.02436
6.46	-0.02339
6.47	-0.02244
6.48	-0.02151
6.49	-0.02061
6.5	-0.01973

6.51	-0.01888
6.52	-0.01804
6.53	-0.01724
6.54	-0.01645
6.55	-0.01569
6.56	-0.01496
6.57	-0.01424
6.58	-0.01356
6.59	-0.01289
6.6	-0.01225
6.61	-0.01164
6.62	-0.01105
6.63	-0.01049
6.64	-0.00994
6.65	-0.00943
6.66	-0.00894
6.67	-0.00847
6.68	-0.00803
6.69	-0.00761
6.7	-0.00722
6.71	-0.00686
6.72	-0.00652
6.73	-0.0062
6.74	-0.00591
6.75	-0.00564
6.76	-0.0054
6.77	-0.00519
6.78	-0.005
6.79	-0.00483
6.8	-0.00469
6.81	-0.00458
6.82	-0.00449
6.83	-0.00443
6.84	-0.00439
6.85	-0.00438
6.86	-0.00439
6.87	-0.00443
6.88	-0.00449
6.89	-0.00458
6.9	-0.00469
6.91	-0.00483
6.92	-0.005
6.93	-0.00519
6.94	-0.0054
6.95	-0.00564
6.96	-0.00591
6.97	-0.0062
6.98	-0.00652

6.99	-0.00686
7	-0.00722
7.01	-0.00761
7.02	-0.00803
7.03	-0.00847
7.04	-0.00894
7.05	-0.00943
7.06	-0.00994
7.07	-0.01049
7.08	-0.01105
7.09	-0.01164
7.1	-0.01225
7.11	-0.01289
7.12	-0.01356
7.13	-0.01424
7.14	-0.01496
7.15	-0.01569
7.16	-0.01645
7.17	-0.01724
7.18	-0.01804
7.19	-0.01888
7.2	-0.01973
7.21	-0.02061
7.22	-0.02151
7.23	-0.02244
7.24	-0.02339
7.25	-0.02436
7.26	-0.02536
7.27	-0.02638
7.28	-0.02742
7.29	-0.02848
7.3	-0.02957
7.31	-0.03068
7.32	-0.03181
7.33	-0.03297
7.34	-0.03414
7.35	-0.03534
7.36	-0.03656
7.37	-0.0378
7.38	-0.03907
7.39	-0.04035
7.4	-0.04166
7.41	-0.04299
7.42	-0.04434
7.43	-0.04571
7.44	-0.0471
7.45	-0.04851
7.46	-0.04994

7.47	-0.05139
7.48	-0.05286
7.49	-0.05435
7.5	-0.05586
7.51	-0.05739
7.52	-0.05894
7.53	-0.06051
7.54	-0.0621
7.55	-0.06371
7.56	-0.06533
7.57	-0.06698
7.58	-0.06864
7.59	-0.07032
7.6	-0.07202
7.61	-0.07373
7.62	-0.07547
7.63	-0.07722
7.64	-0.07899
7.65	-0.08077
7.66	-0.08257
7.67	-0.08439
7.68	-0.08622
7.69	-0.08807
7.7	-0.08994
7.71	-0.09182
7.72	-0.09372
7.73	-0.09563
7.74	-0.09755
7.75	-0.09949
7.76	-0.10145
7.77	-0.10342
7.78	-0.1054
7.79	-0.1074
7.8	-0.10941
7.81	-0.11143
7.82	-0.11347
7.83	-0.11552
7.84	-0.11758
7.85	-0.11966
7.86	-0.12174
7.87	-0.12384
7.88	-0.12595
7.89	-0.12807
7.9	-0.1302
7.91	-0.13234
7.92	-0.13449
7.93	-0.13666
7.94	-0.13883

7.95	-0.14101
7.96	-0.1432
7.97	-0.1454
7.98	-0.14761
7.99	-0.14983
8	-0.15205
8.01	-0.15428
8.02	-0.15653
8.03	-0.15877
8.04	-0.16103
8.05	-0.16329
8.06	-0.16556
8.07	-0.16783
8.08	-0.17011
8.09	-0.1724
8.1	-0.17469
8.11	-0.17699
8.12	-0.17929
8.13	-0.18159
8.14	-0.1839
8.15	-0.18621
8.16	-0.18853
8.17	-0.19085
8.18	-0.19317
8.19	-0.19549
8.2	-0.19782
8.21	-0.20015
8.22	-0.20248
8.23	-0.20481
8.24	-0.20714
8.25	-0.20947
8.26	-0.2118
8.27	-0.21414
8.28	-0.21647
8.29	-0.2188
8.3	-0.22113
8.31	-0.22346
8.32	-0.22579
8.33	-0.22811
8.34	-0.23043
8.35	-0.23275
8.36	-0.23507
8.37	-0.23738
8.38	-0.23969
8.39	-0.242
8.4	-0.2443
8.41	-0.24659
8.42	-0.24888

8.43	-0.25117
8.44	-0.25345
8.45	-0.25572
8.46	-0.25799
8.47	-0.26025
8.48	-0.2625
8.49	-0.26475
8.5	-0.26698
8.51	-0.26921
8.52	-0.27143
8.53	-0.27364
8.54	-0.27585
8.55	-0.27804
8.56	-0.28022
8.57	-0.28239
8.58	-0.28455
8.59	-0.2867
8.6	-0.28884
8.61	-0.29097
8.62	-0.29309
8.63	-0.29519
8.64	-0.29728
8.65	-0.29935
8.66	-0.30142
8.67	-0.30346
8.68	-0.3055
8.69	-0.30752
8.7	-0.30952
8.71	-0.31151
8.72	-0.31349
8.73	-0.31545
8.74	-0.31739
8.75	-0.31931
8.76	-0.32122
8.77	-0.32311
8.78	-0.32498
8.79	-0.32684
8.8	-0.32868
8.81	-0.33049
8.82	-0.33229
8.83	-0.33407
8.84	-0.33583
8.85	-0.33757
8.86	-0.33929
8.87	-0.34099
8.88	-0.34267
8.89	-0.34432
8.9	-0.34596

8.91	-0.34757
8.92	-0.34916
8.93	-0.35072
8.94	-0.35227
8.95	-0.35379
8.96	-0.35529
8.97	-0.35676
8.98	-0.35821
8.99	-0.35963
9	-0.36103
9.01	-0.36241
9.02	-0.36376
9.03	-0.36508
9.04	-0.36638
9.05	-0.36765
9.06	-0.3689
9.07	-0.37012
9.08	-0.37131
9.09	-0.37248
9.1	-0.37362
9.11	-0.37473
9.12	-0.37581
9.13	-0.37686
9.14	-0.37789
9.15	-0.37889
9.16	-0.37986
9.17	-0.3808
9.18	-0.38171
9.19	-0.38259
9.2	-0.38345
9.21	-0.38427
9.22	-0.38507
9.23	-0.38583
9.24	-0.38657
9.25	-0.38728
9.26	-0.38795
9.27	-0.3886
9.28	-0.38922
9.29	-0.3898
9.3	-0.39036
9.31	-0.39089
9.32	-0.39139
9.33	-0.39185
9.34	-0.39229
9.35	-0.3927
9.36	-0.39308
9.37	-0.39342
9.38	-0.39374

9.39	-0.39403
9.4	-0.39429
9.41	-0.39453
9.42	-0.39473
9.43	-0.39491
9.44	-0.39505
9.45	-0.39517
9.46	-0.39527
9.47	-0.39533
9.48	-0.39537
9.49	-0.39538
9.5	-0.39537
9.51	-0.39533
9.52	-0.39527
9.53	-0.39519
9.54	-0.39508
9.55	-0.39495
9.56	-0.39479
9.57	-0.39462
9.58	-0.39443
9.59	-0.39421
9.6	-0.39398
9.61	-0.39373
9.62	-0.39347
9.63	-0.39319
9.64	-0.3929
9.65	-0.3926
9.66	-0.39228
9.67	-0.39196
9.68	-0.39163
9.69	-0.39129
9.7	-0.39094
9.71	-0.3906
9.72	-0.39025
9.73	-0.38991
9.74	-0.38957
9.75	-0.38923
9.76	-0.3889
9.77	-0.38858
9.78	-0.38828
9.79	-0.38799
9.8	-0.38772
9.81	-0.38747
9.82	-0.38725
9.83	-0.38705
9.84	-0.38689
9.85	-0.38676
9.86	-0.38667

9.87	-0.38663
9.88	-0.38663
9.89	-0.38669
9.9	-0.3868
9.91	-0.38698
9.92	-0.38722
9.93	-0.38753
9.94	-0.38793
9.95	-0.38841
9.96	-0.38898
9.97	-0.38965
9.98	-0.39042
9.99	-0.39131
10	-0.39231
10.01	-0.39345
10.02	-0.39472
10.03	-0.39613
10.04	-0.39771
10.05	-0.39944
10.06	-0.40136
10.07	-0.40346
10.08	-0.40575
10.09	-0.40826
10.1	-0.41099
10.11	-0.41396
10.12	-0.41718
10.13	-0.42066
10.14	-0.42442
10.15	-0.42848
10.16	-0.43285
10.17	-0.43755
10.18	-0.4426
10.19	-0.44802
10.2	-0.45383
10.21	-0.46004
10.22	-0.4667
10.23	-0.47381
10.24	-0.4814
10.25	-0.4895
10.26	-0.49813
10.27	-0.50733
10.28	-0.51712
10.29	-0.52753
10.3	-0.53861
10.31	-0.55037
10.32	-0.56286
10.33	-0.57612
10.34	-0.59019

10.35	-0.6051
10.36	-0.6209
10.37	-0.63763
10.38	-0.65535
10.39	-0.67409
10.4	-0.69392
10.41	-0.71488
10.42	-0.73704
10.43	-0.76044
10.44	-0.78515
10.45	-0.81124
10.46	-0.83876
10.47	-0.86779
10.48	-0.8984
10.49	-0.93065
10.5	-0.96464
10.51	-1.00042
10.52	-1.03809
10.53	-1.07773
10.54	-1.11943
10.55	-1.16327
10.56	-1.20935
10.57	-1.25776
10.58	-1.3086
10.59	-1.36197
10.6	-1.41797
10.61	-1.4767
10.62	-1.53827
10.63	-1.60278
10.64	-1.67036
10.65	-1.7411
10.66	-1.81512
10.67	-1.89254
10.68	-1.97347
10.69	-2.05802
10.7	-2.14631
10.71	-2.23846
10.72	-2.33458
10.73	-2.43478
10.74	-2.53918
10.75	-2.6479
10.76	-2.76104
10.77	-2.87871
10.78	-3.00103
10.79	-3.12809
10.8	-3.26001
10.81	-3.39687
10.82	-3.53878

10.83	-3.68583
10.84	-3.83811
10.85	-3.99571
10.86	-4.15872
10.87	-4.32721
10.88	-4.50126
10.89	-4.68094
10.9	-4.86633
10.91	-5.0575
10.92	-5.25451
10.93	-5.45742
10.94	-5.66631
10.95	-5.88122
10.96	-6.10222
10.97	-6.32937
10.98	-6.56273
10.99	-6.80237
11	-7.04835
11.01	-7.30073
11.02	-7.5596
11.03	-7.82502
11.04	-8.09708
11.05	-8.37587
11.06	-8.66148
11.07	-8.95404
11.08	-9.25364
11.09	-9.56044
11.1	-9.87457
11.11	-10.1962
11.12	-10.5255
11.13	-10.8627
11.14	-11.2079
11.15	-11.5616
11.16	-11.9238
11.17	-12.295
11.18	-12.6754
11.19	-13.0655
11.2	-13.4657
11.21	-13.8764
11.22	-14.2982
11.23	-14.7317
11.24	-15.1776
11.25	-15.6365
11.26	-16.1094
11.27	-16.5973
11.28	-17.1011
11.29	-17.6223
11.3	-18.1621

11.31	-18.7223
11.32	-19.3047
11.33	-19.9116
11.34	-20.5456
11.35	-21.2097
11.36	-21.9076
11.37	-22.6438
11.38	-23.4238
11.39	-24.2541
11.4	-25.1432
11.41	-26.1019
11.42	-27.1444
11.43	-28.2896
11.44	-29.564
11.45	-31.0058
11.46	-32.6734
11.47	-34.6624
11.48	-37.1465
11.49	-40.4944
11.5	-45.7523
11.51	-59.8587
11.52	-50.5215
11.53	-43.3122
11.54	-39.542
11.55	-37.0053
11.56	-35.11
11.57	-33.6092
11.58	-32.3756
11.59	-31.3353
11.6	-30.4413
11.61	-29.6619
11.62	-28.9748
11.63	-28.3636
11.64	-27.8159
11.65	-27.3222
11.66	-26.8749
11.67	-26.4681
11.68	-26.0967
11.69	-25.7567
11.7	-25.4447
11.71	-25.1578
11.72	-24.8936
11.73	-24.6499
11.74	-24.425
11.75	-24.2172
11.76	-24.0253
11.77	-23.8478
11.78	-23.6839

11.79	-23.5324
11.8	-23.3926
11.81	-23.2637
11.82	-23.145
11.83	-23.0358
11.84	-22.9357
11.85	-22.844
11.86	-22.7603
11.87	-22.6842
11.88	-22.6153
11.89	-22.5531
11.9	-22.4975
11.91	-22.4481
11.92	-22.4045
11.93	-22.3666
11.94	-22.334
11.95	-22.3067
11.96	-22.2842
11.97	-22.2666
11.98	-22.2535
11.99	-22.2448
12	-22.2403
12.01	-22.2399
12.02	-22.2435
12.03	-22.2509
12.04	-22.262
12.05	-22.2767
12.06	-22.2948
12.07	-22.3163
12.08	-22.3411
12.09	-22.3691
12.1	-22.4002
12.11	-22.4342
12.12	-22.4712
12.13	-22.5111
12.14	-22.5538
12.15	-22.5992
12.16	-22.6473
12.17	-22.698
12.18	-22.7512
12.19	-22.807
12.2	-22.8652
12.21	-22.9259
12.22	-22.9889
12.23	-23.0542
12.24	-23.1219
12.25	-23.1918
12.26	-23.2639

12.27	-23.3382
12.28	-23.4147
12.29	-23.4933
12.3	-23.574
12.31	-23.6568
12.32	-23.7416
12.33	-23.8285
12.34	-23.9175
12.35	-24.0084
12.36	-24.1013
12.37	-24.1961
12.38	-24.293
12.39	-24.3917
12.4	-24.4924
12.41	-24.595
12.42	-24.6995
12.43	-24.8059
12.44	-24.9143
12.45	-25.0245
12.46	-25.1365
12.47	-25.2505
12.48	-25.3664
12.49	-25.4841
12.5	-25.6037
12.51	-25.7252
12.52	-25.8485
12.53	-25.9738
12.54	-26.1009
12.55	-26.23
12.56	-26.3609
12.57	-26.4937
12.58	-26.6285
12.59	-26.7652
12.6	-26.9038
12.61	-27.0444
12.62	-27.1869
12.63	-27.3314
12.64	-27.4779
12.65	-27.6265
12.66	-27.777
12.67	-27.9296
12.68	-28.0842
12.69	-28.2409
12.7	-28.3998
12.71	-28.5607
12.72	-28.7239
12.73	-28.8892
12.74	-29.0567

12.75	-29.2264
12.76	-29.3985
12.77	-29.5728
12.78	-29.7494
12.79	-29.9285
12.8	-30.1099
12.81	-30.2938
12.82	-30.4802
12.83	-30.6691
12.84	-30.8606
12.85	-31.0547
12.86	-31.2514
12.87	-31.4509
12.88	-31.6532
12.89	-31.8583
12.9	-32.0662
12.91	-32.2772
12.92	-32.4911
12.93	-32.7081
12.94	-32.9283
12.95	-33.1516
12.96	-33.3783
12.97	-33.6083
12.98	-33.8418
12.99	-34.0788
13	-34.3195
13.01	-34.5638
13.02	-34.812
13.03	-35.0641
13.04	-35.3203
13.05	-35.5806
13.06	-35.8452
13.07	-36.1141
13.08	-36.3876
13.09	-36.6657
13.1	-36.9487
13.11	-37.2366
13.12	-37.5296
13.13	-37.828
13.14	-38.1318
13.15	-38.4412
13.16	-38.7566
13.17	-39.078
13.18	-39.4056
13.19	-39.7399
13.2	-40.0809
13.21	-40.429
13.22	-40.7844

13.23	-41.1474
13.24	-41.5184
13.25	-41.8977
13.26	-42.2857
13.27	-42.6827
13.28	-43.0892
13.29	-43.5057
13.3	-43.9325
13.31	-44.3704
13.32	-44.8197
13.33	-45.2811
13.34	-45.7553
13.35	-46.2429
13.36	-46.7448
13.37	-47.2619
13.38	-47.7949
13.39	-48.345
13.4	-48.9132
13.41	-49.5008
13.42	-50.1091
13.43	-50.7397
13.44	-51.3941
13.45	-52.0743
13.46	-52.7824
13.47	-53.5208
13.48	-54.292
13.49	-55.0993
13.5	-55.946
13.51	-56.8362
13.52	-57.7747
13.53	-58.767
13.54	-59.8194
13.55	-60.94
13.56	-62.1379
13.57	-63.4248
13.58	-64.8148
13.59	-66.3259
13.6	-67.9811
13.61	-69.8111
13.62	-71.8568
13.63	-74.1762
13.64	-76.8539
13.65	-80.0209
13.75	-80.0209
13.76	-76.8539
13.77	-74.1762
13.78	-71.8568
13.79	-69.8111

13.8	-67.9811
13.81	-66.3259
13.82	-64.8148
13.83	-63.4248
13.84	-62.1379
13.85	-60.94
13.86	-59.8194
13.87	-58.767
13.88	-57.7747
13.89	-56.8362
13.9	-55.946
13.91	-55.0993
13.92	-54.292
13.93	-53.5208
13.94	-52.7824
13.95	-52.0743
13.96	-51.3941
13.97	-50.7397
13.98	-50.1091
13.99	-49.5008
14	-48.9132
14.01	-48.345
14.02	-47.7949
14.03	-47.2619
14.04	-46.7448
14.05	-46.2429
14.06	-45.7553
14.07	-45.2811
14.08	-44.8197
14.09	-44.3704
14.1	-43.9325
14.11	-43.5057
14.12	-43.0892
14.13	-42.6827
14.14	-42.2857
14.15	-41.8977
14.16	-41.5184
14.17	-41.1474
14.18	-40.7844
14.19	-40.429
14.2	-40.0809
14.21	-39.7399
14.22	-39.4056
14.23	-39.078
14.24	-38.7566
14.25	-38.4412
14.26	-38.1318
14.27	-37.828

14.28	-37.5296
14.29	-37.2366
14.3	-36.9487
14.31	-36.6657
14.32	-36.3876
14.33	-36.1141
14.34	-35.8452
14.35	-35.5806
14.36	-35.3203
14.37	-35.0641
14.38	-34.812
14.39	-34.5638
14.4	-34.3195
14.41	-34.0788
14.42	-33.8418
14.43	-33.6083
14.44	-33.3783
14.45	-33.1516
14.46	-32.9283
14.47	-32.7081
14.48	-32.4911
14.49	-32.2772
14.5	-32.0662
14.51	-31.8583
14.52	-31.6532
14.53	-31.4509
14.54	-31.2514
14.55	-31.0547
14.56	-30.8606
14.57	-30.6691
14.58	-30.4802
14.59	-30.2938
14.6	-30.1099
14.61	-29.9285
14.62	-29.7494
14.63	-29.5728
14.64	-29.3985
14.65	-29.2264
14.66	-29.0567
14.67	-28.8892
14.68	-28.7239
14.69	-28.5607
14.7	-28.3998
14.71	-28.2409
14.72	-28.0842
14.73	-27.9296
14.74	-27.777
14.75	-27.6265

14.76	-27.4779
14.77	-27.3314
14.78	-27.1869
14.79	-27.0444
14.8	-26.9038
14.81	-26.7652
14.82	-26.6285
14.83	-26.4937
14.84	-26.3609
14.85	-26.23
14.86	-26.1009
14.87	-25.9738
14.88	-25.8485
14.89	-25.7252
14.9	-25.6037
14.91	-25.4841
14.92	-25.3664
14.93	-25.2505
14.94	-25.1365
14.95	-25.0245
14.96	-24.9143
14.97	-24.8059
14.98	-24.6995
14.99	-24.595
15	-24.4924
15.01	-24.3917
15.02	-24.293
15.03	-24.1961
15.04	-24.1013
15.05	-24.0084
15.06	-23.9175
15.07	-23.8285
15.08	-23.7416
15.09	-23.6568
15.1	-23.574
15.11	-23.4933
15.12	-23.4147
15.13	-23.3382
15.14	-23.2639
15.15	-23.1918
15.16	-23.1219
15.17	-23.0542
15.18	-22.9889
15.19	-22.9259
15.2	-22.8652
15.21	-22.807
15.22	-22.7512
15.23	-22.698

15.24	-22.6473
15.25	-22.5992
15.26	-22.5538
15.27	-22.5111
15.28	-22.4712
15.29	-22.4342
15.3	-22.4002
15.31	-22.3691
15.32	-22.3411
15.33	-22.3163
15.34	-22.2948
15.35	-22.2767
15.36	-22.262
15.37	-22.2509
15.38	-22.2435
15.39	-22.2399
15.4	-22.2403
15.41	-22.2448
15.42	-22.2535
15.43	-22.2666
15.44	-22.2842
15.45	-22.3067
15.46	-22.334
15.47	-22.3666
15.48	-22.4045
15.49	-22.4481
15.5	-22.4975
15.51	-22.5531
15.52	-22.6153
15.53	-22.6842
15.54	-22.7603
15.55	-22.844
15.56	-22.9357
15.57	-23.0358
15.58	-23.145
15.59	-23.2637
15.6	-23.3926
15.61	-23.5324
15.62	-23.6839
15.63	-23.8478
15.64	-24.0253
15.65	-24.2172
15.66	-24.425
15.67	-24.6499
15.68	-24.8936
15.69	-25.1578
15.7	-25.4447
15.71	-25.7567

15.72	-26.0967
15.73	-26.4681
15.74	-26.8749
15.75	-27.3222
15.76	-27.8159
15.77	-28.3636
15.78	-28.9748
15.79	-29.6619
15.8	-30.4413
15.81	-31.3353
15.82	-32.3756
15.83	-33.6092
15.84	-35.11
15.85	-37.0053
15.86	-39.542
15.87	-43.3122
15.88	-50.5215
15.89	-59.8587
15.9	-45.7523
15.91	-40.4944
15.92	-37.1465
15.93	-34.6624
15.94	-32.6734
15.95	-31.0058
15.96	-29.564
15.97	-28.2896
15.98	-27.1444
15.99	-26.1019
16	-25.1432
