# Supplementary material for: Multilayer UWB bandpass filter using liquid crystal polymer technology
Source: Sci Rep. 2024 Jul 8;14:15669. doi: 10.1038/s41598-024-66928-8 (PMC11231142; doi:10.1038/s41598-024-66928-8)
Supplement: Supplementary file 3 — Supplementary Information 3. [file 41598_2024_66928_MOESM3_ESM.pdf]

Frequency (	Simulated	Measured
0.15	0.192937	0.328495
0.2	0.071918	0.280442
0.25	0.183835	0.144457
0.3	0.325029	0.188196
0.35	0.346471	0.262136
0.4	0.308412	0.294337
0.45	0.259548	0.292732
0.5	0.218613	0.277053
0.55	0.189825	0.259132
0.6	0.172207	0.244007
0.65	0.163376	0.233058
0.7	0.160883	0.225986
0.75	0.162646	0.221906
0.8	0.167051	0.219884
0.85	0.172928	0.219136
0.9	0.179482	0.219086
0.95	0.186206	0.219344
1	0.192806	0.219672
1.05	0.199133	0.219945
1.1	0.205135	0.220114
1.15	0.210821	0.220181
1.2	0.216237	0.220182
1.25	0.221447	0.220166
1.3	0.226527	0.220195
1.35	0.231557	0.220332
1.4	0.23662	0.220643
1.45	0.2418	0.22119
1.5	0.247178	0.222037
1.55	0.252838	0.223248
1.6	0.258865	0.224888
1.65	0.265343	0.227025
1.7	0.272358	0.229734
1.75	0.280001	0.233098
1.8	0.288362	0.237212
1.85	0.297537	0.242185
1.9	0.307623	0.248145
1.95	0.318719	0.255241
2	0.330923	0.263652
2.05	0.34432	0.273589
2.1	0.358982	0.285297
2.15	0.374942	0.299061
2.2	0.392181	0.315201
2.25	0.410589	0.334064
2.3	0.429937	0.35599
2.35	0.449833	0.381259
2.4	0.46969	0.409988
2.45	0.488718	0.441966

2.5	0.505954	0.476427
2.55	0.520349	0.51179
2.6	0.530915	0.545497
2.65	0.536905	0.574143
2.7	0.537968	0.594095
2.75	0.534225	0.602569
2.8	0.526246	0.598675
2.85	0.514911	0.583791
2.9	0.501239	0.560993
2.95	0.48622	0.533939
3	0.470703	0.50587
3.05	0.455342	0.479104
3.1	0.44059	0.454993
3.15	0.426724	0.434116
3.2	0.413885	0.416543
3.25	0.402115	0.402043
3.3	0.391392	0.39024
3.35	0.381655	0.380696
3.4	0.37282	0.372973
3.45	0.364799	0.366653
3.5	0.357502	0.361357
3.55	0.350844	0.356748
3.6	0.344751	0.35254
3.65	0.339153	0.348498
3.7	0.333992	0.344439
3.75	0.329219	0.34023
3.8	0.324791	0.335792
3.85	0.320678	0.331091
3.9	0.31686	0.326141
3.95	0.313353	0.321011
4	0.310303	0.315905
4.05	0.309266	0.312236
4.1	0.29292	0.295146
4.15	0.30463	0.302379
4.2	0.298597	0.294092
4.25	0.295596	0.288571
4.3	0.293037	0.283594
4.35	0.290691	0.279023
4.4	0.288503	0.274859
4.45	0.28645	0.271121
4.5	0.284519	0.267826
4.55	0.282702	0.264986
4.6	0.280991	0.262606
4.65	0.279381	0.260685
4.7	0.277864	0.259216
4.75	0.276435	0.258185
4.8	0.275089	0.257574
4.85	0.27382	0.25736

4.9	0.272621	0.257516
4.95	0.271489	0.258009
5	0.270418	0.258802
5.05	0.269404	0.259853
5.1	0.268443	0.261118
5.15	0.26753	0.262546
5.2	0.266662	0.264084
5.25	0.265835	0.265676
5.3	0.265046	0.267264
5.35	0.264293	0.268791
5.4	0.263573	0.270198
5.45	0.262884	0.271433
5.5	0.262224	0.272446
5.55	0.26159	0.273194
5.6	0.260983	0.273645
5.65	0.260401	0.273774
5.7	0.259842	0.27357
5.75	0.259307	0.27303
5.8	0.258794	0.272167
5.85	0.258303	0.271001
5.9	0.257835	0.269564
5.95	0.257388	0.267892
6	0.256962	0.26603
6.05	0.256558	0.264023
6.1	0.256175	0.261923
6.15	0.255814	0.259776
6.2	0.255474	0.257631
6.25	0.255155	0.255532
6.3	0.254857	0.253519
6.35	0.254579	0.251628
6.4	0.254323	0.24989
6.45	0.254087	0.24833
6.5	0.253871	0.246969
6.55	0.253675	0.245823
6.6	0.253501	0.244901
6.65	0.25335	0.24421
6.7	0.253221	0.243751
6.75	0.253114	0.243522
6.8	0.25303	0.243515
6.85	0.252968	0.243723
6.9	0.252928	0.24413
6.95	0.25291	0.244723
7	0.252913	0.245481
7.05	0.252938	0.246385
7.1	0.252984	0.247412
7.15	0.25305	0.248537
7.2	0.253136	0.249735
7.25	0.253243	0.25098

7.3	0.253369	0.252248
7.35	0.253514	0.253513
7.4	0.253677	0.254751
7.45	0.253859	0.255943
7.5	0.254057	0.257069
7.55	0.254273	0.258116
7.6	0.254504	0.259072
7.65	0.254751	0.259932
7.7	0.255011	0.260694
7.75	0.255285	0.261361
7.8	0.255571	0.261942
7.85	0.255868	0.262448
7.9	0.256175	0.262897
7.95	0.256492	0.263307
8	0.256816	0.2637
8.05	0.257148	0.264101
8.1	0.257487	0.264535
8.15	0.257833	0.265027
8.2	0.258189	0.265603
8.25	0.258558	0.266288
8.3	0.258949	0.267113
8.35	0.259379	0.268112
8.4	0.25989	0.269351
8.45	0.260596	0.270986
8.5	0.261904	0.273564
8.55	0.266181	0.280443
8.6	0.220563	0.229929
8.65	0.280071	0.2916
8.7	0.265451	0.282232
8.75	0.263159	0.280516
8.8	0.262761	0.281116
8.85	0.262832	0.282212
8.9	0.263096	0.2834
8.95	0.263477	0.284531
9	0.263954	0.285524
9.05	0.264525	0.286331
9.1	0.265198	0.28692
9.15	0.265987	0.287276
9.2	0.266911	0.2874
9.25	0.267988	0.287309
9.3	0.269245	0.287038
9.35	0.270707	0.286635
9.4	0.272407	0.28617
9.45	0.274376	0.285724
9.5	0.276654	0.285398
9.55	0.27928	0.285305
9.6	0.282301	0.285574
9.65	0.285766	0.286352

9.7	0.289726	0.2878
9.75	0.294237	0.2901
9.8	0.299358	0.293458
9.85	0.305147	0.298102
9.9	0.31166	0.304295
9.95	0.318946	0.312328
10	0.327045	0.322526
10.05	0.335973	0.335236
10.1	0.345716	0.350809
10.15	0.356217	0.369549
10.2	0.36735	0.391632
10.25	0.378918	0.416963
10.3	0.390634	0.444971
10.35	0.402108	0.474358
10.4	0.41285	0.502892
10.45	0.422292	0.527423
10.5	0.429841	0.544333
10.55	0.434938	0.550472
10.6	0.437142	0.544271
10.65	0.436199	0.526393
10.7	0.432086	0.499432
10.75	0.42502	0.466939
10.8	0.415415	0.43234
10.85	0.403816	0.398269
10.9	0.39082	0.366396
10.95	0.377005	0.337564
11	0.362879	0.312041
11.05	0.348853	0.289747
11.1	0.335237	0.270423
11.15	0.322245	0.253742
11.2	0.310011	0.239365
11.25	0.298604	0.226974
11.3	0.288048	0.216286
11.35	0.278333	0.207056
11.4	0.269426	0.199073
11.45	0.261281	0.19216
11.5	0.253848	0.186167
11.55	0.247069	0.180967
11.6	0.240892	0.176455
11.65	0.235262	0.172539
11.7	0.230129	0.169141
11.75	0.225447	0.166192
11.8	0.221172	0.163634
11.85	0.217265	0.161411
11.9	0.21369	0.159471
11.95	0.210411	0.157764
12	0.207398	0.156235
12.05	0.204621	0.154823

12.1	0.202048	0.15345
12.15	0.19965	0.152013
12.2	0.197392	0.15036
12.25	0.195233	0.148251
12.3	0.193119	0.145281
12.35	0.190973	0.140697
12.4	0.188672	0.13298
12.45	0.185999	0.118647
12.5	0.182542	0.088267
12.55	0.177425	0.008509
12.6	0.168549	-0.31564
12.65	0.149975	-4.19283
12.7	0.100189	-3.44419
12.75	-0.10191	-0.17915
12.8	-2.35948	0.241832
12.85	-4.23244	0.92028
12.9	5.19354	5.88254
12.95	2.08229	2.54833
13	0.504712	2.04722
13.05	0.557942	6.80465
13.1	1.01588	1.67295
13.15	-0.84828	0.50489
13.2	0.075256	0.298195
13.25	0.096516	0.240608
13.3	-0.00968	0.222837
13.35	-0.89012	0.218317
13.4	-7.14918	0.218899
13.45	-0.47	0.221674
13.5	0.035369	0.22547
13.55	0.125726	0.229757
13.6	0.15821	0.234304
13.65	0.174017	0.238998
13.7	0.183254	0.243753
13.75	0.189382	0.248508
13.8	0.19383	0.253215
13.85	0.197263	0.257829
13.9	0.199999	0.262307
13.95	0.202186	0.266607
14	0.203868	0.270684
14.05	0.205016	0.274493
14.1	0.205543	0.277988
14.15	0.205302	0.28112
14.2	0.205812	0.28384
14.25	0.207576	0.286096
14.3	0.209161	0.287833
14.35	0.210522	0.288987
14.4	0.211579	0.289486
14.45	0.212197	0.289236

14.5	0.21216	0.288109
14.55	0.211094	0.285914
14.6	0.208339	0.282349
14.65	0.202632	0.276908
14.7	0.191328	0.268583
14.75	0.168091	0.255332
14.8	0.114393	0.232556
14.85	-0.04538	0.187837
14.9	-0.92718	0.077242
14.95	-5.66939	-0.35874
15	-1.09504	-4.32647
15.05	-0.05272	-2.79017
15.1	0.128157	-0.20502
15.15	0.190855	0.090935
15.2	0.219769	0.169131
15.25	0.23425	0.193432
15.3	0.239507	0.192441
15.35	0.234877	0.166002
15.4	0.212707	0.084886
15.45	0.14392	-0.21736
15.5	-0.12517	-2.77932
15.55	-2.74825	-4.28517
15.6	-4.45905	-0.37298
15.65	-0.24869	0.075334
15.7	0.165926	0.192833
15.75	0.27811	0.244491
15.8	0.331494	0.275674
15.85	0.366885	0.299552
15.9	0.396101	0.321389
15.95	0.423878	0.343938
16	0.45268	0.369078
