# Supplementary material for: Multilayer UWB bandpass filter using liquid crystal polymer technology
Source: Sci Rep. 2024 Jul 8;14:15669. doi: 10.1038/s41598-024-66928-8 (PMC11231142; doi:10.1038/s41598-024-66928-8)
Supplement: Supplementary file 4 — Supplementary Information 4. [file 41598_2024_66928_MOESM4_ESM.pdf]

Frequency (	DB[S11]	DB[S21]	DB[S11]	DB[S21]
0.1	-0.00263	-101.354	-0.00343	-98.689
0.15	-0.00394	-90.7764	-0.0051	-85.5005
0.2	-0.00525	-84.0618	-0.00679	-79.7768
0.25	-0.00655	-77.8494	-0.00845	-74.8457
0.3	-0.00784	-72.5894	-0.01005	-70.2542
0.35	-0.00913	-68.2926	-0.01161	-66.1641
0.4	-0.01039	-64.7218	-0.01312	-62.6147
0.45	-0.01163	-61.6553	-0.01457	-59.5282
0.5	-0.01284	-58.9376	-0.01594	-56.8042
0.55	-0.01404	-56.4679	-0.01724	-54.3581
0.6	-0.01521	-54.1818	-0.01846	-52.1268
0.65	-0.01636	-52.0384	-0.0196	-50.0656
0.7	-0.0175	-50.0114	-0.02068	-48.1435
0.75	-0.01864	-48.0831	-0.0217	-46.3386
0.8	-0.01978	-46.2413	-0.02268	-44.6345
0.85	-0.02093	-44.4769	-0.02362	-43.019
0.9	-0.0221	-42.7827	-0.02453	-41.4819
0.95	-0.02331	-41.1523	-0.02543	-40.0148
1	-0.02456	-39.5803	-0.02634	-38.6104
1.05	-0.02587	-38.0616	-0.02726	-37.2619
1.1	-0.02725	-36.5913	-0.0282	-35.9632
1.15	-0.02874	-35.165	-0.0292	-34.7088
1.2	-0.03036	-33.7785	-0.03027	-33.4936
1.25	-0.03214	-32.428	-0.03145	-32.3126
1.3	-0.03415	-31.1097	-0.03276	-31.1615
1.35	-0.03645	-29.8203	-0.03426	-30.0361
1.4	-0.03912	-28.5566	-0.03601	-28.9327
1.45	-0.04227	-27.3156	-0.03807	-27.8475
1.5	-0.04605	-26.0947	-0.04054	-26.7773
1.55	-0.05064	-24.8913	-0.04355	-25.7188
1.6	-0.05629	-23.7033	-0.04724	-24.6693
1.65	-0.0633	-22.5286	-0.05181	-23.6259
1.7	-0.07209	-21.3655	-0.05751	-22.5859
1.75	-0.08318	-20.2125	-0.06467	-21.5469
1.8	-0.09724	-19.0683	-0.0737	-20.5066
1.85	-0.11515	-17.9321	-0.08516	-19.4626
1.9	-0.13804	-16.8033	-0.09978	-18.413
1.95	-0.16737	-15.6817	-0.11853	-17.3559
2	-0.20503	-14.5679	-0.1427	-16.2895
2.05	-0.25341	-13.4628	-0.17402	-15.2124
2.1	-0.3156	-12.3682	-0.21484	-14.1237
2.15	-0.39548	-11.2866	-0.26835	-13.0229
2.2	-0.4979	-10.2218	-0.33892	-11.9104
2.25	-0.62886	-9.17867	-0.43252	-10.7877
2.3	-0.79556	-8.16327	-0.55735	-9.658
2.35	-1.00647	-7.18315	-0.7246	-8.52677
2.4	-1.27113	-6.24697	-0.94945	-7.40248

2.45	-1.59979	-5.36425	-1.25219	-6.29745
2.5	-2.00271	-4.54464	-1.65919	-5.22847
2.55	-2.48922	-3.79708	-2.20358	-4.21683
2.6	-3.06656	-3.1287	-2.92483	-3.28707
2.65	-3.73889	-2.54383	-3.86711	-2.46406
2.7	-4.50645	-2.04331	-5.07676	-1.76828
2.75	-5.36534	-1.62444	-6.60088	-1.21094
2.8	-6.30783	-1.28132	-8.49068	-0.79085
2.85	-7.32302	-1.00579	-10.8145	-0.49483
2.9	-8.39773	-0.78845	-13.6898	-0.30148
2.95	-9.5172	-0.61967	-17.3607	-0.18619
3	-10.6656	-0.49033	-22.4526	-0.12556
3.05	-11.8264	-0.39232	-31.4277	-0.09992
3.1	-12.9822	-0.31871	-37.8307	-0.09427
3.15	-14.1149	-0.26384	-27.892	-0.098
3.2	-15.2058	-0.22317	-24.5079	-0.10428
3.25	-16.2356	-0.19319	-22.9894	-0.10915
3.3	-17.1856	-0.17118	-22.4638	-0.11079
3.35	-18.0387	-0.15511	-22.6604	-0.10886
3.4	-18.7805	-0.14347	-23.5316	-0.10404
3.45	-19.4008	-0.13513	-25.2031	-0.09761
3.5	-19.8949	-0.12927	-28.1112	-0.09118
3.55	-20.2641	-0.1253	-33.8384	-0.08645
3.6	-20.5152	-0.12277	-57.0328	-0.08502
3.65	-20.6596	-0.12137	-31.9419	-0.08829
3.7	-20.7118	-0.12085	-25.8858	-0.09732
3.75	-20.6878	-0.12104	-22.2312	-0.11285
3.8	-20.6038	-0.1218	-19.62	-0.13519
3.85	-20.4748	-0.12303	-17.6087	-0.16427
3.9	-20.3143	-0.12463	-15.9947	-0.19968
3.95	-20.133	-0.12656	-14.6667	-0.24072
4	-19.9374	-0.12885	-13.5553	-0.28658
4.05	-19.7036	-0.13314	-12.6035	-0.338
4.1	-19.6265	-0.13603	-11.8583	-0.38721
4.15	-19.4333	-0.13537	-11.1736	-0.43741
4.2	-19.2677	-0.13746	-10.5991	-0.48882
4.25	-19.1226	-0.13956	-10.1141	-0.53849
4.3	-18.998	-0.14154	-9.70687	-0.58521
4.35	-18.8944	-0.14331	-9.36841	-0.62795
4.4	-18.8129	-0.14484	-9.09178	-0.66579
4.45	-18.7539	-0.14609	-8.87144	-0.69798
4.5	-18.7178	-0.14704	-8.70307	-0.72389
4.55	-18.7049	-0.14767	-8.58332	-0.74309
4.6	-18.7154	-0.14797	-8.50972	-0.75527
4.65	-18.7492	-0.14794	-8.48057	-0.76029
4.7	-18.8064	-0.1476	-8.49491	-0.75814
4.75	-18.8871	-0.14694	-8.55245	-0.74896
4.8	-18.991	-0.146	-8.65354	-0.73302

4.85	-19.1184	-0.14479	-8.79925	-0.7107
4.9	-19.269	-0.14336	-8.99132	-0.68253
4.95	-19.4431	-0.14172	-9.23228	-0.64913
5	-19.6408	-0.13991	-9.52548	-0.61121
5.05	-19.8622	-0.13796	-9.8753	-0.56958
5.1	-20.1076	-0.13591	-10.2873	-0.52512
5.15	-20.3773	-0.1338	-10.7684	-0.47877
5.2	-20.6718	-0.13164	-11.3277	-0.43152
5.25	-20.9917	-0.12948	-11.9765	-0.38438
5.3	-21.3376	-0.12734	-12.7297	-0.33834
5.35	-21.7105	-0.12525	-13.6076	-0.29438
5.4	-22.1113	-0.12323	-14.6376	-0.25343
5.45	-22.5414	-0.1213	-15.8597	-0.21634
5.5	-23.0024	-0.11948	-17.3348	-0.18386
5.55	-23.4961	-0.11779	-19.1626	-0.15661
5.6	-24.0248	-0.11622	-21.5248	-0.13507
5.65	-24.5915	-0.1148	-24.8095	-0.11956
5.7	-25.1997	-0.11353	-30.1178	-0.11023
5.75	-25.8538	-0.1124	-42.6605	-0.10707
5.8	-26.5594	-0.11144	-32.917	-0.1099
5.85	-27.3235	-0.11063	-26.4466	-0.11838
5.9	-28.1553	-0.10997	-22.8332	-0.13205
5.95	-29.0668	-0.10946	-20.3651	-0.15033
6	-30.0739	-0.10909	-18.5207	-0.17253
6.05	-31.1987	-0.10887	-17.0725	-0.19791
6.1	-32.4721	-0.10879	-15.9009	-0.2257
6.15	-33.9399	-0.10884	-14.935	-0.25509
6.2	-35.6722	-0.10903	-14.1294	-0.2853
6.25	-37.7824	-0.10934	-13.453	-0.31554
6.3	-40.4642	-0.10977	-12.8838	-0.34509
6.35	-44.0239	-0.11032	-12.4057	-0.37329
6.4	-48.2666	-0.11099	-12.0064	-0.39954
6.45	-48.2413	-0.11177	-11.6765	-0.4233
6.5	-44.0686	-0.11265	-11.4086	-0.44413
6.55	-40.604	-0.11364	-11.1972	-0.46169
6.6	-38.0073	-0.11472	-11.0377	-0.47569
6.65	-35.9725	-0.1159	-10.9267	-0.48595
6.7	-34.3071	-0.11718	-10.8614	-0.49239
6.75	-32.8972	-0.11857	-10.8399	-0.49497
6.8	-31.6726	-0.12007	-10.8609	-0.49376
6.85	-30.5868	-0.12168	-10.9234	-0.48891
6.9	-29.6083	-0.12343	-11.0271	-0.48061
6.95	-28.7143	-0.12531	-11.1721	-0.46914
7	-27.8881	-0.12734	-11.3587	-0.4548
7.05	-27.1171	-0.12954	-11.5877	-0.43798
7.1	-26.3918	-0.13193	-11.8605	-0.41907
7.15	-25.7044	-0.13452	-12.1785	-0.39851
7.2	-25.049	-0.13732	-12.5437	-0.37675

7.25	-24.4208	-0.14037	-12.9586	-0.35425
7.3	-23.816	-0.14369	-13.4258	-0.33145
7.35	-23.2316	-0.14729	-13.9487	-0.30879
7.4	-22.665	-0.15121	-14.5311	-0.28668
7.45	-22.1142	-0.15547	-15.1775	-0.26549
7.5	-21.5777	-0.16011	-15.8931	-0.24555
7.55	-21.0543	-0.16514	-16.6838	-0.22714
7.6	-20.5429	-0.1706	-17.5569	-0.21045
7.65	-20.0429	-0.17653	-18.5205	-0.19564
7.7	-19.5536	-0.18295	-19.5846	-0.1828
7.75	-19.0747	-0.18991	-20.761	-0.17193
7.8	-18.6059	-0.19742	-22.0637	-0.163
7.85	-18.1471	-0.20553	-23.5093	-0.15591
7.9	-17.6982	-0.21426	-25.1176	-0.15051
7.95	-17.2593	-0.22365	-26.9111	-0.14661
8	-16.8304	-0.23372	-28.9133	-0.14402
8.05	-16.4116	-0.2445	-31.1438	-0.14252
8.1	-16.0033	-0.25601	-33.6027	-0.14187
8.15	-15.6054	-0.26826	-36.2333	-0.14188
8.2	-15.2184	-0.28128	-38.8392	-0.14237
8.25	-14.8423	-0.29507	-40.9642	-0.14321
8.3	-14.4775	-0.30963	-41.9032	-0.1443
8.35	-14.1239	-0.325	-41.1637	-0.14565
8.4	-13.7816	-0.34121	-39.0238	-0.14732
8.45	-13.4501	-0.35842	-36.2182	-0.14956
8.5	-13.128	-0.37734	-33.2775	-0.15309
8.55	-12.8151	-0.40397	-30.3482	-0.16359
8.6	-13.13	-0.48813	-33.773	-0.28889
8.65	-12.3767	-0.42837	-26.9814	-0.1733
8.7	-12.078	-0.44533	-24.636	-0.17633
8.75	-11.8216	-0.46391	-22.7253	-0.18566
8.8	-11.5875	-0.48259	-21.0461	-0.19868
8.85	-11.3719	-0.50103	-19.5418	-0.21538
8.9	-11.1741	-0.51902	-18.1835	-0.23604
8.95	-10.9943	-0.53633	-16.9519	-0.26098
9	-10.8329	-0.55274	-15.8329	-0.29044
9.05	-10.6905	-0.568	-14.8155	-0.32452
9.1	-10.5681	-0.58184	-13.8907	-0.36322
9.15	-10.4667	-0.59399	-13.0514	-0.40631
9.2	-10.3875	-0.60416	-12.2914	-0.4534
9.25	-10.3322	-0.61206	-11.6057	-0.50389
9.3	-10.3026	-0.6174	-10.99	-0.55699
9.35	-10.3009	-0.61989	-10.4408	-0.61168
9.4	-10.3298	-0.61924	-9.9552	-0.66678
9.45	-10.3926	-0.61521	-9.53087	-0.72094
9.5	-10.4933	-0.60755	-9.16618	-0.77265
9.55	-10.6372	-0.59609	-8.86023	-0.82031
9.6	-10.8304	-0.58069	-8.61295	-0.86222

9.65	-11.0813	-0.56131	-8.42531	-0.89667
9.7	-11.4002	-0.53801	-8.29951	-0.92191
9.75	-11.8011	-0.51097	-8.23945	-0.93627
9.8	-12.3028	-0.48056	-8.25118	-0.93822
9.85	-12.9312	-0.44736	-8.34374	-0.92641
9.9	-13.7239	-0.41224	-8.53038	-0.89982
9.95	-14.7375	-0.37638	-8.83049	-0.85793
10	-16.0634	-0.3414	-9.27281	-0.80094
10.05	-17.8628	-0.30938	-9.90108	-0.73007
10.1	-20.462	-0.28302	-10.7846	-0.64802
10.15	-24.679	-0.26564	-12.0408	-0.55953
10.2	-32.8645	-0.26128	-13.8888	-0.47209
10.25	-28.5386	-0.27475	-16.8112	-0.39674
10.3	-21.4035	-0.3115	-22.226	-0.34879
10.35	-17.1565	-0.37759	-30.227	-0.34804
10.4	-14.1412	-0.47939	-19.5615	-0.41802
10.45	-11.8076	-0.62323	-13.8526	-0.58365
10.5	-9.91858	-0.81491	-10.2732	-0.86705
10.55	-8.3523	-1.05917	-7.74915	-1.2826
10.6	-7.03749	-1.35922	-5.88874	-1.833
10.65	-5.92802	-1.71633	-4.49681	-2.50854
10.7	-4.99104	-2.12969	-3.45209	-3.28983
10.75	-4.20103	-2.59653	-2.66827	-4.1526
10.8	-3.5369	-3.11243	-2.08022	-5.07241
10.85	-2.98041	-3.67182	-1.63825	-6.02774
10.9	-2.51554	-4.26853	-1.30479	-7.00141
10.95	-2.12815	-4.89626	-1.05176	-7.98066
11	-1.8059	-5.54901	-0.85844	-8.95658
11.05	-1.53807	-6.22131	-0.7096	-9.92334
11.1	-1.3155	-6.90838	-0.59408	-10.8774
11.15	-1.13045	-7.6062	-0.5037	-11.8169
11.2	-0.9764	-8.31148	-0.43241	-12.7412
11.25	-0.84796	-9.02161	-0.37576	-13.6504
11.3	-0.74066	-9.7346	-0.3304	-14.5452
11.35	-0.65081	-10.449	-0.29385	-15.427
11.4	-0.57539	-11.1638	-0.2642	-16.297
11.45	-0.51192	-11.8783	-0.24002	-17.1569
11.5	-0.45836	-12.5923	-0.22019	-18.0088
11.55	-0.41305	-13.3058	-0.20387	-18.8545
11.6	-0.37461	-14.0188	-0.19038	-19.6963
11.65	-0.34193	-14.7318	-0.17921	-20.5365
11.7	-0.31406	-15.4455	-0.16993	-21.3778
11.75	-0.29026	-16.1604	-0.16223	-22.223
11.8	-0.26988	-16.8776	-0.15583	-23.0752
11.85	-0.25241	-17.598	-0.15053	-23.9381
11.9	-0.2374	-18.323	-0.14616	-24.8157
11.95	-0.22449	-19.054	-0.14258	-25.7128
12	-0.21339	-19.7926	-0.13968	-26.6349

12.05	-0.20383	-20.5408	-0.13738	-27.5889
12.1	-0.19561	-21.3008	-0.1356	-28.5833
12.15	-0.18855	-22.0754	-0.13428	-29.6286
12.2	-0.1825	-22.8679	-0.13338	-30.7388
12.25	-0.17734	-23.6824	-0.13287	-31.9325
12.3	-0.17296	-24.524	-0.13272	-33.2358
12.35	-0.16927	-25.3995	-0.13292	-34.6872
12.4	-0.16623	-26.3183	-0.13346	-36.3464
12.45	-0.16377	-27.2935	-0.13437	-38.3147
12.5	-0.16189	-28.3449	-0.13568	-40.7836
12.55	-0.1606	-29.5042	-0.13747	-44.1839
12.6	-0.16002	-30.8276	-0.13987	-49.817
12.65	-0.1604	-32.4262	-0.14321	-59.4757
12.7	-0.16251	-34.5623	-0.14826	-48.0978
12.75	-0.16878	-38.0563	-0.15729	-41.3279
12.8	-0.19022	-45.7774	-0.18019	-35.6551
12.85	-0.33392	-31.7991	-0.31034	-28.061
12.9	-0.81873	-21.3632	-0.68129	-22.539
12.95	-0.23049	-28.2037	-0.24876	-30.1393
13	-0.18322	-31.4935	-0.41422	-27.7717
13.05	-0.1812	-33.1197	-0.45698	-27.2305
13.1	-0.1862	-40.2903	-0.21122	-33.0218
13.15	-0.16601	-41.5355	-0.18713	-34.392
13.2	-0.16397	-43.8649	-0.18319	-34.5062
13.25	-0.16388	-47.2075	-0.18428	-34.217
13.3	-0.1645	-52.2143	-0.18733	-33.7986
13.35	-0.16552	-62.5154	-0.19141	-33.3514
13.4	-0.16683	-59.9459	-0.1961	-32.9157
13.45	-0.16836	-52.2833	-0.20123	-32.5083
13.5	-0.17008	-48.5239	-0.20668	-32.1362
13.55	-0.17197	-46.1229	-0.21239	-31.8022
13.6	-0.17402	-44.4152	-0.21831	-31.5072
13.65	-0.17621	-43.131	-0.22441	-31.2515
13.7	-0.17855	-42.1349	-0.23062	-31.0355
13.75	-0.18102	-41.3495	-0.23691	-30.8595
13.8	-0.18362	-40.7269	-0.24323	-30.7242
13.85	-0.18636	-40.2355	-0.24954	-30.6305
13.9	-0.18921	-39.8537	-0.25579	-30.5797
13.95	-0.19218	-39.566	-0.26192	-30.5733
14	-0.19527	-39.3614	-0.26789	-30.6133
14.05	-0.19846	-39.2313	-0.27365	-30.7022
14.1	-0.20174	-39.1693	-0.27915	-30.8426
14.15	-0.2051	-39.1698	-0.28435	-31.038
14.2	-0.20855	-39.237	-0.28921	-31.2923
14.25	-0.21213	-39.3794	-0.29369	-31.61
14.3	-0.21583	-39.6007	-0.29777	-31.9967
14.35	-0.21966	-39.9068	-0.30142	-32.4591
14.4	-0.22363	-40.3062	-0.30463	-33.0051

14.45	-0.22775	-40.811	-0.3074	-33.6449
14.5	-0.23201	-41.4381	-0.30973	-34.3914
14.55	-0.23644	-42.2112	-0.31163	-35.2613
14.6	-0.24104	-43.1647	-0.31312	-36.277
14.65	-0.24581	-44.3499	-0.31422	-37.4698
14.7	-0.25077	-45.849	-0.31494	-38.8855
14.75	-0.25591	-47.8034	-0.31532	-40.5942
14.8	-0.26123	-50.4908	-0.31539	-42.7123
14.85	-0.26673	-54.5757	-0.3152	-45.4554
14.9	-0.27239	-62.1965	-0.31478	-49.296
14.95	-0.27821	-62.9347	-0.31419	-55.5941
15	-0.2842	-54.8745	-0.31347	-63.8338
15.05	-0.29039	-50.6296	-0.31266	-54.8117
15.1	-0.2968	-47.9028	-0.31184	-50.1361
15.15	-0.30344	-46.0042	-0.31104	-47.5379
15.2	-0.31031	-44.6708	-0.31033	-46.0091
15.25	-0.31743	-43.7964	-0.30977	-45.222
15.3	-0.32481	-43.3584	-0.30944	-45.0924
15.35	-0.33249	-43.4051	-0.30941	-45.6999
15.4	-0.34048	-44.0851	-0.3098	-47.3658
15.45	-0.34886	-45.7655	-0.31071	-51.0822
15.5	-0.35769	-49.5084	-0.31232	-58.9683
15.55	-0.36711	-58.1676	-0.31479	-49.9448
15.6	-0.37731	-48.2245	-0.31835	-42.6688
15.65	-0.3886	-40.7344	-0.32335	-37.805
15.7	-0.40144	-35.7392	-0.33026	-34.0047
15.75	-0.41654	-31.8207	-0.33977	-30.793
15.8	-0.435	-28.4991	-0.35287	-27.9506
15.85	-0.45846	-25.5569	-0.37104	-25.3571
15.9	-0.48945	-22.8771	-0.39645	-22.9388
15.95	-0.53175	-20.39	-0.43243	-20.6469
16	-0.59112	-18.0517	-0.48408	-18.4474
